# Genome-wide identification of NAC transcription factors and regulation of monoterpenoid indole alkaloid biosynthesis in *Catharanthus roseus*

**DOI:** 10.3389/fpls.2023.1286584

**Published:** 2023-12-20

**Authors:** Jawad Ahmed, Yasar Sajjad, Mansour K. Gatasheh, Khalid Elfaki Ibrahim, Muhammad Huzafa, Sabaz Ali Khan, Chen Situ, Arshad Mehmood Abbasi, Amjad Hassan

**Affiliations:** ^1^ Department of Biotechnology, COMSATS University Islamabad, Abbottabad, Pakistan; ^2^ Institute for Global Food Security, School of Biological Sciences, Queens University Belfast, Belfast, United Kingdom; ^3^ Department of Biochemistry, College of Science, King Saud University, Riyadh, Saudi Arabia; ^4^ Department of Zoology, College of Science, King Saud University, Riyadh, Saudi Arabia; ^5^ Department of Plant Sciences, Quaid-e-Azam University, Islamabad, Pakistan, Pakistan; ^6^ Department of Environmental Sciences, COMSATS University, Islamabad, Abbottabad, Pakistan

**Keywords:** elicitation, cell suspension culture, nodal culture, vinblastine, catharanthine, vindoline, auxin

## Abstract

NAC transcription factors (TFs) are crucial to growth and defense responses in plants. Though NACs have been characterized for their role in several plants, comprehensive information regarding their role in *Catharanthus roseus*, a perennial ornamental plant, is lacking. Homology modelling was employed to identify and characterize NACs in *C. roseus*. *In-vitro* propagation of *C. roseus* plants was carried out using cell suspension and nodal culture and were elicited with two auxin-antagonists, 5-fluoro Indole Acetic Acid (5-F-IAA) and α-(phenyl ethyl-2-oxo)-Indole-Acetic-Acid (PEO-IAA) for the enhanced production of monoterpenoid indole alkaloids (MIAs) namely catharanthine, vindoline, and vinblastine. Analyses revealed the presence of 47 putative CrNAC genes in the *C. roseus* genome, primarily localized in the nucleus. Phylogenetic analysis categorized these CrNACs into eight clusters, demonstrating the highest synteny with corresponding genes in *Camptotheca acuminata*. Additionally, at least one defense or hormone-responsive cis-acting element was identified in the promoter region of all the putative *CrNAC*s. Of the two elicitors, 5-F-IAA was effective at 200 µM to elicit a 3.07-fold increase in catharanthine, 2.76-fold in vindoline, and 2.4-fold in vinblastine production in nodal culture. While a relatively lower increase in MIAs was recorded in suspension culture. Validation of RNA-Seq by qRT-PCR showed upregulated expression of stress-related genes (*CrNAC-07* and *CrNAC-24*), and downregulated expression of growth-related gene (*CrNAC-25*) in elicited nodal culture of *C. roseus*. Additionally, the expression of genes involved in the biosynthesis of MIAs was significantly upregulated upon elicitation. The current study provides the first report on the role of CrNACs in regulating the biosynthesis of MIAs.

## 1 Introduction

*Catharanthus roseus* has garnered significant attention in the past decade due to its abundant repertoire of diverse bioactive compounds. This unique characteristic has positioned it as a “model non-model” plant for alkaloid and secondary metabolite research (De Bernonville et al., 2017). The plant is best characterized for monoterpenoid indole alkaloids (MIAs) biosynthesis, of which vincristine and vinblastine possess notable antineoplastic properties (Carqueijeiro et al., 2018). These alkaloids are used in the treatment of various types of cancer including leukaemia and Hodgkin’s lymphoma (Chagas and Alisaraie, 2019). Vinblastine and vincristine inhibit mitosis by targeting microtubules during metaphase, ultimately resulting in cell death (Chi et al., 2015). Being a dimeric indole alkaloid, vinblastine is synthesized through coupling of its monomers i.e., vindoline and catharanthine (Qu et al., 2015). However, being a secondary metabolite, the exploitation of vinblastine at a commercial scale is challenging.

Elicitation has emerged as a practical approach with promising potential in improving secondary metabolite production. This method entails the activation of plant defense to modulate biochemical reactions subsequently enhancing the transcription of genes responsible for secondary metabolite biosynthesis (Golkar et al., 2019). The use of elicitors has demonstrated a significant elevation of plant secondary metabolite concentration in *in-vitro* cultures of various species (Ming et al., 2013; Sabater-Jara et al., 2014; Vidal-Limon et al., 2018). The response instigated by the application of elicitors does not exhibit a uniform pattern and instead varies depending on several factors including concentration, nature, and exposure time (Murthy et al., 2014). Initially, plasma membrane-localized receptors perceive elicitors resulting in the activation of several signaling pathways involving ion channels, G-proteins, and protein kinases (Ramirez-Estrada et al., 2016). Afterwards, these signaling pathways transmit the signals induced by elicitors to downstream components, resulting in a range of cellular responses. These responses include the generation of reactive oxygen species (ROS), secretion of signaling hormones, and the regulation of gene expression through the activation of specific transcription factors (TFs). Ultimately, these processes converge in the biosynthesis of secondary metabolites (Ramirez-Estrada et al., 2016).

Maintaining a delicate balance between growth and secondary metabolite production is one of the key challenges in elicitor-induced biosynthesis of secondary metabolites. Recent studies on the role of NAC (named after three genes **N**AM, **A**TAF, and **C**UC) transcription factors in the regulation of flower formation (Liu et al., 2023), cotyledon and embryo development (Wang et al., 2022), hormone signaling (Jensen et al., 2010), maintenance of shoot apical meristem (Takada et al., 2001), leaf senescence (Kim et al., 2016) and response to pathogen infection (Bian et al., 2021) has established their potential as regulators of multiple cellular processes. Nonetheless, the role of NACs’ regulation of monoterpenoid indole alkaloids remained to be characterized.

Previous studies report upregulated expression of several NAC TFs in response to oxidative stress (De Clercq et al., 2013; Wang et al., 2016). Interestingly, oxidative stress has been reported as a key player in the elicitor-induced biosynthesis of alkaloids (Matsuura et al., 2014). For instance, vinblastine accumulation was proportionate with the corresponding activity of peroxidases induced by polyethylene glycol (Liu et al., 2017). Based on this evidence, we hypothesized that moderate oxidative stress could positively regulate both expressions of NACs together with enhanced accumulation of MIAs. To test this hypothesis, we screened small phytohormone molecules capable of regulating the expression of NACs and genes involved in MIAs biosynthesis. Among the various phytohormones examined, auxins were well documented to antagonize the production of plant secondary metabolites by reducing the activity of peroxidases (El-Sayed and Verpoorte, 2007; Amoo and van Staden, 2013; Mekky et al., 2018). Therefore, we selected two auxin antagonists namely 5-F-IAA and PEO-IAA for their application in *in-vitro* cultures of *C. roseus* to evaluate their efficacy as elicitors of MIAs biosynthesis and inducers of NAC expression. This is the first report that aims to unravel the involvement of NACs in the regulation of gene expression associated with the biosynthesis of MIAs.

## 2 Materials and methods

### 2.1 Sequence retrieval

The protein, coding sequence (CDS), and genomic sequences of *C. roseus* NAC transcription factors were downloaded from Medicinal Plant Genomic Resources (MPGR, http://medicinalplantgenomics.msu.edu/4058.shtml). The HMM profile of the NAM domain (PF02365) was retrieved from the Pfam database (http://pfam.xfam.org) and was used to search NAC TFs in the *C. roseus* protein database using HMMER 3.0 software with an E-value setting to 1e-2. Furthermore, *Arabidopsis thaliana* NAC (Accession No. NP_171609.1) was also used as a query sequence to perform BLASTP (https://blast.ncbi.nlm.nih.gov/Blast.cgi) against the *C. roseus* protein database. The resultant proteins were screened for the presence of the NAM domain using SMART (http://smart.embl-heidelberg.de/, Schultz et al., 1998), Pfam and NCBI-CDD database (http://www.ncbi.nlm.nih.gov/cdd/, Lu et al., 2020) and the redundant proteins were manually removed.

### 2.2 Structure and properties of CrNAC family

The physicochemical properties of CrNACs were predicted by ExPASy (http://web.expasy.org/protparam/, Gasteiger et al., 2003). The gene structure analysis was performed using the default parameters of the gene structure display server (http://gsds.gao-lab.org/, Hu B. et al., 2015). MEME software (http://meme-suite.org/, Bailey et al., 2015) was used to predict the motifs present using default parameters, with the maximum number of motifs set at 10. The subcellular localization of CrNAC TFs was predicted through WoLF PSORT (https://wolfpsort.hgc.jp/, Horton et al., 2007). A sequence of 1500 bp upstream of each NAC gene was retrieved from MPGR and submitted to the PlantCARE database (http://bioinformatics.psb.ugent.be/webtools/plantcare/html/, Lescot et al., 2002) for predicting cis-acting elements.

### 2.3 Multiple sequence alignment and phylogenetic analysis

A phylogenetic tree using protein sequences of *C. roseus*, *Arabidopsis thaliana*, and *Oryza sativa* was constructed using sequences retrieved from Phytozome. These sequences were aligned with CrNACs using ClustalW with standard settings. Subsequently, an unrooted phylogenetic tree was established by MEGA 7 using the Maximum likelihood method with 1,000 bootstrap repeats (Kumar et al., 2018).

### 2.4 Chromosome localization, synteny analysis, and Ka/Ks calculation

The sizes of *C. roseus* chromosomes and the position of CrNAC genes on them were extracted from the NCBI *C. roseus* genome browser. MCScanX tool was used to analyze gene duplication events, using default parameters (Wang et al., 2012), A collinearity analysis was accomplished using genome files of *C. roseus*, *A. thaliana*, *Mitragyna speciosa*, *Gelsemium sempervirens*, and *Camptotheca acuminata*. The Ka/Ks ratio was calculated using TBtools (Chen et al., 2020).

### 2.5 Gene expression and co-expression network analysis

The available RNA-Seq data was retrieved from the CathaCyc and MPGR consortium. Previously, a detailed gene expression analysis of *C. roseus* performed by Van et al., 2013, (SRA030483) was used in the current study. The expression data of CrNAC TFs was analyzed in plant organs (root, stem, flower, immature leaf, and mature leaf) as well as in cell suspension cultures of *C. roseus* treated with methyl jasmonate (MeJ) and Yeast extract. FPKM values were normalized into log2 values and heat maps were constructed using TBtools software. A co-expression network analysis of CrNAC genes and MIA biosynthetic genes was constructed using Cytoscape V 3.8.2 (Shannon et al., 2003).

### 2.6 Plant material and growth conditions

*C. roseus* plant seeds were first sterilized and germinated on MS basal medium in an LED-based constant climate plant growth chamber (HPP750 Memmert, Germany) set at 25 °C with 16 h photoperiod, 8 h dark, and relative humidity of 50% was used for growing plants. Plantlets germinated *in-vitro* were shifted to the pots containing compost and placed in the plant growth room under the same conditions stated above for further growth and hardening. Two-month-old plants were used for initiating nodal and cell suspension culture. For callus induction, mature leaves of *C. roseus* were aseptically cut into 1 cm^2^ pieces. The leaf explants were placed on semi-solidified (sucrose, 3%; agar, 0.7%) MS basal medium (pH, 5.7) supplemented with different concentrations (ranging from 0 to 2.0 mg/L) of kinetin along with 2.0 mg/L 1-naphthalene acetic acid (NAA) and 2,4-dichlorophenoxyaceticacid (2,4-D). Four best-responding friable calli were screened for their MIA content. The callus with the highest concentration of MIAs was subsequently used for initiating cell suspension culture. The friable callus was chopped into small pieces and added to flasks containing liquid MS basal medium supplemented with 1.5 mg/L kinetin and 2.0 mg/L NAA. These flasks containing chopped calli were then placed on a rotary shaker (New Brunswick Innova 44) set at 110 rpm and 27 °C under white florescent light of 40 µmolm^-2^s^-1^ with a photoperiod of 16 h. The dispersed cells were sub-cultured every week to obtain a homogenous stable cell suspension culture. A completely homogenized cell suspension culture obtained was then used for elicitor application. To initiate nodal culture, nodes of approximately 1.0 cm were cut and placed on a semi-solid MS medium containing varying concentrations of 6-benzyl aminopurine (BAP) and NAA to stimulate shooting, and indole butyric acid (IBA) to stimulate rooting (Mustafa et al., 2011).

### 2.7 Growth measurement of cell suspension and nodal culture

The growth of plant cell suspension culture was measured by counting the viability of cells. 50 µL of cells were harvested daily and stained with Evan’s blue for 1 min and then washed with water to remove excess dye. A hemocytometer was used to count viable cells (Mustafa et al., 2011).

### 2.8 Application of elicitors on *in-vitro* cultures

The experiment employed four different concentrations (0, 50, 100, and 200 µM) each of 5-F-IAA and PEO-IAA dissolved in 0.1% Dimethyl Sulfoxide (DMSO). For cell suspension culture, the exposure time of the elicitor was 96 h which was applied during the log phase of the growth on day 6^th^. On the other hand, in nodal culture, one-month-old *C. roseus* plants were sub-cultured on a medium containing the elicitor and were harvested on the fourth week post elicitation.

### 2.9 Extraction and isolation of MIAs

The cells from the suspension culture and leaves from the nodal culture were dried using a freeze-dryer (Lablyo, UK) at -50 °C for three days. The freeze-dried samples were milled in a planetary ball mill (Retsch 400) at 500 rpm for 3 mins. The powder was sonicated for 60 min in 10 mL 70% methanol and extracted thrice. The mixture was centrifuged at 5000 rpm for 20 min. 10 mL water acidified with 3% HCl was added to the supernatant and washed thrice with 30 mL n-hexane. The aqueous portion was basified with ammonia, pH was adjusted to 8.5, extracted three times with 30 mL chloroform, and washed with water. The extract was dried under a nitrogen gas evaporator (TurboVap, Biotage) and redissolved in 2 mL of 70% methanol. The extracted MIAs were run on Alliance HPLC with PDA detector 2996 (Waters, Milford, UK). Pure fractions of each of the three MIAs were collected, dried in a nitrogen gas evaporator (TurboVap, Biotage), re-dissolved, and quantified on LC-MS (Gupta et al., 2005).

### 2.10 Quantification of MIAs by LC-MS

MIAs of *C. roseus* were determined by UPLC-MS coupled to a QDa mass spectrometer in which the Electrospray Ionization (ESI) source was controlled by Masslynx 4.2 (Waters, UK). Six different concentrations of each MIA standards-vinblastine, vindoline, and catharanthine were used to make a standard curve. Three ACQUITY UPLC C18 columns-BEH, HSS T3, and CSH of dimensions (150 mm × 2.1 mm, 1.8 µm) with column temperatures ranging from 25°C to 45°C were used to optimize the separation. The elution profile is given in **Supplementary Table S1**. Ionization was carried out in a positive mode to confirm the masses of MIAs in the samples. The sample cone and capillary voltage were 30 V and 30,000 V, respectively. Desorption Electrospray Ionization (DESI) was used to confirm the masses of daughter fragments of the parent ions. The matrix effect was assessed by spiking the standard over the analyte (De Bernonville et al., 2017).

### 2.11 RNA extraction, cDNA synthesis, and qRT-PCR

Snap-frozen leaves from the nodal culture weighing 100 mg were ground to powder using TissueLyser LT (Qiagen, UK). Total RNA was extracted from the powdered leaf samples using TRIzol reagent (Thermo Fischer Scientific, US) and quantified on Nanodrop 1000 (Thermo Scientific). The integrity was confirmed through gel electrophoresis using 1.5% agarose. The extracted total RNA was treated with DNase-I before the first strand of cDNA was synthesized using the WizScript™ cDNA synthesis kit (wizbiosolutions). Gene-specific primers were designed using Primer3 software (**Supplementary Table S2**). qRT-PCR was performed using the designed primers and 2x iTaq Universal SYBR Green super mix. Components of the reaction mixture are presented in **Supplementary Table S3**. The CT values obtained were used to calculate the relative expression of the genes using the formula 2^-ΔΔCT^ developed by Livak and Schmittgen (2001).

### 2.12 Experimental design and statistical analysis

A completely randomized design (CRD) consisting of nine biological replicates each in cell suspension and nodal culture was adopted for the experiment. Additionally, LC-MS and qRT-PCR were performed using three technical replicates. Differences in means among treatments were assessed using analysis of variance (ANOVA) and Tukey HSD test, using IBM SPSS v.26.0 and Statistix 8.1. To find out the association between MIA production and biomass, Pearson’s correlation was performed. Graphs for *in-silico* analyses were constructed using TBtools (Chen et al., 2020) whereas graphs for expression and metabolite profiling were constructed using GraphPad Prism 9.0. All the data are expressed as mean ± SD.

## 3 Results

### 3.1 Identification of NAC TFs in *C. roseus* genome and their phylogenetic clustering

In this study, 47 NAC transcription factors were identified in the *C. roseus* genome based on the presence of the NAM domain. **Table 1** shows the fundamental characteristics of CrNAC-01 to CrNAC-47. NAC proteins varied in length from 146 amino acids (CrNAC-45) to 607 amino acids (CrNAC-31) with molecular weight from 17.4 kDa (CrNAC-45) to 67.7 kDa (CrNAC-31). The predicted subcellular localization revealed that CrNACs were primarily localized in the nucleus followed by the cytoplasm.

**Table 1:** Physiochemical properties of identified NAC TFs in *C. roseus*.

GeneName	Transcript ID	Protein Length	Molecular Weight	pI	GRAVY	Sub-cellular localization
*CrNAC-01*	CRO_T003042	353	38555.38	8.89	-0.624	Nucleus
*CrNAC-02*	CRO_T016002	281	32658.87	6.45	-0.754	Cytoplasm
*CrNAC-03*	CRO_T006367	246	28480.58	4.83	-0.845	Nucleus
*CrNAC-04*	CRO_T017196	243	31567.51	5.43	-0.717	Nucleus
*CrNAC-05*	CRO_T025185	330	37681.46	6.23	-0.74	Nucleus
*CrNAC-06*	CRO_T018388	314	36408.88	6.67	-0.879	Nucleus
*CrNAC-07*	CRO_T025827	570	65082.19	7.1	-0.611	Nucleus
*CrNAC-08*	CRO_T024772	199	23576.97	8.74	-0.666	Peroxisomes
*CrNAC-09*	CRO_T001050	351	39571.45	8.78	-0.674	Nucleus
*CrNAC-10*	CRO_T031065	397	44086.46	8.53	-0.638	Nucleus
*CrNAC-11*	CRO_T001821	362	40712.43	7.79	-0.709	Nucleus
*CrNAC-12*	CRO_T003414	343	39132.98	6.17	-0.762	Nucleus
*CrNAC-13*	CRO_T004614	548	60683.22	4.58	-0.516	Vacuole
*CrNAC-14*	CRO_T021116	387	43956.95	5.7	-0.767	Nucleus
*CrNAC-15*	CRO_T022570	181	20847.67	6.59	-0.653	Cytoplasm
*CrNAC-16*	CRO_T013893	372	42064.13	6.76	-0.734	Nucleus
*CrNAC-17*	CRO_T019171	372	41946.97	6.16	-0.66	Nucleus
*CrNAC-18*	CRO_T019786	425	48420.99	6.23	-0.708	Nucleus
*CrNAC-19*	CRO_T013934	393	44043.01	8.5	-0.523	Cytoplasm
*CrNAC-20*	CRO_T004613	388	43333.53	5.28	-0.728	Chloroplast
*CrNAC-21*	CRO_T031948	257	29137.94	9.15	-0.67	Nucleus
*CrNAC-22*	CRO_T017453	344	39131.5	7.04	-0.884	Nucleus
*CrNAC-23*	CRO_T030053	405	46308.87	5.26	-0.603	Nucleus
*CrNAC-24*	CRO_T014565	357	40489.48	8.63	-0.629	Nucleus
*CrNAC-25*	CRO_T022228	162	18763.7	9.63	-0.426	Cytoplasm
*CrNAC-26*	CRO_T023833	162	18882.91	9.41	-0.463	Cytoplasm
*CrNAC-27*	CRO_T019635	303	33860.54	8.82	-0.402	Chloroplast
*CrNAC-28*	CRO_T016367	250	28541.19	9.23	-0.844	Cytoplasm
*CrNAC-29*	CRO_T015465	389	43666.5	7.22	-0.763	Nucleus
*CrNAC-30*	CRO_T014184	170	19903.87	9.51	-0.725	Cytoplasm
*CrNAC-31*	CRO_T008121	607	67747.81	5.52	-0.763	Nucleus
*CrNAC-32*	CRO_T005173	383	43136.23	5.66	-0.696	Nucleus
*CrNAC-33*	CRO_T002139	491	56204.08	4.61	-0.746	Nucleus
*CrNAC-34*	CRO_T010296	405	46699.31	5.8	-1.075	Nucleus
*CrNAC-35*	CRO_T007448	355	39726.66	7.6	-0.519	Nucleus
*CrNAC-36*	CRO_T012770	270	31103.58	5.82	-0.821	Nucleus
*CrNAC-37*	CRO_T029391	211	24767.23	9.2	-0.716	Nucleus
*CrNAC-38*	CRO_T017858	599	67869.61	5.66	-0.754	Peroxisomes
*CrNAC-39*	CRO_T018335	317	36831.52	5.86	-1.042	Nucleus
*CrNAC-40*	CRO_T031642	196	22562.1	4.79	-0.71	Nucleus
*CrNAC-41*	CRO_T032569	247	28444.15	6.76	-0.791	Nucleus
*CrNAC-42*	CRO_T031013	199	23241.57	9.21	-0.563	Nucleus
*CrNAC-43*	CRO_T019487	177	21237.17	5.08	-0.75	Cytoplasm
*CrNAC-44*	CRO_T014010	231	26540.2	5.22	-0.766	Nucleus
*CrNAC-45*	CRO_T031810	146	17397.8	5.31	-0.54	Cytoplasm
*CrNAC-46*	CRO_T021106	283	32942.26	6.27	-0.829	Cytoplasm
*CrNAC-47*	CRO_T003139	299	33929.34	7.59	-0.373	Nucleus

GRAVY, grand average of hydrophobicity; pI, Point isoelectric.

**Figure 1:**
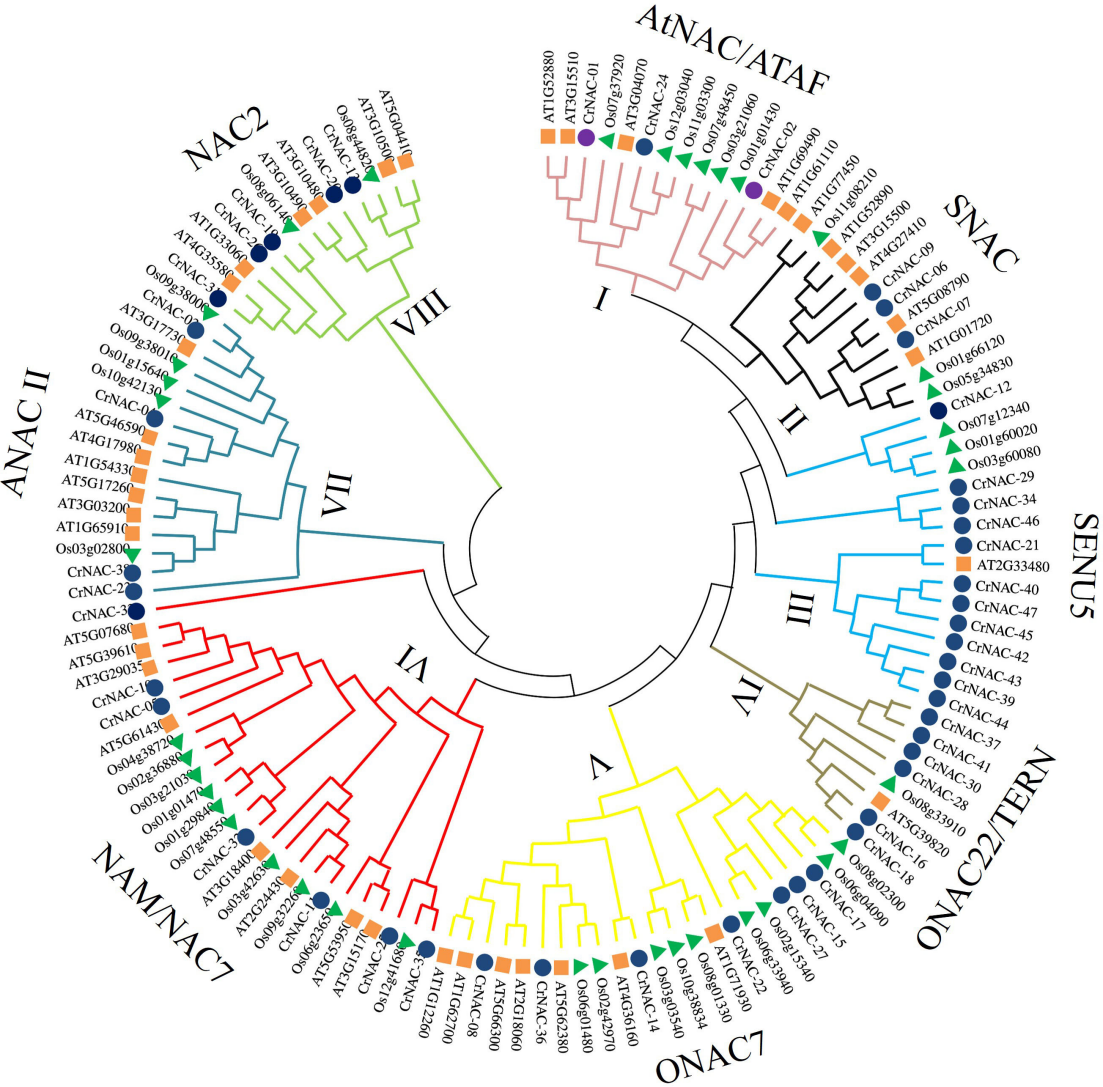
Maximum likelihood phylogenetic tree of NAC proteins of *C. roseus*, *Arabidopsis thaliana*, and *Oryza sativa.* Overall, 47 CrNAC (blue circles), 41 AtNAC (orange rhombus), and 39 OsNAC (green triangles) proteins were divided into 8 groups.

Clustering analysis classified CrNACs into 8 groups based on known NACs in that group. The groups were differentiated as Group-I (AtNAC3/ATAF), Group-II (SNAC), Group-III (SENU5), Group-IV (ONCA22/TERN), Group-V (OsNAC7), Group-VI (NAM/NAC7), Group-VII (ANAC11) and Group-VIII (NAC2) (**Figure 1**). Group-VI was the largest contained 7 CrNACs clustered together with 10 OsNACs and 8 AtNAC, making a total of 25 NAC proteins. In comparison, Group-IV was the smallest group with only 8 NAC proteins that included 6 CrNAC proteins together with 1 each of OsNAC and AtNAC. Nevertheless, the highest number of CrNAC proteins was found in Group-III which contained 12 proteins clustered with 3 OsNACs and 1 AtNAC.

### 3.2 Gene structure and conserved motif analysis

**Figure 2:**
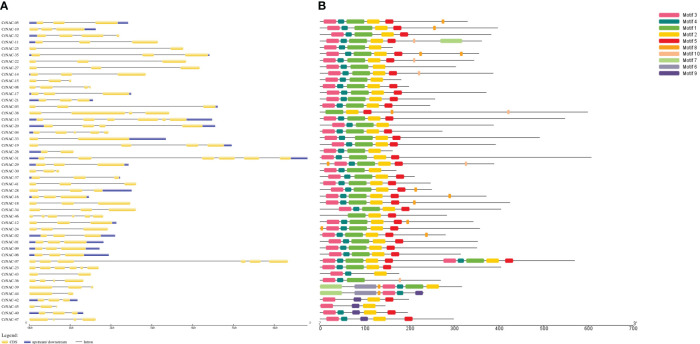
**(A)** The intron-exon structure of CrNAC proteins. Black lines indicate intron while yellow boxes represent exons. The 3´ and 5´ UTRs are shown by blue boxes. **(B)** Conserved motifs identified in CrNAC genes are represented by a different color.

The length of NAC genes in *C. roseus* varied greatly, ranging from 683 bp to 6813 bp. The number of exons in NAC genes ranged from 2 to 6. A majority of genes (29) had 3 exons while 5 genes contained only 2 exons. *CrNAC-07* and *CrNAC-31* were the largest genes each containing 6 exons (**Figure 2A**). A total of 10 different motifs were found in CrNACs (**Figure 2B**). Motifs 3 and 4 were found in all genes except *CrNAC-38* and *CrNAC-46*. The highest number of motifs were found in genes belonging to group-III.

### 3.3 Location on chromosome, duplication, and synteny analysis

**Figure 3:**
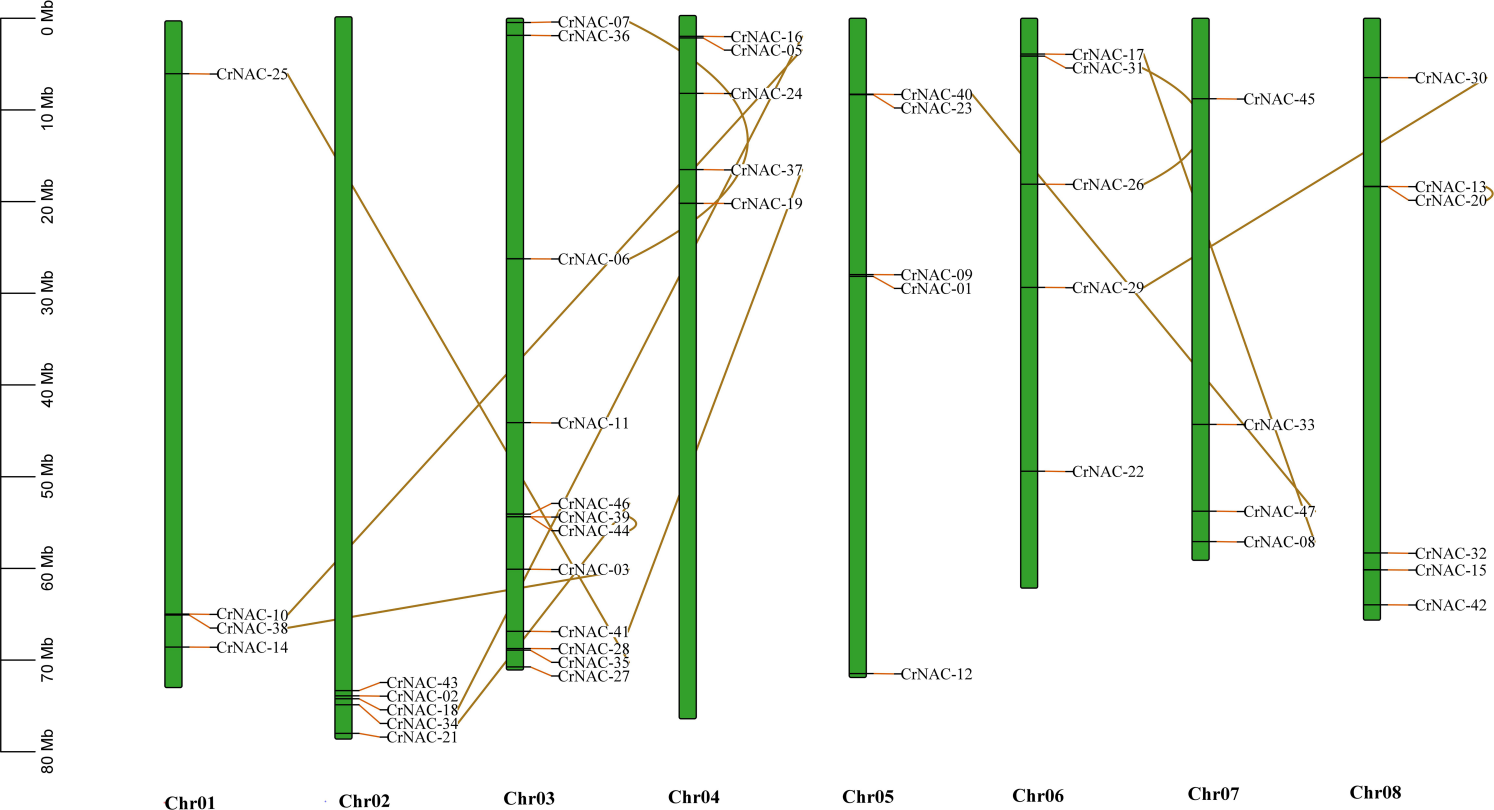
Chromosomal location of CrNAC genes across eight chromosomes of *C. roseus*. The chromosome number is indicated at the bottom. The scale in megabases (Mb) on the left side represents chromosomal distance while duplication of CrNAC genes is indicated by line.

**Figure 4:**
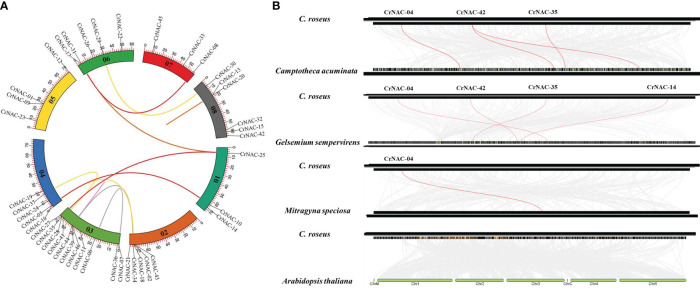
**(A)** Chromosomal locations of CrNAC genes and their synteny are illustrated by the circos diagram. Colored lines indicate duplication events. **(B)** Synteny analysis of NAC gene family between *C*. *roseus, C. acuminata, G. sempervirens, and A. thaliana.*.

Chromosomal location analysis showed that 47 NAC genes were unevenly distributed on all eight chromosomes in *C. roseus*. The highest number of *CrNAC* genes (12) were found on chromosome 3 followed by 6 on chromosome 8. Similarly, chromosomes 2, 4, and 6 contained 5 CrNAC genes each while chromosomes 1 and 7 contained 4 each (**Figure 3**). Gene duplication analysis predicted fourteen CrNAC gene pairs in the *C. roseus* genome making a total of 28 duplicated genes, four of which appeared tandemly duplicated (*CrNAC-06/CrNAC-07, CrNAC-13/CrNAC-20, CrNAC-26/CrNAC-31* and *CrNAC-39/CrNAC-44*) while the remaining ten gene pairs were segmentally duplicated (**Figure 4A**). **Table 2** shows ka/ks <1 for duplicated gene pairs which was maximum for *CrNAC-34/CrNAC-46* gene pair (0.39). **Figure 4B** shows different syntenic relationships of CrNACs with their counterparts in *C. acuminata* (4), *G. sempervirens* (4), *M. speciosa* (3) and *A. thaliana* (0).

**Table 2:** Duplicated gene pairs, non-synonymous substitution rate (Ka), synonymous substitution rate (Ks), Ka/Ks ratio, and mode of gene duplication.

Duplicated Genes	Ka	Ks	Ka/Ks	Mode of Duplication
*CrNAC-05/CrNAC-10*	0.257935451	1.222296944	0.211025195	Segmental
*CrNAC-25/CrNAC-35*	0.39398677	2.339058792	0.168438165	Segmental
*CrNAC-08/CrNAC-17*	0.260255837	1.834839679	0.141841186	Segmental
*CrNAC-13/CrNAC-20*	0.469176211	1.562081754	0.300353173	Tandem
*CrNAC-26/CrNAC-31*	0.263698129	2.881045263	0.091528631	Tandem
*CrNAC-29/CrNAC-30*	0.364507462	3.659662415	0.09960139	Segmental
*CrNAC-37/CrNAC-41*	0.324896467	1.774951051	0.183045311	Segmental
*CrNAC-16/CrNAC-18*	0.325800435	1.221718271	0.266673948	Segmental
*CrNAC-34/CrNAC-46*	0.476347968	1.205381601	0.39518437	Segmental
*CrNAC-06/CrNAC-07*	0.245271716	0	0	Tandem
*CrNAC-39/CrNAC-44*	0	0	0	Tandem

### 3.4 Cis-acting regulatory element analysis of CrNAC genes

**Figure 5:**
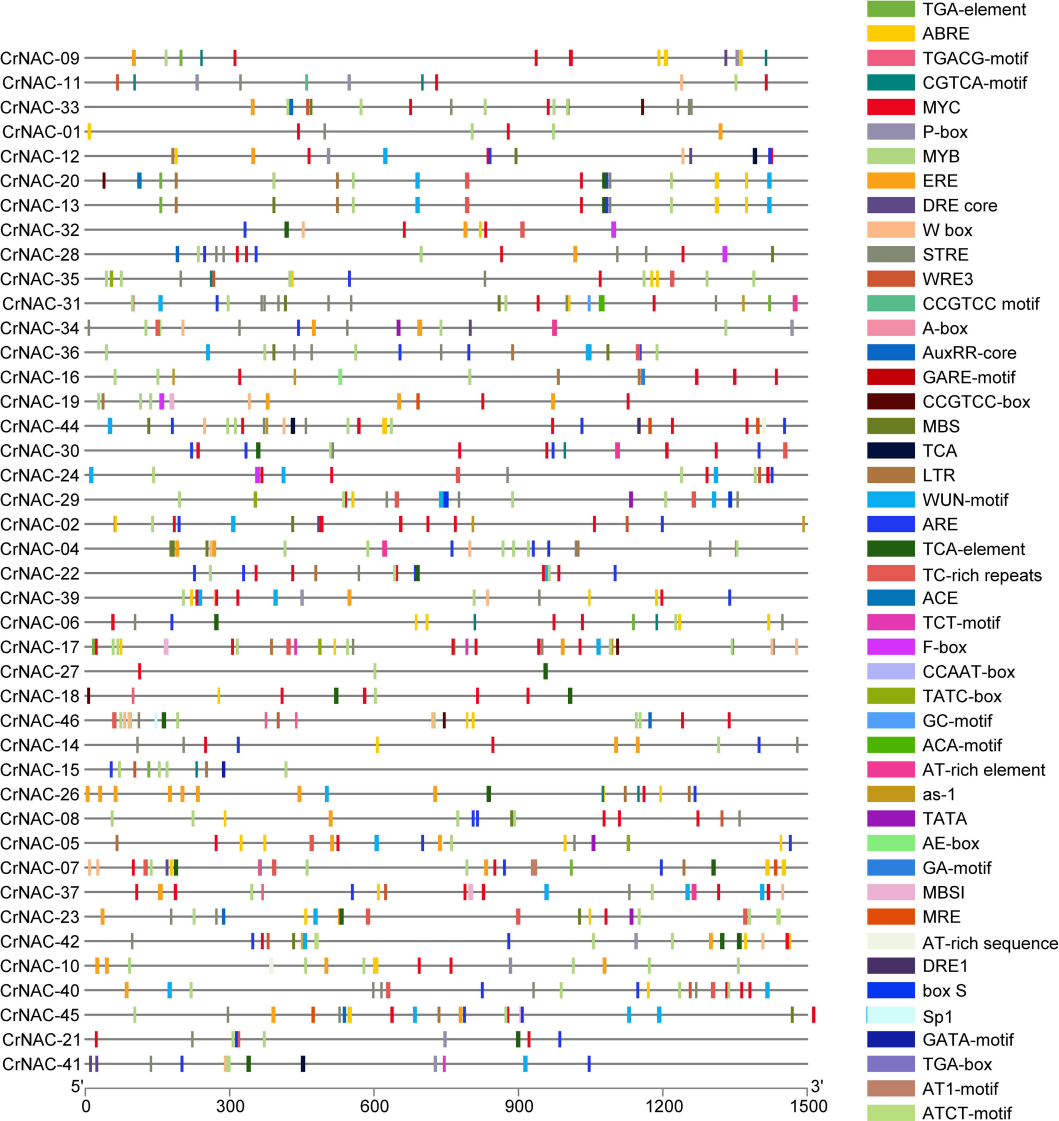
Distribution of cis-acting elements in the promoter region of CrNAC genes. Boxes represent specific cis-acting elements.

The promoter region of CrNAC genes showed many cis-acting elements, falling into six major categories that included core promoter elements, stress-responsive, light-responsive, binding sites, development-related and hormone-responsive elements. The distribution of stress and hormone-responsive elements;’’ of CrNAC genes are shown in **Figure 5**.

### 3.5 Gene expression and co-expression network analysis

**Figure 6:**
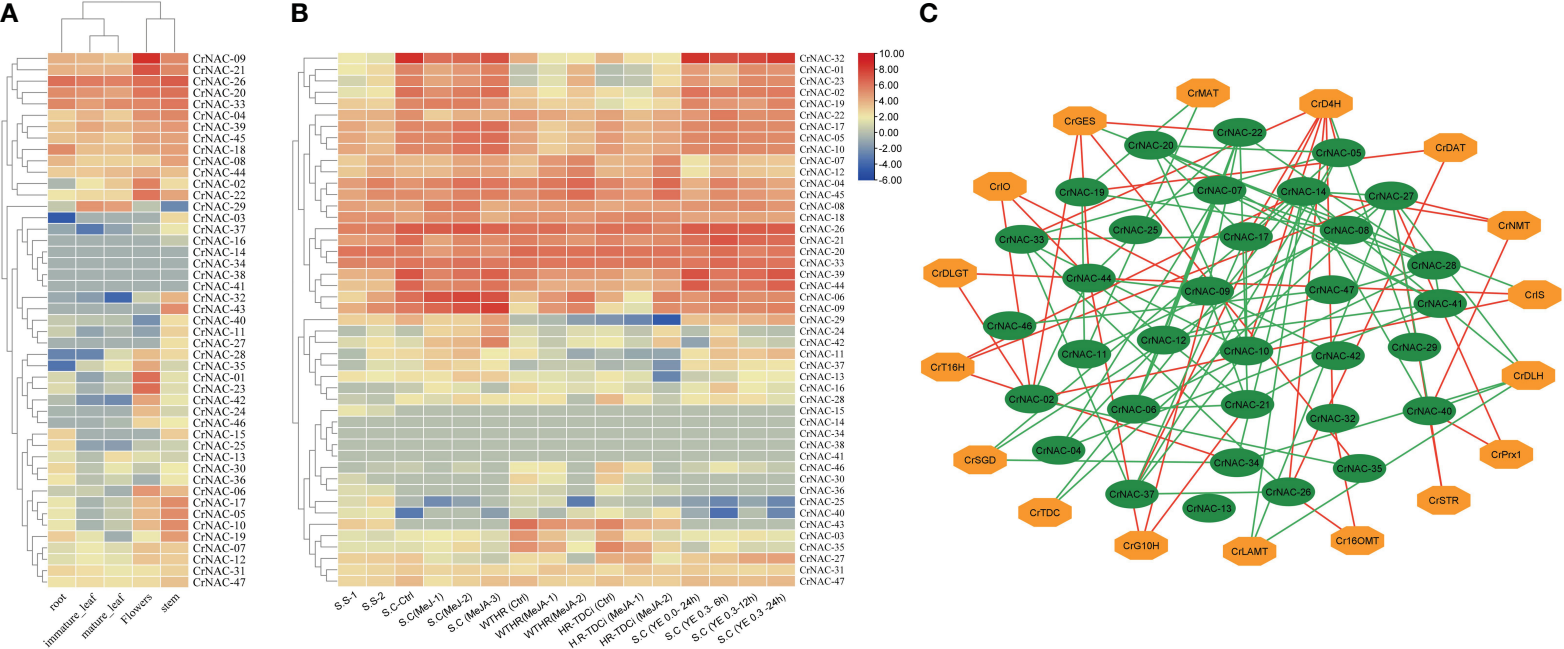
**(A)** Heat map showing the expression pattern of CrNAC genes in roots, immature leaf, mature leaf, flowers, and stem. **(B)** Heat map showing the expression pattern of CrNAC genes in various cultures and response to methyl jasmonate and yeast extract. **(C)** Co-expression network showing a correlation between *Cr*NACs and MIAs biosynthesis genes. Green lines represent a positive whereas red lines show a negative correlation.

The available RNA-Seq data showed that most *CrNAC* genes were expressed in all four tissues indicating their roles in regulating plant growth and development. However, few NAC genes showed tissue-specific expression patterns suggesting their role in tissue-specific physiological processes. For example, *CrNAC-01*, *CrNAC-23* and *CrNAC-35* were specifically expressed in flowers. (**Figure 6A**). Furthermore, the RNA-Seq available on Methyl Jasmonate (MeJ) treated *C. roseus* seedling, cell suspension, and hairy root culture were analyzed for transcript abundance of CrNAC genes. (**Figure 6B**). At the seedling stage, MeJ treatment positively induced the expression of 24 CrNACs while 13 were downregulated. In suspension culture, MeJ treatment (100 µM) for 12 h was found most effective in upregulating the expression of most CrNAC genes compared to 6 h and 72 h treatments. In hairy root cultures, 22 CrNAC genes were downregulated by MeJ treatment while 16 NAC genes were upregulated, and expression of 11 NAC genes remained unchanged in both control and MeJ-treated samples. The expression pattern of NAC genes in suspension cultures treated with yeast extract is shown in **Figure 6B**. Out of 47 genes, 10 NAC genes showed no expression in any treated sample while *CrNAC-24* and *CrNAC-42* expressed only in 0.3 mg/ml yeast extract after 6 h in suspension culture. **Figure 6C** shows a predicted co-expression network between putative CrNACs and rate-limiting genes of MIAs biosynthesis. Among 17 MIA biosynthesis genes, 5 showed a positive correlation with most of the CrNACs. On the other hand, a single CrNAC showed positive interaction with one set of CrNACs while negative interaction with others. For instance, *CrGES* showed a positive correlation with *CrNAC-20* whereas a negative correlation with *CrNAC-02* and *CrNAC-19*. These findings suggested a complex regulatory network working between *Cr*NACss and MIA biosynthesis genes, with certain *Cr*NACss showing a strong positive correlation with multiple biosynthesis genes, while others exhibited negative or no correlation.

### 3.6 Effect of elicitors on MIA production in *in-vitro* cultures

**Figure 7:**
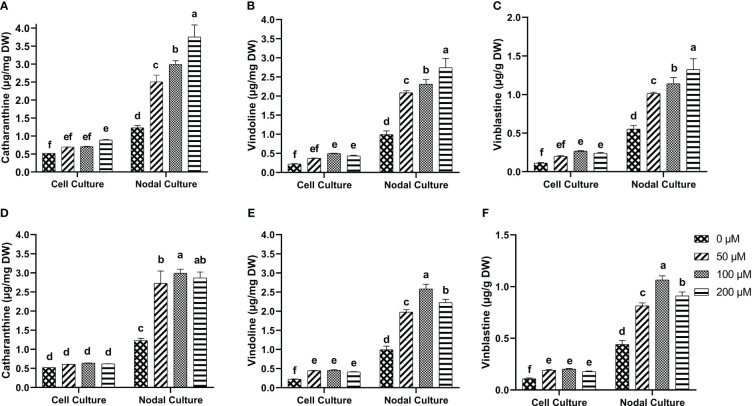
*In-vitro* biosynthesis of MIAs in response to 5-F-IAA elicitor **(A–C)** and PEO-IAA elicitor **(D–F)** in cell suspension and nodal cultures of *C. roseus*. Bars show mean ± SD. Different lower-case letters show statistically significant differences among means at p ≤ 0.05 by Tukey HSD.

Tukey test for the multiple comparisons of means showed that 200 µM of 5-F-IAA was the optimum concentration for significantly highest production of catharanthine in both nodal (3.74 µg/mg DW) as well as cell suspension culture (0.89 µg/mg DW) (**Figure 7A**). On the other hand, 100 µM of PEO-IAA produced maximum catharanthine (2.99 µg/mg DW) in nodal culture while none of the PEO-IAA concentrations in the cell suspension culture was effective enough to significantly increase catharanthine biosynthesis in cell suspension culture compared to control (**Figure 7D**).

5-F-IAA elicited significantly higher vindoline production at all concentrations compared to the control. The maximum vindoline production (2.74 µg/mg DW) was observed in the nodal culture at 200 µM of 5-F-IAA which was 2.7-fold higher than the control. Likewise, in cell suspension culture, 100 µM 5-F-IAA was effective in producing almost double the amount (0.49 µg/mg DW) of vindoline than control (0.22 µg/mg DW) (**Figure 7B**). The highest concentration (2.58 µg/mg DW) of vindoline was produced in response to 100 µM PEO-IAA in nodal culture while 200 µM significantly decreased vindoline biosynthesis. In cell suspension culture, increase in vindoline production was independent of an increase in elicitor concentration as is evident by the values observed at 50 µM (0.44 µg/mg DW), 100 µM (0.46 µg/mg DW), and 200 µM (0.41 µg/mg DW) (**Figure 7E**).

A concentration-dependent significantly linear increase in vinblastine production was observed in response to 5-F-IAA. The highest production of vinblastine (1.32 µg/mg DW) was observed in the nodal culture at 200 µM of 5-F-IAA. On the contrary, 100 µM 5-F-IAA elicited the highest biosynthesis (0.26 µg/mg DW) of vinblastine in cell suspension culture. (**Figure 7C**). Elicitation of vinblastine by 100 µM PEO-IAA was effective in producing 2.46-fold higher vinblastine in nodal culture than control. Vinblastine production significantly increased in PEO-IAA-treated cell suspension cultures compared to the control, however, the increase in vinblastine production was independent of PEO-IAA concentration (**Figure 7F**).

### 3.7 Effect of elicitors on the expression of genes and transcription factors involved in MIA biosynthesis

#### 3.7.1 Genes involved in biosynthesis of strictosidine

**Figure 8:**
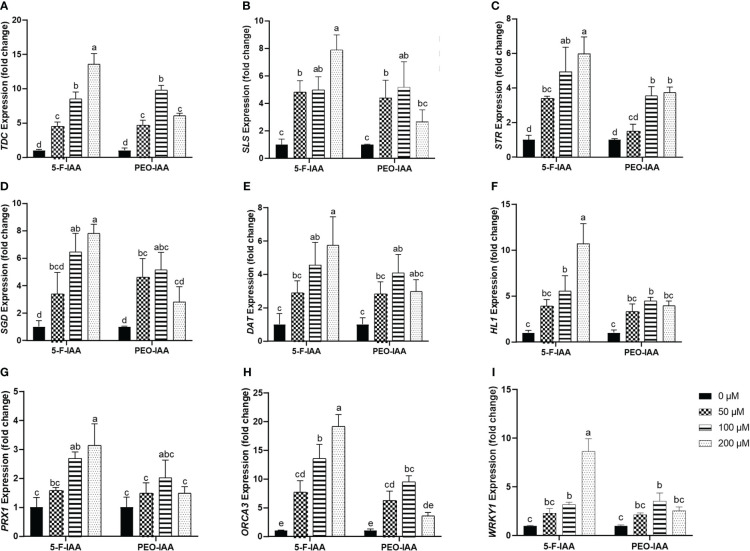
Relative expression of genes involved in the biosynthesis of MIAs upon elicitation with 5-F-IAA and PEO-IAA. Data for **(A)** *TDC*, **(B)** *SLS*, **(C)** *STR*, **(D)** *SGD*, **(E)** *DAT*, **(F)** *HL1*, **(G)** *PRX1*, **(H)** *ORCA3*, and **(I)** *WRKY1* are presented as fold change ± SD. Different lower-case letters show a statistically significant difference in mean values revealed by Tukey HSD at p ≤ 0.05.

Expression of the selected genes of *C. roseus* upon elicitation with different concentrations of 5-F-IAA and PEO-IAA in nodal culture is presented in **Figure 8**. The results showed that all the elicitor concentrations significantly upregulated the expression of *tryptophan decarboxylase* (*TDC*) compared to the control. The highest expression of *TDC* (13.5-fold) was observed in response to 200 µM of 5-F-IAA compared to its control. Both 5-F-IAA and PEO-IAA at 100 µM concentration were similarly effective in giving an 8.49-fold and 9.74-fold increase in *TDC* expression, respectively (**Figure 8A**). *Secologanin synthase* (*SLS)* expression in *C. roseus* nodal culture was significantly higher upon treatment with 200 µM of 5-F-IAA, exhibiting a 7.89-fold increase compared to the control. (**Figure 8B**). 200 µM 5-F-IAA was effective enough to increase 7.79-fold expression of *strictosidine β-D-glucosidase* (*SGD*). Among the rest of the treatments, the lowest value for *SGD* expression (2.8-fold) was recorded for 200 µM of PEO-IAA where the mean value was insignificant to the mean control value (**Figure 8D**). The relative expression of *strictosidine synthase* (*STR*) was 5.9-fold and 4.9-fold higher in leaves of *C. roseus* nodal culture treated with 200 µM and 100 µM of 5-F-IAA, respectively. However, 50 µM of PEO-IAA failed to upregulate significantly higher expression of *STR* (1.49-fold) compared to the control (**Figure 8C**).

#### 3.7.2 Genes involved in biosynthesis of vinblastine and its precursors

Of the three genes, *hydrolase-1* (*HL1*) showed the highest relative expression (10.7-fold) in response to the treatment with 200 µM of 5-F-IAA followed by 100 µM of 5-F-IAA (5.52-fold) and 100 µM of PEO-IAA (4.45-fold) (**Figure 8F**). A similar pattern of relative gene expression was recorded for *deacetylvindoline O-transferase* (*DAT*) in which 200 µM of 5-F-IAA enhanced the 5.74-fold *DAT* transcript level compared to its control (**Figure 8E**). Like *HL1* and *DAT*, the expression of *peroxidase-1* (*PRX1*) was recorded highest upon elicitation with 200 µM of 5-F-IAA (3.13-fold) followed by 100 µM of 5-F-IAA (2.68-fold). No concentration of PEO-IAA was able to elicit statistically significant expression of *PRX1* compared to the control. (**Figure 8G**).

#### 3.7.3 Genes coding for transcription factors involved in biosynthesis of MIAs

In the present study, a notable difference in the expression of *ORCA3* and *WRKY1* was observed, where the expression of *ORCA3* was significantly higher than *WRKY1* (**Figure 8H**). Application of 200 µM of 5-F-IAA was equally effective for the correspondingly highest expression of both *ORCA3* (19.15-fold) and *WRKY1* (8.6-fold) in the nodal culture of *C. roseus*. Except for 200 µM of PEO-IAA, application of all other elicitor concentrations of 5-F-IAA and PEO-IAA promoted significantly higher expression of *ORCA3* than the control. Expression of *WRKY1* upon elicitation with 100 µM of 5-F-IAA and 100 µM of PEO-IAA were not significantly different from one another but significantly higher than the control (**Figure 8I**).

#### 3.7.4 Expression of *CrNAC-7, CrNAC-24* and *CrNAC-25*

**Figure 9:**
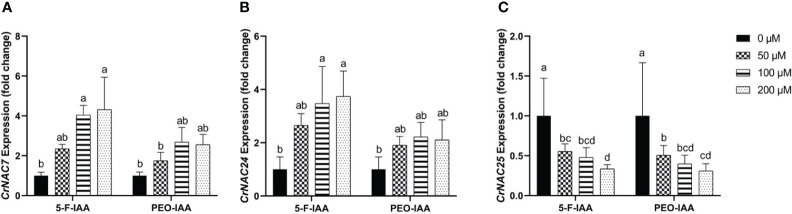
Relative expression of **(A)** *CrNAC-7*, **(B)** *CrNAC-24*, and **(C)** *CrNAC-25* is presented as fold change ± SD. Different lower-case letters show a statistically significant difference in mean values revealed by Tukey HSD at p ≤ 0.05.

Among CrNACs the highest expression was observed in response to the application of 200 µM of 5-F-IAA in *CrNAC-7* (4.3-fold) and *CrNAC-24* (3.7-fold) (**Figures 9A, B**). Interestingly, downregulation was observed for *CrNAC-25* at all treatment levels of both 5-F-IAA and PEO-IAA. The lowest expression observed for *CrNAC25* was -3.2-fold in response to 200 µM of 5-F-IAA (**Figure 9C**).

## 4 Discussion

Several studies have identified NAC families in many plant species, however, there is still no detailed information available on the NAC family in *C. roseus*. In the present study, 47 NAC TFs were identified in the *C. roseus* genome, which were classified into eight groups based on homology with AtNACs. Previously, NACs have been divided into 8 subgroups in banana (Cenci et al., 2014) and peanut (Li et al., 2021), 6 subgroups in soybean (Le et al., 2011), and 16 subgroups in cassava (Hu W. et al., 2015). This indicates that the NAC gene family in *C. roseus* is highly conserved and has similar diversity to that of banana and peanut. Among the 8 groups, group-I (AtNAC3/ATAF) and group-II (SNAC) members were classified as stress-responsive while group-VI (NAM) genes were found to regulate growth (Jiang et al., 2017). Furthermore, group-IV (NAC22) was also found to regulate morphogenesis, particularly root development. In *Arabidopsis*, the subgroup-AtNAC3 members like *ANAC055*, *ANAC019*, and *ANAC072* participate in the abiotic stress response (Bu et al., 2008).

Analysis of available RNA-Seq data showed that SNAC members *CrNAC-06*, *CrNAC-07*, and *CrNAC-09* were also upregulated in response to MeJ and yeast extract treatments, reflecting their involvement in stress responses. Validation of RNA-Seq by expression profiling of putatively identified *CrNAC*s showed that *CrNAC-07* and *CrNAC-24* were upregulated upon elicitation. The results for *CrNAC-07* were consistent with those observed from the analysis of available RNA-Seq data. Moreover, a previous study showed that *ANAC0032*, a member of the SNAC group responded to high sucrose, oxidative, and other abiotic stresses by regulating the synthesis of anthocyanin (Mahmood et al., 2016). The presence of stress-responsive cis-acting elements in the promotor of SNAC genes further confirmed their role in stress resistance. However, the upregulation of *CrNAC-24* was an interesting finding. Ectopic expression of an ATAF member *HaNAC1* in A. thaliana showed an improvement in growth as well as resistance to drought (Gong et al., 2020). Therefore, the upregulation of *CrNAC-24* may be attributed to the application of selected elicitors. Analysis of putative cis-acting elements in the promoter region of *CrNAC-24* revealed the presence of diverse cis-acting elements responsive to both biotic and abiotic stresses which may be the reason behind the enhanced transcript of *CrNAC-24*. The role of NACs in regulating plant secondary metabolites has just surfaced. Recently, 11 *liNAC* genes have been reported to positively correlate with 72 plant secondary metabolites in *Isatis indigotica* (Wang et al., 2023). Likewise, *CsNAC-7* has been shown to positively regulate the expression of *yhNMT1*, a caffeine synthase gene in tea plant (Fu et al., 2013). In apple, anthocyanin accumulation was noted upregulated upon binding of MdNAC25 to MYB genes (Sun et al., 2019).

It was observed that the expression of *CrNAC-25* was negatively regulated by both elicitors. *CrNAC-25* belongs to the NAC/NAM7 cluster of NAC TFs. The phylogenetically closest member in the subclade (*Os12g41680*) is reportedly characterized by its role in plant growth (Jiang et al., 2017). Interestingly, a decrease in biomass was also observed in response to elicitation (**Supplementary Figure S10**). Moreover, it has previously been reported that Osa-miR164a targets *Os12g41680* and downregulates its expression under *Magnaporthe oryzae* infection (Wang et al., 2018). Since both the elicitors are inhibitors of auxin signaling, therefore the decrease in growth-related *CrNAC-25* expression may be attributed to a trade-off between plant primary and secondary metabolisms (Huot et al., 2014).

In this study, the accumulation of three MIAs catharanthine, vindoline, and vinblastine was noted significantly higher in nodal culture than in cell suspension culture of *C. roseus*. In a similar study, (Linh et al., 2021) reported lower total alkaloid content in *C. roseus* calli than in the whole plant. In another study, metabolomic analysis of 20 plants showed that phenolic content in the callus cultures was either lower or completely lacking compared to intact plant parts such as roots and stems (Hagel et al., 2015). The difference in the production of MIAs in nodal and cell suspension cultures may be attributed to the complex biosynthetic pathway of MIAs, and tissue-specific cellular and subcellular localization of the intermediates involved in their biosynthesis (Miettinen et al., 2014). For example, initial steps in the biosynthesis of MIAs in the *C. roseus* plant occur in the internal phloem-associated parenchyma (IPAP) cells. The formation of catharanthine takes place in the cytosol of leaf epidermal cells, and vindoline is formed and stored in the vacuole of idioblast cells (Yamamoto et al., 2019). The presence of a central vacuole in differentiated nodal culture contributes to the enhanced biosynthesis of vindoline as compared to cell suspension culture where the vacuoles are smaller in size (Efferth, 2019).

Several reports established that auxins antagonize the production of specialized metabolites by reducing the activity of peroxidases (Bais et al., 2001; El-Sayed and Verpoorte, 2007; Aslam et al., 2010; Amoo and van Staden, 2013; Mekky et al., 2018). Contrariwise, a study conducted on *Arabidopsis* plants with a double mutant (*tir1 afb2*) of auxin signaling demonstrated an upregulation of genes involved in plant defense against stress (Iglesias et al., 2011). Moreover, salicylic acid has been shown to inhibit pathogen growth by repressing auxin signaling in *A. thaliana* (Wang et al., 2007). Considering all the available evidence, we hypothesized that an instant, transient, and dose-dependent inhibition of auxin signaling by small molecules can positively regulate the production of plant secondary metabolism. To test our hypothesis, we used two synthetic auxin signaling inhibitors namely 5-F-IAA and PEO-IAA to evaluate their potential as elicitors of MIAs in *C. roseus* cell suspension and nodal culture. A differential response of 5-F-IAA and PEO-IAA was observed toward the elicitation of MIAs. 5-F-IAA binds with ABP1 to inhibit auxin signaling in a transcription-independent manner whereas PEO-IAA competes with endogenous auxin and blocks the TIR1/AFB1 receptor in a transcription-dependent manner (Paponov et al., 2019). 5-F-IAA and ABP1 duplex-mediated inhibition of auxin signaling promotes the efflux of K^+^ ions and the influx of H^+^ ions making the cytoplasm acidic and the extracellular space alkaline. Such an environment instigates the events involved in the elicitor-induced biosynthesis of secondary metabolites (Ramirez-Estrada et al., 2016). However, intensive research focusing on the role of ion channels, plasma membrane phosphorylation, and acidification/alkalinization pattern of cytoplasm and extracellular space may provide further insights into the 5-F-IAA-induced accumulation of MIAs. Based on these results, it is recommended that higher concentrations than 200 µM of 5-F-IAA may be used to screen an optimal dose. Our results correspond to those of El-Sayed and Verpoorte (2007) wherein sub-culturing of the cells on an auxin-free medium enhanced the production of mRNA of the enzymes involved in alkaloid biosynthesis. Similarly, 2,4-D a synthetic analog of auxins strongly inhibited alkaloid biosynthesis during the growth phase of cell culture by reducing the activity of peroxidases vital to alkaloid biosynthesis. In a study, the substitution of 2,4-D with NAA in cell suspension cultures of *Tribulus terrestris* increased the concentration of steroidal glycosides (Tomilova et al., 2020). Likewise, in the current study, the concentration of MIAs produced in the callus induced by NAA was higher compared to that induced by 2,4-D (**Supplementary Figure S3**).

## 5 Conclusions

In the current study, we identified and characterized the NAC family in *C. roseus*. Furthermore, transcriptional and targeted metabolite profiling was carried out in elicited *in-vitro* cultures of *C. roseus*. An upregulation of stress-related *CrNAC*s whereas the downregulation of growth-related *CrNAC*s was observed under the same elicitation conditions. Based on these findings, it was concluded that NACs have their role in both growth and defense (plant secondary metabolite biosynthesis). The dual facet of NACs provides theoretical insights into the trade-off minimization between growth and defense. Owing to this property, NACs can be an ideal target for regulating growth and secondary metabolite production in medicinally important plants.

## Data availability statement

The datasets presented in this study can be found in online repositories. The names of the repository/repositories and accession number(s) can be found in the article/**Supplementary Material**.

## Author contributions

JA: Data curation, Formal analysis, Investigation, Methodology, Writing – original draft. YS: Data analysis, Software, Writing – review & editing. MKG and KEI: Funding acquisition, Visualization, Writing – review & editing. MH: Formal analysis, Software. SAK: Supervision, Technical support, Resources, Writing – review & editing. CS: Conceptualization, Methodology, Supervision, Resources, Validation, Visualization, Writing – review & editing. AMA: Technical support, Resources, Visualization, Funding acquisition, Writing – review & editing AH: Conceptualization, Methodology, Project administration, Resources, Supervision, Visualization, Writing – review & editing.
