# Supplementary material for: Genome-wide identification of NAC transcription factors and regulation of monoterpenoid indole alkaloid biosynthesis in Catharanthus roseus
Source: Front Plant Sci. 2023 Dec 20;14:1286584. doi: 10.3389/fpls.2023.1286584 (PMC10785006; doi:10.3389/fpls.2023.1286584)
Supplement: Supplementary file 1 [file DataSheet_1.docx]

Supplementary Materials

Genome-wide Identification of NAC Transcription Factors and Regulation of Monoterpenoid Indole Alkaloid Biosynthesis in *Catharanthus roseus*

Jawad Ahmed^1,2^, Yasar Sajjad^1^, Mansour K. Gatasheh^2^, Khalid Elfaki Ibrahim^3^, Muhammad Huzafa^4^, Sabaz Ali Khan^1^, Chen Situ^5^*, Arshad Mehmood Abbasi^6^*, Amjad Hassan^1^*

^1^Department of Biotechnology, COMSATS University Islamabad, Abbottabad Campus, 22060, Pakistan

^2^Department of Biochemistry, College of Science, King Saud University, P.O.Box 2455, Riyadh, 11451, Saudi Arabia

^3^Department of Zoology, College of Science, King Saud University, P.O. Box 2455, Riyadh 11451, Saudi Arabia

^4^Department of Plant Sciences, Quaid-e-Azam University Islamabad, Pakistan

^5^Institute for Global Food Security, School of Biological Sciences, Queens University Belfast, BT9 5DL, United Kingdom

^6^Department of Environmental Sciences, COMSATS University Islamabad, Abbottabad Campus, 22060, Pakistan

*** Correspondence:**

[c.situ@qub.ac.uk](mailto:c.situ@qub.ac.uk)

[amjadhassan@cuiatd.edu.pk](mailto:amjadhassan@cuiatd.edu.pk)
[amabbasi@cuiatd.edu.pk](mailto:amabbasi@cuiatd.edu.pk)

Jawad Ahmed [Jawadahmed850@yahoo.com](mailto:Jawadahmed850@yahoo.com)

Yasar Sajjad [yasarsajjad@cuiatd.edu.pk](mailto:yasarsajjad@cuiatd.edu.pk)

Mansour K. Gatasheh [mgatasheh@ksu.edu.sa](mailto:mgatasheh@ksu.edu.sa)

Khalid Elfaki Ibrahim [kibrahim@ksu.edu.sa](mailto:kibrahim@ksu.edu.sa)

Muhammad Huzafa [mhuzafa@bs.qau.edu.pk](mailto:mhuzafa@bs.qau.edu.pk)

Sabaz Ali Khan [sabaz@cuiatd.edu.pk](mailto:sabaz@cuiatd.edu.pk)

Chen Situ [c.situ@qub.ac.uk](mailto:c.situ@qub.ac.uk)

Arshad Mehmood Abbasi [amabbasi@cuiatd.edu.pk](mailto:amabbasi@cuiatd.edu.pk)

Amjad Hassan [amjadhassan@cuiatd.edu.pk](mailto:amjadhassan@cuiatd.edu.pk)

Figure S1. Growth curve of cells in suspension culture of *C. roseus*.

Figure S2. Effect of five different concentrations of kinetin (0.5, 1, 1.5, 2, and 2.5 mg/L) in combination with 2.0 mg/L of 2,4-D and NAA each on callus induction of *C. roseus* leaf explant. Data is presented as mean ± SD. Different letters show statistically significant results by Tukey HSD at p ≤ 0.05.

Figure S3. A comparison of best callus-inducing combinations of phytohormones on the biosynthesis of MIAs in calli of *C. roseus*. Comb-1; 2.0 mg/L 2,4-D + 1.5 mg/L kinetin, Comb-2; 2.0 mg/L 2,4-D + 2.0 mg/L kinetin, Comb-3; 2.0 mg/L NAA + 1.5 mg/L kinetin, Comb-4; 2.0 mg/L NAA + 2.0 mg/L kinetin. Data is presented as mean ± SD. Different letters show statistically significant results by Tukey HSD at p ≤ 0.05.

Figure S4. Separation of target MIAs by T3 C18 column (Acquity Waters) at 30 °C.

Figure S5. Separation of MIAs by BEH C18 column (Acquity Waters) at 30 °C.

Figure S6. Separation of MIAs by CSH C18 column (Acquity Waters) at 30 °C.


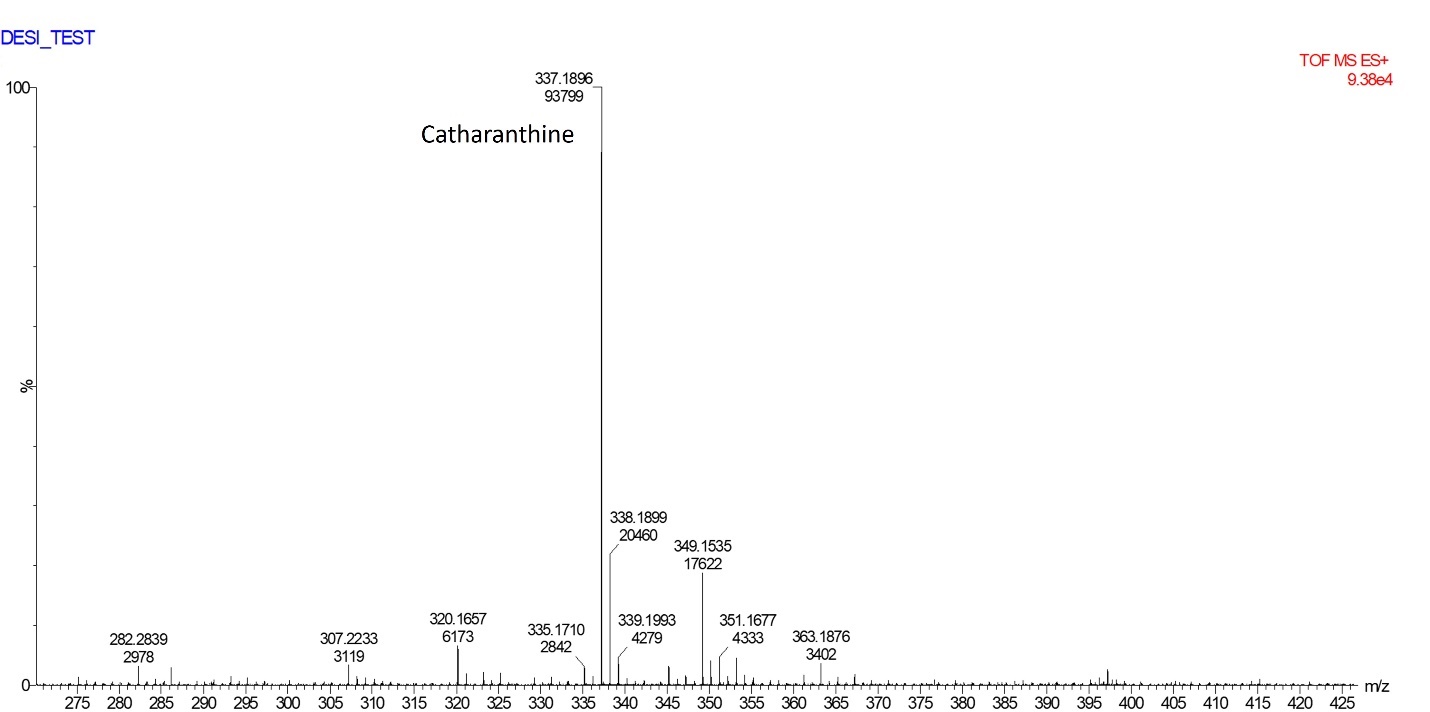


Figure S7. DESI spectra showing peak for catharanthine in methanolic extract of *C. roseus* leaves from nodal culture.


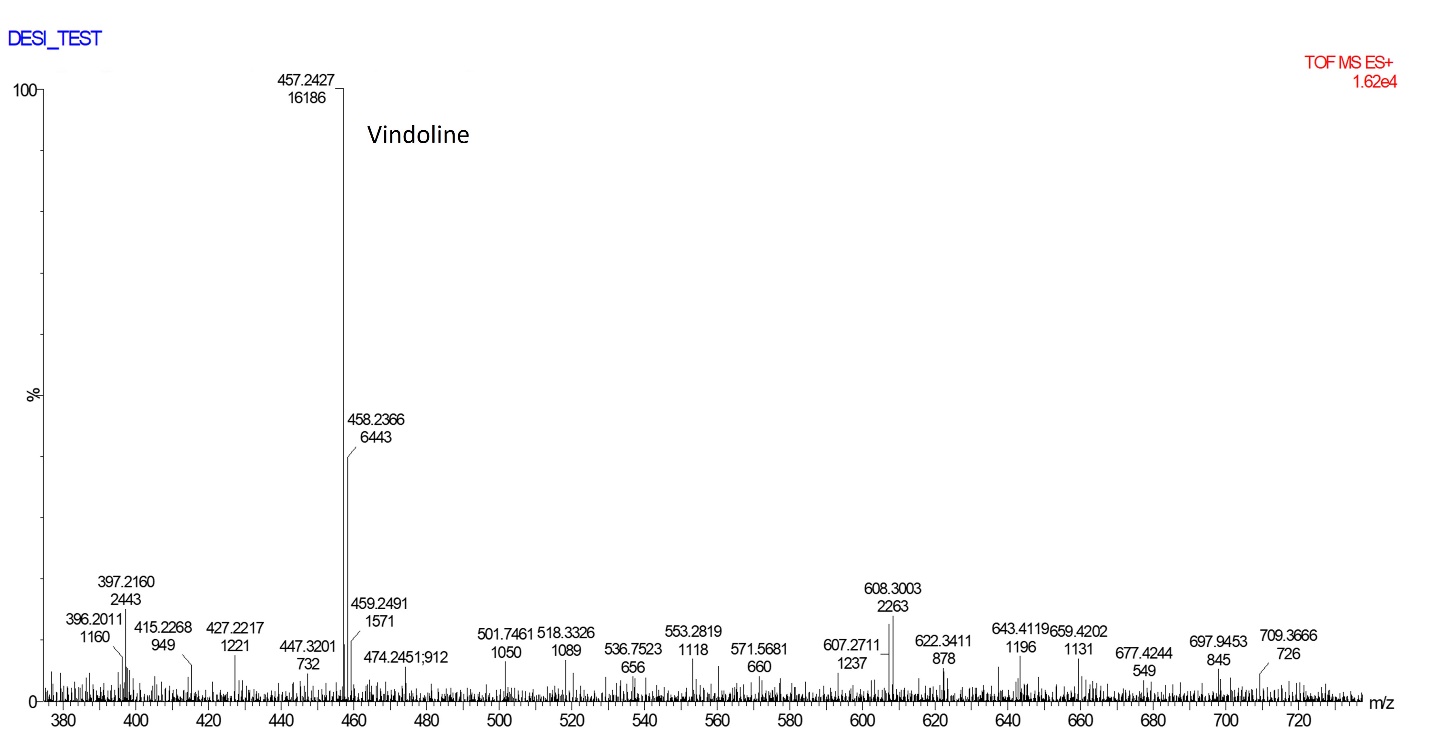


Figure S8. DESI spectra showing peak for vindoline in methanolic extract of *C. roseus* leaves from nodal culture.


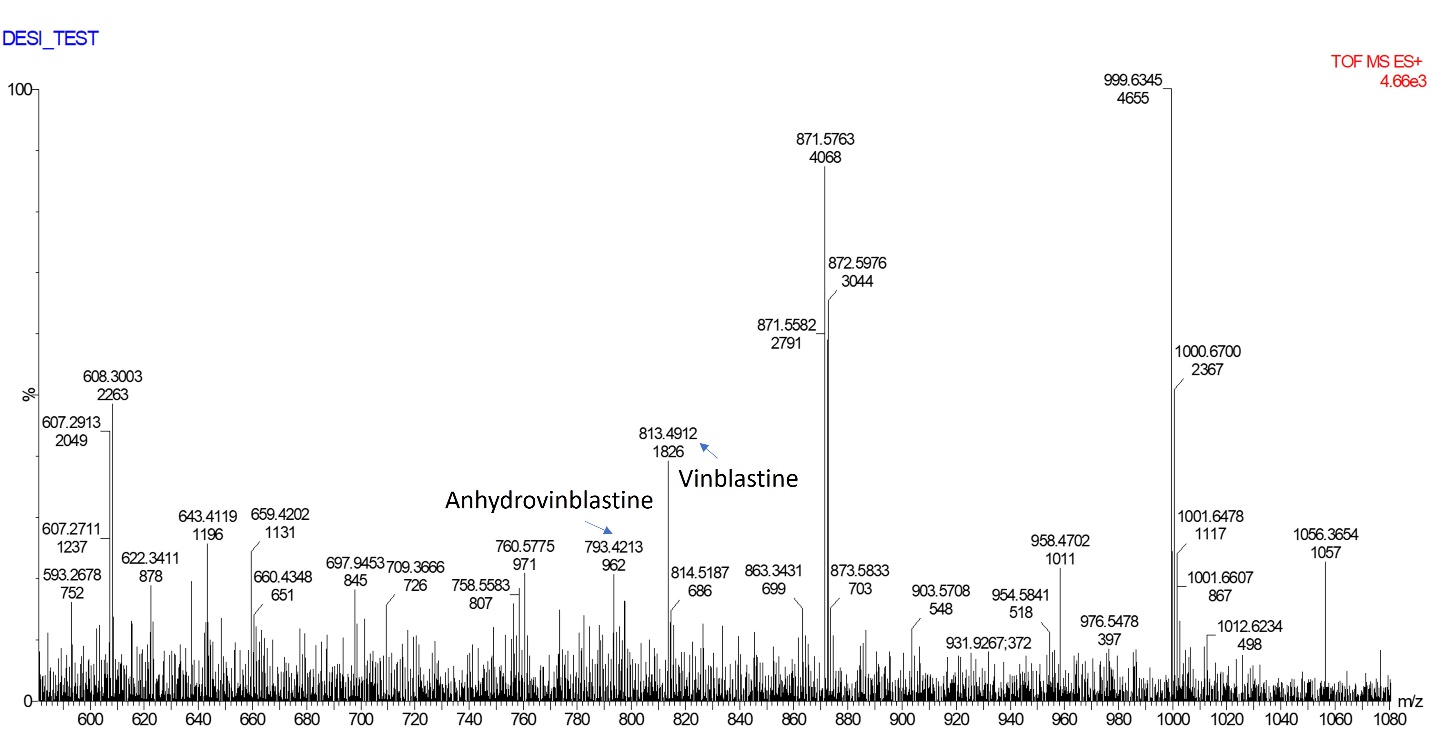


Figure S9. DESI spectra showing peaks of anhydrovinblastine and vinblastine in methanolic extract of *C. roseus* leaves from nodal culture.


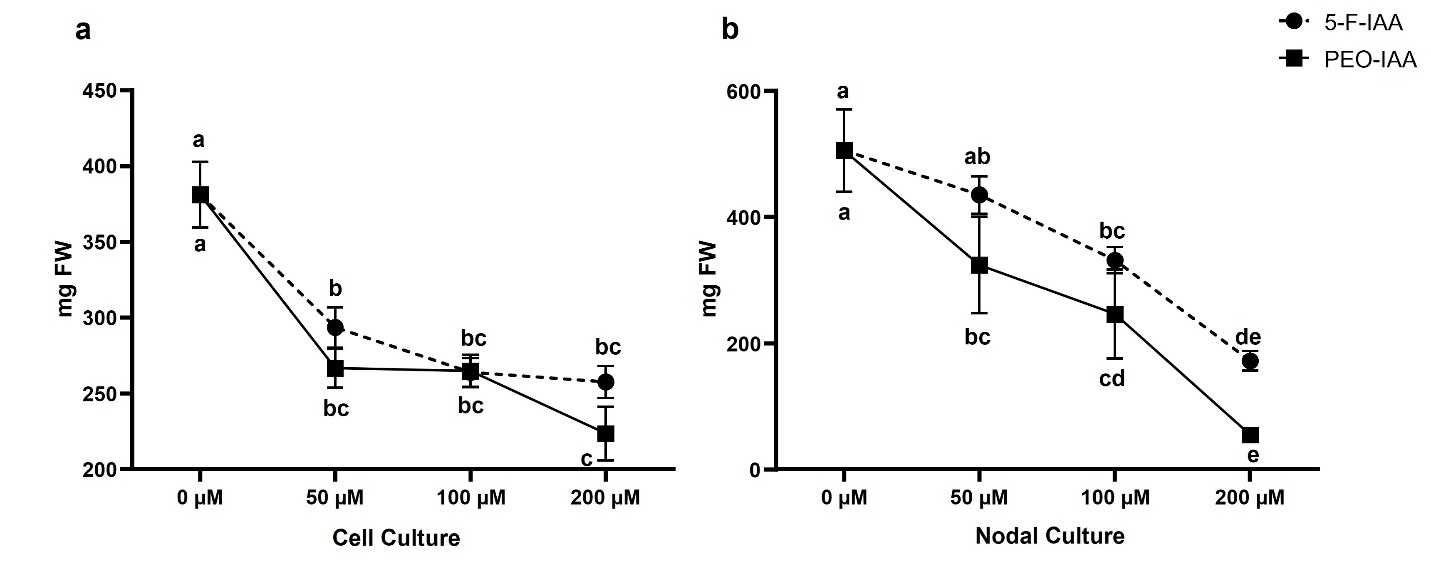


Figure S10. Effect of elicitation by 5-F-IAA and PEO-IAA on fresh weight in (a) cell suspension culture; (b) nodal culture of *C. roseus*. Data are presented as mean ± SD. Different lower-case letters show significant differences among means at p ≤ 0.05 by the Tukey HSD test.


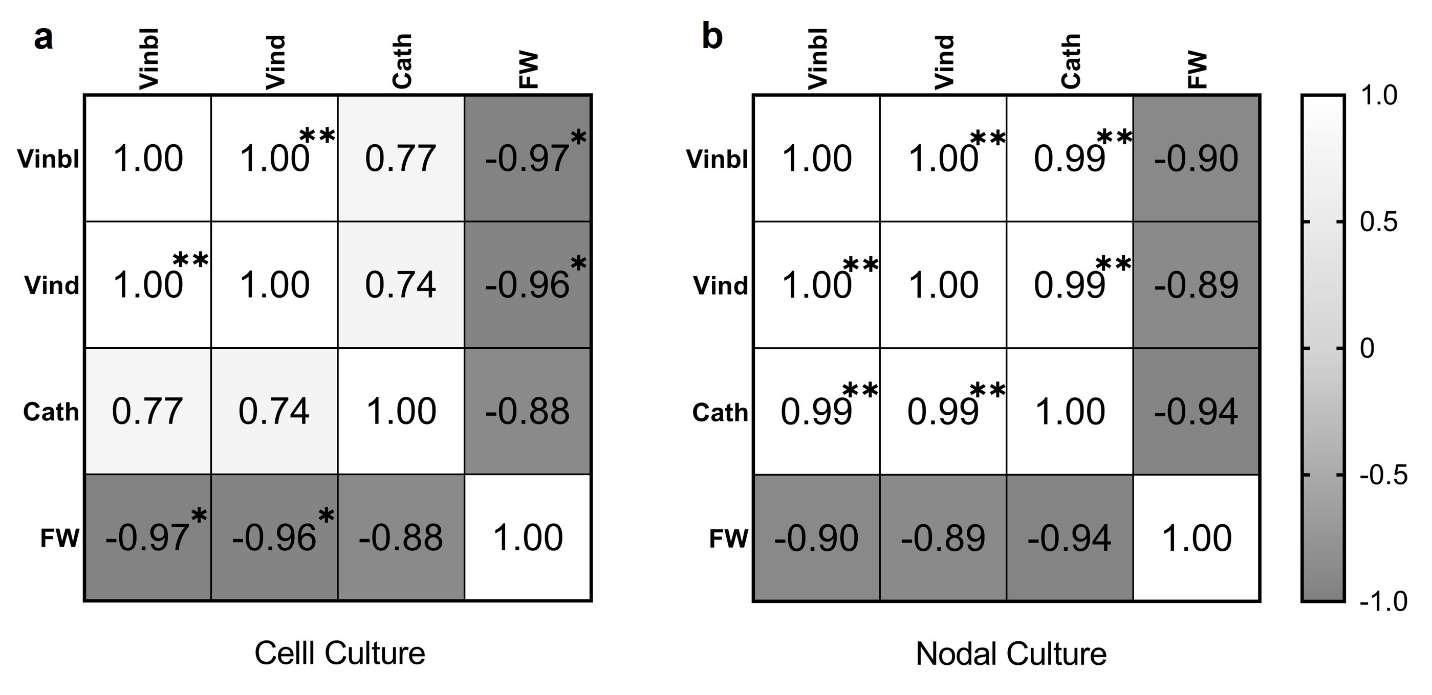


Figure S11. Correlation between the production of MIAs and fresh weight in 5-F-IAA treated samples in cell suspension culture (A), in nodal culture (B). *; significant (p ≤ 0.05), ** highly significant (p ≤ 0.01), Cath; Catharanthine, Vind; Vindoline, Vinbl; Vinblastine and FW; Fresh Weight.


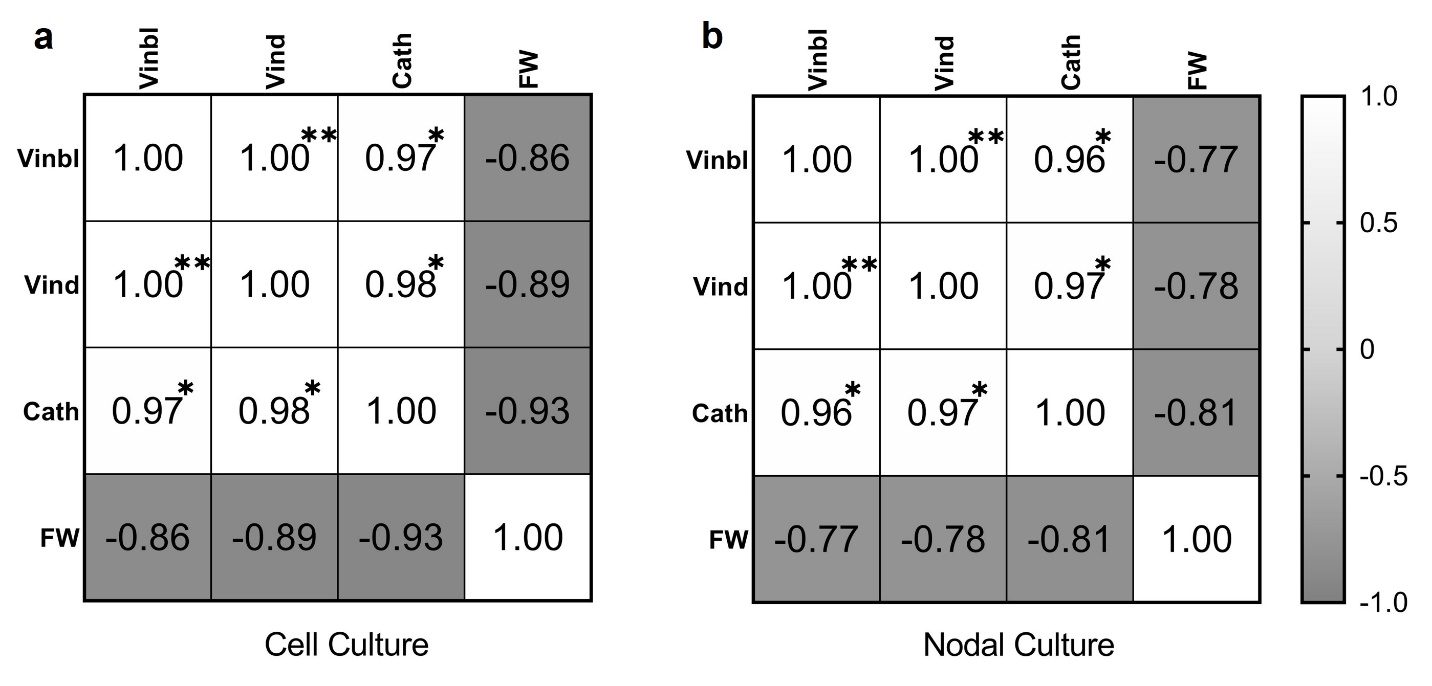


Figure S12. Correlation between the production of MIAs and fresh weight in PEO-IAA treated samples in cell suspension culture (A), in nodal culture (B). *; significant (p ≤ 0.05), **; highly significant (p ≤ 0.01), Cath; Catharanthine, Vind; Vindoline, Vinbl; Vinblastine and FW; Fresh Weight.

Table S1: Elution Profile for Separation of MIAs

| **Time** | **Solvent A (Formic Acid)** | **Solvent B (Acetonitrile)** |
| --- | --- | --- |
| 0 min | 75% | 25% |
| 3.5 min | 67.5% | 32.5% |
| 4.5 min | 5% | 95% |
| 5 min | 5% | 95% |
| 5.5 min | 75% | 25% |
| 7 min | 75% | 25% |

Table S2. The sequence of primers of selected genes

| **Gene (Accession Number)** | **Primer** | | **Sequence (5´-3´)** | **Product size** |
| --- | --- | --- | --- | --- |
| *TDC* (MG748691) | Forward | CTTACCTCCCCGAACCACTT | | 200 |
|  | Reverse | ATTTCTAATTCGGTGGCGGC | |  |
| *SLS* (KF415117) | Forward | ACCCACTCCAGCAAATACCA | | 166 |
|  | Reverse | CAAAAGCCGGCAACATTGAC | |  |
| *STR* (X53602) | Forward | TCCTATGCTCCGAATGCCTT | | 173 |
|  | Reverse | TTCTCTGGATCGGTGCTGTT | |  |
| *SGD* (AF112888) | Forward | AGAGCTCTTGTAGGAAGCCG | | 206 |
|  | Reverse | CGCACTTCCTTCCCATCAAC | |  |
| *DAT* (AF053307) | Forward | TTTACGAAAATCCCGACGGC | | 249 |
|  | Reverse | TGCCAACAGTTACCCGTTTG | |  |
| *HL1* (MF770512) | Forward | AAGAGAAACTGAAGGGCGGA | | 227 |
|  | Reverse | AACCAACAACCTCGAGCAAC | |  |
| *PRX1* (AM236087) | Forward | TCAGCAGGCTTTCAGGATCA | | 244 |
|  | Reverse | TGCGAGTGAAGTTAGGAGGG | |  |
| *ORCA3* (AJ251249) | Forward | CGGAAAGCTGTCAGGAGGAT | | 193 |
|  | Reverse | GCATCCTCAGGTGTCTCGTA | |  |
| *WRKY1* (HQ646368) | Forward | TGCCGAACTCTCCGTACAAA | | 162 |
|  | Reverse | TACTTGACCCCTGCCGATTT | |  |
| *CrNAC-07* | Forward | TAAGCTTAATCGCCACGCAC | | 242 |
|  | Reverse | GGCGAGAGAAAGAGAGAGCT | |  |
| *CrNAC-24* | Forward | ACACAAACAATGCTGCCTCA | | 194 |
|  | Reverse | ACCCTTACCTTGTCCGTGTT | |  |
| *CrNAC-25* | Forward | GTGACCGAAAGTATGCGACC | | 186 |
|  | Reverse | CATGCATGATCCAACCGGTT | |  |
| *RSP9* (AJ749993) | Forward | TCAATCCACCATGCCAGAGT | | 162 |
|  | Reverse | TCTCTTCACTCTGCCAGGAC | |  |

Table S3. Components of the reaction mixture for qRT-PCR

| **Component** | **Volume** |
| --- | --- |
| iTaq Universal SYBR Green super mix | 13 µL |
| cDNA | 3 µL |
| Forward Primer | 1 µL |
| Reverse Primer | 1 µL |
| Nuclease free water | to make final volume of 25 µL |
