# Supplementary material for: Genome-wide identification of NAC transcription factors and regulation of monoterpenoid indole alkaloid biosynthesis in Catharanthus roseus
Source: Front Plant Sci. 2023 Dec 20;14:1286584. doi: 10.3389/fpls.2023.1286584 (PMC10785006; doi:10.3389/fpls.2023.1286584)
Supplement: Supplementary file 2 [file DataSheet_2.docx]

>CrNAC-37

ATGGATATGCAGCAGCAGGAAGATCAAAAGCAAAAAGAAATAAAATTAGTAGAATTCCAACCTGGGTTTCGATTTTATCCAACTGAAGAAGAGTTGGTTTCTTTTTATCTACGGAAGAAGCTGGAAGAAAATATCAGAGAGCCCAACCTCCTCGATCGTGTTATTCCAGTTATCCACATTTACGAATTCGAGCCATGGCTGCTTCCAAAGCTATCAGGAGAATTGTGCAGTGGAGATAGTGAGCAGTGGTTTTTCTTTGTACCAAGACAAGAAAGAGAAGCACGCGGCGGACGTCCTAACCGGACCACAGCCTCCGGTTACTGGAAGGCAACAGGTTCCCCCAACTACGTTTACTCTTCTAACAATAGAGTCATTGGTGTGAAGAAAAGCATGGTTTTTTATAGAGGGAAAGCTCCTACCGGAAAAAAGACCACTTGGAAAATGAATGAGTATAGAGCCATTGAGGAACAAATTTTGCCTTCCTCATCATCTGCTCAACCTATTCCCAAGTTAAGGCATGAATTATCATTATGTAGAGTTTACATAATATCAGGGAGTTGTCGAGCATTCGATCGAAGGCCATTATGGACAGAGACAAGGAGGCTGGGGGAGATAGCTCAAGACTTCAGCAGCTAA

>CrNAC-41

ATGGGAGATGATGAAATATATCTTCAACCAGGATTTAGGTTTTATCCAACAGAAGAAGAATTGGTTTCTTTTTATCTCAAAAACAAGCTTGAAGGATTAAGAGTTGAAGAAATTAATATGGTTATTCCAGTTCTTGATATTTATCACTACAATCCATGGGAGCTTCCGAAATATGCAGGAAAATATAGTAGGAAAGATCCAGAAGAATGGTTTTATTTCATACCAATGCAAGAGAAGGAATCACGTGGAGGAAGACCAAATAGGCAAACAAATGAAGGGTATTGGAAAGCCACTGGTTCTCCTGGTTATGTTTATTCTTTAAAAAATAACAAAATTATTGGAGGCAAAAGAACTATGGTTTTTTACAATGGAAGAGCTCCTTATGGCAAAAAGACACCATGGAAGATGAATGAGTATAAATCTATTGATTATACAGCTCCTCAACTACCCCCAAAGTTGAGGCAAGAAATGAGCATATGCAGAGTTTACGTAAAATCAAAATCTGTAAGAGCATTTGACAGGCGACCACCACCGCCACCATCATCCATGCCAATTCATGATCCTCCATTAATACTTCCATCAGCAGCAGCAGCAGCAGCAGCTGGCTCTCAACTTAGTTCATCCCCCGGAGATGACAGCGCTAATAATGTGGAAAATAATTACGGAATGATCAACTGCTGGAGTTTCGCAGATGATGATGATGATAATACCCAACCTATGTGGGATTGGAAATTACTCAACTGA

>CrNAC-19

ATGGAGCAAGAGGAGAAGAAGGAGGTTTTCGTTTTGTCAAGGAAGAATATGGAAATTTCAATTGCAGAAGCATCATCAATGTTTCCTGGGTTCCGTTTTTCGCCCACCGACGAAGAGTTAATTTGCTATTATCTGAAGAAGAAGCTTGAAGGGTCAGATAAATGTGTTGAAGTTATTCCAGAAATTGATATTTGTAGGCATGAGCCTTGGGATTTACCAGCCAAATCAATCATTCAATCAGATAATGAGTGGTTTTTCTTTTCTCCTCGCGGGAGGAAATACCCCAATGGCTCCCAGAGTAAGAGGGCAACTGCCTGTGGCTACTGGAAAGCCACTGGGAAAGAACGAAATGTGAAGTCAGGTTCAGCTGTTATTGGCACAAAGAGGACTTTGGTTTTCCATACTGGTCGGGCACCAAAGGGACAGCGGACAGAGTGGATAATGCATGAATATTGCATGAGCGGAAAAGCACAGGATCTCATGGTAGTTTGCCGTCTTCGGAAGAACATTGAGTTCAATTTAGATGAGAATCCACGTAAGGGATCAGCAGGACAAAGACATTCATCCACTCTAGCCAACGATGTTGCAGCTTTATCTGTTGTTGAGCAGAATGGGGGAAAAGCTGGCACCCTGGTAGCTGACTCTTGCTCAAAGGAAGGTAGCAGCAGCTATAATTCCCATTCAGTTGAGCAGAATGACTCTTGGTTTGATTCCGTAGATAAAACAACTAATGAAATCTCTCCACATGGCTCTTCCAGCTGCCGGCAATTCCAGGGCAGTGACAGAAATGAAGAGGATTGGTTTGCAGATATAATGAACGACGATATCATCAAGCTCGATGAATCTTCGCTGACTGCGAATCCTCATCTACTGCCAACAGCTCCTGGAGAACCTCAACCCAAGATCACTTCTACAGAACCAAGCCGAGTTATAGCACCTGCTGCCCTTCCTTTCCAGGGCACAGCAAATCGACGACTCAGACTCAGGAGGCCGAAAATCGAACTGAAGAATGCAACACCGTTAACAGCATATGAATCAGGCGTTGTCACCTTAACCAGAGAGCTTGTCCATTCCCAAGATTCACAAAAGTCGCAGAAAAACTTGGGCATGGCCATTAAGCATCGGGTAGTTTTAGTGCTATTTGTGATCATACTTGTGTTACTTTTGTACATGAAGTTGTGA

>CrNAC-04

ATGGGAGGAGCATCTCTGCCTCCGGGATTTCGTTTTCATCCAACTGATGAAGAACTTGTTGGATACTATCTTAAAAGAAAAACTGATGGACTTGAAATTGAGCTTGAAGTAATCCCAGTAATTGACTTGTACAAATTTGATCCATGGGAACTCCCAGAAAAGTCATTCTTACCAAAGCGAGACAAGGAATGGTTCTTTTTCTGTCCTCGTGATAAAAAGTACCCTAATGGTTCCCGGACTAATAGAGCAACTAGATCCGGATACTGGAAAGCCACAGGGAAGGACCGGAAAGTAGTCTGCCAATCTGCAGTGATTGGTTTTCGCAAGACCCTTGTGTTCTACCGTGGAAGAGCTCCACTTGGGGATAGAACAGATTGGGTAATGCATGAATATCGTCTTTGTGATGATGTCTCTCAAGGCATCCCAGTTTTTCAGGGACCTTTTGCCTTGTGCCGTGTCATCAAGAAGAATGAGCAGAAGATGAAGGATGATTACGCCCAACCACGCGGTTTACATCCAGAATATGGATTTCAAAACTCAATGTGGCAGTCTTCATATGATCAATTCGAAATCTCACCAACTTCATCAAACTCAAATCTTCAAGAAGAAGGGGAACCTTCTGATGATTTTAGTCGATATGGATGCATGTCGCCCTATTCGGTTTATGGAAGCTATATGGGGTACTATGGAAATGACATGTTATATGAAGGATTAGAGACTTGGAACCAAGCTCCTCCATTATGTAGACAGAGTAGTGAAGATGGAACTCTTGGAGAAATTAATGGTCTTTGGTCATTGGAAGATTTAGCTAGAGTGATGTAA

>CrNAC-33

ATGTCTTCTTGTTTTGACGATGGTAAATTTTTCCCGCCGGGATTTAGATTCCACCCGACGGATGAAGAGCTTGTTCTGTACTATTTAAAGAGGAAGATCTGCCGGCGGCGACACAGGCTGGACGTTATCGGCGAGACTGACGTCTACAAGTGGGACCCAGAAGAGTTGCCTGGTACAACTATTGATACTGAATTTCGTTTTGATTTAATTTTTGATAAAATATCTAAGTTGAAAACTGGAGACAGGCAGTGGTTCTTCTTTAGTCCCAGAGACAGGAAGTATCCAAATGGTGCAAGATCGAATAGAGCAACCAGGCATGGTTACTGGAAAGCAACTGGGAAGGACCGTATCATTACTTGCAATTCTCGTCCTGTTGGGGTAAAGAAAACTCTGGTTTTCTACAGAGGGCGTGCACCTACAGGGGAGCGTACTGACTGGGTAATGCATGAGTACACCATGGATGAAGATGAGCTGAAAAGATGTCCTGCAAAGGAATATTATGCTCTGTACAAAGTCTTTAAAAAGAGTGGACCTGGTCCAAAGAACGGTGAGCAATACGGTGCACCTTTTAGGGAAGAGGATTGGGCAGATGAAGTTGAGCCTAATGGGCTTGTTGAGCGGTGCAAGTCTGTAGAGCAGGTTGCAGAATGTGTGCCTGTTGATGATAATAGAATTAATTCTCAGCTGCAGTCCCAATTAGAAGATCTTGAGGAGTTCATGAACAGGATTGCAGATGATGCAATACTTGAACCACCACCAGTTGATGACTTTGCTTATGCTTTAGGGGAGCTTGTGCGTGAGGAGGAGGCACAAAGCAATATAGTGGATCAGTCCTCAAAGGAATACAATTTACCTGAGCAAAGCATTGTGGTTCTGCCACACTGTCAACAATATGATGTGCAAGCTAGTTATGACTTGACTCAGTCTGCTACTTCTCAATTTCAGTTGCATGAGACTTCTGAGGTCACGTCTGCTCCAAAGGGTCACATTCCAGAGCCTTACACAGTTGAAGAGGATTTCCTTGAAGATTTTCTGGAGTTGGATGATCTTATGGGTCCAGATCCTTCTGTTCAAAGGTCTCATAAACAAGCAGAAGCCCATGGGAACCAAGATTCCATTGATGAACTTGATGGTCTGTTCGAATTTGAATTGTACAAGGATGCATCATTGGTTCTTTCTGAAGTAGGACCAGGGGGAGAAGGGCAGCTTTGTCAAACATACGTGAATAATATGGTGAGCAAGGCTGCAGATCCGGTTTCAAATTTGTATTCTAATAATTTTGAGACAGGGACCATAAACTATCAGCAGACCAGCCATTCTCCAACTGAAAATGAGGTGAATTTTCAGCAGTGGGAGAGGTGCAGTGTCTTTACTCCTGCAGAGGCACAGCAGGGCACTATAACTCCTGCAGTTCCAGGTACTGAAACGACTGATGACTTTGTTGGTGCCCTTTATGATTGGGTTTCACAATATTAA

>CrNAC-26

ATGGCTGTGCTTCCGGTTAAATCGCTGCCTGTGGGCTATAGGTTCCGGCCGACGGACGAGGAGCTAATCAATCACTACCTGAGGCTGAAAATCACTGGATTCGACAAAGAAGTTAACATTATTCGCGAAGTTGATATCTGCAAATTGGAGCCTTGGGACTTGCCTGATTTGTCGCTGGTAGAGTCATATGATGATGAGTGGTTCTTCTTCTGTCCCAAGGATCGGAAGTACCAGAATGGCCAGCGGCTGAACCGAGCTACTCTGAAGGGTTACTGGAAAGCCACTGGTAAAGATAGGAATATTGTTTCGAGGAAAGGAGTGAAGATTGGGATGAAGAAGACTCTGGTCTTCTATACTGGGCGTGCTCCAGATGGAAAGAGGACTAATTGGGTGATCCATGAATACCGTGCAACTCAGAAGGAGCTTGACGGCACCCACCCTGGACAGGTTCTCTCTCTCTCTCTTTTCCTCCCCCTCCCCCTCATTTGA

>CrNAC-31

ATGGAATCCTTACCGCTGGGCTTTAGATTTAGGCCGACCGACGTCGAGCTTATCGATCATTACCTCAGGCTTAAGATCAACGGCCGTCATTCCGAGGTTCAGGTCATACCCGAAGTCGATGTCTGCAAGTGGGAGCCATGGGATTTGCCTCGGACTGATACACTGCTGTTGTTAACAGGGCTTTCGGTTATAAAAACAGATGATCCAGAATGGTTCTTCTTTTGTCCACGTGATAGGAAGTATCCAAATGGACATCGTTCTAACAGGGCTACCGATGCTGGTTACTGGAAGGCGACTGGCAAGGATCGCACTATCAAGTCTCGCAAATCTTCTCCATCTGGTCAGTCTAACCCCCAGTTAATTGGCATGAAGAAGACTCTTGTTTTCTATAGGGGTCGTGCACCTAAGGGTGAGCGCACAAATTGGATCATGCATGAATATCGCGCTACTGAAACAGATCTTGATGGAACAGCCCCTGACCAGGCTGCATATGTTCTTTGTCGCTTATTTCATAAGCCTGATGAAAAGACTGATAATTCTAAAGATGACGAAGCTGAACACACTGGTTCTTCTCCTACCACAGTAAGATCCTCACCTGATGACACATCATCAGATCTGTTTCAAGAACAACCTATCCTGGATATGGAAGTAGGCAAACAACCAGAAGGTATGACGAGGTGGCTGACAGATAAATCCGATGAAATGACCTCAAATAGTCATGCACATGTCGAAAGTTGCATGTCTGATGCTGAAGATCACTCGACAGGTGCAGCTGGCACTGAGGTATATCCACTTCTAGGAGGTGATCCAACAAGCCAAGGGCCCATGCATATTCATGATCAGAAGGTTTTCTCCGCGTCGTGTTCAAATTGTAATACAGATAAAGCATGTGCAGATTCACCATTTGCTGATGACTTTGGCAGCAATCACAATGGATTGCACTTTCAAGATGGTACCTCTGAACAGGATGTTTCCTTAACAGAATTGTTAGATAACCTTCAGAAACATGAGGAATATCCTTGTGAGGAGTCAAGAAGTAGTAAATTCTCGGATATTGGTAGCAAGAGCATTGTGTCTGGGAACAGTTATCCGCTCCATCAAGGGCCAGAGAAGAGCGAAAATCTCCGTGTCTCTGGTTTATTCACTGAGAATGGTGGTAGTATGTTTCGAGGACAGGTTTGTGGTTCACCACAGTCGCTCCAGGCTCAAGTATCCAACTATGATGCAGAGGGCAGCATGAGCAATTTATGCTCACTTCATGATCAATATGCTGAAAACAATATGTCACTCGCCTATTCTGTGATGCAATCTCTCCACAACCCCGAACAATCTTTTGATCATATAAATCCTGCTAGTCATGGTAGCAATGTCGATGATAGATCTGGGATCAGAATTCGTCAGCGTCAGCGACAAAATCATGCAAGTCCTGAAAATCTTTCTACACAGGGTACTGCAGCCAGAAGAATACGTTTGCAGATGGATAACACCCAAGCATGCTGTGATGCCAGCAGCTCTAGTGATGGAATGCAAGAAGAGTCTGTCATCATTGAGGCCAGTGAGGATTCGTGGAATGGTTCAGAGGTGGGAAGAACCATTAATGACCACAGGAAGATTGTTGAAGAAAAAGGAAAAACGGGAGTTGGGTTGATAAATAGAAATAGGAAGGAAGATATTGGTAAAGGAATGAGGTCAATGTTTTCATGGAGGACAACGCATGGTGTCTGGGGAAGAATTTATGCGATCAGCTTGTACATAGTTGTAGCATTGTCGGTAATATTTGTTAGGATATCTCGGTGTCCTAGCTTCCGTGCTTTAGATAATTGA

>CrNAC-32

ATGGAAAGGGAAGCAGCTAATATGGTGTTGAATATTAATGAGGAAGATCGAGAAGATGATCATGATTTGATGGATTTGCCTCCGGGTTTTAGATTTCATCCCACAGATGAAGAAATTATCTCTTATTATCTTACGGAGAAGGTTATGAATAGTGGATTCTCAGCTAAAGCTATTGGTGAAGTTGATATGAACAAGTGTGAACCTTGGGATTTGCCCAAAAGAGCAAAGATGGGATCAGAAAAAGAATGGTTCTTCTTTTGTCAAAGAGATAGGAAATATCCAACAGGGATGAGAACCAATAGAGCAACAGAATCTGGATATTGGAAGGCAACTGGAAAAGATAAAGAGATTTATAATAGTAATAATAATAAATCAGGAAAAGGTGTTGGTGGTGGTGGTAATTGTGTTGTGGTGGGGATGAAGAAAACTCTTGTTTTCTACAAAGGAAGAGCTCCAAAAGGGGAAAAAAGTAATTGGGTCATGCATGAGTACAGACTTGAAGGCAGATTTTCCTATTATAACTTTCCTAAAGGCGCTGCAAAGGATGAATGGGTTGTTTGCAGAGTTTTCCACAAGAATAATAATAATAATAATATTTTGAGGAGGGTTGATTCTTTTCTGGATCATATTTTGGATAGTCCTAATTCGCTACCACCATTAATGGATCTTCCTAGGATCAGGAGCTCTAGTACTGCTGCTGATAGGCCAGCAGCAGCAGCAGCAGCTTCCACCACCAGCTATACTCACGATGACGAAGACGAATTATTCAAACCTTCTATTTCTGCTTCTGCTTCTTCCTCCCAATCAGTACTACTACAAGAAGATCAAAAAAACTTTACCATACCCCCAATTAATTATTACGCCACCCCGACCCCGACCCCGACCCCGACCCCGGCAGCTTTCATGATCAACAATTCTACTTACCAACCAAGTTCTAGCTGCCAAAATCCATTATTAATCATTCCAAACTCCATTTTCTGCCCTCAAAACTCACAGGATCCGAATCTATATGAAACTCAAGACCGAAATCTTGGTTCCATCCTGAATTACCTAGGAGGCTCCGAATTCAAACCGCCGGCTGAATTTGCGGATTATTCTTCTTATTTGGAGGCTAGTTTAGTGCAACAAAGGATGGTTCTAACTCCGAATTGA

>CrNAC-03

ATGGCACCAGTCGGATTGCCTCCGGGATTTAGGTTCCATCCGACCGATGAAGAGCTTGTGAATTACTACCTAAAGAGGAAAATTCATGGACAAGAAATTGAACTTGATATCATTCCTGAGGTTGATCTCTACAAATGTGAGCCATGGGAATTAGCAGAAAAATCATTTTTACCAAGTAGAGATCCAGAATGGTACTTTTTTGGACCAAGGGATAGAAAGTATCCAAATGGATTCAGAACAAATAGAGCAACTCGAGCTGGATACTGGAAATCAACAGGGAAAGACAGAAGAGTTTCTTCCCAAAATAGACCAATAGGAATGAAGAAGACATTGGTTTATTACAGAGGGAGAGCTCCACAGGGAATCAGAACTGATTGGGTAATGCATGAATATCGACTTGATGATAAGGAATCTGATCAAGATACTTCCGGCATTCAGGACTCGTATGCATTGTGTCGGGTGTTCAAGAAAAATGGGATCTGTTCAGAAATAGAAGAACAAGGGCAAAGCAGCAACAGCTTATCTTTGCTTGATTACTCTTCTCAAGGAGTAGTTATAAATGAATATGAACAAACATTTTCACCAGATGTTCCCTTAGCTTCATCTTCATGTATGGATGAAGAGGACAAAGAAGACAAAGATGATTCATGGATGCAGTTTATTACAGATGATGCATGGTGTTCATCAACTAACCCTTTTGGAAATGAAGAGGTTGTGGCTCAATTAACATTCACAAACTAA

>CrNAC-38

ATGTTGAATTGTTTTACAGGGAAGTCATTGTTGCCAAGCAAAGATCTAGAATGGTATTTTTTCAGTCCGAGGGATAGAAAATACCCGAATGGATCAAGGACTAATAGAGCAACGAAAGCTGGGTATTGGAAGGCAACTGGAAAGGATAGAAAAGTAAATTCACAAATGAGGGCAGTGGGGATGAAGAAAACCCTAGTTTATTATAGAGGGAGGGCACCTCATGGAGCTCGTACTGATTGGGTTATGCATGAATATCGCTTAGATGAACGTGAATGTGAAACTAACACCGGTTTGCAGGATGCATATGCACTTTGTCGCGTTTTCAAGAAAAGTTTGAACATTCCAAAGATAGGAGATCATTATACTAGTGCAACAGCGGCAGCTAGTGATAGATCTTCCAGCATAGATCCATATTCTGCAGATGATATTGAGAGTTTTGATTATGCAATGCCAACAGCAACAGTAATTAGTGATAATAATAATAATAATTCTAACTATCATAACGTTTCGGCATCAGCTGCAAGTTCTTCTAACATCATCCATGGCAGCTCTCCAATGAACACTACTAGTACTGCTGCTGCTCCAACCCATGATAGTAGATGGATGCAATACTTGTCCGATGAAGCATTCAGTTTTAACAATAACCCCTCTTTCCCAAATTACATGAACAACATGCCATATCCTCCATCCAAGGTTGATATAGCATTAGAGTGTGCAAGATTGCAGCATAGGTTTGCATTGCCACCATTGGAAGTTCAAGACTTTTCCCAAGCAGCTGGTTTTGCTGACCATGCTAGATCCATGGGACAAACAAGTTACAATATTCAAGAACACAACAGCAATAGTAATCATCAGCAGCCGGATATTGTACAAGAGATTCTTTCAGTAGCTGAAGCTTCTCAAAACTTTATGAATCAAGATCATACAACTACCTTTGGAGGAAATTATACTCATGACCATGATGATTTCACATTTTTCTCCCCCAACAATAACCAAATGTACGATCAAAGTTTCAGTAGGTCCATTGAAATTGGAGGCCTCCAGAATGAAGAATTCATTAGATCTGACAGAATGGTTGAAAACTTGAGATGGGTTGGAATGTCTGACAAAGATCTTGAGAAGACCTTCTTGGAAGATTACAAGACAGTTCCAATAGAAAACATTTCAAGTTTCCAAAGACAAGAGGAGAATCATCAGGTTCATGTTCAAGGTTGATATAGCATTAGAGTGTGCAAGATTGCAGCATAGGTTTGCATTGCCACCATTGGAAGTTCAAGACTTTTCCCAAGCAGCTGGTTTTGCTGACCATGCTAGATCCATGGGACAAACAAGTTACAATATTCAAGAACACAACAGCAATAGTAATCATCAGCAGCCGGATATTGTACAAGAGATTCTTTCAGTAGCTGAAGCTTCTCAAAACTTTATGAATCAAGATCATACAACTACCTTTGGAGGAAATTATACTCATGACCATGATGATTTCACATTTTTCTCCCCCAACAATAACCAAATGTACGATCAAAGTTTCAGTAGGTCCATTGAAATTGGAGGCCTCCAGAATGAAGAATTCATTAGATCTGACAGAATGGTTGAAAACTTGAGATGGGTTGGAATGTCTGACAAAGATCTTGAGAAGACCTTCTTGGAAGATTACAAGACAGTTCCAATAGAAAACATTTCAAGTTTCCAAAGACAAGAGGAGAATCATCAGGTTCATGTTCAAGGAGAGAGCAGTCATCAAAATAATCTAAAGGAAGGAATTATGGAGGATCATCATCATCATCATCAAAATGATTACTCACTTGGATTTGGGAATAATCATGGTGATGAGAATAATAATGGTGAACATTTCTTAGATGATGATGGAAATATTGTAGATCATGACTTCTCTAGCAGTCCAAACTTTGATGAAGTATTTGAGAAAATAGAAGTGAGCCATGGAATGCTTATATCAACTAGGCAGACAGCCAACACATTCTATCATCAATTGGTACCTTCAAAGACTCTTAGGGTTCATCTTCATCCAATTCATCATCAAGATTTTACAATTACCAAATCAGATTCAAGAAAAGTACCAAACAATTCTAATTATAGACATGTCACCAACAAAGTCACAACAATCTTGATTGCAATTATTGCAAGTATGATGGCAGTACTAAATGCTTACTGGATATGTTACGGAGAATGTTTGACAGAGAAACAATGCTTTAAAGATGATGATGAGACGATAGAGGATGAATTTTCGAGCAAGAAAATAATGGGGTCGGTGGCGCTGGTGGCTGGCGGTGGCGGAGGAAGCATTTCTAGGATG

>CrNAC-20

ATGGGGCAAGAATTGGTTGCCGTCACACCTGCGACGCCGGTCGGAGGTGGGATTAGTGGGTCTGCGCCGCCGGCAACTTCTTTGGCTCCGGGTTTTAGGTTTCATCCGACTGATGAGGAGTTGGTTAGGTACTACTTAAGGAGAAAAGCCTGTGGAAAGCCCTTTAGGTTTCAGGCGGTGTCTGAGATCGATGTCTACAAATCTGAGCCATGGGAACTCGCATGCTTCTCATCTCTGAAGACAAGAGATCTAGAGTGGTATTTCTTCAGCCCAGTAGATAGAAAGTACGGTAATGGGTCTCGGCTCAACCGTGCTACTGGGAAAGGGTACTGGAAAGCAACTGGGAAGGATCGTCCTGTACGCCACAAGAACCAGACCATTGGGATGAAGAAAACTCTTGTGTTCCATAGTGGACGAGCTCCTGATGGTAAGAGGACAAACTGGGTAATGCATGAGTACAGACTTGTAGATGAAGAATTGGAAAAGGCTGGAGTGCCACAGGTGGTGACTAGTGGTCTAGGACCGCCAAATGGTGACCGTTATGCTCCATTTATTGAGGAGGAATGGGATGATGATGCAGCTCTTATGGTGGTTCCTGGAGGAGAGGCCGAAGATGATATGGCCAATGGTGATGAAGCAAGAGTCGGCTGCAATGACCTTGACCAGGATATGAATAAGGCTACTCGCCGGAGCGAGAATCCGGTTGAGGGCATGATTCCGTTTATGTGCAAGAGGGAGAGATCAGAGGAACCAGAGCCTCTCTCCTTAGGCCAAGCTAAAAGATCAAAGCACGAGGATCCAAACTCCAGCCATGCCAACGGTTCAGAAGATTCGACCACCACAAGTCAGGATCCCACCTTGATGACGACGACAAATTTTTCCTCCGCACTTTTGGAATTCCCCTTACTAGAATCTGTCGAACCTAAAGAAAGCCAGCCTTCCAACCCGCAGACTTTCGACTCTTCAAATCTCGAGAAGTCTGTGCCCCCCGGTTATTTAAAGTTTATCAGCAACTTGGAGAATGAGATACTAAATGTTTCCATGGAGAGAGAGACTCTGAAGATTGAAGTGATGCGTGCACAAGCGATGATCAACATCCTCCAATCGCGCATTGATCTTCTGAACAAGGAAAATGAGGACCTAAGGAGACTTGGACGAGGCGGCTAG

>CrNAC-25

ATGGGTTTAAGGGACATTGGAGCAAGTTTGCCACCTGGTTTTAGATTCTATCCTAGTGATGAAGAGCTTGTTTGCCATTATCTTTACAAGAAAATTGCTAATGGAGGAGAAGTTTCTAAAGATACCTTAGTTGAAATTGATCTCCATACTTGTGAGCCATGGCAACTTCCTGAGGTGGCAAAGCTAAATTCGACAGAGTGGTACTTCTTCAGCTTCCGTGACCGAAAGTATGCGACCGGGTTCCGAACAAACAGAGCGACAACGACCGGCTACTGGAAAGCCACCGGCAAAGATCGAACGGTGATCCATCCGGTAACACGTTCAGTAGTTGGAATGAGAAAGACATTAGTGTTCTACAAGAATAGAGCTCCTAATGGTATTAAAACCGGTTGGATCATGCATGAATTTCGCCTCGAAAACCCTCTTCTTCCTCCTAAGGTTCGTTCCTATATAAATATTAAATATACACCATCTATACCTATAATGTAA

>CrNAC-35

ATGAGTAGTAATAATAATATTCTAAGTATGGTGGAGGCAAAATTGCCACCGGGATTTAGGTTTCATCCGAGAGATGAAGAGCTAATTTGTGATTACTTGATGAAGAAGGTGGTGGGTGGCGGCGGTGGTTGTGATGAAGATCAAGTGCAGCGATATCCAGGAGTACGTATGGTGGAAGTAGACCTCAACAAATCCGAACCTTGGGAAATTCCCGAGAGTGCATGTGTTGGAGGGAAGGAATGGTATTTTTACAGCCAGAGAGACAGGAAATATTCAAGCGGATTAAGGACAAACAGGGCGACTGCAACTGGTTACTGGAAAGCCACAGGTAAAGATAGAGCTGTTTTCCGTAAAGCTAAACTTGTTGGGATGAGGAAAACACTTGTTTTCTACCAAGGAAGAGCACCTAAGGGTAGAAAAACTGATTGGGTTATGCATGAATTTCGCCTTCAAGGATCATCCCTCTCCTCTTCCTCCTCCTCAATTCATCGAATCATCAAGGAGGAGGATTGGGTGTTATGCCGGGTATTCTTCAAGAACAGAGAAATTCTCCCAAAACAACAACTAGTCAACGGCGCCGTCGTCCACGGTGGTGACCACCATGATATTATCTGCTCGTCCCTGCCGCCATTAATGGATCCTTACATCAATTTTACCCAAACTCATAATAATAATAATAATAATAATAATGAAACGAACGAGCAAGTGCCCTGCTTCTCCATTTTCACTCCAGCTGATCAAATTAGCATTAGCAGTAGCAGCAGCAGCAGCTGCCAACCATTCTCATACCTCATGGCTTCCAATACGTCGACGTATGATCCAAACTTAATTTCCAATATTATGGTGCCTAACTTTGGTGGTAATTTACCCGAAGAATTTGGAGGGAATAAAACGGTGGTGGTAAGGGCAGTCCTAAATGAGCTCACCAAGATGGAACAAAACAACAACAACCTTAATAACCCATTAATTACAGCCGTTAAATCTTCTCCAAGCTTTGGGGAAGCTGCTGCTAGTTCTGATAGTTATTTATCTGAAGCTGCTCTCTCTACCATGTGGAACCAATATTAA

>CrNAC-11

ATGGAAGGAGATCAAGTGAATAATAAAGGCGCTGACGAGACGCTGCCACCGGGGTTTAGGTTTCATCCGACGGATGAAGAGCTCATCACATATTATCTCATAAACAAGATCTCAGATTCAAGTTTTACAGGAAGAGCTGTTGCTGATGTTGATCTCAATAAATGTGAACCGTGGGATCTTCCAGGAAAGGCAAAGATGGGAGAAAAGGAGTGGTATTTCTTCAGCCTAAGGGATCGGAAATATCCAACGGGAGTGAGAACAAACCGGGCAACGAATACCGGCTACTGGAAGACGACCGGTAAAGACAAGGAAATATACAATAGTGTAAGTTCAGAATTGGTTGGGATGAAGAAAACATTGGTATTCTATAGAGGAAGAGCTCCAAGAGGTGAAAAAACCAATTGGGTTATGCATGAATATCGTATTCATGCTAAATCCTCCTATCGAACAACTAAGCAGGACGAATGGGTGGTTTGCCGAGTTTTCCAGAAGAGCGCTGGAGGAAAAAAATTCCCATCATCAAACCATTCAAGAGCAGCAGCACTACTTAATGCTAATTACAACATTAATATTGATCATCATCATCAAATCAACCAAAATTCAATTTCATCATCATCATCATCTCATCATCATCATCATCCTAATTTTCAATTTAACAATATTATGGGAATTAGAAACTACATGATGATGAACCATGCAGATATTCAAGAACAACAACTATCTTCCTCTTCCTCTAGGGTTTTGCCAATTCATCATCAATCCCAAATGATGAACTATGCTGCTCATCATATGTTGGCTACTTCTGGATCTGGTGGTGGAGGAGGTTGCTTTACCATATCTGGGCTGAATCTTAATCTGAACGGCGCCACCACCACCACCACCACCACCACGGCACAACAACAAGATGTTACTAATAATATTAATGGTGTTATTGGGAATAATGAGGAGGTGGTGGTTGGATATGGCACGGCGGATGGAGAGATGATGATGATGATGAACAATAATGCAATGGGAAATAGATTTCATGTGCCGCCGATGCCCATGGATCACTCATGTGCTGATTTAGACACTTACTGGCCTCCCTATTGA

>CrNAC-13

ATGATCTCAAAAGGGTCGTCGTCTTTAGCGCCGGGTTTCAGATTTCACCCGACTGATGAGGAGCTTGTTCGATACTATTTGAGGCGCAAGATTTGTTCGAAGCCTTTTCGTTTCGATGCAATTTCTGAGATCGATATTTACAAGGCTGAGCCTTGGGACCTTCCAGGTATGTCAAAGCTGAAGACCAGGGACCTGGAATGGTATTTTTTCAGTGTGCTTGATAAGAAGTATGGTAATGGATCCCGTACCAACAGAGCTACAGATAGAGGCTACTGGAAAACAACTGGAAAAGACAGGCCTGTATACCACAAGTCCCAGGTTGTGGGGATGAAGAAAACCCTGGTTTATCACAGTGGTCGAGCTCCAAAGGGTCAGAGAACTAACTGGGTGATGCATGAATACAGACTTATTGATGAAGAGTTGGGAAAGGCTGGAATTTCTCAGGATGCCTTTGTGTTGTGCCGAGTTTTTCAGAAAAGTGGTTCTGGTCCAAAGAATGGGGAACAGTATGGGGCACCCTTTGTGGAGGAAGAATGGGAAGAAGATGAATTGGAAATATTGCCCAAGGACGAGGCTGCAGATGAAGTTGAAGTTGGTGATGATGCCTATTTGGATGGAGATGACCTCAAGCAGATTCTTGCTGCAGATATACAAACAGATAATGTTTCTTTTACTCCGAATTGCTATACTGGGGAAGATGCTGCTTTTGTTGAGGAAGCTACAGAGTCCATTGGCGATTCACAGAAGCTATCAGTTGGCGCTGGGGAATATGATAATGGAAATAACCAATCTGATGACCAGAATGCTTATCATTTGCCAGTTCAGTATGATATGTATCAAAAAGCAGTCAAGCATGAATACATTGGTGAATCAAGCAAGACTGCTGATCCCCAGGGTGTAGACTATTTGCTTGAGGAACCATTTGTGGATGCTTCTGACAATCTTCAGTTTGGTGATGGAGGATTCCTTGAAACTAATGACCTCTCTAATCCAATTGATGCCGATGCTGCAGGTTTTGATATGCTCGAGGAGTATCTCACATTTTTGGATGCAGATGGTGATATTGCATTTGACCCTCTAATTATGGAAGGAGGTCAGAACAATATTTCTGGAGAATCATCTGCCGCAGAAAAGGACGTTGAAGAAGGAATTGAGACAGGCATGCCAAGCAAACAGCTTGAACATGATAATGATGCTACATCTTCCAAGGAGGCTTCTTCAAAATTTGGATCAGGTTATCAATATCCATTCATGAAGCAGGCGAGCCAAATGTTAGGCAGCTTTCCTGCTCCTCCTGCATTTGCATCCGAGTTCCCATCAAAGGATGTTGCTCTGCGTTTGAATTCTGCCTCTCAGCCTTCGAGTTCAGTTCATGTTACTGCTGGCATGATAAGAATTAGGAATATGAGCATGGACGGGCATGGAACAACAGACTGGTTCTTAGGAAAGCACAGTCAGTACAACGTGACCCTTTCTTTTGGCGTATCAGGAGGTGATGATAACTCTACAACCTTAGAATCAGCTGTCAGAATACTTCCAGGAAAGGCAGTCTCCCCAACACCCATCGGTTGGTTCTGCTTTATATTTTTTTGGGTCCTACTCCTTTCCGTTAGCTACAAGATTGGAACCTTAATTTGTGCTCAGTAA

>CrNAC-10

ATGGAGAATTATTCAGGTCTTATTAATGATGATGATGATCAGCTGGAATTGCCACCGGGTTTTCGATTTCATCCAACTGATGAAGAATTGATCACTCATTATCTGTCAAAAAAGGTTCTTGAAACCAATTTTTCTGCTAAAGCTATTGGTGAAGTGGATATGAACAAAGTGGAACCTTGGGATTTACCTTGTAAGTTTTTCAATCCAAATAAAAAAAAGGGGAGAGCAAAAATGGGGGAAAAAGAATGGTATTTTTTCTGTGTTAGAGACAAAAAGTACCCAACTGGTTTAAGAACAAACAGGGCTACTGCTGCTGGTTATTGGAAAGCTACTGGAAAAGATAAAGAAATTTTCCGTGGAAAATCTCTTGTTGGGATGAAGAAAACTCTTGTTTTTTACAAAGGAAGAGCTCCTAAAGGGGAAAAAACCAATTGGGTTACTCATGAATATCGATTAGAAGGAAAATTTTCCCTTCTTAATCTCCCCAAATCTGCTAAGAATGAATGGGTGATTTGCAGGGTTTTTCAAAAGACTGCTGGTGGAAAAAAAGTTCATATTTCTGGATTAGCAAGATCAAATTCATTTGGGAATAATGATTTGGTTTCTTCTTTGCTTCCGCCATTGATGGATTCTTCGCCTTATAACAATGGAAATTCAACAAAATCCACCCCCGTGGCGGCGGAATCAGGTCACGTGCACTGCTTCTCCAATTCAATTACTGCTCAAAAGAGCCAACAAGAAGAAATCTTCAATTATTTCAACAACAACAACAACAACAATCCATTTGTTGTTTCTTCAACAAATGGAGGATATTCTTCTTCCTTCACAAATGGTGGATATTCTTCTTGGGGAGGTCAACAACTTCTTGTTCCTCCTCCTTCTATTGTTCAAGGAAATTACCAATTTTCACCTTCATTTCAAATGCAAGATCCTTCAATTCTGAGGAGTTTATTGGAAAATTGTGGACAAAACATGATGAAACAAGGGTTCAAAATAGAGAAAGATGGAATTAGTGGATCACAAGAAACAGGGGTTAGTACTGATATTAATACTGAAATTTCTTCAGTTGTTTCAAATCTTGAAATGGGAAGAAAATCATCCCTTGAAGATCATCAAGAAGTACCACCACCATCCTCATCATCTGCTGCTGCTGTTGTTGGACCTCAAGATCTTGATTGTCTTTGGGGTTATTGA

>CrNAC-05

ATGGAAAGAGATGAAAAGATGGATTTGCCACCTGGTTTTCGCTTTCATCCAACTGATGAAGAGCTAATAACTCATTATTTATCCCCAAAAGTTCTTGATAATAGTTTTTCTGCAATTGCAATTGGGGAAGTTGACTTAAATAAAGTTGAGCCATGGGATTTGCCATGGAAGGCAAAAATGGGTGAAAAAGAATGGTATTTTTTCTGTGTTAAGGATAGAAAATATCCAACAGGATTAAGGACTAATAGAGCTACTGATGCTGGTTATTGGAAAGCAACAGGAAAAGATAAGGAGATTTTCAAAGTGAAATCACTTGTTGGGATGAAGAAAACTTTGGTTTTTTACAGAGGTAGAGCTCCCAAAGGAGAAAAAACTAATTGGGTTATGCATGAATATAGATTGGAAGGTCAAAATTCCATCCACAATTTGTCTAAAGTTGCTAAGAATGAATGGGTGATATGTAGAATCTTCAAGAAAAGTACAGGAGGAAAGAAAGTTCATATTTCAGGGTTAATAAAGATGAACAATTATGGTGATCAAGAAGATTTAGGAACTTCAAATTTGCCTCCATTAATGGATTTATCCTCTGAAGAAACCCAACCAAGAACGACAACAACAATTGCTGAGGCATCTCACGTGACCTGCTTCTCCAACCAAATGGAGGACGGCCAAAAACCTCAAATGCCTGCTTCTTCAATTCTTTCTTCACAAATAATGCCTTACTTAGAAAGTATGCAGTTTGGGGATTCAATTTCCATGGATGATCACTCAATAATGAGGCTTTTAATGGACGAAAATGGAGGAAATTCAAGACAAAATCCTAATGCTAATAATAATCAAGAATTCTCCCAAGAAACAGGAATTAGTACCGACATTTCATCCCCTGTTTCGAACAGGGTAGAATGGAGGAGGCCTAGTTATGGGCATCATCAAGAATTTCATCAGATTAGTTCTGCAGGGCCAGTTGATCTTGATTGTTTATGGAATTATTGA

>CrNAC-22

ATGGCTTCATCAAACGGCGGCGTTCCACCAGGATTCCGCTTTCACCCCACGGATGAAGAGTTGCTTCATTACTACTTGAAGAAGAAAGTTTCCTTTCAAAAGTTTGACATGGAAGTTATTAGAGAAGTGGATTTGAACAAGATTGAGCCATGGGAATTGCAAGAGAGATGCAAGATTGGAACCACACCACAGAACGAATGGTACTTCTTCAGTCACAAGGATAGAAAGTATCCAACAGGGTCAAGAACAAATAGAGCAACAAATGCAGGGTTTTGGAAGGCAACAGGAAGAGACAAATGCATTAGGAACACATTCAAAAAGATTGGTATGAGAAAAACATTAGTTTTCTATAGAGGAAGAGCTCCTCATGGCCAAAAAACTGATTGGATTATGCATGAATATCGACTTGAAGATGCCGATAATAATGACCCCCAAGGAAATTCCAGTGAGGACGGTTGGGTAGTTTGCCGTGTATTCAAGAAAAAGAACTTATTCAAAGTCGGAAACGACGGAGGAGGAGGCACCGCCGGCGGCAGTATTCATATAGGATCCGACCCGCTTAACCATACTTCCTCCCGTGCCACATTCATGCCAAGGGACAATCAATATTTATTACATCCTCAACAACATCATCATCATGGACTCAATTATTCTCACAATATCCCAAACATAACTCTCCCACCTCATTATTCCCAAATTCAACCACAAAATTTCATACCAACACATAATAACAAGCCATTAGATTATCATGACTTTTCGGAACAGTCACCTATAATGGTCAAACAACTCATGGCTGCAAGCAGTACGTGTGAGTCATCGGCTAATTTGGGAGTAACAGCAGCAACTGAAAATTGTAATGAAGAACAACAACATGAAAATCATTTAAATGAATGGGGAATGATGGATGGACTTGTTACTTCTTCTTCACAAGTAAATATAGGAGGAGGAGGGAATATTAATCCCAACGATGAATCTTCTCACATTAATCAACTCTCTCTACGTAGCGAGATGGATTTCTGGGCTTATGGCAAATAA

>CrNAC-08

ATGAATACATTTTCACATGTACCACCTGGCTTTAGGTTTCATCCTACAGATGAAGAATTAGTTGATTACTATCTTCGAAAGAAAATCGCTGCCAAAAGAATCGATCTTGATGTTATTAAAGATGTCGATCTTTACAAAATCGAACCATGGGATCTTCAAGAACTATGCAAGATAAGTAACGATGAGCAAAACGAATGGTATTTCTTTAGTCATAAAGATAAGAAGTATCCAACAGGAACAAGGACTAATAGAGCTACTAAAGCTGGTTTCTGGAAAGCAACTGGAAGAGACAAAGCCATTTATTCTAAGCATAGCTTAATTGGTATGAGAAAAACTTTGGTTTTTTATAAAGGTCGTGCCCCTAATGGACTTAAGTCTGACTGGATCATGCATGAGTACAGACTTGAAACTAATGAGAATGGCACTCCTCAGGCAAGCCCCCCCTACCTCCATCTCCTTATTTATTATGAAGAAGGATGGGTAGTGTGTAGGGTGTTCAAGAAGAGGCTGGCAGCTATAAGGAAAGATGGAGAACATGAACAAATGTGTTGGTATGAGGATCAAGCGAATATCTCAATCGCCATATGCTTCATACAATAA

>CrNAC-27

ATGATGAGTGCTGGTGCAGCTGTGATGAACAGTCAGCTTTCTGTTCCTCCGGGATTTCGATTTCACCCTACTGATGAGGAGTTACTTTATTATTATCTGAGGAAGAAGGTCTCTTACGAGCCCATAGATTTGGACGTTATCAGAGAATTAGATCTAAACAAACTCGAACCTTGGGATCTAAAAGATAAATGTAGAATTGGATCGGGTCCTCAGAATGAATGGTACTTTTTTAGCCACAAAGACAAAAAGTATCCAACTGGAACGAGGACAAATCGAGCAACAATGGCTGGATTTTGGAAAGCTACAGGGAGAGACAAGGCCATTCATCTCAGCACTGCCTTAGCCTCAAAGAGAATTGGAATGAGAAAAACCCTTGTGTTTTACATAGGACGTGCTCCTCATGGCCAAAAGACTGATTGGATCATGCATGAATATCATGGCTGGGTGGTGTGTAGAGTATTCAAGAAGAAAAACATTAACAGAGGAGGATTCCAACCGATTGAAGCTGCTGCTCAGGAAGAACATTTAGCTCACATGGAGGTGGCGGCGGCGGCGGCTACTACTTCAACGTTGTTTATGAATCCAAAATTACCAAATTCTGGTGGAGCTCTTTGTGATTATACTACGTTTGATAATTCCATGCATCTTCCCCAACTGCTAAGCCCAGAGTCGGCAGCAGCGGCAGCGGCAGCCCATGCACATCCGGCGGAAAAGTTCGGCAGCGATTGGTCATTTTTGGACAAGCTTCTTGCTTCACACCAAGGTTTCGATCGAAGCAAATGCTACCCATTGTCTCAATTTTCTGATGTGCCGGCGCCGGCGCCGGCAGCGGCGTCTTCGGCTGATCATCAGAAGTTTTCGTGCCACTACTTATTAGGTTCTCAACTCGATTTCTTGAATTTTTCCAAGTAG

>CrNAC-14

ATGGTTGAAGAAGAAGAGAAGAGGGTGATGATGGAGTCCATGGAATCATCATGTGTTCCTCCAGGATTTCGATTTCATCCGACGGACGAAGAACTCGTAGGTTATTACCTAAGGAAGAAAGTTGCATCTCAAAAGATTGATCTTGATGTTATTAGAGATATTGATCTTTATAGGATTGAACCATGGGATCTTCAAGATAAATGTAGGATTGGATACGAAGAACAGAATGAGTGGTATTTTTTCAGTCACAAAGATAAGAAGTATCCAACAGGAACAAGAACAAACAGAGCAACAATAGCTGGTTTTTGGAAAGCAACAGGAAGAGATAAGGCAGTTTACGATAAATCAAAACTAATAGGAATGAGGAAAACACTTGTGTTCTACAAAGGAAGAGCACCTAATGGACAGAAAACTGATTGGATTATGCATGAATATCGTCTTGAGTCTGAAGAAAATGGTCCTCCACAGGCAAGTTGGGTAGTGTGTAGAGCATTCAAGAAAAGAACAACCGGACAAGCGAAAACCGTTCACGAAACTTGGGACACAAGTTACTTCTACGACGAACCACCCACCGCTACTACTAATAATACTACTGTTAATACCACCACAGCCACAGTAGTCGATCATCATCCCATTAATATTGATTACATCACAAGACATCATCATCATCAGCCGCCGCCGCCGCCACCAAACTTTCTTCATCAACGTCGTCAGAATACTAATTTCCTATGCAAACAAGAAATAGAATCATCAGAAAACATCATTAATAGCTTCATAAATTCCTCCTCCGATCCATTCGGACAACTTCCACAATTAGAAAGTCCGTCAATGCCCTTAGTAAAACGACAGAATTCAATATCTTTGATATCGGATTCGTCTCCGGAAGAAGAAGATGGACATCTTGGTGGTGGAAGTGGAAGTAGCAGTAGTAGTAGTAAAACGGCGGTGACGACGGATTGGAGAGCACTTGATAAGTTTGTTGCTTCTCAATTGAGCCATGGAGATGGAGTTGGAGTTGGAGGGGTGGATGATAGTACGGAAATGGGAATGCTATTAATGCAGAGTGGAAGAGAAGAACATGAAGGGAATAAGTTTATGAGTGAATTGTTTTTGAGTTCTAATTCGGATTGTGATATTGGCATTTGTGTATTTGATCATAAGTGA

>CrNAC-36

ATGAATGCGTTTTCACATGTTCCTCCTGGATTTCGTTTCCACCCCACTGATGAAGAATTGGTTGATTATTACTTAAGGAAGAAGGTTACTTCTAGAAGAATCGATCTTGATGTTATCAAAGATGTTGATCTTTACAAGATTGAACCATGGGATCTTCAAGAGCTATGCAGAATAGGCACAGAAGAGCAAAATGAGTGGTATTTTTTTAGCCACAAAGATAAAAAATACCCAACAGGAACAAGAACTAATAGAGCAACAGCAGCAGGATTCTGGAAAGCCACAGGAAGAGATAAGGCCATTTACTTTCTGTCTATGCTGACTGACACACGCGATCGTTTTGGCAGGAAGAAGGATCATCATTTTCTTCAACAACTACCTCTTCTTGAGAGTCCAAAATTGCTACAATCTGCACCATCATCAGTAGTCCCAAATTCAATGCCTGCTGCATTTGGTCTTAATATGAACCATGGAAGCTCTTTGCAACCTTCACTGTTACAGGTCCAGCAGGAACATATATTGCATCAGTCTCAAGATCATCATCATCCCCTGCATAATTCGGTTTATAGTAGCGATATGAGCAATGATCAAGGAGGAGATCAAGTAGCAACAGATTGGCGAGTTCTTGACAAATTTGTCGCGTCCCAACTCAGCCAAGAAGAACTATCAAAGGAAAATGACTACACCAATGCAAACAACAACTTTCGTGGTTCTGATGATTCGAATATGATGATCAAGGATTCGGAAAAACAGGAAATGGCAACAGAAAATGTCTCAACTTCATCCTCAAGTTGTCAAATTGATATGTGGAAGTGA

>CrNAC-15

ATGAGCATATCTGTAACCGATGAATCCGGCGGCGGGGTTCCACCTGGATTTAGATTTCATCCCACGGAGGAGGAACTTCTGCACTACTATCTGAAGAAGAAGATTGCACACGAAAAGATTGACTTGGATGTAATCCCAGATGTTGATCTCAACAAGCTAGAGCCATGGGATATTCAAGAGAAATGTAAAATAGGATCCACACCGCAAAATGATTGGTATTTCTTTAGTCATAAAGACAAAAAATACCCAACTGGCACGCGTACCAACCGCGCAACTGCTGCAGGGTTTTGGAAGGCCACTGGCCGTGATAAGGTGATCTACTGTAATTCTCGATGCCGGATAGGCATGAGGAAGACGTTGGTTTTTTACAAAGGACGAGCTCCCCATGGCCAAAAGTCCGACTGGATCATGCATGAATATAGACTACTGGATGACAACCTCATGATCAGCACCACTAATAATAATGCTGTCGATGTGAATAATAATGTCTGCATTAATGCCATGGAAGAATTACCACCAGTACTACAGGAAGAGGGTTGGGTG

>CrNAC-17

ATGGCACCAGAGCAGATGAACCTTTGTGTAAATGGTCAATCTCAAGTGCCTCCAGGATTCCGTTTCCATCCAACGGAGGAGGAGCTTCTGCATTATTATTTGAGGAAGAAAGTTGCCTCCAAGAAAATTGATCTTGATGTAATCCGCGATGTTGATCTCAACAAGCTCGAACCCTGGGATATTCAAGAAAAATGCAGAATTGGATCCACTCCGCAGAATGATTGGTACTTCTTTAGTCACAAAGATAAAAAGTATCCAACAGGAACTAGAACAAACCGTGCAACTGCAGCTGGATTTTGGAAGGCAACGGGTAGGGATAAAGTCATTTATAGCAACTCTAAAAGAATTGGAATGAGGAAAACTCTAGTCTTCTATAAAGGGCGTGCCCCTCATGGACAGAAGTCAGATTGGATCATGCATGAGTACAGACTCGAAGACACCACCCCGCCCCATGATGCATCATCTCTTGCGTGCAGTATGGTAGGAGAGGGAAATTCTGAAGAAGGGTGGGTTGTGTGTCGAGTGTTCAAAAAGAAGAATTACCATAAAGCCCATGTGGATCAGAGTAACTCCCAAAACTCAATGATGGATCTTCCGGAGGGAAGTCTAATTGGAACGTCCAAAGATGGTATTCTTGATAGAATCCTTGTATACATGGGAAGATCCTCCTGCAAACAAGAAAACCCACCAATTAATAACATCCATGACGAAGACGACGTCGCCATGCAATTCATCAACAATAACCCAATTATGTCCGATGATAAATTCATGCATCTTCCTAGGCTGGAGAATCCATTATTCAACCAACATGATTGTAGTAGTTTCAGCGCCTCGCAGCCTTCAATGAATGATATAGCCGAAACTAATTCAGCTTCATGCACCACCGAAGCAGCCGCCGGGCTGAGTGACTGGGTGGCTCTTGATAGACTCGTGGCTTCTCAGCTAAATGGACAGGAAGAGGTTTTCTGCTTTCCGGCGGCGGCGGGATCCATCAGATCACACGGCTACCATCATCAACCACCTCAAACTCAAGCGTACGAGGGTGCAGGCAGCGAGTTGGACTTCTGGACCTTTGCCAGGTCATCATCCTCCGATCCGTTATGCCGCTTGTCAGTATGA

>CrNAC-47

ATGGAGGATCTAAAAGCTCCCGTTGTTCCAACCTGGAATTCGCTTCCGCCTGGTGCCCGATTTTACCCTTCTGAAGAACAGCTCGTTTACTACTATCTCAGTTCAAAAAATGATGGCAGTAATTATTATGGAATTGATGTAATTAGGGAAATTGATCTTTACAGTTATGACCCATTCAATTTGCCCGAAATCTCTTGCTTTCGGTTTGGTAGGGGAGGGAGGAGGCGCCACTGGTACTGTTATGTGGGAAGGATCATCAGAGAAAGAGGGAGGAGAAGAGCCGGGTCTGGGTATTGGAAAAAGAGAGGGAAGGTTAAGGATATTGTGGGCGGCGGAGCTGCAGAGAAAATTGCAATGGGGACGCGCAAAAGCTTTGTATTTTATTTGGGAGATTCGCCAAAGAATGCAGTTAAGACTGACTGGGTCATGTATGAATATGCTCTTGGTGATCAAAACATGATTAGTTCAAAGTTGAGTTCGGGATTGGCCTCCAGCGGTATGACCTATGATTCTCGAATGTCACTTCTCAGTCCAGAAGATCTAGAAGATTTGAGTAGTGCACAGAGGGGAAGCGCTTCTTTTGTCCTCTGCCGAGTGTTTTTGAGATCTTGTCCATTCAATAACTTAGAGAAGCACGTAGTTGTAAGTTTCTGTGGTGATGATAGTATTGCAACAGTGCATCGTTGTGTTGGTGTTCAGTGTGAGGGGACTACTGCATCTGTAATTGCAGAATCTAAAAGTCACAATAACAATTTTCATGATAATGACAATGAGGATTTGAAACTTTCATCTGGCCTGGATACAGTTAATCAAATTATTCATGAGCCAATGAATGAGAAGGTATCACTTTTATATCATAGTTGGCTTTACCCACTTATGTCATTTTTTTATTTTTTTTAA

>CrNAC-02

ATGGTTGGAGGAAATTTACCACCTGGTTTTAGATTCCATCCTACTGATGAGGAGTTAATTATGTATTACCTCAAAAACCAGGCTACTTCTAGGCCGTGCCCGGTTTCTATCATCCCAGAAGTTGACATTTATAAATTTGATCCTTGGGAATTACCAGAGAAAACGGAATTTGGGGAAAATGAATGGTATTTTTTCACACCAAGAGATAGGAAGTATCCAAATGGGGTGCGTCCAAATAGAGCAGCAGTTTCTGGTTATTGGAAAGCCACGGGAACAGATAAATCAATATATAGTGGTTCTAAATATGTTGGTGTTAAAAAAGCCCTTGTTTTCTACCAAGGAAAACCTCCAAAAGGTATCAAGAGTGATTGGATTATGCACGAGTATCGTTTGATTGAATCAAGATCTCAAGTACCCACCAAACAAAATGGTTCCATGAGATTGGATGATTGGGTTCTTTGTAGAATCTACAAGAAGAAAAATCTTGGAAAATTATCTATGGACATTAAAGTTGAAGATCAAAGTCAAGAAACATTAGTGGCAAATGAACTTGTTACTAGTCATGATGATGAACAACAACAACAAACATTCAAGTTTCCAAGGCCATGTTCATTATCTCATCTGTGGGAAATGGATTACATGGGCTCAATTCCTCAAATTTTTGGAGAAAATTCCATCTTTGATCAACAAAATATGTTCATGCTGAACAATAATAATAATAATAATGGAAATGTTAATACTCCTCGTCAATTAGGTGATCAAATGGGAAACCAATATTCAGAAGCCATGGTTAGATTCCAAGGAAATCAACCGGTTTATGTGAACCCGGTTTTTGAATTTCAGTGA

>CrNAC-24

ATGGATCATAATAATAATAATAATAAACAGACCATTTTCCAATTCCCACCTGGCGTTAGATTTCATCCCTCTGATGAAGAACTCATCGTTTATTATTTGCTCAACAAACTTAATTCTCTTCCTTTGCCTGCTGCTGTTATTGCTGAAGTTGAGCTCTATAATTATAACCCTTGGGACTTACCAAAGAAGGCTCTGTTTGGAGAAGATGAATGGTATTTCTTTAGTCCAAGGGACAGGAAGTATCCAAATGGAGCAAGGCCTAATAGAACAGCAGCTTCAGGTTATTGGAAGGCAACAGGAACTGATAAACCTATTCTAAATTCTTGTGGTGGAGAAAGAATAGGAGTCAAGAAAGCACTTGTTTTCTATATTGGAAAACCTCCAAATGGTAGCAAGACAGATTGGATTATGATTGAGTATCGAATTCCTGACACACTTATTATTCGACCTCAAAGATCTAAAGGTTCCATGAGGTTGGATGACTGGGTACTCTGTAGAATTAAGCAAAAAGGCAACATGTCAAAGAATGCATGGGATCATGTTCAACATAGTACCAGCAAACTGGTGGGAGACCTAACAAACATGAAAGAACAATTTCCATCAGTCAACACAAACAATGCTGCCTCAGACATCTGCTCAAACTATTTCCTATCCAAAGACTGCCATCTATTGGCTAAACTTCTTGCTACTCATCAATATTTTCCTAGTTCCATCTCCACTCTTTCTAGAACAACCTCTCAAAGCAGCAACAACAATGTCAAGAACTGGGATAGAGTCTATGAACACGGACAAGGTAAGGGTATTCCGGTGATTAACTCTTATAACTTCCATGGTTCTTTCAATTCACAAGAGAAGCCAAATGATGAGACTGAATATGGAAACTTTTCTCAACCAATACCACATTCAAACAAGCATGAGAATCTAGTAATCAGTAGTATGCTAGCTGCAAATGGTACGAGTTTCTGTAATCAACATGAATCTCAGGGAGCTGTATTCAAGAATAACTTGTCTAACGCCATCATGAACTTACAGGAGCTAGATGTAGCCGCATTTGCAGAAAGATTTCTACAGTGA

>CrNAC-01

ATGGAAAGTACTGATTCGTCTACCGGAGGCGGCGGCGGTTGTTCGCAGCAGCCTCATCTTCCGCCGGGCTTTCGATTCCATCCGACCGATGAAGAATTGGTGGTCCACTATCTTAAGAAGAAAGCTGCCTCTGCTCCTCTTCCAGTTTCCATCATAGCCGAAGTTGATCTCTCAAGTTTGATCCGTGGGAACTTCCAGCTAAGGCTACGTTTGGGGAACAAGAATGGTATTTCTTTAGTCCGAGGGATAGAAAATACCCTAACGGTGCCCGGCCGAACCGGGCGGCTACTTCTGGATACTGGAAAGCTACTGGAACTGATAAACCAGTTCTTACCTCTGGGGGAACTCAAAAGGTTGGTGTCAAGAAAGCGCTGGTTTTCTATGGTGGTAAACCTCCTAAAGGAATCAAAACCAATTGGATCATGCACGAGTATAGACTTGCCGATAATAAAACCAATTCAAAGCCTCCTGGATGCGATATTGCCAATAAGAAAGGCTCACTCAGGCTTGATGATTGGGTATTATGTCGGATCTACAAGAAGAATAATACACCAAGGCCGATGGATCATGATAGGGATGATATGAATGATTTGATGGCATCAATACCACCTTCAATATCTCTAGGCCAGCCAAAATTGCCAGGACTAAAAACGACCAACTATGGAGCATTACTTGAGAATGAACACAACCTGTTTGATGGAATGCTGAGTGCCGATTTAAACAGTGGTGGATCTATGTCCCAATTAACTTCATCCGCTTCTAAGCAACAGCTTTCTCTACTCGCCGGAGCATCCAATGTTCTCCCGGCGAAACGGACGTTAAATTCTCTGTACTGGAACGATGACGTCGGTAACGGAAATTCGCCGCCTACTAAGAGATTTCTCGCGGATAGTAGCGACGGAAGTATGGCTACGAGAAATGATGAGAACGCATCAATCGCTAGTCTTCTCAGTCAACTTCCTCAAACACCATCGTTGCACCAACAATCAATGTTAGGTTCTCTGGGGGATGGCGTTTTCCGTCCCTTATCAAGTTTCCGGTATGAATTGGTACTCTTAG

>CrNAC-09

ATGGGTGTTCAAGAAACGGACCCTCTTGCTCAATTGAGTTTACCTCCTGGTTTTAGATTTTATCCAACGGATGAGGAGCTTCTTGTTCAGTATTTGTGCAGAAAAGTTGCTGGCCATCATTTTAATCTTCAAATTATTGGTGAAGTTGATCTTTACAAATTTGACCCATGGGTTCTCCCCAGTAAGGCAATTTTTGGGGAAAAAGAATGGTATTTTTTCAGTCCGAGAGACAGAAAGTACCCAAATGGATCGAGGCCGAACAGAGTAGCTGGCTCTGGTTATTGGAAAGCTACGGGAACGGACAAGATAATCACCACGGAAGGGCGAAAAGTTGGAATTAAAAAAGCTCTCGTGTTTTACGTTGGCAAAGCACCTAAAGGAACTAAAACCAATTGGATTATGCATGAGTACAGACTCTCTGAACCCCCTAGAAAAAATGGAAGCGCCAGGTTGGATGATTGGGTACTTTGTCGAATTTATAAGAAGAATTCAGGCGGTGCAGCAAAGCAACTTTCCGGTGTTAATAGTAAAGAATACAGCCATGGCTCGTCGTCGTCTTCTTCTTCTCAGTTCGACGATATGCTGGAATCCTTGCCGGAGATTGATGATCGGTTTTTCTCATTACCTAGAATGAATTCTCTGAAAACTCTTCATCAAGACCAGAAAATCAATCTTCAGACCTTAGGGTCCGGGAGTATTGATTGGGCCACTTTAGCCGGACTCAGTTCGGTGCCGGAACTCCTTCCCGTCGGTCAGAATCAAGCTCATCAGATTCATCAGGCAGGACAGGGGAATGTGAACCCGAACGACGTCGTCAGCGGTCACAGAGATATGTTCGTTCCTTCGTTTCAACAAATGTGCCACGTAGATGAGGAAGTACAAAGTGGGCTGAGAACTCATAACCGGGTTGATAATTCGGGTTTTTTACAGCCGCCGCCGCAGCAGCAGCAGCAGCAGCAGCACAGCTCGAACTTGATGAGTTTTCATCAGAATTCGATAGACCCGTATGGGATCCGGTACCCGACCCAAATGGGAAATGTGGGGTATAGGCAGTAA

>CrNAC-12

ATGAATTCAATGGAGAAAAATCCCAAATCAGAAATAATATTTCAACTTCCAGCTGGTTTTAGGTTTCATCCATCAGATGAAGAACTTATAGTTCATTACTTGGGAAAAAAAGCTAACAAATTCCCACTTCCTGCTTCTATTGTTGCTGAAGTTGAATTATATAAATTCAACCCTTGGGATCTACCAAAGAAATGTTCATTTGGAGATGAAGAATGGTATTTTTTCACACCAAGGGATAGGAAGTATCCAAATGGAATGAGGCCTAATCGTATGGCAGGTTCAGGTTATTGGAAGGCTACTGGAATTGATAAGCCAATTGTTGCTTCTTGTGGATCAAAGGTTATAGGAGTTAAGAAAGCATTAGTATTCTACACTGGAAAACCCCCTAAAGGGAACAAGACTGATTGGACCATGCATGAGTATAGATTGCCTGAACCTGCTGCTCCTGCTGCTGATTCATCATGGACAACATCTACCACAAAACAAAACTCTATGAGATTGGATGAATGGGTACTTTGCCGGGTGAGACAGAAAACAGGAATGAATTTTGGAAACTTTGGAGAAGAAAGAAATGGTCTTAATAACAACAACAACAACAACAAAACAGTCCAAAAACCTCAAAAAACAGCTAATTATTCCACCTTTGAAATTGTCAAAGATTATGATGATCTTTACTACAAAGAATATGGTCCAATGCTACCATTTCTCTTCAATGATAACTCTTCTTCTCAAGATTTAGCTTCCACAGATCAGACAACAATAAGTTTTGAAGGAAAAAATGTTAAGGATTCATCTTCATCATCAGTTTGTGAAGACAACTTCAACATTGGCATTGGCATTGTCAATGGCAAGAGAAAAATGTTTGAAGATCAAGACAACATTATTAGTCATAAGAAATTAGCATATCAAAAGGAGATGGAAAATGATGATGATGAGCTACTACCAATTACACCTTCTTCAAACAACAATAATATTGATCAACTTTGCTCAAGCATGATGTATCAAGAGCTCTACACTTTGGCATCACATAA

>CrNAC-46

ATGGCTTTCTTCCACCATGTCATTATCAACATTGCCAATATTGTCGTTACTCATTGTGAGAAATTTCAGACAATTGGCTCTGATACCATGAAAATAAAGAAGAAGAAATTGCCTCGTAATCTGATATTACATGAGAATCTTTACCATGATCCCCCTGATGAAATCACAGGAAGATACAAGGAATTAACTACAGGGAATGAATGGTATTTCTTCACTCCAAGAGATGGAAAGTATCCTAATGGAGATCAGCCAAGTCGTGTTGCTGGGTCGGGTTATTGGAAGGCTATCAGAGCAGATAAACCTATTAAACATAATGGGAATGAGATTAGATTTAGAAAGGCATTAGTTTTTTACCAAGGAAAACCGCCTAAGGGTGAGAAAACTAGTTGGATTATGCATGAATCATGTCATGTAAAGGACGAAGGTACTAAGGAGAAAAGTAATGACACAGATATGAGGGTGGATGATTATATCTTATGCCGGATTTATAAGAAAAATGATAAGATGTTTAGGAGTCAACTATGTGGTGAGATTCAAACTCAAGATCTTCTTCTGGATGAAGAAAACAATATTAATATGACAAAAGAAGAAAATAAGACCGGAGTGAATTTAAGGGTGTATCCTTCCGGAAACAACCCCTTTGGATATCTGACAGAAGAACATATGTGGAGTCTACCACCAGAAAATCAAGTGCTTCCAGAATTTAATTTTCCTGAAATTAAGTATTATGATTCTGGTTTATCAGAATTGGATAATATTCTTATATATCCAAATCCAACCATGGATGATCATCATTTATCAATGCAGCAAAGTCAGGTGGATAATCCTTCATTACCATTAACAAAGAGCTGA

>CrNAC-07

ATGGCTGCAGAGTTGCAATTACCGCCTGGATTCAGATTTCATCCGACCGATGAAGAACTCGTGATACATTATTTATGCCGGAAATGTGCGTCTCAACCGATTGCGGTCCCGATTATAGCTGAGATTGATCTCTATAAGTATAATCCTTGGGATCTTCCTGGTATGGCGTTGTACGGGGAGAAGGAGTGGTATTTTTTTTCTCCGAGGGACAGAAAGTATCCGAACGGTTCGAGGCCGAATAGGGCGGCCGGAAGTGGTTATTGGAAGGCGACCGGTGCGGATAAACCGATTGGAAACCCGAAACCGATGGGGATTAAGAAAGCCTTGGTGTTTTACGCCGGCAAAGCCCCAAAAGGAGAGAAGACTAATTGGATTATGCATGAATACAGACTCGCCGATGTTGATCGATCCGCCCGTAAGAGGAACAACAGCTTAAGGTTGGATGATTGGGTTCTGTGCCGCATATACAACAAGAAAGGTACAATCGACAAACAACAAATGATGGGCATTCGTAAAGCGGCAAGTCAGGTGATTGAAGAAGAGGATAAGAAGCCGGAAATTCTGAAGTCGGTGCCGGAAACAACACCGGTGGTGTACAGCGATTTCATGTACCTGGATCCATCAGATTCCGTTCCCAAGCTGCACACGAACTCCAGCTCATCTGAGCAAGTGGTGTCGCCGGAATTCTCTCAGAACACATGCGAAGTACAGAGCGAGCCGAAGCTGAATGACTGGGAGAAATCCACTTCTGCCCTTGATTTTCCATTCAGTTACGACGGCAATGTGCTTATGAACACTCAGTTTCCGAGCAATTATCAGATGTCGCCTCTCCAGGATATGTTCATATTCCATCCGACTGATGAAGAACTCGTGATCCATTATTTATGCAGAAAATGTTCGTCTCAACCGATTGCGGTTCCGATTATAGCTGAACTTGATCTGTATAAGCATGATCCTTGGGATCTTCCTGGTATGGCGTTGTACGGGGAGAAGGAATGGTATTTTTTTTCTCCGAGGGAGCGTAAGTATCCAAACGGTTTGAGGCCAAACAGGGCAGCTGGGAGTGGTTATTGGAAGGCGACGGGGGCAGATAAACTGATTGGAAACCCTAAACCGATGGGGATTAAAAAGGCGTTGGTGTTTTATGCTGGCAAAGCACCTAAAGGAGAAAAGACTAATTGGATTATGCATGAATACAGACTAGCTGTTGTTGATCGTTCCGCCAGCAAAAAGAACAGCTCGAGGTTGGATGATTGGGTTTTGTGCCGCATATACAACAAGAAGGGTACAATCGAGAAACAACAAATGATGGGCATCATGAAGGCAATAACTCAAGTGATTGAAGAAGAGGATAACAAGACGAAAATACTGAAGCCGGTGCCGGAAACAACACCGGTGGTCTACAATGATTTCATATACTTGGATTCGTCGGATTCTGTCCCGAAGCTACACACGACGAACTCCAGCTCATCGGAGAAAGTGGTAGCGCCAGAATTCTCTCAAAACACATGCGAAGTACAGAGCGAACCGAAGCTAAACGACTGGGAGAAGTCAGCTTCCTTCCTTGATTTTCCATTCAGTAGTTATGTGGATGTGCCGCCGCCGCCGCCGATGGACAACGGCGGCAATGTGCTTATGAATTCTCAGTTCCCGAGCAATTATCAGATGTCGCCGCTCCAGGATATGTTCATGTTCCAGCAGAAGCCATTTTAA

>CrNAC-06

ATGGATTATATAGGTTATAGGGAGTCCATAGACAGCGGCGATCGAAATCAGAAGCTGGATTTGCCGCCTGGATTTAGATTTCATCCGACGGACGATGAGCTCGTCACGCATTACTTGTGTCGGAAGTGCGCTGCTCAGCCGATTTCGGTACCAATTATAGCTGAAATTGATCTGTACAAGTTCAATCCCTGGGATCTTCCTGGAATGGCTTTGTATGGTGAAAAGGAGTGGTATTTTTTCTCTCCAAGAGATAGGAAATATCCAAACGGTTCGAGGCCGAACAGGGCGGCAGGGACGGGGTATTGGAAAGCCACAGGAGCGGATAAGCCCATTGGGAAGCCTAAACCACTGGGAATAAAGAAGGCGCTTGTGTTTTATGCCGGAAAAGCCCCCAAAGGAATCAAAACCAATTGGATAATGCACGAGTACCGTCTCGCTAATGTCGACCGCTCCGCCGCCGGCAAAAGAACCAACTCCAGGCTTGACGATTGGGTATTGTGCCGAATATACAACAAAAAGGGTACTCTTGAAAAGTACAACAATGTGGATCAAATCCAAAATTCGATCGAATTCCAAGAAGACAAGCCAAGAATAATCGGATTTGGACAGTCAGAGACAGCCAGGAAATCTAATCCTCCGCCGATGCAACCAAATGTTCAACAAACTAGGAATGATTATCTGCATTTTGAAACTTCGGAGTCGGTGCCAAGGTTACACACAGACTCGAGCGGATCAGAGCAAGTGTTGTCTCCAGATTTCCAATGTGAAAAGGAAGTTCAAAGTGCGCCTAAATGGGATGAATTGGAGAGAACTCTTGATTATCAGATGAATTTCATGGACAGCTTCCAAAATGATGATGACCCTTTTGGTTCACAAATGCAGTACCATCAACACTTCTCTTCTCCTTTTCAAGATGTATTTATGTACATGCAGAAGCCCTTCTAA

>CrNAC-34

ATGTCCAATAATGCTGATGTTACAAATGACAATCATGGATCATCAGATCAGTGTAATAATTCTGATCGAAGGGATGAAATTCATGTAGTCAAGATTGATGAAGAAACAGAGGAATATTTTAAATCTTTTCCACCTGGTTATCGTTTTTGTCCAACTGATGCTGAACTTATTCTTCATTATTTGGAGAAGAAAATCAAGAATGAAAAATTGCCTCCTCATAGGATTCAAGAGGAAAATCTTTATAAATTTACTCCTGATGCTATTTCAGAGATGTATCCAGTACTAGGTGAAAGGGAATGGTATTTTTTCACTCCGAGGGATAGAAAGTATTCGAACGGGACGCGTCCAAATAGAGCTGCTGGAACTGGTTATTGGAAGGCTACTGGGGCAGATAAGCCTATTAGAAACAGTGGTTCTACCATTGGATTTAGAAAAGCATTGGTTTTTTATGAAGGAAAACCACCTAAAGGTGACAAAACTAATTGGATCATGCATGAGTATAGAGTCAATGCTGAGCCAAGGAAGAAGAGTTGTGCCACTGATATGAGGTTGGATGATTATGTATTGTGCAGAATTTACAAGAAGACCGATAAGTCATTCAAGGGTCGTCAAAGGAATAACGGAGGAGGGGATGATTATGTCCCTAATCAAAATGAATATCTTTCTGCAAATTCTCAGGATCATAAGAACAATGCACAGCCAGGAACGGATAATGTTCATTATGGAAATCATGGTGATGGTTATAATCACATGGAAGATTATTCAAAACTGGACATTCCCACTCATTTACAACATCATGAAATCCCTGACATTTCTTCTGCAGAAGATCAAGAAATTCCTAATTATGATTCTGGTAATTTTTTTCAATCCTCCTCTGACTATGGATTTGGGCAAACTCCTCCAACTTGTTTCCTGCCAATGTCTTCTATAAATCCGATATCTTCGTGGGAATCAAAGCCAAGATTTATGCCTGTGCCTCCATTTTATTCTACCACAAACAATGTGTTTAGGACGGGCTATAATATGAGAGAAGCTGCCACTGCATCATGGGATTTCAAGCCGCCTCCAAGAGGATTTCAAGACTTTTATGATTTTGATATTCATAATAATTTAAATTTTGTTGATCCTGAAGTCAATTTAGATAATATTCTTAACAACCATCCTTCTTCATCTGATTGCAGTCCCTTAAACAGTCTTCAAAAAAGACAAAGAGATTGA

>CrNAC-39

ATGGAACACTACTTTTCTTGCCCGAATTTGCAAGAGTTCTCTTCAAACAATCCAATTCCTACTACTACTACGACTACTACTACTACTACTACTCATTCCGAAACCAATTACACCCATAATAATATTAATGATCATCAATACATATTAGGCCAACCTACATTAGCTTCTTCTGGTGGTGGTGGTGAGATCGGGGGTATCGCGGCACTTGATAATTACAGCTTATTCGGAGATCAACAAGGTCATGATCATGACATAATTAATAATATTAATATTGATCATTGCATGAGTGGCTTTTATGACCATGAAAATCTTTTTCTTGATCAATATTACGAACCAATACAAGCTGTACCTGTTTCTGATGTACCTGATGTTCATGTCCATGTCGTCAATAATAATAATATAAATAATGAACGGATGATGCTTCCGTCTGGTTTCCGATTTTGCCCTTACGATATTGAGCTCATCCGAGACTATTTGATGAAGAAGATTGCCCATCCCCAACTTAATTGGGATCATATTAAACAAGTCCAGCTTTATGACTGTGATCCGTCTCAACTTGCAGCTTGTTATCCAAATGATGAAGGTGAATGGTACTTTTTCACTGAAAGGGACAAAAGATATCCAAACGGCGAGAGACCAAATAGATCAACAAGAAGTGGATATTGGAAAGCAACTGGAGCTAGAAGAAAAATTGTTGATAATGAAGGTATAGAAATTGGGAATAAGAGGCCATTAGTCTTTTATCAAGGTAAACATAAAGACAACCGAGAAAGAAGCAGCAAAGAGGAACCCAAAAAGACTGATTGGATTATGTATGAATATCAAGTTCATGATCCAACAACAACAACTTCTAATTCTAATCGCCGAAAAAGGGATGAATTTGAAAATACAACAATGAGGGGAAAATATAGAAATAATCCCAAAAGTGAGGGCACTTACTTGCGTAATCCCTAA

>CrNAC-44

ATGGAACACTACTTTTCTTGCCCGAATTTGCAAGAGTTCTCTTCAAACAATCCAATTCCTACTACTACTACGACTACTACTACTACTACTACTCATTCCGAAACCAATTACACCCATAATAATATTAATGATCATCAATACATATTAGGCCAACCTACATTAGCTTCTTCTGGTGGTGGTGGTGAGATCGGGGGTATCGCGGCACTTGATAATTACAGCTTATTCGGAGATCAACAAGGTCATGATCATGACATAATTAATAATATTAATATTGATCATTGCATGAGTGGCTTTTATGACCATGAAAATCTTTTTCTTGATCAATATTACGAACCAATACAAGCTGTACCTGTTTCTGATGTACCTGATGTTCATGTCCATGTCGTCAATAATAATAATATAAATAATGAACGGATGATGCTTCCGTCTGGTTTCCGATTTTGCCCTTACGATATTGAGCTCATCCGAGACTATTTGATGAAGAAGATTGCCCATCCCCAACTTAATTGGGATCATATTAAACAAGTCCAGCTTTATGACTGTGATCCGTCTCAACTTGCAGCTTGTTATCCAAATGATGAAGGTGAATGGTACTTTTTCACTGAAAGGGACAAAAGATATCCAAACGGCGAGAGACCAAATAGATCAACAAGAAGTGGATATTGGAAAGCAACTGGAGCTAGAAGAAAAATT

>CrNAC-29

ATGGCAATTGCAGCAAATATGAGTACTAGCAGCAACCAACAAGATGAAAACAACAACAGCAACAACAAAGATAATGATGAACATGATCAAGATATGGTTATGCCTGGTTTTCGTTTCCACCCTACTGAAGAAGAACTTATTGAATTCTACCTTCGCCGTAAGGTTGAGGGCAAACGCTTCAATGTCGAACTCATTACTTTCCTTGATCTTTATCGCTACGACCCCTGGGAGTTGCCCGCTTTGGCGGCGATTGGGGAGAAGGAATGGTATTTTTATGTGCCAAGAGATAGGAAGTATAGAAATGGAGATAGGCCGAATCGGGTAACAACTTCAGGGTATTGGAAAGCAACTGGAGCTGATAGAATGATTAGAACTGAGAACTTTAGATCAATTGGGTTGAAGAAAACACTTGTGTTTTACTCTGGGAAAGCCCCAAAAGGGATTAGAACTAGCTGGATTATGAATGAATATCGATTGCCTCATCATGAAACCGAGCGTCTACAAAAGGCAGAAATTTCACTTTGCAGAGTATACAAAAGAGCTGGAGTCGAAGACCATCCATCTCTCCCTCGTTTACTTCCAACAAGAACATCATCATCATCTTCAAAAGGAGGAACGCTACCCAATAATAAGAAAAACAACAACGAAACAACAACAACAACAACCACCTCATCATCATCTCCCAATCCCATTGAAAGTTTTCAAAATTTCGTAGGAAATCCAAATCCCCAGAAAATTATAGATGACAAAGTAAGCGAAACGAGTGGAAGCAGTACCACAGATGTCGGAACATCTCTAGGCCTCTCCCAACACATGAGCACCTATATTTCTTTAGCCCCGGCGGCCACCATTACGACCACCACCACATTAGGTCCAGATTGGGCCTGTGTTGGTGGTGGTGGTGGTCAGCCATCTTTCGTGGCGCCCAATTCAGTTGATGATCTTCATAGACTAGTTAATTATCAACAATATCATCATCATCATCATCCCTCACTGATGTCTAGTACTTTACAACCGCCCTTACCTCAGACGTCGTCGTTATCTAATACCTTAGCTCTTAACAATGTGCTTCCACCGGGTTCTCTTCAAGCAGCTTTTAATGATAGATTGTGGGATTGGAACGCGATGTCTGATCAAGCAAGTAAAGATTACAACAACGCGTTCAAATAA

>CrNAC-23

ATGGAGGGAAATGGTTTTCCACCAGGGTTCAGATTCCATCCAACAGACAGTGAATTACTTGAATATTATCTCAAGAGAAAAATCATGGGACTAAATTTTGATTTTCAACTCATTTCAGAGCTAGACTTATACAAATTTTCCCCTTGGGATCTTCCAGAAAAAAGCCATTTCCAAGGAACAAACCAGGAATGGTACTTCTTTTGTCCAAGAAACAGAAAATTTGCATCAGGAGGGAGAACGAACCGGTCGAATGAAATCGGATACTGGAAAGTGAGTGGAAGAGATAGAGTTATTTATCATGGAAACAGAGTTTTAGGGATGAAAAAGATTTTAGTTTTCTACATTGGAAGAACACCAATAGGGGAAAGAACTGATTGGATTGTTCATGAATATAAACTTCCAGATAATCAGGAAATTTTGCAGGAAAATAATTCATTTGTACTTTGTAGATTTTTTCATAAGGGTGGTTTAGGTAGAAGAACAGGGGAACAAGTTGTGTCCCTTTCTGAGGAAAATCAGACTGAAAATTTGAAGAAGAATGATCAATGTGGTCCTTCTGATTCAACCCTTTTGTTTGATGAAGAAAAGGGTCTCATTGATTCATACTTTGATTCAGAAAATTGGCAGCTGCAGACACAGAATTCAACAATAGTTCATGCCTATAATCCTCAAGAACGCGATAAGATTATTGGCAAAAGTGTAGAAAATTCAGTTGGCCTTCAAATCCCAAGAACTGAAGAAATTGCCCTGTTTTCTAATCCTAATAATACTGAAAATGTCTCATTTGATCCAGAAAATTTTGACCCTTTTTTTGAAGAAGATGGCAGTTGGTTAAATGATCTCTTTGATGTAATCCCAGAGGAATTTAATGGCCCTGGAGGAGTTTTTGATCTTGGAAATAATACAAATGCAGAAGCTACCCCTCCTTCCTCAGGTAACAAAAAAGAAGGAAAAGACAAAGAATCAGTCTGGGATTTTGTACCAGCACCTCCTGCATCAGCTGCTGAATTGCCGGCGATGAAAAATAGGGTAACAAATGAAGAAGAATCACTAAGAAACTCAAGCAAAATTCATAAGAAGATGAAAACTAAGAAGGCATCATCATCATTATCAGAGGCTAACAAGTTTGTATTTGTCTATGATCTTGCATTGTGGCTTGTTCTTCTTATTGTGGCTGCAAACATTGGCTTCTTTGCTTACAAGATTATATTGTCCTAG

>CrNAC-28

ATGGCAGCAGTTGAAGACATGAACGGTGGCGGCGTCATAGTTAGTGGTGGGAAAGATGAAGAAGATGATGTTCCACTGCCGGGGTTTAGGTTCCATCCAACTGATGAAGAACTTGTAGGGTTCTATTTAAGAAGGAAAGTGGAGAAAAGGCCCATCAGTATTGAGCTTATCAAGCAGATCGATATCTACAAACATGATCCTTGGAATCTACCAAAAGCAAGTAATGTAGGAGACAAGGAATGGTACTTCTTTTGTAAAAGAGGAAGAAAATATAGGAATAGTATAAGACCAAATAGAGTAACAGGATCAGGATTTTGGAAAGCTACTGGCATTGATAGACCAATTTATTCCGCCGGTGGAGAAGGTCGAGATTGCATTGGCCTCAAGAAATCATTAGTTTACTATCGTGGAAGTGCTGGTAAAGGTACCAAAACTGATTGGATGATGCATGAATTTCGCCTTCCTGCTGATCATGACTTGAAAACCACCAAACATATTGATGCCAAAACCATTGCTCAAGAAGCTGAAGTTTGGACATTATGCAGGATATTCAAGAGGAATGTATCATACAGAAAGTGCATGCCGGAATGGAAGGATCATCAACCATCTTCTGCCAAGAAATTAAACCCCAATAATAATAATAATAATATAAATACTGATGCAAGCTCCAAAGCATGCAGTTTTGAGTCTAATAATGATCATGATATTAGACAAACTTACATCAGCTTTAGTACTTCTTCTCCAAAGACCTAG

>CrNAC-30

ATGGATGATTCTTGTGCTGCTGATCTTCAATTGCCCGGATTCAGATTCCATCCCACCGAAGAGGAGTTGCTTCAATTTTACCTCAAGAACATGTTACAAGGCAAGAAATTGCATTTTGATATTATTGGTTTTCTCAACATTTACCATCATGATCCTTGGGAATTGCCAGGGCTGGCAAAGATTGGGGAGAGAGAATGGTATTTTTTTGTGCCAAGAGACAGAAAACAGGGAAGTGGAGGAAGACCAAATAGAACAACAAAAGCTGGATTTTGGAAGGCTACTGGTTCCGATCGTAGAATACTTTGTATATCTAATCCCAAGAACATGATTGGACTCAAGAAAACACTTGTTTTCTACAAGGGCAGAGCACCCCGTGGCTGTAAGACTGATTGGATCATGAATGAATATCGCCTACCTGATACATCCCCAGTCTCCCAGACAGATTTAAAACAAGATTTTTATGTTAAATGTAGGACCAAAACACAACTTAAATACACCTATAATAATAGTTAG

>CrNAC-16

ATGGATGAGAAAAATGATGGAGATAAGATTGATGATGTTATGATGCCTGGGTTTCGATTTCATCCAACAGACGAGGAACTCGTGGGATTTTACCTAAAGAGAAAGATTCAGCAACGACCACTTCCAATTGAATTGATTAAGCAAGTCGATATTTACAAATATGATCCATGGGACCTTCCAAAGGTGGCATCGACAGGGGAGAAAGAGTGGTATTTCTACTGTCCAAGAGATCGTAAATACAGGAATAGTGCACGTCCGAATCGAGTTACAGGAGCTGGATTTTGGAAGGCTACTGGAACAGACAGGCCAATTTATTCATCAGATGGTACTAAATGCATAGGATTGAAGAAATCGCTGGTTTTTTACAGAGGCAGAGCTGCTAAAGGAATTAAAACTGATTGGATGATGCATGAGTTTCGTCTCCCTACTTCTGATTCTCCACCACCTAAGAAACTCTTGGACAAGGGCCTTCCTCCCCATGATGCATGGGCAATTTGCCGGATTTTCAAGAAAACAAATTCAATGGCACAGAGAGCACTTTCTCATGCCTGGATATCATCATCTCCCTTTTCCGAACCATCTTTACCCGAAATATTCCCACAATATTCTCAATTCAGTTCAGAAAACATATCATGTACTACAGAAACAGGATCTAACTTGCATCTCTGCAGCAACAAAAATGACTTAAATCCAACTTCTACCCCCACATTCTCACCTCTGAATTCCATGGTTTCAAAACCATCATTCTGCTGGGCAAATCCAAATGGGGATTTTCCTCCTAATTTCATGTTTTCCCCTCCTACATTCGATGTTTCTTCAATGATTTTCAATCCATCCCCTGTCTTAATCCCAGGAGGAGATGGAAATAATAATAATAATAATAAGGTCCCAGAAAACATTGAATTCGAAAGCTCAAATCAACCACAACATCATCATTTCAACAGTTTTTCATCGCCGGAAATTCAAGAAAGGACAATTGAATTTCCATTCAATTTGCCAACTACAAGTATAGGTGGCGGTGGTGATGATTGGAAGTCTCATTTATCGTGGGATTCTCCGGGAAATTGCAACAGTGAAATTTCTACTACTTATTCTTCCCCAAACAAGTGTTACACATGA

>CrNAC-18

ATGGATCAAGAGAATAGAAATGAGAAAATGGAAGAAGTGATGTTGCCAGGGTTTAGGTTTCATCCAACAGATGAAGAATTGGTAGGGTTTTATTTGAGGAGAAAGATTCAGCATAGACCTCTTTCCATTGAACTTATTAAGCAACTTGATATCTACAAATATGATCCATGGGATCTTCCAAAGTTGGCAACAACTGGGGAGAAAGAATGGTACTTTTACTGTCCAAGGGACAGAAAATATAGAAATAGTGCAAGGCCAAATAGAGTAACAGGAGCTGGATTTTGGAAGGCCACTGGCACAGATAGACCTATTTATTCATCTGAAAATTCCAAATGCATTGGCTTGAAGAAATCCCTTGTTTTCTACAAAGGTAGAGCTGCAAAAGGGATTAAAACTGACTGGATGATGCATGAGTTTCGGTTACCTTCAATCAATGATTCTGGACCTGCCAAACGATACTTGGACAAAGACATCCCTCCAAATGATGGTTGGGCTATTTGTAGGATATTCAAGAAAGCTAATTCAACAACACAAAGAGCTCTTTCTCATTCTTGGGTATCTCCAACAGTACCTCATGAAAATGACCAAATATTATTAGGCCAACATATTATTATTGATGGCACTAATAATAATAATAATAATAACATTTTTAATTCAGACAACATTTCTCATGTGACAACCAAAAATAGTTCATCAGTAATCCAATTTGGAGATAGCAGCAGTAATAATGAAGTACAACAATCAATATCTAGTTTTTCTCCATTATTAGATCCTATAAATAATTTCCCTTATAAATCTATAATTACTCATGATCATCACCCAATATTATCATTTCCTAGTAGCATTGAAAATCATGATGAACCACCCTCATCAAAATGTACCACCAATAATATTATTAATGATGCTTCTTCCCTTCTCCTAAACATGTCATCCTCTTTCTTTGGTGATTTTGGAAGATCAAATTACAAAGATTTTGCAGAATTCGGCCCCGTTTCATCATCGATATCATCTCAAGATCAACAGTGTAACAATTTCTTGATGACATTACCTCAAGATATGATCATGAGAAGATCAAACTTTGATGATCAAGAAGATCAGACGGCTCTGATGAGTGCATCTCATCAAAAGGATGATCATCAATGGGGAGTTCCCATAAATTTGCCAATATTGAGTATGGGTGATCAAGCATGGAAATCAAATTTGATATGGGATTCTTCTTCACCTTGTCCTAGTAGTGATATTTCCACCACTTATTCTACTAACAAGTGCTATTCTTGA

>CrNAC-21

ATGGAGAAGCAATTAAACTTTGTCAAGAATGGTGTCCTCAGATTACCACCTGGATTTCGATTTCATCCAACTGATGAAGAACTTGTTGTTCAATACTTAAGACGTAAGGTCTTTTCTTGTCCTCTGCCAGCTTCGATTATCCAAGAAGTTGATGTTTGTAAATCTGATCCTTGGGATTTGCCAGGTGATTTGGAGCAAGAGAGGTACTTTTTCAGTACTAGGGAGGTAAAGTACCCAAATGGAAACAGATCAAACAGGGCAACTGGTTCTGGCTACTGGAAGGCAACTGGTGTAGACAAACAAATTGTAACATCTAAGAGCCACCAAGTAGTTGGCATGAAAAAAACCCTTGTTTTTTACAGAGGAAAGCCACCAAATGGGACCAGAACTGATTGGATTATGCACGAGTATCGCCTTGTTAATGTCCAAGAAACCGTTGCTGCTGCTGCCTGCCCCCATCAGCACTTAGCTCAGGATAATTGGGTTCTGTGCCGCATATTTTTAAAGAGGAGAAGTGGGAAAAAGGATGAAGAAATGGTGAAGTCCCAAAACTGCAAAACAAAATCAGGGGAAATGCAGGGGAAAAAATCTGGGGTTGTTTTCTATGATTTCATGGCTAAGGAAAGAACTGATTTAAATCTAGCACCAGTTTCATCATCTTCGGGTTCCAGTGGAATTACAGAGGTTACTAGCAATAATGATTCAGATGATCATGAAGAAAGTAGCAGTTGCAGTAGTTTTAGTGCTTCAGTTAGAAGAAAAACTTGGCTTTAA

>CrNAC-42

ATGGATAGTATAATAAGAGTTCCAATTGGGTTCAGATTTCGTCCTACAGATGAAGAGTTGATCATTCACTACTTGAAAAGAAAGGTTTTGTCTCTGCCTTTTCCTGCTTCCATTATACCTGAATTCCATGTTTTCCAAACCAATCCTCTTCACTTTCCAGGTGACCCGAGGGAGAATAGGTATTTCTTCTGCAACAGAAAGGTTTTGCCCATATCTACTATTGTCAGTGTCAGGGATGGTTCTGGGTACTGGAAACCCACCGGCCGCCAACGGAAAATTATATCCCCCGCCGCTAACAACCGAGTAGTGGGTACAAAAAGATCCCTGGCGTTCTACCATTATCAGGGGAAACAAAAACATGGCCATGGTTTAATGACTGATTGGGTTATGGATGAATACTGTCTATTGGCCCCAGATGAACAGCAGCAGAATGTTCTGCAAATTGGAGACTGGTTTGTGTACTGCATACGCAGGAAGAAGAGGAAAACCAAGAATCAATATTCTTCAAGAAAGGATGAATCTTCTTCATTATGTTTAAGTGAAATAATTGATGTTTCTTGCAATAATGAATTAGATCAGGAAGCTAACAGTGAGTTTTAA

>CrNAC-43

ATGGTTGCAGAGGAGAAGGGCAAAAAGAGAAATTATTGTGGAGGAGAATGGAAACAACATAGGATGAAAATACCAATGGGTTACAGGTTTATACCAACTGATACTGAATTAATATTGGATTATTTGCTTCAGAAAATTATTGGTAAACCACTTCCTGCTGACATAATTCCACAGATAGATGATCTCTATAGAATAGATCCACAACAACTCCCACTAGGCAAATATCAGTACTGGAAGGAGAATGAAGCATACTTTTTTACCCACCAACATCAAACCTATTTGCCAGGAGATAGAATGCCAAATGGTTTCTGGGAGGCTGATAAGAAAGATGAATACATTCTTGATGAACACAACCTGCAGATTGTTGGCTTTAAAAGTACGTTTACGTTTTATCGCGTAATAGAAAGAAAAGAAGAGGAGACCGATTGGGTAATGACTGAATTCAAAGTTCATTCAACCCTAATCCCTTATGATGCAGCAGATGAGAATCTTATGCTATGCAAAATTGAATATAACCCTCAAACAGAAGAATGA

>CrNAC-40

ATGGGAGACGACAATGTGAAGCTGCCACCCGGATTTCGATTCTATCCTACCGATGAAGAACTAGTCGTCCATTTCCTCCACCGTAAAGCGGCCCTCTTACCTTGCCATCCTGACGTCATCCCCGACCTTGATCTCTATCCTTATGATCCTTGGGACTTGGATGGTAAAGCAATGGTGGAAGGGAATAAATGGTACTTTTATAGTAGGAGGACGCAAAGCAGAATCACGGGTAACGGATATTGGCAACCGTTGGGAGTGGAAGAACCAATATTCTCCATGACTTCCGGCCAAAAAGTTGGCATGAAGAAATTTTACGTGTTTTACATTGGTGAACCACAAGAAGGTGCCAAAACCAACTGGATAATGCAAGAATATAGACTTAGCTCCGACAGCGGTTCCACCAGTAGCAGTAGTAGTAGTAGTAGATCCTCCAAGAAAAGACATTCCAAATTGGATTATAGCAAATGGGTAGTATGTCGAGTGTACGAACGCAACAATGACGATGATGACGACGATGGCACAGAGCTTTCATGCTTGGATGAAGTTTTCTTATCTTTGGATGATCTGGAGGAGATTAGCCTGCCAAACTAG

>CrNAC-45

ATGTTGCCGGTGGGCTATAGATTTGCACCCACTGATGAAGAATTGATAAAGTATTATCTGGCCAATAAAGTCTTTTACAAACCTGTTCCAGTTAAAATCATCCGAGAAATTGATGCTACTTTTCTTTATATGGGAGATCCATATATTGAAAAAGAATGGTTTTTCTTTGTATATAAAGATGAATATTTTCGAGGGAAAATTATGAGGAATCGTCGGGTTGAAGATGGGGAAGGTTTCTGGCAATGCATTGGAGGGGAAGAGCCAATTTGCAACTCTAATGGACAAGTATTGGCTTACAAGATTCATTTGACCTATTTTTCAGGACCAATAACTAATGGCAAGAAAACAAATTGGAGAATGGAAGAATATAGGTTACTATTTGAATGTAATACAACAAATACACAAGAATCCTCAGAGAATGGATACTGGGGAGAATTGTAA
