# Supplementary material for: Genome-wide identification of NAC transcription factors and regulation of monoterpenoid indole alkaloid biosynthesis in Catharanthus roseus
Source: Front Plant Sci. 2023 Dec 20;14:1286584. doi: 10.3389/fpls.2023.1286584 (PMC10785006; doi:10.3389/fpls.2023.1286584)
Supplement: Supplementary file 3 [file DataSheet_3.docx]

>CrNAC-37

ATTAAAAGCCAGAGAAAACAATATCCACACAAATCAATTCGCATATATAGATTTTATGGATATGCAGCAGCAGGAAGATCAAAAGCAAAAAGAAATAAAATTAGTAGAATTCCAACCTGGGTTTCGATTTTATCCAACTGAAGAAGAGTTGGTTTCTTTTTATCTACGGAAGAAGCTGGAAGAAAATATCAGAGAGCCCAACCTCCTCGATCGTGTTATTCCAGTTATCCACATTTACGAATTCGAGCCATGGCTGCTTCCAAGTTCGTCCAATCTTTATCTTTCTTTTTCTTTTTTTTTTTCCTTTAATTGGCATATAGTTCATATATATTTGTACTTTTTAAAATGAGCTTTTTTTTTATAATATATGTACTCTTATCAAAAGTAATTCATTTTAGTTGTTTATTTTACAAAAAATGTCATTTCATTTTATACCTCTTACATTTTATTATTATTTGTATTTTTTATTTGTCACATTTTAAAAATAATGTGATATTTAATGATTATTTTTCATTATATTCTTTCATTAAAAAAATCAAATTTCATAAGATCATAAATTGTTTTGCACACAAACTTATAGAAATAATAATAGTAATAATATTAAGATTGATTTGGAGATATTTTAATTCCTGAAAATATATTTAAAATTCTGCAGTTTATTAAAGAATTGACTAAAATCTTTTGAGTAAAAAAATGAAAGGACAAATGAAATGTGATATATATATATATATTTTTTACTTGTCATATTGTGAAAATCATGTAACATTTAATGAGTTTTTTCTCATTATACCTTTTCATTACAAACAAATTTCAATATATTTTTCAACTCTTCTCACATAGAAATTTGTGTAAATAATAATGAGGAAACAGAAAGTTTGAAGAGCATTATAGTCTAAAAAAAATATTTAAAATTTTGTATTTTCTTAAATAGTATACTAAAAATGTGATAAATAAAAAAAAATAGATGGAATATTAATGAAGGGCATATTACTATATGCAAAAGAATCACAAAAATTGTTGTGTTTTCTTAAAATTTGTAGTTTGGATTTTCTTTTAAATTTTTTTATTTTTATGGGTGCAGAGCTATCAGGAGAATTGTGCAGTGGAGATAGTGAGCAGTGGTTTTTCTTTGTACCAAGACAAGAAAGAGAAGCACGCGGCGGACGTCCTAACCGGACCACAGCCTCCGGTTACTGGAAGGCAACAGGTTCCCCCAACTACGTTTACTCTTCTAACAATAGAGTCATTGGTGTGAAGAAAAGCATGGTTTTTTATAGAGGGAAAGCTCCTACCGGAAAAAAGACCACTTGGAAAATGAATGAGTATAGAGCCATTGAGGAACAAATTTTGCCTTCCTCATCATCTGCTCAACCTATTCCCAAGGTGCACGAACTTTTTTATTCTTTTTAATTATACTTATGAGAAGTCCTGCTCATATCTAAGATTGGTATACAATCTTGACTAATTCAAACTTAACTTATGAAGCATTATAAAAAATTTAAGTTTTATTCGATAGTTAGAGTTAGGATCTGCTCTCACATGGGATAAATGCTTAATCCTTTCATCTATTATTAAGACTACATAACGTTTTTGTAAATTATATCCAGTACCAAATATATAACCAGTGATTTCAATTCCAAAGGTTAATCATACTTAATTTTATGTAAGACAAAGTTTTATTTTTTGGATATCTAGTGAAAAAAGTTAAAAGATAAGCCACCTTTATTACCACTCTACAAAACTATACATAAGATTTTTACTTTATATAATTCCTTTAAATAATTCCTCCTTCACTTCTTTCGATTTCATTGAATCTTTTTCTGGGTTCGAGAATCACCTCTCTCCTTTCGCTTCTCTCTATACTTGAACGTTTCATGGCACATAAATTGATGGGATGAATTCACTTAAGAATTTCTGACAATATATGTACAATATATAAATAAAAAATGATTATAAATGTCATGTAATTTATTTACTTTTTAAAAATATATATAATTAATCAATCTTTTTCTCCTATTCGTTGTGCTAATTTAAATGAGTGGTTTTGTTTTCTCTTTTTGGGGTAAATGCAGTTAAGGCATGAATTATCATTATGTAGAGTTTACATAATATCAGGGAGTTGTCGAGCATTCGATCGAAGGCCATTATGGACAGAGACAAGGAGGCTGGGGGAGATAGCTCAAGACTTCAGCAGCTAAAAAAAAACTTATTATATGCATGGGA

>CrNAC-41

ATGGGAGATGATGAAATATATCTTCAACCAGGATTTAGGTTTTATCCAACAGAAGAAGAATTGGTTTCTTTTTATCTCAAAAACAAGCTTGAAGGATTAAGAGTTGAAGAAATTAATATGGTTATTCCAGTTCTTGATATTTATCACTACAATCCATGGGAGCTTCCGAGTAAGTAATTCAAATTATTATCCCTTTTAATTATTTGATTCTGCTTTTATATATATTAATTCATGGCCGAGATTATATTATTCTCAAAGATTTCATATACAGATCATTTAGGTGGTGCAGATTGATGAATTAATTAATTAAGCACCATTTTATGAATTCAAACGCCATAGATGATTGAGTAATACTATAATCTTAAATGATTTTTCTTCCTTCACGTTCATCTATTATTCATTGCTTAAAATAAATAATTTTAAGCTTATAAATTGCTGTTTTTGAGCCGTTATCTATTATATACATTATAAATTTACTTTTAAAAAATTTCATTTTTGAAAAGCTATTCTAAAGGTGTTTTTTAAAACTTCTTCTACTACTCCTGATACTATTAAAATAAAAATAAAAAAAAAAATATTAACTTGAGAAAAAATAGGCTTCATATTTTCTACCCATTAAATTCATGATGCTGATATAAATGCTTCGAATTTGATTAAAAAAGAACACCAAGGTTTTTTTTTTTGCATGGAGAGACTCAAGGTTTTTATTTATAATTTTCCATGGGGGGAAACTTGGCAGAATATGCAGGAAAATATAGTAGGAAAGATCCAGAAGAATGGTTTTATTTCATACCAATGCAAGAGAAGGAATCACGTGGAGGAAGACCAAATAGGCAAACAAATGAAGGGTATTGGAAAGCCACTGGTTCTCCTGGTTATGTTTATTCTTTAAAAAATAACAAAATTATTGGAGGCAAAAGAACTATGGTTTTTTACAATGGAAGAGCTCCTTATGGCAAAAAGACACCATGGAAGATGAATGAGTATAAATCTATTGATTATACAGCTCCTCAACTACCCCCAAAGGTATTTCATTCCCCATTCACCTTCTTTAATGAAACACTTTCTTTACCATACTTTCTTCTATAGTATTTTTATTAACAATAATTCTTATTGGATAAGATTTATACAATTGATTTGATTAAATGATAGTAAAATTATCAACACAAGAAAAAAAAATTTTTTTTTTGATGCCATATTTGTATAAATATTTCCACCAATAAATGATAATTCCACTCTTATTTGATCTAATTAATTAGATCAGTATTCATCCCTATAACATCATTCTTAATTTTATTATAACCCTATAAAATATACTTTTCCAATTATAAATATAAGGAGTTTTAGACTTTTACAGGAGGATTAAAGGTCTAAAAAACAAGACAATTTTTCAACTGCAATGCCCTTCATAAATAGAGAAAGAGATAAACAATGTGAGTAAAATAAATTAATAGAAAGATGGATAAAGATTTAAAAAAAAAAATTCAATAAATACTTATTAAAAATTGTAAAAACTTTATATTTTTAACAATTCTCAATAAATCTAAAACCCTTTATATATTTGACCGGAAGGAATAAAAGATAAATTACAATAAAAAAAAAAAGTAAATTATCAACAGAATGTCACACATGGATGCCATCCTCTTCATACGTGCCAAGATTATTAGTTTTTATGGGTTAACATTTCTTGTAATAACTTTCTTAATTTAATATATTTTATTAATAAAATTTATTAAATAAAAAATATATTTTTAAAAAATATATATTATAAAAAGAATATTACAAAAAATATTATATAAAATGTTGCAAATGATCTTAAGTGATATTTTATGTGAGTATTTTTGTTGACCATTCCCAATAAGTAGTGTCAAAGTTCGCTGTGAACCTAACATTTCTTGATATCGTGTTTTTTTCGTCAAATCATCTAAGGCCTCTTTTGATAAAACTGAATACTAAAGTTTGAAGTTTGAATTATTTCAGTTAAATTAATTAATCAGCATTAATTATATATATAAAGTGTTATAATTTTACAAGTAATTATATATTTTTAGAATTAAGAGTTCAGAGTTAGATTAAAAATTTTCACTTAAATTTATAACTATAATTAAACACATCTTAAACAATTAAGTGATTTACAAAAATTGTTATTAAACGCACTTAAAAATGTTAAGTTCTTAAAAATTATATGTTATCAAACAGACTTAATTTAATAAATTTTTAATTGATTCATTTTAAAAGAAAATTTAATTTATCAAACAAGTCTTAATTAATTTGATGATAATATATATATATATATATATATATTCTGATGTAATTAATACTGATTGTAGTTGAGGCAAGAAATGAGCATATGCAGAGTTTACGTAAAATCAAAATCTGTAAGAGCATTTGACAGGCGACCACCACCGCCACCATCATCCATGCCAATTCATGATCCTCCATTAATACTTCCATCAGCAGCAGCAGCAGCAGCAGCTGGCTCTCAACTTAGTTCATCCCCCGGAGATGACAGCGCTAATAATGTGGAAAATAATTACGGAATGATCAACTGCTGGAGTTTCGCAGATGATGATGATGATAATACCCAACCTATGTGGGATTGGAAATTACTCAACTGA

>CrNAC-19

ATGGAGCAAGAGGAGAAGAAGGAGGTTTTCGTTTTGTCAAGGAAGAATATGGAAATTTCAATTGCAGAAGCATCATCAATGTTTCCTGGGTTCCGTTTTTCGCCCACCGACGAAGAGTTAATTTGCTATTATCTGAAGAAGAAGCTTGAAGGGTCAGATAAATGTGTTGAAGTTATTCCAGAAATTGATATTTGTAGGCATGAGCCTTGGGATTTACCAGGTACCCCCTTCTCCTCTTTTTCTTCACAGAAATTTAATCAAATTTCGATATGTTTCACTGAAGGGGGAAAAGGGAGAGTCTTTGTTTAAATTGGAGTGCAATTGGTTAAAGGGATGTTCAATAAAACCTTAAAAGACCTATTGGGAATCAGTTCAGAGGATGGTTTGGTTATTCGAATGGGTCAGGACATATGCTTTTATACTTGCTTATTCCTTTTAGCTAATTGAGATTCACTGATAGATGGTAGAACAAACTGATTGCACATACTTGCGTTGTACACAAAATGATAGCAGTTCCTAATTTCCTGTTCCTCCATTTTCACGGCCATCGCTATTTTAGGACACAAAGTAGTAACTTTTTGTTTTCGTTGTGGATGATTGGTCTTCTGATATGAGGGCCTTTATCCTTCCAAGGATCGGTATAAGAGATTAGTGCAAACTGGAAACACAGTTTCAGCCTTCAGGGCTTATTTTCAACTAACCTAATACTTTACTCGTGACTTGTAGAAATGGTCCATTTTATGAATTTGCTTATTGGAGGTTGGATGCCCGGGTTACATCTTTGCAATTAATTATCCTGTTTAAGTTTGATAAATGGATCTTAAATCAAATTCTTGAGGGGAAAAAGGCATATGCTTGTGCAATGAGGTTCTTCCTTGAGACCCTGGACCTATGTCCCCTAGCAATAAGTTTTGATCATACAATGTTTTGGATTCCCATCAGGCGTTGTTCATATTTACACTGCATAGATTGTACTCAAGTTGCTTGGGTTGAATGTTACATTGGAAAAGTCTAGGACGAGGTGATTTTTTCTATTAGACTCTTCAATATTGCCTCATTATGACAATTTGTTCTCAAAGGAGTCTGCTTCTGAGGTACTGCCCTACATATACATTTGTCTCGCGTCTACTGTAGCGACTGATTGATTCTATGGTGTAATCAAGATTATAAAATTTTCATCACATCCTACTGGAGATAACTGTAATATTATACATGTAGGGTGGGGACTTTGATGGACTAATCAGCACAAATCTGAGCTGCTTAGGATGAGAGTTTCATAACAGTTTTAAACATATTAATTTTTTGGTGATACAAGTAAGGATAGTTTCCAACCTGTATAAAACATAATTTCTTGGCTAGCAATATGGTATTGCTTTTGTTCACTGCAGATGAACCTTCTGAGAGTAATTGTAAGGTCTTCTCCTCAGAGAATATATATGTTTCTAAACCTGTATAAAACATAGTTTCTTGGCTAGCAACCTGGTATTGCTTTTGTTCACTGCAGATGAACCTTCTGAGAGTAATTGTAAGGTCTTCTCCTCAGAGAACATATATGTTTCTAAATCATTTAGTTTACTCGGTATTGTCCGCAAAGAAAAAAAGAGAACATACTTTCTTGGCTAGCAATCTGGTGTTCTCCCCCTTTCGAAGAGAGCACCTTTGCTACATCATTTTCTTCCAGATGTTACTCCTGGCGAAAAGTAAACATAAACATGGGGTGTTGAAGTGGATTTTATCTTAAGCAGTGATACATTGTTCATGATTTTCCACCAATGTTAATACTTTTACTTTCTCACAGGTTTTCTTTAATTTTACCCTGTTCCAGTCTCTGATTGTGGCATATAATGTTACATGCAAACAATTGCTTTCAACAGTTTTAAAATGAAAAATGCTTGAAAAATGAAAACGTGCATATATTCCAGAAACTCCTGATTGTTTTGGTGCCTCTGGAATGCAAGCTGTAATGTTCCTTGGAGATGTTTACATCTGTTTTAGACTATCATGAGAATGGTCCGGAAACTTTTGGAGCACTTTTTACTTTGGCCCTTGAAGAAGTTGATCGAAGTTCATCTCGAAATTTGTGCTATTAATGCTTGCATTAACATGAGTATTAAGGAATTTAGAGACACATGACTTTGGTTTACTTTTCCCTGTTTCTCCTTGACATAGGTGGTTTGGACATTAACCAAGTTTTACTGACTTGTGTTGATTCTGTCATTCTAAACTATTTTCCCTTTCCTGCCACCAGCCAAATCAATCATTCAATCAGATAATGAGTGGTTTTTCTTTTCTCCTCGCGGGAGGAAATACCCCAATGGCTCCCAGAGTAAGAGGGCAACTGCCTGTGGCTACTGGAAAGCCACTGGGAAAGAACGAAATGTGAAGTCAGGTTCAGCTGTTATTGGCACAAAGAGGACTTTGGTTTTCCATACTGGTCGGGCACCAAAGGGACAGCGGACAGAGTGGATAATGCATGAATATTGCATGAGCGGAAAAGCACAGGTAACTGTGTTATATACTATGCAGTAGATTAAGTAAATATGTGCTTGTTCCATCATATTTCTTTTCACAGATTATGCAGGAATTCGCGTAAGACTGCTTACCAGAACTTAATTCCAATATTTCTTTCAGGAGGATACATAGTTTTCTACATTATCTTTTACCTTAATTGAACCAAGTAATATTTCAGTCCTTTGCTTTGTCTGGAACTAGCTGTTCTGTCCTTGAACTAACAGATGGGTTACAGTATATTTCAGTGGAATAATTTCTTCATACTGTAAGTTGTCCAGAAATTTTCATTCTTAGAATAGTTTCTCTTACTATAATATTGCCATTGTTGCTCTGTAAAGATGACCATAATGACACTACTTAGAGCAATTTGACTCAAATTCTGTGCTCAATGCCGTAGCAGGTATTATTTGGAGAAATATTTTAGTTGAACTCCCATTATTATAGGATGAGTACTTTGCTCCAATGAATAAGAAAATCTAGGAAATCTTCAGATGAATTTTGTCCATTACGGAGTTGAATATTTCAGTCTGATGCGTAAAGAAGTTATCATTGTCAATGGGATAATAGGGAAACTAGCACATCTTTTTGGTGTCACCTTGGCTTCGATTAGTTTTTCATTATTTCTTCTGATTGTCAAGGGTGCAGCTTTATGCGTCTAGTTTAGAGTACAATCCAAGAAACATTAAGAGATAAAACATGAAATATATTCTGCTTTTACAAGATATTCTTCATATGCCTTGCACGAATAGAGAATGTGAGATATTAATTTTGCCTAAGTTTTCTTTTAATTATTTAAAACAGAAATACTGAAAAAGTATTTGATAGAGTGAATATAATGCATGCTAGACTTTTAGCAGCTCAAGCTCATTTAAAATTGAACCGTTGGTTCTATTGTCATAACTCACTGGGAACCTAGCAGCTCGAGCTCTTTGTTCGACTTCTCAATTCTCTCTTATAGCTTTGCTACTTTCAAACGAACTCAAGACTTCAGCTTGTTGCTGCATTTGTGGATTTCTTATAGGATTCCAATTTCATCCAGAATTGAAGTCGCTGACAGCAGATGATATTTAGCTGACGCGGTCATATGCTGCTATTTGCTAGATGTAGGCCCTCTTCTAAAAGATTCTTTAAGAATCTAGTGTTGATTTTTAGAATCTCTTGGTTGAAAATCACGTCATGAATCTTCATCTTCTCTTTCTATGAGTTCTTTCGCTTTTGCTAAATGCAGGATAATATACGAAACTAGTAAAAAACTTGAACCTTGAATGATTCGTTCGGTTAGTTAGCTGCATTGGACCTAAAATTTTTTTATCACTTGGAATATCTGTAGGATCTCATGGTAGTTTGCCGTCTTCGGAAGAACATTGAGTTCAATTTAGATGAGAATCCACGTAAGGGATCAGCAGGACAAAGACATTCATCCACTCTAGCCAACGATGTTGCAGCTTTATCTGTTGTTGAGCAGAATGGGGGAAAAGCTGGCACCCTGGTAGCTGACTCTTGCTCAAAGGAAGGTAGCAGCAGCTATAATTCCCATTCAGTTGAGCAGAATGACTCTTGGTTTGATTCCGTAGATAAAACAACTAATGAAATCTCTCCACATGGCTCTTCCAGCTGCCGGCAATTCCAGGTTTTAAGTTTCTCTCTTCAACTGTCTTGTTTGCTTCTGATTGCATGCTTAATGCATTTCCAAGAAGAAAAACCTTGTATGGTTGAATTCTAGATAATAATCGTAAAAGATTTTTTCCTTCCCCAGTTATATTAATCAATAGAGAATGCGAGATTTACACGAGAGCACCCTAGTTAGTATATCCATGTCAAGGCTTCTGATCTATTTTCGAAATGCAGGGCAGTGACAGAAATGAAGAGGATTGGTTTGCAGATATAATGAACGACGATATCATCAAGCTCGATGAATCTTCGCTGACTGCGAATCCTCATCTACTGCCAACAGCTCCTGGAGAACCTCAACCCAAGATCACTTCTACAGAACCAAGCCGAGTTATAGCACCTGCTGCCCTTCCTTTCCAGGGCACAGCAAATCGACGACTCAGACTCAGGAGGCCGAAAATCGAACTGAAGAATGCAACACCGTTAACAGCATATGAATCAGGCGTTGTCACCTTAACCAGAGAGCTTGTCCATTCCCAAGATTCACAAAAGTCGCAGAAAAACTTGGGCATGGCCATTAAGCATCGGGTAGTTTTAGTGCTATTTGTGATCATACTTGTGTTACTTTTGTACATGAAGTTGTGATAGCCCTTCTAAAGTTCTTTCCCAAGTCATCTTGCTCCAACTAGCGCTTAAAAGAGAGTTTGCAACAAACATGGTTTGTACATATATGCAAGTTACTCTTTAAGATATCATGAATTGTACAGCACAAATACGTAGTAATTTCACACTTGTGAATGGGAATAAATCTAATTACAGCAGAAATTACTTCAAA

>CrNAC-04

GGTCCAAAACTTCTCCAGAAGCCTTTTCTTGCTGAAATTTTGTTGTCTTTTTGAAAAATTTTGAATATATTGTGTAATTTGAAGAGAAGAACACAAAAATGGGAGGAGCATCTCTGCCTCCGGGATTTCGTTTTCATCCAACTGATGAAGAACTTGTTGGATACTATCTTAAAAGAAAAACTGATGGACTTGAAATTGAGCTTGAAGTAATCCCAGTAATTGACTTGTACAAATTTGATCCATGGGAACTCCCAGGTATTTTAATAATATTCATCTTTACAGACAGAAATTCTACACTTGCTCAAATTTTCCTGAATTTATAAGTTTTTAGAAACTGTTGGAAACATTAGCCTTTGCTTTCCATGATGATCAAGAATTCAGTTTTTTTTTTTTTTTAATAAACTCAATTATGAACTTGAACATCATTCCAATTTGTGGGGGGGCTATATTTTTGGAAAGTTTGATGCTTTATTGCTCATCTGTTCATTCCTTTTCTTCTTTATTCTGCTTAGGAAAAGTGCAAGTCATTGTGGGACATCTGACTTTTATACTCTTCTAATCCATTTTGTTTTCACACATCAAAACTTTCTTGTTTGCATTGGAAATTTGAAGTCTCATAGGATGTTTATGAAATGCAGAAAAGTCATTCTTACCAAAGCGAGACAAGGAATGGTTCTTTTTCTGTCCTCGTGATAAAAAGTACCCTAATGGTTCCCGGACTAATAGAGCAACTAGATCCGGATACTGGAAAGCCACAGGGAAGGACCGGAAAGTAGTCTGCCAATCTGCAGTGATTGGTTTTCGCAAGACCCTTGTGTTCTACCGTGGAAGAGCTCCACTTGGGGATAGAACAGATTGGGTAATGCATGAATATCGTCTTTGTGATGATGTCTCTCAAGGCATCCCAGTTTTTCAGGTAACTAGCAAACTCATTTCTGCAGTCTGCATAGATTGAACCGATATGTAAAGAAATGAACGCTTATAAAGGACGTTTACCAAATGGTTCCTTCTTCATGCATTAGCTAGGCCTTTGATCCTTCAAATGGTGGAAGATACGTTAACAACAGATTATTTGTGTGTTTTTGCAGGGACCTTTTGCCTTGTGCCGTGTCATCAAGAAGAATGAGCAGAAGATGAAGGATGTTCAAGGAGAAGCAACATTTAAGCCAGTTGGAAGCAGTTCACAGACTGCAAATGTGGCCTCAGTTGAACCAATTGTCATCACTGATGATAATCCAATTCAAGCAAGTTATATTTGCAATGAAAGTAATCATTCAACTCCTATGACCTCTCCATATCAGCCAACAACAATGGGAGACTATGATCAGCCTTCAATGGGGACTAATAATCCTCCAAGCCTTTTTGTCTCACCTGATATGATTCTTGATTCATCAAAGGTATTCAAATGTGATTTTCTTTGTTGATAACTTCTTGATTTGTTCTTTTTTGTTTTCTCCGAAACTAAAACATATAATGCCCTTAACAAGTGCAGGATTACGCCCAACCACGCGGTTTACATCCAGAATATGGATTTCAAAACTCAATGTGGCAGTCTTCATATGATCAATTCGAAATCTCACCAACTTCATCAAACTCAAATCTTCAAGAAGAAGGGGAACCTTCTGATGATTTTAGTCGATATGGATGCATGTCGCCCTATTCGGTTTATGGAAGCTATATGGGGTACTATGGAAATGACATGTTATATGAAGGTTATGATCAAACAAATTCACTAAGAAATCAACAACCATTTTGAAGAACAAAAAAATGGGACAAAACAAATGAATGAATGTATCTTTTCTCTTTTTGTGTTTCTTCTCAATGCAGGATTAGAGACTTGGAACCAAGCTCCTCCATTATGTAGACAGAGTAGTGAAGATGGAACTCTTGGAGAAATTAATGGTCTTTGGTCATTGGAAGATTTAGCTAGAGTGATGTAA

>CrNAC-33

ATGTCTTCTTGTTTTGACGATGGTAAATTTTTCCCGCCGGGATTTAGATTCCACCCGACGGATGAAGAGCTTGTTCTGTACTATTTAAAGAGGAAGATCTGCCGGCGGCGACACAGGCTGGACGTTATCGGCGAGACTGACGTCTACAAGTGGGACCCAGAAGAGTTGCCTGGTACAACTATTGATACTGAATTTCGTTTTGATTTAATTTTTGATAGTGGGTTTGAATAAATTGATTCGTCTTGCATGTAATTTTGGCGTTGACTTGATTTTATCTGCTGTGTTTGCATACTTTTGGATTGGATTTGAGTTTTCATTTGGCGTGATTATTTGATGAAATCGCACATACTTAAAACGAGTTTCATGGTACTTATTTGACTTGTATCTGCATTTTTGGGCCTGCTGTTGATTTTTTTTACTCTTACCGCGTGATCTTTTGCAGTTTGCTAATCGGCTTGTACACTCTGGCACATGAATTCGTGTCAGTTTGGTTTAATTTTTTGTTTTATTCGATCTGGTTTTGAGGGTAAAGGCTTAGTTTTGACTTCTGTCTGTGTGGTTGGGATGATGTTGGTGAAACGTTGTTATGAATATTGCAGCATTTGCTTGTGTACTTGCATATTTTGGTCTTCATTTGTATGCTTTTGATTTAGTTGTTTGTTATGCATGCATCATTTTCTGGTTTCTTGGTATTTCTCATTGTCAACTAGTTAGATTATCCGTCTGTTAGATTGGTTGATTGGTGTTGACATGAGGCTAATTGAAGATTCTTTTTGCTTGTATTTCTATAGTTTAGGTTGATTTGGGGATTCATGAGAGAAAATTGCTTTTGTCATCTAAAAATGCTAGTTGAGTGGCTTTCCCCCTTTTAGTGAAGTTAAAATTTTCAAGTACTCTTTCCGTTACCATTTGTGATCTGGCTTATAAAATGCAAACTTTAGAGGAGATTAAATTAGCAGCGTTTACTGAAACTAGACAAAACACTGTACTTGTATTTCATTTAATTGATTAAGTTCATTGCTGAACCATAGGTTTAAAGACTGCATGTTGTGGAAGTATGCTTCTAGGGTCTGGATGCCTCAATAGATGTTTACTTCATCAGGACATTTGTGCATCATGCGTATTCATGCCTTTGAAATATAATTTTCAGCTTATTATACTAACCATCTTGTCTCACATTTTTATTGCAGAAATATCTAAGTTGAAAACTGGAGACAGGCAGTGGTTCTTCTTTAGTCCCAGAGACAGGAAGTATCCAAATGGTGCAAGATCGAATAGAGCAACCAGGCATGGTTACTGGAAAGCAACTGGGAAGGACCGTATCATTACTTGCAATTCTCGTCCTGTTGGGGTAAAGAAAACTCTGGTTTTCTACAGAGGGCGTGCACCTACAGGGGAGCGTACTGACTGGGTAATGCATGAGTACACCATGGATGAAGATGAGCTGAAAAGATGTCCTGCAAAGGAATATTATGCTCTGTACAAAGTCTTTAAAAAGAGTGGACCTGGTCCAAAGAACGGTGAGCAATACGGTGCACCTTTTAGGGAAGAGGATTGGGCAGATGAAGTTGAGCCTAATGGGCTTGTTGAGCGGTGCAAGTCTGTAGAGCAGGTTGCAGAATGTGTGCCTGTTGATGATAATAGAATTAATTCTCAGCTGCAGTCCCAATTAGAAGATCTTGAGGAGTTCATGAACAGGATTGCAGATGATGCAATACTTGAACCACCACCAGTTGATGACTTTGCTTATGCTTTAGGGGAGCTTGTGCGTGAGGAGGAGGCACAAAGCAATATAGTGGATCAGTCCTCAAAGGAATACAATTTACCTGAGCAAAGCATTGTGGTTCTGCCACACTGTCAACAATATGATGTGCAAGCTAGTTATGACTTGACTCAGTCTGCTACTTCTCAATTTCAGTTGCATGAGACTTCTGAGGTCACGTCTGCTCCAAAGGGTCACATTCCAGAGCCTTACACAGTTGAAGAGGATTTCCTTGAAGATTTTCTGGAGTTGGATGATCTTATGGGTCCAGATCCTTCTGTTCAAAGGTCTCATAAACAAGCAGAAGCCCATGGGAACCAAGATTCCATTGATGAACTTGATGGTCTGTTCGAATTTGAATTGTACAAGGATGCATCATTGGTTCTTTCTGAAGTAGGACCAGGGGGAGAAGGGCAGCTTTGTCAAACATACGTGAATAATATGGTGAGCAAGGCTGCAGATCCGGTTTCAAATTTGTATTCTAATAATTTTGAGACAGGGACCATAAACTATCAGCAGACCAGCCATTCTCCAACTGAAAATGAGGTGAATTTTCAGCAGTGGGAGAGGTGCAGTGTCTTTACTCCTGCAGAGGCACAGCAGGGCACTATAACTCCTGCAGTTCCAGGTACTGAAACGACTGATGACTTTGTTGGTGCCCTTTATGATTGGGTTTCACAATATTAGTGCATGTATTTCATATATTATAAAAGACTATACTATTCTATTAGCTAATCTCACCGAGCTCTTGGTCAGATGGATGGTATGGGATGACCATGTCATCGTCCACATGGCGAAAACTCTACTGCCCAAGTTGAAATGGGAGAGTTTGAGCCAGCTTCAAGAGCTCCTCTGCTTAAGCAGCGGTATAAGATTGAATTCTAATTTTCACAAGTTTTGGCATGTTTAGGTGTTTCTTATGATGGTAATTCCACAAATCATCACACTGGAGCCAATGAAAAAGGGGATGATGGTTCAGGCTCTTGGTTCTCCTCGACTCTCTGGTCTTTTGTGGAGAATATACCAACGACCCCTGCTTCGGCTTCAGAAAATGCTCTGGTGAATCGTGCTTTTGAAAGAATGTCTAGCTTTAGTAGGGTAAGAATAAATGCTAGGAACATGAATGTTGTTGCTGCAGGTAATACCTCTGCAACTTCAAGAAGTTCAGGCAAATTTAGAACTGGATTTTACTGTTTTTCATTATTGGGTATCATGTGTGCAATCTTGTGGATGTTGATAGGAACATCTGTGAGAGTCCTTGGAACATCTTTATCCTAATAAATATGTATATCCTATATCCTAGTTTTAACTGTTTTGTCAAAATAGTTTGAAGGCCAAAGTTGCTGCTGAAGAATGAGGCTTTCTATGCGCTTGATTATGCCTTAAAAAATGTAATACATATTCATGCAACAGGCCTTGTTTTAATGAGGTGTATCTAGATCTGGTTCCGATTTGGATTGGAAGATAATTTGAAATCATATGACTTCTTATGACAATGTAAATATTTGCTGATCTGCAGCTAAAGAATCTTACAAACATTCTTCTGCACCTTTTTCTATCTTGCCAATGGCATTTGAGTGCAT

>CrNAC-26

CATCTTTGCTGCTTTCTGTGATGCTTCCTCCGCTACGAGATCCATACGGAGCTGGGCCTTCGAAGCCCCACTGAATTTTCCTTGAATCTTTATCTGTTCACGAATTTCGCCCTAGGGTTTTGTTCTCGTTTTCACCCTTCCTAAAAATCCCCCAAAATCTCTCTAATCCTTTCTCCTTTACCATTTTAACCTAATTGTATCTCTCCAGATATATATATATATATATGTACTGAGTTAGTGGTATCTTTTTCTTCTATTGAATTAAGAAAAGTCTTCGATAACAGTGATGGCTGTGCTTCCGGTTAAATCGCTGCCTGTGGGCTATAGGTTCCGGCCGACGGACGAGGAGCTAATCAATCACTACCTGAGGCTGAAAATCACTGGATTCGACAAAGAAGTTAACATTATTCGCGAAGTTGATATCTGCAAATTGGAGCCTTGGGACTTGCCTGGTAATTAACTTTATTGCGATTATACCCTTTCTATTGTGCAAAGATTTAGGTTTTACGCCCTGAGCATTCTTTTCATGGGGGAACAAGAGCTCGAGTGCGAGATTTTCAGAAATGTGGGTTAATTTTGCTGTGATTCTAGAATTATAACTGTTGGGTGCTCAGATTGTAGAAGAGGGAGATAATTGCGTATAGGATAATAATTTTCCCTGTTTTACGTTGTCAAATAATTTCAAATTTTTCTTTAAAGAAATTTGTAATTGTGCTTAATTGCGTTGGAGAAACTAATTTAGCATGTCTCTGAAATTTTGCAGATTTGTCGCTGGTAGAGTCATATGATGATGAGTGGTTCTTCTTCTGTCCCAAGGATCGGAAGTACCAGAATGGCCAGCGGCTGAACCGAGCTACTCTGAAGGGTTACTGGAAAGCCACTGGTAAAGATAGGAATATTGTTTCGAGGAAAGGAGTGAAGATTGGGATGAAGAAGACTCTGGTCTTCTATACTGGGCGTGCTCCAGATGGAAAGAGGACTAATTGGGTGATCCATGAATACCGTGCAACTCAGAAGGAGCTTGACGGCACCCACCCTGGACAGGTTCTCTCTCTCTCTCTTTTCCTCCCCCTCCCCCTCATTTGA

>CrNAC-31

AAAAAAGGAAAAAAAAGGGATTAGTACTTGTGAAAAATATAAATAATATAAAGAATATATAGAAGAGAGGAAGAAGGAGAGGAAAGTGGACTGTTGTTGTACTGCTCCTTTTTTTTTTTCATAGAAATAGAAGATTGAAATTGAGGGAGATCTGCTGGGCTGGGCGTGTTTTGCAGAGGAATTGGGTGTGGGGGGTTTTGGTTTTGAATGGGTGTTTGTCTCCATGGAATCCTTACCGCTGGGCTTTAGATTTAGGCCGACCGACGTCGAGCTTATCGATCATTACCTCAGGCTTAAGATCAACGGCCGTCATTCCGAGGTTCAGGTCATACCCGAAGTCGATGTCTGCAAGTGGGAGCCATGGGATTTGCCTCGTACTTCCTCTCTGCTCTTTCTTCTTTTTAATTTTTTTTTATTGTTTATTATTTTCCCCCTGCCCTGCCTCTCTCTGCTTGCTAATAGTAATAAGAAGGGAGTTTTATTTATGCGTGATGTGCCTTCCTTGTTTCATTTTGATTCGATTCCTTCCTTCTTCTTCCTCCACCAAAATTTCCTTTTTTCTTTTTTCATTTCTTCTGCCGCATCTTTCTTTCATTCTTTTGCTGGTTTTTGGTTTTCGGCTAAACTATAATAATGTGCTCCTCTTCGTTTAAACTTGTATGTCTACCTCAAAAAGAAAATTCTGCATCTCGACTCGAATCTGTCCTCACCCCCTCTTTTTCCCTACAGCTATTCTATTATAGACAGAGGTATCAAATTCCTCTTTCCACGCGTCACAGTAGACGAATTTCAATCAGATTGCAATTTCCTGGGTCTATCCCTCCCTTTCTTAGCTGAATGATCATATCTGCTCTATATTTTGCAGACATGCTTTCCTCTCGTATCTATCTCCTATTATAGATTTCTTTTGATAAATCTCCAAATTTGAAACCTCTAGGGTTGATTTTTCCTTACCTAGACTGATAATGTGGCTGTATCTCCTCTTCATTTTCTGTTTATGTTACTTGTGGATGTGACTGGGATATCACTAATGTATGTTTGTGTATGGAGCTCGTCATCCATGCAAACTATGAAATTGGATACATTTTTATGCAGAAAAGAAAAAAGGAAACGTCTTTTTGAAATGGTTTTCCAACGGTGTTCAAACTGCTGTATAAATTTTCTGCTATTGGAATTGAGTTGCATGCCCTGCAGGGACTGATACACTGCTGTTGTTAACAGGGCTTTCGGTTATAAAAACAGATGATCCAGAATGGTTCTTCTTTTGTCCACGTGATAGGAAGTATCCAAATGGACATCGTTCTAACAGGGCTACCGATGCTGGTTACTGGAAGGCGACTGGCAAGGATCGCACTATCAAGTCTCGCAAATCTTCTCCATCTGGTCAGTCTAACCCCCAGTTAATTGGCATGAAGAAGACTCTTGTTTTCTATAGGGGTCGTGCACCTAAGGGTGAGCGCACAAATTGGATCATGCATGAATATCGCGCTACTGAAACAGATCTTGATGGAACAGCCCCTGACCAGGTTGGAGCTGTTTTTATTTGAACATGCTTTCTTTTTCCTATTAATTGTTTCCGAAGTTTATTCCTAAATTTCTTTTATTACTGAACTCTGTGTTCTTGATGTAATTTTTAAAGAACTTTGGAGGAATGAGATTGAAGCATATGCATTTTATTCTTCAATAGTTAAAGATGCTTATGTAATGTAAATATTGAGGTATAGGCTGATGATGGCATTTTAAGCTCTGTACTTCATCATCTGAATCAAGTTGTTAGCAATAGTGATAGTAGAAATGACAAATTCTGCTATTGATATGGAACCAGGAAGTTCCGAATGAGTATTGTGGAAATGCATTAGATAAGAATGTAACTTTCTGTGATCAGTAGCTCATCTTATAAAATATTTTGTTCAGAAATTAATCCTTTGGACTTGTGTTCAGTTATTTGGGAGTTATACAGTCGTGTGCTCCACTATGAGGCATATTAGTGCACTGGCACTGGTTGGCTGGCAGCCAATGCCAATGGGAGGATATTCTTTCTGGTCATGAGTTTATGGTTAGTTTAGGCATTTCCTGCATAGTAAAAGGATTTTAAATTTGACTTTGTCCCGTGCTTAGTTGATACATATCCAATACAAAGTTGTGGTACCTTTCATGATCCACTGGTAGATGACTGGGGTTGATTCATTGGTATGCTGTCTTAGCTGACATCATATTGACAGACTCCTGCTTTTCTCCTTCGAGGATGGGCATCTAAAGTATCTTGACATCTCTGTAGACCTTTACATCATGAGGGCTGAATACCACGTAATTGCCTACATTTGTTAATTGTGACACGAATAACATTCTGTAACTCCCGCTTGTGCATATTCTTGCACGGCATGACCACTGTGTCTCCAATACACTTGATCGGTAGTCTTGTGTTGTCTGCTGTAACGACGATCCGGCTGTCCTTATATTCTGTCTTGAGGAAAAACTTTCTTCGTCATTAATCATGTTATTTGCTGCCCTAAGTCGATAATCCACTTGCTGCTGTATGGATTTGTTTTTGCTAGCAAGGTTGCTACTTCTGTCGTGACGTAGCTAGTTTTTATCTCCACTTTGGCCCGCATTGCAAGGGTTGCTTGAAAGTCCCACTCATCTTCACGATAACTTGAACGAGCTTATGATATTGTTACGTTTCCTTCTATTTGTTTTTTTTTTTCCTTGGACCAACAATCCTGTGCATAATGCCCTTTCTTGCCACAATTGTAGCACAGACCATTGTTTGGGCTCTTGTAATCTCCTTTGCTATCTTCAACTCCCATGCTTCTCGGGATGTCTATTAATACTTCTAGAGCCTTGACTCTTGTCTGGTCTTGGATTTCTTCTCAAGCCTCCTCTTCCTTGGCTGCTATATGATGCCTCCTCTTCACCTTTCGCTTGAGCTTTAGATTTCTGTTTATTAAGGGGTATCTCAACATATTTTCCGACTCATTAAGGTTTGGTTCTTTGGTCCATCCCCATGTGGCTGTTATGATGGGGTTGAACTCTTGAGAACACGTATTATTATCCTTCTCTTGTCTCATCGACAGGATGAAGAGGATTTAATTTTGTAATTTTCTCGAACAATGATTTAACTTTGCCAAAGAATTGACTTCCAGTTAGGTCATCCTGTGTAAGTGTCACCAGCTCATTTCCGAGTTGTTGAGAATCTCACATGGCTAGTGTTGATGAAAAATTTACTCAAAAGGTTTGGCCAAGTCTTCCCTCTTGAGCTACCTTTTGGGGTGAGTTAGGCCCAAATCCAAGATTTTCAACATGGCATCAAAGCTACCTAGTTCATGTTCGGCTGCCTGTATTGGGCTACCAGTCGATGTCCACTCCTGCAAATTTCACGCTCTAGATGTCCAGGCTTGGGTGTGAGGAGGGTATGTTGTCTAAGAATAAATGTGAAATCTTGGGGCCGCTTCTTGCAACTTGGAATGCTTTCCTCCCTCCACCTCCTGAGTGATCCGCTGTCCAAATACCTCCATACAAAGGACGAAAAGGTAAGGGGATAAAGGATCCCCCTGCCTAATCCCCCTTGAAGGCTGGAAGCTCTCTAGTTTCCCACCATTCCAGAGTGAGGTCAAAATCGTACAATCCATAATCAATCCAGCCAAATGATTGCTAAATCCCACACTTAGCAGCACTTGTCGAAGGAAACTCCAATCTATTCGAACATATGCTTTTTGAGGTCCACCTTCACAGCATTCCATTCGGAAGAATTGGTTCAGCATCAACCCCGGGATTCTTTACCCGACTAAGAATCGCACATGGCTAGTTTTGGAAAAAAAATTACTCCTTAAAAGGCTTGCGCAATCCTTCGCTCTTGAGGTACCATTTGGGGTGAATTAGACCCAAGCCCAAATTTCAACACGAGTCTTTATAGCTTAGAGTTGTTAGCCTTCATGAAGCAGTCCGCTAATACATACTATGCAAATGAACCCAAGCGTCTATTTATACTAGTTGTTCACTGACCTTACTATGTTGGTTTAGCTAATTCTTAGACAAGAGTCTAGAGACAATTCAAGCTCCATTTTTTTCTGCTTCTAGAGTACTCATTAATATAAAAAATTAGATCTTGAATCCTCCATATAGTTCTAGAGAATTCTATTGTTGCTTTCTCAACATGCTTTTGGGGTTGTTTGTATCCTTTAGCTAATTGATTCTATTTTTATAATTTCTCCTGCTTTTACATTGTGCAGGCTGCATATGTTCTTTGTCGCTTATTTCATAAGCCTGATGAAAAGACTGATAATTCTAAAGATGACGAAGCTGAACACACTGGTTCTTCTCCTACCACAGTAAGATCCTCACCTGATGACACATCATCAGATCTGTTTCAAGAACAACCTATCCTGGATATGGAAGTAGGCAAACAACCAGAAGGTATGACGAGGTGGCTGACAGATAAATCCGATGAAATGACCTCAAATAGTCATGCACATGTCGAAAGTTGCATGTCTGATGCTGAAGATCACTCGACAGGTGCAGCTGGCACTGAGGTATGAATTTCAATGTTGTTGAGTGAAATCTCTTACTGCAGCCAAAGATTGCCATGTCACTTTCCAAGAAACTTCATGTCAATCGTTTTGTTTCCTGCTGTTTGTTTTAGGTATATCCACTTCTAGGAGGTGATCCAACAAGCCAAGGGCCCATGCATATTCATGATCAGAAGGTTTTCTCCGCGTCGTGTTCAAATTGTAATACAGATAAAGCATGTGCAGATTCACCATTTGCTGATGACTTTGGCAGCAATCACAATGGATTGCACTTTCAAGATGGTACCTCTGAACAGGATGTTTCCTTAACAGAATTGTTAGATAACCTTCAGAAACATGAGGAATATCCTTGTGAGGAGTCAAGAAGTAGTAAATTCTCGGATATTGGTAGCAAGAGCATTGTGTCTGGGAACAGTTATCCGCTCCATCAAGGGCCAGAGAAGAGCGAAAATCTCCGTGTCTCTGGTTTATTCACTGAGAATGGTGGTAGTATGTTTCGAGGACAGGTAAAAAATGCCTTCTGTCCTCTGGATTCTTTGCTGCAAATCTGATCTTTGCGAGTTTGCCAAGCTAGCATAGTTTTTGTGCTCCACAGGGCTTGCTCTGATTGTGGTTGTAGGACTTGAAGACAGATATTTCATACACTGGAATTGTGTTTTCTTCTTCTCATAACTACAGTTGGTGCTTAGCTTTTCTCTATGCTAGTATTCATTTTCGGGCATTTCTGCAGGTTTGTGGTTCACCACAGTCGCTCCAGGCTCAAGTATCCAACTATGATGCAGAGGGCAGCATGAGCAATTTATGCTCACTTCATGATCAATATGCTGAAAACAATATGTCACTCGCCTATTCTGTGATGCAATCTCTCCACAACCCCGAACAATCTTTTGATCATATAAATCCTGCTAGTCATGGTAGCAATGTCGATGATAGATCTGGGATCAGAATTCGTCAGCGTCAGCGACAAAATCATGCAAGTCCTGAAAATCTTTCTACACAGGGTACTGCAGCCAGAAGAATACGTTTGCAGATGGATAACACCCAAGCATGCTGTGATGCCAGCAGCTCTAGTGATGGAATGCAAGAAGAGTCTGTCATCATTGAGGTGAGATTTTATAATCTATTAGTTGATGCTAGTGAAAAATATGACTTATAAGTAAGGAAAATTTGTTTTCGCGGCGATGAAAGAAAAAAAAAGAAGACTTCTTACTTTGAGTGGCAAATATGTCCCTCCTTTTCAAGAGATGGAGCCTTTCAGCCTTGTTCTTTCTCGTCAAAATACTAATTCTTTTACAAATTTGAAATTCATTTTCTCAAAATTACTCCCATCCATAACATTCTCTCATATTAAACACAGCCTTTATCAATATTTTGGACCAAGTGTTTGGAGAGGGAAGAGGGAGGAACGTATGTAGGTGGAAAAAGATGAAAGAGAATTTGTAGGATTTATTAAATGTATGTTTAGGATTCTTAAGTATTGTACAATAGGTTAAAATGTGGGGTGGGATCATTTGTTACTGAAGTTTCAATCTTGTGTAGAACACTGTGATTTCTGCGAATTTGCTGCCTCTTAAAATTAAGGAATATATTTGCATTTTTGCATGCAGGCCAGTGAGGATTCGTGGAATGGTTCAGAGGTGGGAAGAACCATTAATGACCACAGGAAGATTGTTGAAGAAAAAGGAAAAACGGGAGTTGGGTTGATAAATAGAAATAGGAAGGAAGATATTGGTAAAGGAATGAGGTCAATGTTTTCATGGAGGACAACGCATGGTGTCTGGGGAAGAATTTATGCGATCAGCTTGTACATAGTTGTAGCATTGTCGGTAATATTTGTTAGGATATCTCGGTGTCCTAGCTTCCGTGCTTTAGATAATTGAGGTGAGATGCAGGCAGTAATCAGTTGATGTATGTATTAGCCCAGCATTTGAGTTTTTGTTTGTAGACTGTTGGGCTGGTGGCTGTAAATTGCAGATCCTTGTAGGGTTATATTGTGCTGGCCTAGTGGCCTTGCTCTTACTCTCCTCAAGTGCTTTTTAGCTGCATCCTAGTAAATGTATTATTGGGAATGACAATATTAGCAGAGTTATTAGTAGTGTTAGTAGAATAGAAGTAGTGCTAATACTCATTAGTAGCAGGTACTGTAATACAGGTAAGGGCAGTCCACTACAGAGTTTTCATCTGGTTAATGCCGTAAAGAAAATAATAATACAAGTGCTTTCTTTCTCCCTTCTATTATTATTAATCTTTTTTTGGTAAAACGGTGTGTATACAGCGTGCAAGGGTG

>CrNAC-32

TTCTCAATTGCTTTCTTTGAAAAACCTCCTTCTGTTTTGATCATAAAGTTGCCTTTTCTTTTTTTTTTTAAGCTTTATTTTTCAGTTTTAGTTTATGCTGTGAAAAGGGGTTTTCAGTTTCTTTTTGTATTCTTTTAGCATATATATCCATCTAGAGTTGAATATTTAAGGAAGATCAAGCAAGAGTATATATGGAAAGGGAAGCAGCTAATATGGTGTTGAATATTAATGAGGAAGATCGAGAAGATGATCATGATTTGATGGATTTGCCTCCGGGTTTTAGATTTCATCCCACAGATGAAGAAATTATCTCTTATTATCTTACGGAGAAGGTTATGAATAGTGGATTCTCAGCTAAAGCTATTGGTGAAGTTGATATGAACAAGTGTGAACCTTGGGATTTGCCCAGTAAGTTTTTTTTCCTTTTTTCTTTTTTTCATCTTTCATTCAATTTTTTTTTTTTTTGTCAGCAAAAAGGGTTTTGAATATCTTTGACTTTGCATTTAGATTTTCATCTCGATCGCACAAATTACAATGAATAATTATATTTTCCTCCATTTCTTTGCACAAAATCATATATATATATATATATAGATCACAATTGGATATATATATATGATCATGGATTTTACTGAGTTAATTATTAGGCAGGGATTAATTATCTGCAATTAATTATATGATGATGATGATGATGATGATGATTTCCTGATGATTTGATGATGTTGTGGGATGCAGAAAGAGCAAAGATGGGATCAGAAAAAGAATGGTTCTTCTTTTGTCAAAGAGATAGGAAATATCCAACAGGGATGAGAACCAATAGAGCAACAGAATCTGGATATTGGAAGGCAACTGGAAAAGATAAAGAGATTTATAATAGTAATAATAATAAATCAGGAAAAGGTGTTGGTGGTGGTGGTAATTGTGTTGTGGTGGGGATGAAGAAAACTCTTGTTTTCTACAAAGGAAGAGCTCCAAAAGGGGAAAAAAGTAATTGGGTCATGCATGAGTACAGACTTGAAGGCAGATTTTCCTATTATAACTTTCCTAAAGGCGCTGCAAAGGTAAGTACGTACCTAGCTATAGGTTGCTTTTCTAATAATAATTTCTTTTCTTTTGCTTAAGTAATTATCTTCTAACAACAACAAAGTAGTAGTAATTAATGAACAAACAAACACTTTTCTTTTCCTTTTGAGTTCAAATTAATCTTATAGTAATTAAAATTCTTGAAAAATTAGTAGAACATGATGATGATGATGATTACGCGAATGCTTGGTGCAGGATGAATGGGTTGTTTGCAGAGTTTTCCACAAGAATAATAATAATAATAATATTTTGAGGAGGGTTGATTCTTTTCTGGATCATATTTTGGATAGTCCTAATTCGCTACCACCATTAATGGATCTTCCTAGGATCAGGAGCTCTAGTACTGCTGCTGATAGGCCAGCAGCAGCAGCAGCAGCTTCCACCACCAGCTATACTCACGATGACGAAGACGAATTATTCAAACCTTCTATTTCTGCTTCTGCTTCTTCCTCCCAATCAGTACTACTACAAGAAGATCAAAAAAACTTTACCATACCCCCAATTAATTATTACGCCACCCCGACCCCGACCCCGACCCCGACCCCGGCAGCTTTCATGATCAACAATTCTACTTACCAACCAAGTTCTAGCTGCCAAAATCCATTATTAATCATTCCAAACTCCATTTTCTGCCCTCAAAACTCACAGGATCCGAATCTATATGAAACTCAAGACCGAAATCTTGGTTCCATCCTGAATTACCTAGGAGGCTCCGAATTCAAACCGCCGGCTGTTGATCAAGAAATATTTAGGCAGTACTGTAAAATGGAACAATTCTCATCAAACAACAATTCTAATATGGTCATGAGCCAATCACAAGAAACGGCCTCCCGAATCAGCAACAACGATGTCAACTACCTACAAAACTCATCAATGATGATTAAGAAGAGATCATCAACATCGGACAACATCATCATCGGAAACAACATGATATCATCATCATCATCATCATTTGATCATGAAGATCTCGAAGCTGGGCTCGCGGTGGCTCCAAATGACATATCTGATCTAGATTCTTTGTGGGACTACTGACTATATTTCCGTGAACAGGAATTTGCGGATTATTCTTCTTATTTGGAGGCTAGTTTAGTGCAACAAAGGATGGTTCTAACTCCGAATTGA

>CrNAC-03

ATGGCACCAGTCGGATTGCCTCCGGGATTTAGGTTCCATCCGACCGATGAAGAGCTTGTGAATTACTACCTAAAGAGGAAAATTCATGGACAAGAAATTGAACTTGATATCATTCCTGAGGTTGATCTCTACAAATGTGAGCCATGGGAATTAGCAGGTCTTATTCATTTCTTGCTTTCTCTCCTTTTTTTCAATCTCCTTTTCTAGCGAGATCTCGTTCAAAGTGTATTAAGAAATGCACAAGCCGATCTGAGTGTCTTGCCTTCTTCATCATTATCAATGTGTCCTTTTTATCATCATCATCGTTCTCATGATTTTTTTTTAATCATCACATACTTTTTCATATTCTTGATGATCTTCCTCTTCTATATTTTAAGCATGTTTCTTAGCTACCTTATTCCTCAAGATTCACGTTGATCATAAAAACTTAAAGTTGGCTTTAAAAAAAAAAAAAAAAGGACAGAACTTAACGAGCATATCTGATTTTACTACTACAAGGAAATTAACAATTTTTATTATTCTAATAGTACTCGAAATTGCATAAATCCTTAATTTAATCAGCTATGCTATTCAGAGATTATTGAGTTCCAACCAAATCCTCCAGTTCTAATGTATCAAAATCATTTAATTATTTTCCGAGTTTATTAATTTAAAATTTTTTTTTTTCGAGTAATTGCAGAAAAATCATTTTTACCAAGTAGAGATCCAGAATGGTACTTTTTTGGACCAAGGGATAGAAAGTATCCAAATGGATTCAGAACAAATAGAGCAACTCGAGCTGGATACTGGAAATCAACAGGGAAAGACAGAAGAGTTTCTTCCCAAAATAGACCAATAGGAATGAAGAAGACATTGGTTTATTACAGAGGGAGAGCTCCACAGGGAATCAGAACTGATTGGGTAATGCATGAATATCGACTTGATGATAAGGAATCTGATCAAGATACTTCCGGCATTCAGGTAGTTGTAAACTTACTTACTCTTTCTAAATCAATCATCCCCCACCCATTTATCAACTATAATTATTTTTAAACTTAAATATACTCTCATTTTTTACTATTAATTGTTACATTTTGAAAATCACATTATTTAAAAAAATATAAAATTTTAAATTTTTTTCTTGTCATGTGGACTAGAATGCCTTTTCAATTATTCTCTCTACTCATTATTATTTTTACAAATTTCCTCGTAGAAGAATTAAAAAAGATTTAAAATTTACTTTTAATGGCAAGGTAAAATGAAAAACAATTAATTAAATATTGCATTATTTTTACAATGTAAAAAGTAAAAAAAAAAAAAAAACAAGAATTAGTAGAATGGCATTTTATTTGTCACAATGTGACAAATAAAAAACATAAGGGAGTATTAGTCAATGCTATTTTATACAAATTTTCATTGGTATTTCCATTTTCCTCTTCTTTTAGGATGCTGCATTATATATATTGGTTATAAATTTCTTGCATAGGAGCGATTTTCAATTTTGAAAAAATGATTATAGTTTTACATAAATGTGTTATTTAATAACAGTGCATCATATATATTGTTTTTGATTTTTTTATTTATATACTATTTTATTTTCATGTATCTATTTATATCTTACCATCACCATAAGTAGACAATGAAATCAGGTGAACCAAATTAAGTACATCTATTGAACATTATATTCCAAAATTGCGCTAAAATTAATGGTATGGTCAATAGGTATGCTTCAGCTTGACTGATTCCACTACATGTAATGCTGGTGTGATATAAAAGACTTGAGAAAATAATATATATACATATATAATGCACAATCAGTAATCAGTAAATAGTAAGTGGGTAACTTTAACTAATTTTCCTTTGAATTTTCTTGACTTTCTTGATGATTAGGAATGCTGTTTCAAGTTAAAATGTGAATATGGAAGAATGGGTTTAGAAGTTTTCTTGTTTATTTTTGGAAGTGTCCTTAAACTGAAAATGATGAAAGCGAATTGGATTCTGGTGATGCAAGTTTGTAAGTTTCCACAAAATCAGTTTTCTTGGGAAAGATGTAAACATCATATATTTTTTTAACCCTTTCCTTTTTCTAGTTTATGATTTCAAACTTTTTTCAGGAAAAACAAAAAAGAATGATTAGTGAAGTCTTTAATCATCTTCTTACAGATTTGACTCTGAAAGTATGAATTTCCAGATCTTAGTTAGTTGGAAAGTAACCCCCTTGTTGAAATTTTCACTTCTTTTACACAAAGGATGGGATAAAAAAGCAAGATGATTTAACATTACCATGTGCCCCATGTTTTTATGATACTCCTCCTATACCCCTAAACCCTCACCTCCATCCCACCAAACTTATACAAAAGATTATCCTAAGGATTTTTCAGATTGGAGTTTTATTTGAAAAAAAAAACAAGGTTTAGTCAAAAATAAAATCAGACCTAACACTTTTAGTATTACACCACTAGAAATATTTAAATCAGAGAAGTTTCATAATATTTACATTATGCTATTCATTAGGCCAACCTAACAGGTTAAAGTAATTAACAAAAGCTAAGATATGGATGGTAAATATGATATCTAGCAAGTTCCCCAAGATAGATCATAGATGTACAATACAAGGAACGTGATTTGCATGGGCATGCATGGAAAGCTAGGGCTTTTGTGATTATTTTTTTTTCTAGTACATAATTTTCTATTACGGAAAAAAAAGGTGACAAGACAAACATATATTCTTTACTTGCAATAAATAGCATTCCTTAGATCTCTTGAGTGACAGAGCTTGCATTGCTTCCCACTTTGTCTTTTCTATAATAATCAGACAAAAAAGTACAATCCAACCAACCCAACCATATATCACTGAATCTCATCAAATTCTTCTATTCTCTTTTGTTTTCACAGATCATATGATCTGATCTGCTAACATTAGAATACAAGATTCTTCTACAAAATTTAGGATTTTTTTATTATTTTTTTCTCTTTCTAGACCTCAAAAAGCAGCCCATCTCCATCCCCCAACCCTTCACTTTCATTTTTGTAAGAAGGTGTCGGTCACTTTTCACTTTATTCCCAAATACTGGAATAAGCCAATAAATAATCATCATGCTCTCTCTCTCTCTCTCTCTCTCTCTNNNNNNNNNNNNNNNNNNNNNNNNNNNNNNNNNNNNNNNNNNNNNNNNNNCTCTCTCTCTCTCTCTCTCTCTCTCTCTCTCTCTCTCTCTCTCTCTCTCTCGGCATGCCTTCTTGAGAATATCCAAAGACATGATGATCTAAGCACAAAAATAAATACATAAATGAGAGTTATTTTCTTTTTCGAAACTAAAAGCTACCATCTCTCTTTCAAAAACTTCACCATCTGCTTAATAAATTAATAGAGAATGTAAATTATAATAACTCAATAGTGCACTATATTAGAGGTCATTTACCTTGTATTCACAAGAAATCATTTCAAAAGAGTAAAAATTTCCCGTTATAAGTATGAAAACTCTATCTTTGAACTTTGAAAACCAACTTTGCATCTCCACAACCAACTAAACTATCCTCATTGGGGCAGAGACCTTTTCTTTCTTTATCCCATAAGTTCTTGTTTTTAACGTTATTGTTCCAATCTTTGGTTCAGAAAATATTTATAACAAAGGCCTAGACTTTATCAAATATAAAACTTATGTAATGAGAATTAGCAACTTTATTTTTTCTTTAAAGGTATATTTGTTTATAAAATATGCAATTGAGAATGTGTCATTTTGAGGTTATATCTTTGTCCATACTTGTGAAGAAAAAGACTTGGAAAGACGAAGGAAAAACCATAGACCAGAAACATCTAAAAGCTGAAAGAAATAGTATAAAATTTGTTAGAGAGAGAGAGATAGAGAGATTTTCAGATGACTTTTGTTGGTAATAGAGATCTTCTTTGTACTTGAAAAGCTTCTAAATGAGAAATTATGATGTTAGCTTTCTGGTTTATGTCTCACATCTTTTTCTCTGCTTCTCCTTCTCAACTCTCAACTCCAATCCTTTAGCCACCATTATTGACTGTTCATGACTACATCTATGACTTAAGATTTACTTGTCCCCCCTCACTCTAAAAGATAAAACAAGGGGATCTCACTCTCTCTTTACACATACAAACACAAAAAAAGATTACCGGGGACTCAATCCATAAATGGAGATATAGACACCTTATATCATATTAAGTCACATCATCCATCTTATTAACCATTAGGATTCCATCGATAATAGAATTTCTCTCTCTCTCTCTCTCTCATACTGCAATTCTTTCATGCAGGACTCGTATGCATTGTGTCGGGTGTTCAAGAAAAATGGGATCTGTTCAGAAATAGAAGAACAAGGGCAAAGCAGCAACAGCTTATCTTTGCTTGATTACTCTTCTCAAGGAGTAGTTATAAATGAATATGAACAAACATTTTCACCAGATGTTCCCTTAGCTTCATCTTCATGTATGGATGAAGAGGACAAAGAAGACAAAGATGATTCATGGATGCAGTTTATTACAGATGATGCATGGTGTTCATCAACTAACCCTTTTGGAAATGAAGAGGTTGTGGCTCAATTAACATTCACAAACTAATTGGATTGGGGTCAAATAATTTCAAATATTTTTTTCAAGTTTTTTTTAATTATG

>CrNAC-38

ATGTTGAATTGTTTTACAGGGAAGTCATTGTTGCCAAGCAAAGATCTAGAATGGTATTTTTTCAGTCCGAGGGATAGAAAATACCCGAATGGATCAAGGACTAATAGAGCAACGAAAGCTGGGTATTGGAAGGCAACTGGAAAGGATAGAAAAGTAAATTCACAAATGAGGGCAGTGGGGATGAAGAAAACCCTAGTTTATTATAGAGGGAGGGCACCTCATGGAGCTCGTACTGATTGGGTTATGCATGAATATCGCTTAGATGAACGTGAATGTGAAACTAACACCGGTTTGCAGGTATTTATTTTTTAAAATTACTTTTTTAGCCCTTTTCTTTTTACATATATATATATATATATATATCTTGCTTGAATCATTCATTTTCTGACTCAGCCTCTCTAGATTCTTGCTTCTTTTTTTATCAAAAGAGGTTTTGCTACTTTATAGTTTTCATCAAATTAATTTCTTAGGATTATTTTCTCAATATCCCTAAAATTATTTTAGTAAAGATTTACTCTATTGAAATGGTAGTTTTAATTTATGTGAAAAGACCAAATCATGTCAAACAGCTGTGTTGTTTGGAGATTGTCAAGTACACTTTCTAATACAATTATTTTTGTGCTACATCATTTTATATTAATTTTATTAATAAATCATAAGAATAAAAGTTAAGCTTTAAACTATACAAGATAAAATCCTAAGAAAAACATCACTATTAAAGAAAATGTACAATAGAGTATATATTAAAAGATGTATTAAACGATATGGAATGAAATTCTTATATTCTCATTTTGTTACTCCTTCTGTTCTTTTTGTTTTTCACATTTTAGACATTGCACTATTAAAAAAAATATGTAATTTTAGATATGTTTTCTTCTATGAACTAGAATACGCTTTAAGTTTAATTATAAAATGTTACATTGTTTCACAATGTAACAAGTAAGAAAACACATATAGTAATAAGATGTGATAAGTAAAAGAAAGTGCTCGACAGATGCTTACATATTTCTTTTTGGCATTATTTTTTCAGGATGCATATGCACTTTGTCGCGTTTTCAAGAAAAGTTTGAACATTCCAAAGATAGGAGATCATTATACTAGTGCAACAGCGGCAGCTAGTGATAGATCTTCCAGCATAGATCCATATTCTGCAGATGATATTGAGAGTTTTGATTATGCAATGCCAACAGCAACAGTAATTAGTGATAATAATAATAATAATTCTAACTATCATAACGTTTCGGCATCAGCTGCAAGTTCTTCTAACATCATCCATGGCAGCTCTCCAATGAACACTACTAGTACTGCTGCTGCTCCAACCCATGATAGTAGATGGATGCAATACTTGTCCGATGAAGCATTCAGTTTTAACAATAACCCCTCTTTCCCAAATTACATGAACAACATGCCATATCCTCCATCCAAGGTAAAATTCACCACAATTCTATATTGTTGTCTTTTTTATGTTTATTTTAGAACGAGAAAACCTAATAATACTATCTGAAGGCGTGTACTAGGTAAACTTTGTCATGTGCAAGTGCAATGATATAAATTGCGCAAGAAGGTAAATCACGATATCACATTAGGCAAGTCTAGTGTGACAGACTGATGTCACAAGTCATGTAGGTCTATATTGTTGTCTTCAAATGACAGTAAATCGACTCTTTACAAGTTAATTACCACAAAGAAATTTACTGTCACTATTTAAAGGTATGTCCTGAATAAATCATATATGATATAATTACATGATAGTACAAACTTCATGAGAAGAGAAATTATGTGCAATGTACTGATGTTAGAAGTTACACGTCTAAGTTCTTTTCTTTAAATGATAAAACTAGTGTAAATAAACCAACCCTTTACCAGTTAATTTTTCCACATTACCACATTCTCATATAAAGATTTATATGATGCAGGTTGATATAGCATTAGAGTGTGCAAGATTGCAGCATAGGTTTGCATTGCCACCATTGGAAGTTCAAGACTTTTCCCAAGCAGCTGGTTTTGCTGACCATGCTAGATCCATGGGACAAACAAGTTACAATATTCAAGAACACAACAGCAATAGTAATCATCAGCAGCCGGATATTGTACAAGAGATTCTTTCAGTAGCTGAAGCTTCTCAAAACTTTATGAATCAAGATCATACAACTACCTTTGGAGGAAATTATACTCATGACCATGATGATTTCACATTTTTCTCCCCCAACAATAACCAAATGTACGATCAAAGTTTCAGTAGGTCCATTGAAATTGGAGGCCTCCAGAATGAAGAATTCATTAGATCTGACAGAATGGTTGAAAACTTGAGATGGGTTGGAATGTCTGACAAAGATCTTGAGAAGGTACGACTCTTTCTCCTTTTTTTTTTTGGCTTATCACATTTTAAACGTCACACCTATCTGACAAATATTTTGATAACTTTTAACTTACAGACCTTCTTGGAAGATTACAAGACAGTTCCAATAGAAAACATTTCAAGTTTCCAAAGACAAGAGGAGAATCATCAGGTTCATGTTCAAGGTTGGCCATTGGCCTTTTTTCCATTTGTATAAAAACTTTTCCTTTTTCTTCTTTGGCCTATATAAATATATAGTTCTGTTATGGATTAGGTCGTCTGGACCCTTTGTATTATATTAAGCCACGTTACTAAATAGATCAGATCCTCATCTATGGGGTGACGTGTCTTAATATGTTCCATCCAAAGGGTTCATACTCTACTTGTGAGGCGTGGCCTGGTCTGTAACAGAATTTTCCTATCTTCCCGCCACGTTTCTTCTTAAAGTTTTTGAGTTTTTAGTAATCAATCACTGATATATGCAGTGATAATCAATTGAGTGCAGGAGAGAGCAGTCATCAAAATAATCTAAAGGAAGGAATTATGGAGGATCATCATCATCATCATCAAAATGATTACTCACTTGGATTTGGGAATAATCATGGTGATGAGAATAATAATGGTGAACATTTCTTAGATGATGATGGAAATATTGTAGATCATGACTTCTCTAGCAGTCCAAACTTTGATGAAGTATTTGAGAAAATAGAAGTGAGCCATGGAATGCTTATATCAACTAGGCAGACAGCCAACACATTCTATCATCAATTGGTACCTTCAAAGACTCTTAGGGTTCATCTTCATCCAATTCATCATCAAGATTTTACAATTACCAAATCAGATTCAAGAAAAGTACCAAACAATTCTAATTATAGACATGTCACCAACAAAGTCACAACAATCTTGATTGCAATTATTGCAAGTATGATGGCAGTACTAAATGCTTACTGGATATGTTACGGAGAATGTTTGACAGAGAAACAATGCTTTAAAGATGATGATGAGACGATAGAGGATGAATTTTCGAGCAAGAAAATAATGGGGTCGGTGGCGCTGGTGGCTGGCGGTGGCGGAGGAAGCATTTCTAGGATG

>CrNAC-20

CCGTGTCAAGCAAGCATCCCATCTCTAATCCAATCCCCCACCATTTCTCCAGCTTTCTTAACCGATGGGCAATACCCTTCTCTTTCTACTGAGGGCTTATTAGCCTCTCAACTTCTCCTCTTCAATATATCAACACCAACTCCAAAAACTCCCCTTCTTCTTTACTTCTCTTCTAACCTTTTCCCTTTAACCTAACGAAGAAATCGTACAATCGCGTCATCTTTCTCTGTATATATGTAATCGGTGGAAGAGATCTTTTTTGTGGGCCTTAATTGTTTTTTTTTTCTCTTGGGTTCCTTTTATTTTTTGTTTTCCTTCTCCGAGTGTTCTGTTTTTCTTGAAAATTTGACGGAAAATGGGGCAAGAATTGGTTGCCGTCACACCTGCGACGCCGGTCGGAGGTGGGATTAGTGGGTCTGCGCCGCCGGCAACTTCTTTGGCTCCGGGTTTTAGGTTTCATCCGACTGATGAGGAGTTGGTTAGGTACTACTTAAGGAGAAAAGCCTGTGGAAAGCCCTTTAGGTTTCAGGCGGTGTCTGAGATCGATGTCTACAAATCTGAGCCATGGGAACTCGCATGTAATTTTTCCTCTCCTTTTCCTTGGGAGTTGTTTATTTGAGGATGGGTTTTTGTTTTTGTTTTTTTTAATCTAAACTTGCTTTGGTCTGACGGAATTCTTTTTTCTTTTCTGTCATCTATGGGGATTATTTTAGGTTTAATTTGTGTTAAAAGTACTTCAATTGTCATTTTCTTTTATCCTGACAGTAATTTTCATCTCTCTCTGTTTTTCCTCCCCACCGAACCATTTTCTATTTGTTTTCTTTTCTTGAAATTGCTTTTAAATTTAGATGAGATTTTGCTAAAACTTGAAGTTGAAATGAACCTGAGATTTTTACCAATGAGTTGCAATATCTGGGAGGTTTTCAATTGTTGTAATTCAATAAAAATGGGATCTTTTTTAGACTGACATGCAATATAAATGAGTTTTGAGCAAAGATAGAACTCTGGTGTTTGTTTGGTGCATCTGTACTTCTGGTATGTGTACAAATGAGTGAATGTTGTTTTTACTGATCAATAACACTAATTAATGAAGTGTTTATCTTTGTGTTTTCTAGTGTTTTTCCTGGTTAAAAGCTTGTCTTAGTGGTTTGATGTTTAACAAGTACATGATTTCAGATATGCTGCTTTATTTGATGTGTATGAATCTGATCCTTTTGTGGTCTGTCTTGTTTGCTTAATTTCTTTCACATTTTTTTTGGGTGCCTATCCTAATTCATTCTTGTCTTCTTTTTATGGTCACAGGCTTCTCATCTCTGAAGACAAGAGATCTAGAGTGGTATTTCTTCAGCCCAGTAGATAGAAAGTACGGTAATGGGTCTCGGCTCAACCGTGCTACTGGGAAAGGGTACTGGAAAGCAACTGGGAAGGATCGTCCTGTACGCCACAAGAACCAGACCATTGGGATGAAGAAAACTCTTGTGTTCCATAGTGGACGAGCTCCTGATGGTAAGAGGACAAACTGGGTAATGCATGAGTACAGACTTGTAGATGAAGAATTGGAAAAGGCTGGAGTGCCACAGGTGGTGACTGTGAATTAAGATTTGTAGTTTATAAATAGTTGATTGTGATTTTTTTTCTTGTTGGAACTTACAAAGAACTTTTTATTGCCATGGGGAAAAGGATACTTTTGTGCTCTGTAGAATTTTTCAAAAGAGTGGTCTAGGACCGCCAAATGGTGACCGTTATGCTCCATTTATTGAGGAGGAATGGGATGATGATGCAGCTCTTATGGTGGTTCCTGGAGGAGAGGCCGAAGATGATATGGCCAATGGTGATGAAGCAAGAGTCGGCTGCAATGACCTTGACCAGGTTTGTTTCCCTATTGGGTATTTGTTGAAATTTCCCTGTACTGCTGTTGCGTGTTCGTAATGAGTAAATGTTGTTTGCACATTGTTATCTCCAATCACTTCTGCTGGCGGCGAGGATCACGACAAACAACTTTTCGGGAATATCCTTTATTTCAGTATCTCGCCCGCTTTTGACATATCTGTGTGCTCTTTTTAACTTTCCCCACGTAATTACACATTGGAGATCAGGATGTCAAAGATCTTTATAAAAGCCATCTGTGGATTTTATGCACTATTAATTGCATGCACAAAGTTGTTCATTTAGGGTTCTTTGCCTTCTCAATAACTTTGGTAGAGTGTGCTCAGTTTAATGTCATCTCACGTTGTAGTTGACGATATTAAAGACGAAAACTTCTCGTACATTCTTTTTGTGTCGGCTCCGAGCCTTTCTCTCCGCGATGATGGGTTTTTTCTTATCATTTTCGTAGCAGTCATGAGAATGAACTGCTCTTCCTTCTGAGTCCTTTGACTAGCATGAGGTTCTTTTTAATGTTGTTGGTCAAAGCTTTGTATTTAGCGGCCTCATCTTTCTCTGTGCGCACACATCGGAAGCATGAAGGTTTAAATCTAAAGCAATTTGTAAGCAGTGGTTTATTCCTGCATATGATATCCCGTTGAGGGTTGCAACTCCTGAGAATATCTTTGCAAACTTGCGTTCAATGAAGATTAGTTCTTTACAATTTATTGCAGAGAGTTCAATTCTTCAATATCCTAAATCATTTGGATACCCTTGCTAGCACTTTCATGCATTTGAGACAACTGCAGATGTCTTGTCTCTCCTGTTATTATCCTTAAAGGGATATTGTGAGAGATAAAGGCCGTAGAATCTGCTATTGATGATCTCATATCAGTTGGGATGTGATATTTTCTGTCAAATGTGTGATTCCTCATTCTCTTGTTTCAGCCTGGATTTGAAATGCATAAGCATCCCTCTTACGAAGTCCATTACAGAGATTTCTGGTTTCCTCCTTATCAGTTTCTCTAAGAAAAACTATACACAAGTTAGAGCTTTCTTTCTAATGCTTATATTTTTTATCTTAGATTTGTCGTTCCTTGTCAACGTAACTTCACAGTGTTTCCTGAAGTTTCCTGTATCCCATTTCTGAAAGCTCTTTTTGGTTACTGATCATTTGTTGTCTGTGTGGTGTCTTGTGAACTTGTTCATCTATTAATTTAAGCTATTACAGGAACTAGATAGTGGGGCAATAATCAAATATATAAAATTGTATTAATCTTAGTGATAGAGTTTGTGTGGCAAATGTGGAGTGATATTTGACTAGTCTTTCTATTATATTCCACCCACATTGTGGATTAGTCCACAGCTGAAATTGATGTTACCGCACCATGTATGAACATTTTATCTTTTAGAAGTGTGAAAGTGATTAGCAAACTTGTTAGCATAAGATGGTCATCAAAGACTTCGGCCTTTAGCCCTTATGTTAAAAATAATTGATGTGATAGTTGCCAGGTGGCAAGTACACAGAACTAGTTTAGTTCTATTGCATCGCCTAATGATAAGCTTCTGGACCACAGATGTTGACTATGTCCAGCTATTATGATTAATAGGGGAGTGTTCTGAATAAACACTTCGATTTATGTAGTTTAATGAACAGACTCTGCATAATGTTTTCTGTCATTTATAAATTTCAGCGGATAGTTTCCTTCATTTTTGCTGTTCCTTCAAATACTCTTCTCCAATAGGATTGTATATGATACTCCTCCTTTGCGCTGTTCTTGCTTGTTTTGCTTTCTTGAGTACATCAGTAACATGTCCTATAATTTCCGAACCTACTAATTCAATGTGATATATAAGGGCTTTTGCTGGTTATTGTAGGATATGAATAAGGCTACTCGCCGGAGCGAGAATCCGGTTGAGGGCATGATTCCGTTTATGTGCAAGAGGGAGAGATCAGAGGAACCAGAGCCTCTCTCCTTAGGCCAAGCTAAAAGATCAAAGCACGAGGATCCAAACTCCAGCCATGCCAACGGTTCAGAAGATTCGACCACCACAAGTCAGGATCCCACCTTGATGACGACGACAAATTTTTCCTCCGCACTTTTGGAATTCCCCTTACTAGAATCTGTCGAACCTAAAGAAAGCCAGCCTTCCAACCCGCAGACTTTCGACTCTTCAAATCTCGAGAAGTCTGTGCCCCCCGGTTATTTAAAGTTTATCAGCAACTTGGAGAATGAGATACTAAATGTTTCCATGGAGAGAGAGACTCTGAAGATTGAAGTGATGCGTGCACAAGCGATGATCAACATCCTCCAATCGCGCATTGATCTTCTGAACAAGGAAAATGAGGACCTAAGGAGACTTGGACGAGGCGGCTAGATGTTTGAGTTAGCAAGCAGCATCAGTTTCTAGTTTTGTAGTTTTCGGCTAGTAACTGCTTCTATAACCTTTCTCCAGTGGCATCTTTTTCATCTTCAGAACAAAGAAGATGAGGTTAAAGAGCCATTTGAGATGTCCAGTAGTAGTTTGTGGCACCTTTGTAGTTGATATTGTAATCTGAACAAATGTTAGTAGCTCTGTTGACAGAACCTTGTCTTTCAACTTAATTTATCTAAAATAAAGTTCTGTTTCAATTCATGTGTTCTCTTGCAACAAAGTTGTATCAGAGAAACTGAGAATTCAGAGTTGC

>CrNAC-25

ATGGGTTTAAGGGACATTGGAGCAAGTTTGCCACCTGGTTTTAGATTCTATCCTAGTGATGAAGAGCTTGTTTGCCATTATCTTTACAAGAAAATTGCTAATGGAGGAGAAGTTTCTAAAGATACCTTAGTTGAAATTGATCTCCATACTTGTGAGCCATGGCAACTTCCTGGTATGTTTTGTCCCTCTCTCCCCATCTACCTACTAAAACGACGTGCTTCTACTGATGAACTAAACTACTATATTAAAGAAAACGACAAACTCTTCATCTCTTTCTCTTATAATTTTTTGAGGAACTTGATCCGTTCATATGTTCTGGGTGATTTGTTTATGTTTTATTATGTATATACGAATATTTCTATAAAAAAAGCTGAATATATGAATTTATTATTTTTTTCTGCTGAATTATATGAATTTATTATTATTATTATTATTCATATAATTTATGGGGATGTAATAGTGGTTCTCCATCTAATAATCAAGATGGAGAGAGGAAAAGGATAAAAATGAACAAAATCAGGAATTATTAATTATTTGCATAATAAATTTTAGAATAGTTCTAAATTACTTTTAGAGGCATGACGTTTAATATTGTCTATTCCTTTTTTGTTTTTCCTCCACCGGCATTTTAAATTTATGATTCTTAAAGTATGATTCTCTCTTGAAAACTAAGGGTGTATATGGTATATTAATAGAGTTTAAAATAAATATATTTTATTTTATTTTAAACCTTACATTTTCTCTTGAAAAACACTTTTTACAAAACACAAAAAAAAAAAAAAAAACTTTTCCAAACTTAATTCTTATAAAAAAATTATATTTTTTATTCATTAATATTTCGATACTACAAATGTACAGCCTAGCTTGGTTTGTTGCGTGTTTTTATTTCTTTGTAATGCTTAGTGAAAATAATAAGCTCGTAATATATAATCTAATTTCTATAGACTCTGCTAGTTGTCCGGTTTGGACTCTAAGCCTACTATGAGTGTATATCCAATGTTCGAGATTTGATAAATAAAATTTTTCGGGTTTTTGTTGTAAAAAAAAAATGTAAAAAATTGTTTTATGATCTCTGAGAGAAGTGGGAAAGCATATTTTCGTTTGTAGCTAGGTTTAAGCTTGGTGGAGAAGATTGTTGTTATAGTTGAACACATGAACTGCTTTGATCATGTGTGTATGGTCCTATCCTATTTGTTGCCATGCGGAAAAAAGGGTTCCCAAATTATTGCCAAGAATTATCGGAGCAAAAGGGACTAACAAACCATATAAGGAATTTTATAAGTCGTTTTCGGATAAATCCTACATTCGAAAAACTATTAAATCAATTATTAAGAAATTGAAGAAGATGGAATTAGAGTAGATACACACAAGTATGAATGAATTGCTAATTTCTGTCAAGAATCCATTGTCTTCAAATACAAATGTCTAGATTTTCTTCGAAGAAAGATGGGGTTAGAAGTAGAAAGTGGGACCAAATATTATTAGGAGATAATTATATGATAACATAATAAATTAATTTTTTAAAAATAATTACAATTAATTTGTGTATCGTAATATTGTTTCCTACATCAATAGAGGAGATGTGGTAAAACAGTCTAAAATTTTTAGTATGATATTTTTATCACTCAAGATCTTTTAATATACTTTTTAATACACGTTTTTTAATAGTAATTTTTTTCTATAATTTCTCTTTTATTTTATATAATTTATTAATAAACTTACTATTACTATAAGAAGAGTGTAGGAAAAAAGAGTATAGCAAAGACCGTGTGTATTAGACTATCCTCACCAGTATTGTCATCATTGAGCTAAACTCATAATAATTAATCTCCTTAAAACACATAATTATATGTTAATTATTTGATGAATTTTATTTAAATTTTTTTATTAATAAGTTATCTGATATGTTTATTGATTTTGTCTTTATTTGTATACTGTTGAGAATCCCACATCGACTAATTAAAGAAAATAATTACTCCTTATAAAGACTTGGGCAGTCCTCTCCTCTTAAAGTATCTTTTGGAGTGAGTTAGGCCCAAGCTCAAAATTTTCTAACATATACTATTATCTTTTTATATAAAGACAGATGATATATGATTTTCTCTAAAATTAAAGTTTGCTTTTCCGTAGTGTTTCTCCAAGTATGTTATTGTAGCATATATATATATATATTTCTATATAAATATATATGTATATTTCTATATATATGCATTTTTTCATTACCTTTTATATATATATATATATTATAAGAGAATTCTATAATCAAGAGAGAAGAATTGATTCTTCTTGTCGACAGCTACCAAGAGTAGTCCAATTAGCCTTGTCAAAAGTCTTCTTAGGCAGCATACAATACCTAATAACCTCTATATATATATGTGTGTATATATATATATGCACCCCATATATATCTATATATGTATATATAATATATAGTCTATATATGTATATATAATATATAGTCTTCATTAGGAATAAAAACTATATATAGTTCATGGTATACAGCTACTATAGCATGTGAGTGCGGTTATATTTTGCGTGCATGAGTTTCTGTTTTTTTTTTTTNNNNNNNNNNAAATTGATCCGTTTCTGTTTGAGAAATATATATAGTAGAGATGATCATAAGATGAGGTGTAAACTAAGCATCTCATAATACTATAAAATAAAAACATTCCATGACTTGTACGACTTTCAAACTTAAGAAATATATTTTGGAATAGTATTAAATTTGAAAAATATGTGTTTTTTTCTTGAATTTTCTTAAGATTTAAATTGTTTATAAAGGGAGATATTTACAATAAAATTGAAAAAAATGAAAAATAATTTAATTGAGAACTAATTATACATCAGGTTTAAAATCTCTATAATATCAAAAAAATATTGTAACTAAGCAGATTGACATGGTGAATAAGTGCATTGCCATTAATCTCATGCCATAGTATCTTTAATTAGATACTAGATACTTCATAGAGAGGTTAAAAAATATAGAAGCTTGCACTACCTCTGTTCCATATATTAATTTTCATATATTTAGGTTTCATATCATAAGTAAATTATCTCCAAAAATACTCATATTCAATTAGTATACCATTATTTGATTAATAATCTTAAAATCTAAATCTAATTCATATCTGATAAGATCTTAAAATATGGAAAGATGAAATAGAAGATAATGTCTTCAAATATGAAGTTTATTAAAATTAATATAAGGATATTAGAGGTATTTTATTTTAAAAACACATCTCAGACTTTTATAATTTGTATAAAATAGCAAATTGCATTATAGTACTATTATAATTAATTTTTGTTGTTATTAATTTTATCGTTTAACAGTTTGAAACTAATTTTCTTTATACGAATCCTTGTTCCTGAGTTAAGTTCTAATTTGGTGGTTTTCTGTTTATGAATAATTTGAATTTGCATGGATGGTGTAATTAATTAATTAATTAATTTTGACAGAGGTGGCAAAGCTAAATTCGACAGAGTGGTACTTCTTCAGCTTCCGTGACCGAAAGTATGCGACCGGGTTCCGAACAAACAGAGCGACAACGACCGGCTACTGGAAAGCCACCGGCAAAGATCGAACGGTGATCCATCCGGTAACACGTTCAGTAGTTGGAATGAGAAAGACATTAGTGTTCTACAAGAATAGAGCTCCTAATGGTATTAAAACCGGTTGGATCATGCATGAATTTCGCCTCGAAAACCCTCTTCTTCCTCCTAAGGTTCGTTCCTATATAAATATTAAATATACACCATCTATACCTATAATGTAA

>CrNAC-35

TCTCTCTTTGTTTGTTTCTCTCTCTAGCTAAGAGTGACAGTAATTTATAAGATGAGTAGTAATAATAATATTCTAAGTATGGTGGAGGCAAAATTGCCACCGGGATTTAGGTTTCATCCGAGAGATGAAGAGCTAATTTGTGATTACTTGATGAAGAAGGTGGTGGGTGGCGGCGGTGGTTGTGATGAAGATCAAGTGCAGCGATATCCAGGAGTACGTATGGTGGAAGTAGACCTCAACAAATCCGAACCTTGGGAAATTCCCGGTGAGTTCATCTTCCTTCTTCTTCACCAAATGTTTTAGAAGCCCTAATTATAATCTCTCTCATTAATTAATTTGTTTGATAAGAACTATGGTAATGTAGTTCCACATATGTCTAGTTTTGGGCGTGAAAAATATTAGTTTTTATATTCAAATTAGTTTTTAATTTCTGAATGAGTCAGATTTTGCAAAGAATTAGCGAGAACTATACATCATTAAATATGGACATTTTTTAAAAGTAGGTAAAAGATATTAATTTTGCACACTTATTAACTTCGTAAACGTATTAATTCCTTTTTTGAAGTATTTTTGGAGGTGGAATTTGATTCTTTTTATAGAGAGACTTTCTATATATGGATAAGGTTGAATGAATCTTTTGTGGTAAAATGTCTCCAATTCATATTTTTGGCACTGATTTTTCCATGAATTAAAAGAGAATCTTTCTTTTAATTTCTCTCCAGCTATATATGGAAAGCAACTATATATATATATATATATATAGTGGAATATTGGCCTGCCAAAATTCATTAATAAATACTATTTATATCATTAATCTTGCAAAACTAATTTATTAGGTCTAGATGAAATTGTATACACACATCATAATTAATTTACATGCTTTGGTTTTTCAAAAAGCACCAATAAATAGTGATAAAAAAAAGAAAAGAAAAAGAAGCACAAGTAAATATTATATAATTATGGTTCATAAAACTTGAAATAGTAGTAATTATCAGAAGAAGAACTTTTAAGCTCTTAATTTTGTCTCTGAGGTGAACTCTTTGGATTATGAATTGGGGAATATCCCCTTTTAGTCTATATTGACAATTTATAAAAGATCACACCATTTTTTGCATAAATATTTATACATCAAGAGAGTACTTGTTATGGCCTCCTCAAGATTAATCTCCACTTATATAGCAAGCTGATAATTTAGTTTTACAAGTATTTTTTACTATATATGTTAGGTCAGATGTCTCGATTCAAACACATAATATTTCTCGATGTAAAAACGAACACTCTATCTATTTATAATGATTTATTTAACACATATATCTAACATACACGCTTAATATGAGTTAATTTTTTATTTTAATATTACATTTTTCTTGAAATTTAATAATTAACTATATGTATAGAGTGTATATAATAAGAGCGAAGCCAAACAGTGACTTTTAGGGGTCAAAATTTTTATATAAAAATATCCTAATTTTTACACAAAATTAACACAATTTCATACGATAAATTATATATAATATATTTTATTTATACTGTCGTCTACACTTTATATATGCCTTCATCAATGTATATATACAAAAATATGTTGAATCATATATAGAGTTATATCTCAACCACTGGTTAGAAATTTCCAATCAAATTTCCTTTTTGAGGAAATTGACAGTCTTTTTGGCTTATCAATATTTCCAGAAGATGGCCACAATACTATACGATGGTGATTGAAAAATAACATTACTAATGCAAGTCTCTTCCATTCAATTTAAACCTTTGTCTCCATCAAACACTTCTAAAATTTTCTTCGTTTTTTTTAAATTCAATATCTGAGAGTCATCTACTAATACAAAAGAAATTGAAAAATTTTTAAATAACAGTAGTAAGTTGGCATATAATACTAAATCGCTATTTTAAGAACCGTAAGTGAGGAAAACTACACCTCTCTCGTCCTTTTCTTTCACCTTCACGAATAAAGCCATTCATTCTTTTTCATTCCGAAATTTTCTTTATAAATCAGCAGCTTTCTTGTAAATTGACTAGTGACTCTCCCATTAATTCTTCTATCCCATGGCCCCCCCAGGAAGGCAGGCGGCCCTGTGGAGAGCTTCTTCTCCATACTGGGCGTGGGGTGGGTCGGGGATTTTGACACTTTAATTTGATTAATTCCCATACCCATACCCATACCATACATAATAATAATAATTGGAGGATAAATTTGTAAAGGTCAGCTTTCGTATGTAGTGGCTCTCTTTCTCTCTCTCTCTGGACCGGCAACGTGTCGATTAATTAGCAGCTGCTTTTAATTTTCAAACCCTTGCATCATCCTATGATGTCAATACAGATTCTTCTCTTAATCATGAGATGAGATATATTCGTGAGATGATTAATTTGTTTTAGCTTTAATCAAAGAGTTCGAACTAGTATTTCAAAAATGAAGTTTTGTCTTGTGAGAGAGATTTTATCTTCCTTGTAGTCTTACCTACTGATTCGAATAGAAATCAGTGCAAGTTCACTCAGAATATCTCATAATCAAACAAAAAAACTCCAATTATGGATGTTATAGTTTTAATATTTTGGGAAAATGATCCTTTTGGCCTCTAAAATAAGACCAAAGTGATGAAATTATTGAGATTAAAAAAAAATGGATGAAATTGATCTAAGTTTATACTTTCCGTTCATTTTGTGTTTGATTCTCTATGGGGTCTTCCTTATTAAAATGTTGATGCAAATATTAATAAATAATGAATGAATGAATTGATAAAATGGGGATGTGGGCAGAGAGTGCATGTGTTGGAGGGAAGGAATGGTATTTTTACAGCCAGAGAGACAGGAAATATTCAAGCGGATTAAGGACAAACAGGGCGACTGCAACTGGTTACTGGAAAGCCACAGGTAAAGATAGAGCTGTTTTCCGTAAAGCTAAACTTGTTGGGATGAGGAAAACACTTGTTTTCTACCAAGGAAGAGCACCTAAGGGTAGAAAAACTGATTGGGTTATGCATGAATTTCGCCTTCAAGGATCATCCCTCTCCTCTTCCTCCTCCTCAATTCATCGAATCATCAAGGTAATCAATTCATTCCATTCATTCATTCTCATTATATATATTTAATTTATCATCTCAGTTGTGTTGTGTTGTGTTTGTATCCTATAAATTAGATTAATTATAAGAATTACAGGACTAAATATTCGATATAAACTCTGAGATATTCTGGTCAACTTTTAGTATCTTTGGAGTTAATAATGGTTCTTGTTTTTCTATGTAAATTCCTAAATATTATCTTACTGATTTAATTAATTTTATGTTCTAAATCTGACCAAGGATATTTTTGCAATAAGAAAATAATGTAGACGACATTTTCAAAGAATTGTTTTATTTGGTGGTAGTTGACTCTCCAAGATGATCAACACTTTTGAATCAATCATTATATTTATAAAAATGTAAATCATAATATTCGACTGTACTATATTTTTTTACTATTGCTTAATTAATTAAATTTTACCAAATTAAAAATTATTTTTAAAAAAAAAATATATATTATAAAAATAAATAGTATGAAGGATGGTACAGAATAAATAACATAAAGAATTTAGGTGCACAGTTAGGGTGACCCTTGACTAATTCGAATAAGATGATCTAAATAATAGTTGTAAAGTTTGATTCTTACTCATGATTAGAGTATGGCCTTCTGTGTTTCAAAATGCCACGAGAAAAATTCAAACATTTGACTCTAAACAGTCACATTTTGCTTATTAAATAAGTGAATCACCGAGTTTATTTTAAAGATATATTATATTAAGTATTTATTGAATAATTGCATGCAGGAGGAGGATTGGGTGTTATGCCGGGTATTCTTCAAGAACAGAGAAATTCTCCCAAAACAACAACTAGTCAACGGCGCCGTCGTCCACGGTGGTGACCACCATGATATTATCTGCTCGTCCCTGCCGCCATTAATGGATCCTTACATCAATTTTACCCAAACTCATAATAATAATAATAATAATAATAATGAAACGAACGAGCAAGTGCCCTGCTTCTCCATTTTCACTCCAGCTGATCAAATTAGCATTAGCAGTAGCAGCAGCAGCAGCTGCCAACCATTCTCATACCTCATGGCTTCCAATACGTCGACGTATGATCCAAACTTAATTTCCAATATTATGGTGCCTAACTTTGGTGGTAATTTACCCGAAGAATTTGGAGGGAATAAAACGGTGGTGGTAAGGGCAGTCCTAAATGAGCTCACCAAGATGGAACAAAACAACAACAACCTTAATAACCCATTAATTACAGCCGTTAAATCTTCTCCAAGCTTTGGGGAAGCTGCTGCTAGTTCTGATAGTTATTTATCTGAAGCTGCTCTCTCTACCATGTGGAACCAATATTAATTCACTTTTTTCTTTCTTTTTTTTTTTTTTT

>CrNAC-11

AACAATTTATCTTTTCCTTTTTGTTATCTAGGTTTTAGGGTTTTCTTTCAACATGTCAGATTCTTGAGGTTTTTAACATAGTAGTTGAAAAAGACCTTAAGCTAGGTAATTATAGAACAAATAAATTAGGGAATTAGGGGTTTAGAAATGGAAGGAGATCAAGTGAATAATAAAGGCGCTGACGAGACGCTGCCACCGGGGTTTAGGTTTCATCCGACGGATGAAGAGCTCATCACATATTATCTCATAAACAAGATCTCAGATTCAAGTTTTACAGGAAGAGCTGTTGCTGATGTTGATCTCAATAAATGTGAACCGTGGGATCTTCCAGGTACGTACTTGTAGTAATTAACCTTTAATTAGATCTTGTTAGTTAATTACCCTAGCTAGCTTTTAATTAATTCCTTGTCAATAAGTTCTTATAATTCAATAATCAAGATTTTTAGCTACTTTATTTAGGTTTTTTTTAAGTAATTAGAATTAATCAGCTTAGAGAAATTAGCTATTCATAATCACAAATAATTTTATTATTTATATTCATAAATTTTTATTTTATTTTTTATATACATAAATTATTTTTAGTTTTTGACAAATTAATAAGGCCACGCCGGAATCCGGCCAATTTTTGCTGATAGGTATGCGCTCTGTCATCCATGGCTTCAAAAATTGGTCGGATTCTGCTACAAATTTGCTAATTTATCAAGACTGTGAGGTTAAAAATAGTTTATATGTATCAAAAAAAAGAAAAGAAAAGTTTATGGGTAAAAGGTGAAAAATAAAAATTACTTATGAATATGAACAGCCAATTTTCTATCAATTAATTAAGCATTCATCAAATATTTAGGATCATCTATTACATCTTCTAATACACTCATTTTTATAATAATTTTATTAACAAAATCTTTGTAGAATAAAATATAAACTCTAATCTCTAAAAAATGAACCCTAAAGGAAAAGTTACTACTAAAGAAAGTATATTAGAAATTGCATTAGAATATATACTAGACGATGTGAGTATTCATTAGAAGACAAAATATTTTTTGTGTTATTATAAATTTTTTTTTTCTTTTTTTGTTGTATAATTTTTTTAAATTTATAATTAAACAAAATTCTTATCTAAGAAATTAATTAATTAATCTATGATATATATATAGTTATGTTCATATATTTCTTTGATGTAATAATAATATATTGATGGGAAATAATTAAATAATGAAAATAGGAAAGGCAAAGATGGGAGAAAAGGAGTGGTATTTCTTCAGCCTAAGGGATCGGAAATATCCAACGGGAGTGAGAACAAACCGGGCAACGAATACCGGCTACTGGAAGACGACCGGTAAAGACAAGGAAATATACAATAGTGTAAGTTCAGAATTGGTTGGGATGAAGAAAACATTGGTATTCTATAGAGGAAGAGCTCCAAGAGGTGAAAAAACCAATTGGGTTATGCATGAATATCGTATTCATGCTAAATCCTCCTATCGAACAACTAAGGTACGTACACTCTTTTTTCTTTTTCTTTTTTTTCCCCTTTATTTTTGGTTGGGTCAAAAATTATTTTACTTAATGTTAGAAAGATCGAATTTTTATATAAAATTCAGTACTAGTATACTATTTTTCAAAGTTCCATGTCTTTTTTTGAGTTTTTTTTTTTTTTTTCTGTAATTCTAACTCATCATGAATAATTTGGAAATTTCTCTAACCTTTTTTTTTTTTTAAATATTTATTTTTTAGAAAAGTTTGTAGCAAAACTTTTATAAGATCCAAGTTAAATAAAGTATGCATGTGACATTTCATTTCAAAGATCCTATAGGATATTTTGAAGGATTTAAAAAATTTACGTCTTTGGATCTTTTGATGCTCTTGTAAAAGAAAATTTTAAAATAAATAAGCTAAATTCACTTTTTCTTTTTCTCTTTTTCTGGAAAGTGGTTTTTTTTTAACATTCATTAAAAAAAATTAAATACAAATTTGAATATGTATACTTTTCCATTTTGCACTAATAAAATGGAAAATTTTATTTTTTCCTGCAGTAAAATCATAAAATTACAATTACGTAGTCTTTACCAAATAATTATAATTGAACTTATAGCATTTACTGAATAGAAAGATAAAGGGTAGGTAAGACATTATAAAATTGCCCTTCTATTTAGGTTTTAATTTCTTGTTATCCTTTGTATATATGACTGACTTCTTGACATGCACCATATTATATATAAGACTCTGTAAGTCCCTCCCTCTTGAAATTTTTCCACCAAAAAATATATACTTTAGGAAGAATTATCTCTAATTTCGGTTCAGAATCTATAAAAAAAAAAAAAAAAATGTTCAAAATACTGGGACAGAATAAACGTGAAGAAAAAAATGTTAAGAAATTTAGAAAAATATTACAGAAAATATTTATAAAGAATGTTAGATACGATCAAGAATGCATGAATATATATATATATATATATGTCCGATATATCTTTTGTAAGAAGGCGTTTTATAGAATATAAATTGGATTAATGAATGCAGCAGGACGAATGGGTGGTTTGCCGAGTTTTCCAGAAGAGCGCTGGAGGAAAAAAATTCCCATCATCAAACCATTCAAGAGCAGCAGCACTACTTAATGCTAATTACAACATTAATATTGATCATCATCATCAAATCAACCAAAATTCAATTTCATCATCATCATCATCTCATCATCATCATCATCCTAATTTTCAATTTAACAATATTATGGGAATTAGAAACTACATGATGATGAACCATGCAGATATTCAAGAACAACAACTATCTTCCTCTTCCTCTAGGGTTTTGCCAATTCATCATCAATCCCAAATGATGAACTATGCTGCTCATCATATGTTGGCTACTTCTGGATCTGGTGGTGGAGGAGGTTGCTTTACCATATCTGGGCTGAATCTTAATCTGAACGGCGCCACCACCACCACCACCACCACCACGGCACAACAACAAGATGTTACTAATAATATTAATGGTGTTATTGGGAATAATGAGGAGGTGGTGGTTGGATATGGCACGGCGGATGGAGAGATGATGATGATGATGAACAATAATGCAATGGGAAATAGATTTCATGTGCCGCCGATGCCCATGGATCACTCATGTGCTGATTTAGACACTTACTGGCCTCCCTATTGA

>CrNAC-13

AATGGCGTCGGTCTATACTCTCAATTAAAGTTTTTCTTTTAAAAAAAGAAAGGAGGAAAAAATGCAGTAATTTACATAAACGTTGGACTTGGATTTTTCTTTGTTTCGATTCAATTTCTTTATCTCTCAAGATTTCTTGGTACTTCTCAAAAACCCTAGGATTGGATAATTTGGGGAATGATCTCAAAAGGGTCGTCGTCTTTAGCGCCGGGTTTCAGATTTCACCCGACTGATGAGGAGCTTGTTCGATACTATTTGAGGCGCAAGATTTGTTCGAAGCCTTTTCGTTTCGATGCAATTTCTGAGATCGATATTTACAAGGCTGAGCCTTGGGACCTTCCAGGTTTTTCTTTCAACTGGCCTTGCTTTTTGGGGTTTTAGTTTATGGGTTTGTGGAAATTTTATCAAGTTTAGTTTGATTGGTTGTTGGGATTTTCTTTTCTTGGTGAATCTATGAAGCGTTTATGTGTGTAATTCATTATTTTAAACTTGGGTATGAGAAAATTTGATCTTTTTTCTGTTCTTGGTTCGGGGGTATTGTGGCTTAGTGTTTTCCTTGGATCTGGGACATTTAGTTAGTGGGTTTATCTGTTTAAGGTAAGGGCTTTACTAATTTTCCTTTTTTAGATGAAAAGGGAGTATGTTTTGCAAAATGAATTATGGGCTTTTAGCTTTACTAATATGGGGTAAGTTTAATTTGGAATTTCAAACAATTTTCCTGACTCTTATTTCCAAGAAAATATGCATCCCTTTAAATTCAACTGCGTTTCTGTAGTTTTGTATGTATTACGGAAATGAGCTGAAAATATTGTTGGACAGACACATTTATTTACACGTGGAGGCATGTTAAACCAGGTCTGCACATTTGCACCCGCCAGGATATGAAGCTTCATCTGCTAGATAGTATTAGACATTACCCAACAATCTTCATTTTCACTTCAAGGCAATGCCTTATTTCCAAGTAAATATGCATCCCTTTAAATTCAACTGCGTCTGTGTAGTTTTGTATGTATTACGGAAATGAGCTGAAAATATTGTTGGACAGACACATTTATTTACACGTGGAGGCATGTTAAACCAGGTCTACACATTTGCACCCGCCAGGATATGAAGCTTCATCTGCTAGATAGTATTAGACATTACCCAACAATTTTCATTTTCACCTCAAGGCAATGCCTTACCACTTCTATTTTGTTGTGATGTATGTTTGTTGGTTAGAGTTTTGGCATTTTATAATATTACCTTAGACACGCAACTTTGGTTGACTTTTTGGTTTTTCAGTTGATTACTCATTATACTTTCTTCAATCATGTGACTTTAGGTATGTCAAAGCTGAAGACCAGGGACCTGGAATGGTATTTTTTCAGTGTGCTTGATAAGAAGTATGGTAATGGATCCCGTACCAACAGAGCTACAGATAGAGGCTACTGGAAAACAACTGGAAAAGACAGGCCTGTATACCACAAGTCCCAGGTTGTGGGGATGAAGAAAACCCTGGTTTATCACAGTGGTCGAGCTCCAAAGGGTCAGAGAACTAACTGGGTGATGCATGAATACAGACTTATTGATGAAGAGTTGGGAAAGGCTGGAATTTCTCAGGTACACAAGTGTGTATGGATTATTCTTTGACGTCTGCCTATTTGTTCTTTCAGCACTCCTTGCAATTACCATAAAAAGAGTGTGGAGGTTTATAGAGATATAGGTCTATGAGGAAGGAAACTTATCCCTTCCAACCATGATGATTATGTTATACAAGTCTTTGACTTATTAAGAGATATTTGGATACCTTGATGATTCGTAAGTGAAGTAGTGTATTGTGGTCACTATATTTTCTTGCTATGAACAATTCTGATTTTTCTTTGCTTTTCTATCCCCCTAATTCCTGCTTTGTTTCCAAATGAAGGATGCCTTTGTGTTGTGCCGAGTTTTTCAGAAAAGTGGTTCTGGTCCAAAGAATGGGGAACAGTATGGGGCACCCTTTGTGGAGGAAGAATGGGAAGAAGATGAATTGGAAATATTGCCCAAGGACGAGGCTGCAGATGAAGTTGAAGTTGGTGATGATGCCTATTTGGATGGAGATGACCTCAAGCAGGTGTGTGCTTTATTTTGCTTTGGTGTTCTGTACTTGAATATCTGCTACTCTACCCCCCAGAAAAAGGATGTTTTCATGTGGAAATATGAACATGATTAGCCAGAAAATTTACTGCATTGAGTTAGATAAATTGTTAAGAAATGGAAGTAGTCTTTTCTATGAGGTTTTTCCTACATATGCAAGTGTCGGGTAAGCTTGTGTTATTGTTGGCTATGACAAGTGCAATGTCTTCGTTGTCTTAGTTGTTAACAAATGGAAGTAGTCTTTTCTATGAGGTTTTCCTACATTATGCAAGTGTTTGGGTAGGCTGATGTTATTGTCGGCCATGATCAGTGAAACGTCTTTGCGGTCCTAATGAATTTAATTTGTTTCTGGGGCTAATGTATTTGATGCCACTATAAGATATTTGTCAAACCCCTTTTCTTCTGGGTGGAAACTTTGGACTTACCTTGGTCACGTAAAATTACTACTCAAAGGAAACTGTCCAAAAAACCTTCAAGGGCAGGATACAGCAGTCAATAGCTAGAACATATACTTACTTTTAGAATGTTTTTTCTGCCAGATTCTTGCTGCAGATATACAAACAGATAATGTTTCTTTTACTCCGAATTGCTATACTGGGGAAGATGCTGCTTTTGTTGAGGAAGCTACAGAGTCCATTGGCGATTCACAGAAGCTATCAGTTGGCGCTGGGGAATATGATAATGGAAATAACCAATCTGATGACCAGAATGCTTATCATTTGCCAGTTCAGTATGATATGTATCAAAAAGCAGTCAAGCATGAATACATTGGTGAATCAAGCAAGACTGCTGATCCCCAGGGTGTAGACTATTTGCTTGAGGAACCATTTGTGGATGCTTCTGACAATCTTCAGTTTGGTGATGGAGGATTCCTTGAAACTAATGACCTCTCTAATCCAATTGATGCCGATGCTGCAGGTTTTGATATGCTCGAGGAGTATCTCACATTTTTGGATGCAGATGGTGATATTGCATTTGACCCTCTAATTATGGAAGGAGGTCAGAACAATATTTCTGGAGAATCATCTGCCGCAGAAAAGGTGAAATAAGTTGCATTTTATGTCATGGCTGACCTGTTAGGATGCCAAATTCGTCATTTGACATCCTTTTTTTTTTCTTGTCTTTCCTTGTCCCAGGACGTTGAAGAAGGAATTGAGACAGGCATGCCAAGCAAACAGCTTGAACATGATAATGATGCTACATCTTCCAAGGAGGCTTCTTCAAAATTTGGATCAGGTACTTGTAAGATGAAATCTATTTTGGTATATGGTTTGTGTTTAGTGGAGGTTAGTTATCTTAATTATTCTCCTTTTTATGTTTCAGGTTATCAATATCCATTCATGAAGCAGGCGAGCCAAATGTTAGGCAGCTTTCCTGCTCCTCCTGCATTTGCATCCGAGTTCCCATCAAAGGATGTTGCTCTGCGTTTGAATTCTGCCTCTCAGCCTTCGAGTTCAGTTCATGTTACTGCTGGCATGATAAGAATTAGGAATATGAGCATGGACGGGCATGGAACAACAGACTGGTTCTTAGGAAAGCACAGTCAGTACAACGTGACCCTTTCTTTTGGCGTATCAGGAGGTGATGATAACTCTACAACCTTAGAATCAGCTGTCAGAATACTTCCAGGAAAGGCAGTCTCCCCAACACCCATCGGTTGGTTCTGCTTTATATTTTTTTGGGTCCTACTCCTTTCCGTTAGCTACAAGATTGGAACCTTAATTTGTGCTCAGTAACTCTCCATTAAGTGAGAAGATTGAAGAACTGGAAATAGCCATAGAAAACAAACATTTTTTTTTTTTGTATGTTTAGAATTGACTTACTGAACATAGAACTAAGTGGAGAGATTTAGCTGAAGTATATTTACATCAAGCGTTCTCCCTTTTGGGGATTAAGGAACAGTGGTTATGAAAAAAGCTTCTAGCTTGGAAGCTTTGGCATGGCGTATTTTGATGATGTCTGGCTTTCTTTAGTTTATTTGTTTGAATAACCAAACAATCATCTTGTATAATTGCTTAAAAGGAAGGAGCTATATACTTGCAACAATTAAGGAAACATCTGTCGTTGGTGACCTCTTACTGTTGCCGACCAGTGATATAATCATTGGTTCATTCCAGCTCAATTCCCCTTCCTCAAGCAACCATGGTGGATAGAGTTTAGTTTGCTGATAGATAGATATTCGTTTAAAGTTGTGTCAAAGTTGCATTATATTTGAAATGAGTTACACTAAAGTTGTAGCATCTGCGAACAGAGAGAGTTACAGTGTTATTTGTGAAATGAGATGGATGTAAAGTTACAGCACAATGTTTGTAAAATGAGTCGAGATATGAACGCATTGGTCTGAGCAGTTCTTAAATTATTC

>CrNAC-10

ATGGAGAATTATTCAGGTCTTATTAATGATGATGATGATCAGCTGGAATTGCCACCGGGTTTTCGATTTCATCCAACTGATGAAGAATTGATCACTCATTATCTGTCAAAAAAGGTTCTTGAAACCAATTTTTCTGCTAAAGCTATTGGTGAAGTGGATATGAACAAAGTGGAACCTTGGGATTTACCTTGTAAGTTTTTCAATCCAAATAAAAAAAAGGGTTCATTTCAAAATTTGATTTTGATTAATTAATTTTGATGGGGTTTTTTTTTTTTCTTTCTTGATTTAGGGAGAGCAAAAATGGGGGAAAAAGAATGGTATTTTTTCTGTGTTAGAGACAAAAAGTACCCAACTGGTTTAAGAACAAACAGGGCTACTGCTGCTGGTTATTGGAAAGCTACTGGAAAAGATAAAGAAATTTTCCGTGGAAAATCTCTTGTTGGGATGAAGAAAACTCTTGTTTTTTACAAAGGAAGAGCTCCTAAAGGGGAAAAAACCAATTGGGTTACTCATGAATATCGATTAGAAGGAAAATTTTCCCTTCTTAATCTCCCCAAATCTGCTAAGGTAAGAGATTCTTCCCTGTCTTTTTTTCCTCTGTTTTTTTTTTTTCTCTCTGTTTCAAGAATAAAGATTTTTTTTTGTTGTTTTTTGCAGAATGAATGGGTGATTTGCAGGGTTTTTCAAAAGACTGCTGGTGGAAAAAAAGTTCATATTTCTGGATTAGCAAGATCAAATTCATTTGGGAATAATGATTTGGTTTCTTCTTTGCTTCCGCCATTGATGGATTCTTCGCCTTATAACAATGGAAATTCAACAAAATCCACCCCCGTGGCGGCGGAATCAGGTCACGTGCACTGCTTCTCCAATTCAATTACTGCTCAAAAGAGCCAACAAGAAGAAATCTTCAATTATTTCAACAACAACAACAACAACAATCCATTTGTTGTTTCTTCAACAAATGGAGGATATTCTTCTTCCTTCACAAATGGTGGATATTCTTCTTGGGGAGGTCAACAACTTCTTGTTCCTCCTCCTTCTATTGTTCAAGGAAATTACCAATTTTCACCTTCATTTCAAATGCAAGATCCTTCAATTCTGAGGAGTTTATTGGAAAATTGTGGACAAAACATGATGAAACAAGGGTTCAAAATAGAGAAAGATGGAATTAGTGGATCACAAGAAACAGGGGTTAGTACTGATATTAATACTGAAATTTCTTCAGTTGTTTCAAATCTTGAAATGGGAAGAAAATCATCCCTTGAAGATCATCAAGAAGTACCACCACCATCCTCATCATCTGCTGCTGCTGTTGTTGGACCTCAAGATCTTGATTGTCTTTGGGGTTATTGAAAAAGAAAATCATCAATCCTTTGATTGATTGGAAATAAATAGAGAGATTAGAAGAAGAATGAAGAAGGAAATTATCATTGATAGTATTATAATTGTCTGAATTGATCATATATTATTTGTATAAAGTTCAAGATAATTGAGGGATTTTATGCAATTACATACAACTATTTTGAATATTATTAACTTTTTTTTGGTCTTGTAATTGTTTACATAGTGTTTGGATTTGAAATTTTTTTACTAAATTTTAGTAAAAGATTATATTATTTGTCAAAAT

>CrNAC-05

AATACTTTTTTTTCTGTTAGAAAATAGATATAACCTTCTCTCCCTTCTCCTTCTCCTCCTCCTCCACCCCCTTCCTTCTTCACACTATCATAAAATTCCTCTCTTTCCTCTGTTTTTTCTTCATTTGTTTCTTCCTTTCAAGAAAAATTCATCTGGGATTTGGAATTTCTATTTCAAAAATGGAAAGAGATGAAAAGATGGATTTGCCACCTGGTTTTCGCTTTCATCCAACTGATGAAGAGCTAATAACTCATTATTTATCCCCAAAAGTTCTTGATAATAGTTTTTCTGCAATTGCAATTGGGGAAGTTGACTTAAATAAAGTTGAGCCATGGGATTTGCCATGTAAGGTTTTTTTCTTCTTCTTCTTCTTTTGTTTTTATGGATTCTATGTGTTTCTTTTTTGTTCGGGGAGGGGACAAGGTCCCAAACAGCCATACAAACAGCCATACAAGGCTGACGGACATATAATAGTAATTTATTCTTGAATCGCTCGAATTCAAAACTTCTTACTTGACATCTTTTATGTTTTTCTTTTGTACTGAAAAAAAAAAAAAAAAGAAAAATTGATGTGGTTTTTGTTGCAGGGAAGGCAAAAATGGGTGAAAAAGAATGGTATTTTTTCTGTGTTAAGGATAGAAAATATCCAACAGGATTAAGGACTAATAGAGCTACTGATGCTGGTTATTGGAAAGCAACAGGAAAAGATAAGGAGATTTTCAAAGTGAAATCACTTGTTGGGATGAAGAAAACTTTGGTTTTTTACAGAGGTAGAGCTCCCAAAGGAGAAAAAACTAATTGGGTTATGCATGAATATAGATTGGAAGGTCAAAATTCCATCCACAATTTGTCTAAAGTTGCTAAGGTAATTGAACAATCCTCTGTTTTCATCCCCTGTTTTTCTTTTCAAATGCCTGAATCCTCTGTTTTCCTCTGTTGTAAATAGTTATGAATTATCATTTTTTTTTAAAGAATTTAGAATATTTATTACTTTCATTCCTGCATTTTTAGAAGTAATTTGTATGAACATCTTTAACCATATTCTTTATTGTTTACAATATCTCGTTTTTAGCTTATTTTTAACTATAACATATTTTTGATCTATTCACAATTAACTAGGACCTATTCTCTATTGGTTTTGCTTTTTTTTTAAGCATTTAAAATTAAGTATTTTTGAAAAAATTGTTTTAAAAGAATTATTTCAATATTTTTAATTTTAATAGAATCAGCAATTAAACATAATTTTAAAAAGCATCCATTGGATAATTTTTCAAAAATAACTTTTTCAACCTATTTTAAGCTTAAAATAGTTAAGGACGGTTAAAAAATAACTTATAAACTAAAAGAAAAAACAGGACCGATGTTTTTTTTTTTTTTCTAAAAGTACTCTTTTACTATTAATATTGAACAGATCTTTTAACAAATATTTAGTAATTAAACCAAAAAACACCTTAAAGAAAAATATTATGCACCCCTCAAAATTCTTTTATCTCACTTCTTATTTCTTCTCGTGTAATTGTTACCTTTTATAAGTTAGTTTATCTTGGAATATCCGTTTTCTTGAACATTCTCTTCGTCCATAAGTAATTTATTTCTTTTTCTTTTTTCTTTTTTCAGAATGAATGGGTGATATGTAGAATCTTCAAGAAAAGTACAGGAGGAAAGAAAGTTCATATTTCAGGGTTAATAAAGATGAACAATTATGGTGATCAAGAAGATTTAGGAACTTCAAATTTGCCTCCATTAATGGATTTATCCTCTGAAGAAACCCAACCAAGAACGACAACAACAATTGCTGAGGCATCTCACGTGACCTGCTTCTCCAACCAAATGGAGGACGGCCAAAAACCTCAAATGCCTGCTTCTTCAATTCTTTCTTCACAAATAATGCCTTACTTAGAAAGTATGCAGTTTGGGGATTCAATTTCCATGGATGATCACTCAATAATGAGGCTTTTAATGGACGAAAATGGAGGAAATTCAAGACAAAATCCTAATGCTAATAATAATCAAGAATTCTCCCAAGAAACAGGAATTAGTACCGACATTTCATCCCCTGTTTCGAACAGGGTAGAATGGAGGAGGCCTAGTTATGGGCATCATCAAGAATTTCATCAGATTAGTTCTGCAGGGCCAGTTGATCTTGATTGTTTATGGAATTATTGAACTTTTTAGGATTAAAGAGTAGAGAGATAAGTAGGGATTGGATTAAGCAAnAAAAAAAAAAAAAAAAAGATGTTTGATTGGTTCCTTAATATTTTAATTTTTGGGTCAAAATTGTTCTTTAGAGAACAAAGTTTGTGTACATATTCCAAAGCCAATTATTGTATGTGCAACAATAGAATTGAAAAATTGATGACGACCCAATTCAATATGTTTATATCAATACTAGTTATCCAACTCAAGTAATGAGAAGAATA

>CrNAC-22

ATGGCTTCATCAAACGGCGGCGTTCCACCAGGATTCCGCTTTCACCCCACGGATGAAGAGTTGCTTCATTACTACTTGAAGAAGAAAGTTTCCTTTCAAAAGTTTGACATGGAAGTTATTAGAGAAGTGGATTTGAACAAGATTGAGCCATGGGAATTGCAAGGTAACTCAACCTAGCCTACCTCATCCTTCAAGACTAGCTATTTCTCTCCAAGAAATTATTATTTTTTAATCTAAGATTTTTATTTTTATTTTTGTAAGAAGTTAATTAAAATTGTTTATTCAACTATCTATGTTTTGCTAACTTAAAGATTAATATTTTTTTGTCCCCGTTTATGTTCATCTATTATTTTCATATACCTGAAAATATTTTCGTGGAAGCCAGTGTTTTTTTACGAAGCTATTCAAGTAATGTGGACATGAGCTAAACTAGCTAAAGGGATACAATTGACATAACACTACAATCTTGATCTTGATGACATCTAGACCTAGAAAAATCTAGGCTTTTAGTTAGGCTTAAGTACTTATTTGGGTTGGAAGGAAGAAAATATAGTGTTATGCTAATTTTACGGATTTATTTACAGCTCATGTGCAAATCACACTAGTTGAGCAGCTCTATCATCCGATACTAACTTTAGTGTATTAGGACATTTTCAATCATACGTAAATAATACTTCAATCATGCATAAATAGTATAAGGATGTAAATAGCGATGAAAAAAATATTAATCCACCAGTTGACTAAACATTAATCAGTTGAAGGAAATTTTGCTACTTTCTCTTTTTTTTAAATTAAGATATATATTAGCCTTAACAAGATTACACATAGTGCTTCTCTACTTACTATTTATTTAATTATTGAACTTTAATTAAATTTTTTTATACTGGCCAACAATTGATCAAGGTGAGTTGGGAGTTTTTTTATACTGTTTTATTTTCAAACTCAAAGTAAAATTTCAATTTTAAAACACATATTTCATCTTAAAAACAACTTCAATTTTTATAAAAAAAGAAAAAAAAAAAAACCTTCCAAAAATTTTGTGAAAAAAAACCCTATTGACATATACATGATATACATCAACATTTTGTTCAATAATTAGGATGTGATTAATCAATGTGCATTTATATATTATACATACATTATAATATTCTTCAAATTAATTCCATGCAGAGAGATGCAAGATTGGAACCACACCACAGAACGAATGGTACTTCTTCAGTCACAAGGATAGAAAGTATCCAACAGGGTCAAGAACAAATAGAGCAACAAATGCAGGGTTTTGGAAGGCAACAGGAAGAGACAAATGCATTAGGAACACATTCAAAAAGATTGGTATGAGAAAAACATTAGTTTTCTATAGAGGAAGAGCTCCTCATGGCCAAAAAACTGATTGGATTATGCATGAATATCGACTTGAAGATGCCGATAATAATGACCCCCAAGGAAATTCCAGTGTAAGCACCTAGCCTTTTTTTTTTTTTTTTCCATGCACCATGGCTAAATAATTGGAAATTAAAATAAAATATTAAGTAATATTGTACATATAGTACTCTTCTGTTGTTTTAAAATACTTATATAAATATTTTTAAATTGTAAACAAAACGAATTTTGAAGAAAAACAAAAATTATGATATATAAGGACTTCATTTCCTTAATATACATAAGATAAAAAAGGAATGTTGAAATAAAGAAACATGGATAAAGTTTGAAAACACAAAATATATATTTTAAAAAGTATGAAAAATAAATATCCTCTAACTATTTTTTATTATTCAAAGATAGTCGTTTTTCATTTTTTGCCAAACATACCCACCTCTAGTAAGGATAAAATGACAATGAAACACTTTCCCTCCAAAATTCATAGCGCATGTTAGCTAGCCAAAATGGTTGTTTCAATTTGTTGTGTGTGAATTTTTTGAGGAAAAGAAGTTATTGCAAATTTTACCATTTTAATAGGCACATATATATTTCAAATGTTTTTTCTACCCAGAAACCACTGTTTAAGGTGAGCTTTGTGCTAATAAAGGTATAAAAGAATTTATTGTGATTTTGCAAAAATTAAAAAGTACCCTTTCTATTTTATATTATAATTATTTGGATATACTTTTTTCTTTTCAAAATATGTGTCACTTTCGCAAATCAATAGAGTTTTAAATGATTTTTTTCACATATATCCCTATTTTATTAGAATTGTCTAAGAAATTTTCATTAGAACTAGTTAACAAAGATTAATGCATAATTTAATCTATTTACCATATATACAATCTAATTATTTCCTTAATTCGTATGTAAATCATTCTAAGATGATATATAGTATGAAAATATAATATGAGATAAAGGTAATATATTTTGAAAAGTTATTTCGAAAAGGTTATTCTGGTCCAACAGACATTATGCTAATAAAAGGAATAGAATGCCCACATAGGGTTGCAGAGTTAGTAAGAATAAAAGCGTCAAGGATTGTCTAGTACATCTTTTAGTATATTTTTCAATACACACTTTTAATAGTAATTTTATTAATAAAAATTTTATAAAATAAAAAATAAATCTTAAACTTTAAAAGAAAAATTTTAAGAGAAAGTTACCATTCAAATAAAATAAATATATTAGAGAGTGTATAATGAAAAAAGGGATAGAATCCTAATGTTTGACTTGATTAATGGGCGTTAAATTCCTCCCATACAAAGTCAAAGTAAATCAATATCCCCATAACTGAGTTTCACGCTTTCAGCTTTTCTGATATTCTTTGACTATTTACCACTTACTAAGGAGTACTACTAGGATCATTCTTGGGGACTTAAAAAACATTTTTCTCCATCATCATGTAATAAAAAAGAAGTTTAGAAGGGACCAAACTCCATCAAATCTTTTATTATCTACATAGAATATATGAATAATAAGCAAAAAAAATTTTGGAAATCATGGCTTCTTTATATCTCTTCTAAGCTCAATTTCTATTCAAAATCGTAAAGTATATATTCTAATTACACCATTTACTAAACTTTCATTAAGAATAATTCTTCTTTTAAACTTTAAATTTATTTTTCATTTTATTAATTAATATTAATAAAACTGTGATAAAAAGTATTAGACAGTTTTTTTTTTTTTTTGAAATACTAAACAATATTTTACAACATGTATTAAACAATCTTAAATAGTACAAGGACCGTACATATTGATCATTTGATCATTTGACTTTTTTTTTTTTTTTTATAAATATGATAATTCTATGTTCATTGCATGTTGTTGTATAGGAGGACGGTTGGGTAGTTTGCCGTGTATTCAAGAAAAAGAACTTATTCAAAGTCGGAAACGACGGAGGAGGAGGCACCGCCGGCGGCAGTATTCATATAGGATCCGACCCGCTTAACCATACTTCCTCCCGTGCCACATTCATGCCAAGGGACAATCAATATTTATTACATCCTCAACAACATCATCATCATGGACTCAATTATTCTCACAATATCCCAAACATAACTCTCCCACCTCATTATTCCCAAATTCAACCACAAAATTTCATACCAACACATAATAACAAGCCATTAGATTATCATGACTTTTCGGAACAGTCACCTATAATGGTCAAACAACTCATGGCTGCAAGCAGTACGTGTGAGTCATCGGCTAATTTGGGAGTAACAGCAGCAACTGAAAATTGTAATGAAGAACAACAACATGAAAATCATTTAAATGAATGGGGAATGATGGATGGACTTGTTACTTCTTCTTCACAAGTAAATATAGGAGGAGGAGGGAATATTAATCCCAACGATGAATCTTCTCACATTAATCAACTCTCTCTACGTAGCGAGATGGATTTCTGGGCTTATGGCAAATAA

>CrNAC-08

ATGAATACATTTTCACATGTACCACCTGGCTTTAGGTTTCATCCTACAGATGAAGAATTAGTTGATTACTATCTTCGAAAGAAAATCGCTGCCAAAAGAATCGATCTTGATGTTATTAAAGATGTCGATCTTTACAAAATCGAACCATGGGATCTTCAAGGTTAGTAACGTACTAATTAATTCTCTAATTATTTCTTTTGGGGTTAATATATATATATATATTAATTTTATTTTTGGTTGGTATTTTAATGGTGAGGCATGCAGAACTATGCAAGATAAGTAACGATGAGCAAAACGAATGGTATTTCTTTAGTCATAAAGATAAGAAGTATCCAACAGGAACAAGGACTAATAGAGCTACTAAAGCTGGTTTCTGGAAAGCAACTGGAAGAGACAAAGCCATTTATTCTAAGCATAGCTTAATTGGTATGAGAAAAACTTTGGTTTTTTATAAAGGTCGTGCCCCTAATGGACTTAAGTCTGACTGGATCATGCATGAGTACAGACTTGAAACTAATGAGAATGGCACTCCTCAGGCAAGCCCCCCCTACCTCCATCTCCTTATTTATTATGTACATGTTGAATATATTTTTTTTCGTTTCAAAATATACGTTCGCGTTAGTTAAATATATCCTTATGTACTGTTATTGGAATGATATAATCTAATAAATTTACATTCCCAAGTAAGGACAATTTAATCTATTCAACATTTCGATTAAAATAATTTAATCATTTTTTTAATAATTTCGTCATATTCTAATTAGGTGACACATAAATTGTAATTGAGGGAGTAATTAATTAAAACATATGAAGCGTGACATTGTAGCTATAACTAATTAATTTATTATCATATCCAATTAGAAAATTAAATTGCCAAAGAATATGATAGTATTCGATATTTTATATCTTGTGTTGATAATTTTAGATTATTTTTCTCTTGAGAAATTTTATAATGTCACTGCAGAAGGAATTGAAGTAGGAAAAAAGTTTCATAAAGATGTGAATGGAAAGCCCTATCTATAAAACAATGCAGTTCTGGATAAACCCTTTTGATATTAATTAGTCACATTTTAGGGTTTAATTGTTTGATCACTCCTGCTCTTAACTATTTATTTAATTGATGAAAATAAGTGCTTATATTTAGGATTTTATCCCTGAATTTGCATTCTAGTTATTTGCCTATTTTTTTTCATTATCGCATTTAAATAGTGACAAGATATGCAGTAGCTTAATAGTAAAAAACGCTCTTTATCAGATATTATGTATTCTGATCACATCCTCTCCTCTCTCCTCTGAACGTATGATCATATTATATAACATATTCGTTTCGTTATTTATCAGGAAGAAGGATGGGTAGTGTGTAGGGTGTTCAAGAAGAGGCTGGCAGCTATAAGGAAAGATGGAGAACATGAACAAATGTGTTGGTATGAGGATCAAGTTTCTTTCATGCCTGATTTCGATTCTCCAAGGCGAATATCTCAATCGCCATATGCTTCATACAATAA

>CrNAC-27

ATGATGAGTGCTGGTGCAGCTGTGATGAACAGTCAGCTTTCTGTTCCTCCGGGATTTCGATTTCACCCTACTGATGAGGAGTTACTTTATTATTATCTGAGGAAGAAGGTCTCTTACGAGCCCATAGATTTGGACGTTATCAGAGAATTAGATCTAAACAAACTCGAACCTTGGGATCTAAAAGGTCAGCCATACCTAACCATATATATATATATATATATAAAATTATGTGATAATATAGTTAAAATCCCTTCAACTTTTTATGTTTTTTTAATTTAGAAATTTGTTCCGTTTGTCTAATTCTTTTAACACTTTATAAGGGTGTTTATTTACACTCTAATGAATAAAATTACGTTGACAAAAGGGACGTCAACTGCCGTCCTCTTTTTGTTTGAAAATTAATATAAAGTTACTATAGTAAAAACCGAATTTTGAAAAGAAAATGAGCAAATCAACGAATTTTGTTTATTTAGTTAGAAAATTGTGAAAAATAATTTATATTTACCATTTTTTTTATTAAATTTAGAATTGTTTGAACAGTCTTTTTACTTGAAAATATCCATTATATATACGAAATTTTACAGGAATTTGTAGTGTAATTTCCACTCCCATCCTTTTAATTTTTTGGCATTAAAAAAAACCTTATGATTAGTCAGAAAATTGTGAAAAATGATTGATATTTACCATTTTTTTGTTAAATTTAGAATTGTTTGAAAAATCTTTACGCTTGAAAATTTTCACTATACATACAAAATTTTAGCAGAATTTGTAGTATAATTTCGGCTCCCATCTTTTTAATCCTAATTCTTTCCCAGTTATGTGATAATTTCCACAAAAGATACTATAAACATGCACATTCGGCATTAAAAAAAAATAAATACTCATTATTAGTTAGAAAATTATGAAAAACGATTGATATTTACTATTTTTTTTGTTAAAATTTAAATTTGTAATAATTTTCACAAAAAGACGCTATAAACCTACACATTTGGTATTTTTTGGCATTTAAAAAACGTTATATAACTTAAATTGTATAAGTGGCAAAAAAATATAATAGATAATATGTGATCATTTCCACATTATTAATTTATGTTGCTACTATTATATTAATTTTTACATTTAAGCCCAATTCTTTTTTCAATTTAGCCCCCACAAAATGTAGGGGCTGATTTATAAAAAAAAATTAAAAAAAAAAACCTCGGCTGCGCCAGCAGAAACAATAGAAAAAATTTCAATTTTCTTCTTCTCTCTCCCAAATATTATATGTAAATTTAGAATTTGTAATGTTTATCCCGTGAGTGATGAGTGATCACTCCTCATTGGTAATNNNNNNNNNNNNNNTCTTTTGCTATATTATATGTAAATTTGGAATTGTTTGAGTGTCTTTTGTACATGGAATGCATCATTTTATAGACGAAATTCTGACAAAATTTTTTTTCCTCCAGCCCCCAATTATATTGTTTTGGTTCCGTCCCTGTTTGTTAATAATTAATTTGTTCATATATATATAGATAAATGTAGAATTGGATCGGGTCCTCAGAATGAATGGTACTTTTTTAGCCACAAAGACAAAAAGTATCCAACTGGAACGAGGACAAATCGAGCAACAATGGCTGGATTTTGGAAAGCTACAGGGAGAGACAAGGCCATTCATCTCAGCACTGCCTTAGCCTCAAAGAGAATTGGAATGAGAAAAACCCTTGTGTTTTACATAGGACGTGCTCCTCATGGCCAAAAGACTGATTGGATCATGCATGAATATCGTCTTGATGATGATAATAATGAAATTCAGGTACTTATATTTCTTGTTCATTATATATATATATATATATGTGTTGAGAATCTCATATCGGCTAATTAAAGAAAATAATTACTTCTTATAAAAACTTGGGCAGTCCTCCCCTCTTGAGGTACCTTTTGAGGTGAGTTAGGCCCAAGTCCAAAAATTCTAACATGGTATCAGAGCCAGTTGTTGGGCTGCCCGAGTTGGGCCACCGGGGTGTGTTGAGAATCCCACATCGGCTAATTAAATAAAATAATAACTTGGGCAGTCCTCCCCTCTTATATGGTATCAGAGCCAGTTATTGGGCTGCCCGAGTTGGGCCACCAACGCAGATGTCCAATCCTGCAAAATTTTAGAGGTACCTTTTGGGGTGAGTTAGGCCAAATCCAAAAATTCTAACATGGTATCAGAGCCAGTTTTTAGGCTGCCCGAGTTGGGCCACCCGGGGTGTGTTGAGAATCCCACATCGGCTAATTAAAGAAAATAATAACTTGGGCAGTCCTCCCCTCTTGAGGTACCTTTTGGGATGAGTTAGACCCAAGTCCAAAAATTCTAACATAGTATCAGAGCTAGTTGTTGGGCCACCCGAGTTGGGATAATTACAAATTTTATAAAGATTTGTGCAGTCCTTCCCTCTAGAGGTACCTTTTGGGGTGAGTTAGGCCCAAGTCCAAAAAACCTTTTGGGGTGAGTTAGGCCCAAGTCCAAAAATTCTAACAAAGGCTCAATCCCAAATTCTAACAATATATATATATTGAATCATCAAATCGTTAGTGTTAAAATTTGAGCTTGGATCTAGCTTACTCTAAAAAGTACCTTAAGAAGGAAAGACGGCCCATTTCTCAAACATTAGTCATGTGGGATTCTCAACAGTTAGCTAGCTGTTTATTGATAAAAATGTAATTTTAATGAGATAGGATATATAAGGAAGTAACAGTTAATGTGAAACGGAGGTAGTAGTTACAAAATATTTAATTAAGACCCTTAAAGTTAAACTATATAAAAAGGAGTCGATTAACTGTTTATGGTTTTGAGGTAGAAGCCTAATATCTGATGAGAATCACACATGGTTAGTATTTGAGAAATATTTATTTTTTATAAAAGTTTGGGCATTCTTTCTCTCTTGAGAAACCTTTTGGGATGAGTTTAGGGTTGTAAACAGGGAGGGTCTTATCAGTACTTGCGCGAGTCTATACCCGTTTATAGCGGGTAAAATTAAAATTTTATAGCAGTGGATCAGTAGGCCCACACTCATACCCTTATAGACTCTAGAGTATGACGATATGCAAACTCATATATCCATAATAAAATAATAAATACATAATTTATAAATTTTATATGATATTATTTCTCTCAAATGATTGTATTTGTTGAAAGAGAAGTTAAAAGAAATTATGTTAATCATGTTATAATTTTTAAATAAGGGAAAACAATATAAAAATGTCATATGAGTATAGATATATCCAAACTCTACCCTACACCCTGAATCATCGGGTATATCTCATGGTTTAGATCCATATTTCACTATAAATGTTTGGGTCTAGAAGCAAGGAAGGAACCAGAGGCGGCTCGGGTAGGCTCGAGCCCCTCGGGAAATTTTGGAAACTTTAGAATATTTAGGTATTTTACCTTCAAGCTCCTCTAATTTTTTTTTTCAATACAACATCAAAACTTGTTATTTTATTTGTAAATTTGGGATTATTTGAATAGTTTTGTACTTAGGATGCATCTATAAAATAAACGAAATTCTAGCAAAATTTTTATTGTTTTTCAGCCTCAAACCTTTTGTATCAGAGAATCTGTATCCGTTTATACCCCTAGATGAATTACTCGCACAGATGTCCAATTCTGCAAACTTCACGCTCCTCCCGATGAATTTATTTATGTTTGATTGTTAAATTTGATCATGCAGGAAGATGGCTGGGTGGTGTGTAGAGTATTCAAGAAGAAAAACATTAACAGAGGAGGATTCCAACCGATTGAAGCTGCTGCTCAGGAAGAACATTTAGCTCACATGGAGGTGGCGGCGGCGGCGGCTACTACTTCAACGTTGTTTATGAATCCAAAATTACCAAATTCTGGTGGAGCTCTTTGTGATTATACTACGTTTGATAATTCCATGCATCTTCCCCAACTGCTAAGCCCAGAGTCGGCAGCAGCGGCAGCGGCAGCCCATGCACATCCGGCGGAAAAGTTCGGCAGCGATTGGTCATTTTTGGACAAGCTTCTTGCTTCACACCAAGGTTTCGATCGAAGCAAATGCTACCCATTGTCTCAATTTTCTGATGTGCCGGCGCCGGCGCCGGCAGCGGCGTCTTCGGCTGATCATCAGAAGTTTTCGTGCCACTACTTATTAGGTTCTCAACTCGATTTCTTGAATTTTTCCAAGTAG

>CrNAC-14

ATGGTTGGTTGGTGAATTTTGATTTAGAAGAAGAAGAGAAGAGGGTGATGATGGAGTCCATGGAATCATCATGTGTTCCTCCAGGATTTCGATTTCATCCGACGGACGAAGAACTCGTAGGTTATTACCTAAGGAAGAAAGTTGCATCTCAAAAGATTGATCTTGATGTTATTAGAGATATTGATCTTTATAGGATTGAACCATGGGATCTTCAAGGTAATAATACACATCATTTCATTAATTTTCTTCCCCTTTTATTACAAATTCTCCAGGGTTCTCCCTTCTCTTCTTCTTTTCTCAAAGCCCTTGTAAGAGCTTCTCCGATCTATCATCCACAGTTCAAACATCTTCTATAACATCTTCTTTATAACTTTTGTTTTTAATCTATTATCTTGTATTGTCTAAATGAATAAAAATGTCGTCATCAGTTCTTCATGCACTTTGATTACCATCCACACTCCAGATCTTCTGCAACAATATTTCTTTTGATCCTTTGATCTACCATCTACATTTTAGATTTTCTATACTATTTCATTTCGCTCTATCATCCGGTCTGAATGAACAACAATAGATCTGTCATCAGTTCTTTTCCAAAGTGCATGATTTTAATCTACCATCCAATGTGTCTGGACAAATAGTAAACAATATTGTCCATTTTCTTGGTGAAATTCTAATTAGAACAACATTAAACCCTAGATTATAACATGTATATCTCCAATTATCTGAACATGAACATGCATGTTTTTAATGCTAGTTTGGTGATGGATGAACAGATAAATGTAGGATTGGATACGAAGAACAGAATGAGTGGTATTTTTTCAGTCACAAAGATAAGAAGTATCCAACAGGAACAAGAACAAACAGAGCAACAATAGCTGGTTTTTGGAAAGCAACAGGAAGAGATAAGGCAGTTTACGATAAATCAAAACTAATAGGAATGAGGAAAACACTTGTGTTCTACAAAGGAAGAGCACCTAATGGACAGAAAACTGATTGGATTATGCATGAATATCGTCTTGAGTCTGAAGAAAATGGTCCTCCACAGGCAAGTTCAtattattattattattattattattattattattattattattatTATTATTATTATTATTATTATTATTATTATTATTATTATTATTATTATTATTATTATTATTATTATTATTGGTCCCTTTTCTCAAACTTTAATTTCTTCGCCCCAATCAAATATGATTAATTTCCTAATTTCAAACCTTCTCTCTCTCATGCAAAACCTTTCCATCCAAGAAATCCAAGGTATTAGTCACCAGTATGACATAATATATATATATATATATACATAATATAATTCAAGAGGTCTATATATATATCCCAACTCTCGAATAATTCATTACAAATTTTCTCAAATATATATATATATATATGTTTTCTATATATCATAATTTCTAGACAATATGGAAAATTCATATTTCTCAATACTTAAAACACAATATTTTTTTTTTTATTTTATATGAATCTTCAGTTTTTATTATATCCATGGGGCCCATTTTCCTAACTTGTGACTTAGGAATCAGGCTATGTTTTTTTGCATGTTTGAATGAAAGTATTGAATCCTAAATGAAAGTCAAAAAGATGATCAATTACATGAATGAATACGCGGAAGGAAGTGCCTTTGTTTTTGGACTCAGAAGAGTCACTCTCTAAAAGAAAAGCTCCAACAAAAATTCTTTTCCACCTACAATTAACCTTTGACTAGTCTCCTAACTTCTATAAAAGTCAATAAAAAAGAATAATACATCAAAAAAGCAAAAGAATTTTCCACCACATATATATATGTTGAAAGAAAAAGTCTTTGTTAATTATCCATCTTAAAAATAAAAAACTACACTCAAAATCTTCTTATTATATATAGTAACTTCACTTTTTTCTTGAAATTTATCTTTTAAAATTTAAGATTTATATTCTATAAATATATTAAAAAGTATATTAGATTATTCTCAACAAAAAACGATATATATTTCTAATCTTAATGTAATACTCATAATAAAATTCTTATTATTATAGTTTCTTTCATAAATTCTACCAAATAAAATATGAAAAAATATTAGGTGGAGAGTTTCATTTTACATTATATACTAAAAATGGAGAAGTTGTTGGTTTTTGATGATGCAGGAAGAAGGTTGGGTAGTGTGTAGAGCATTCAAGAAAAGAACAACCGGACAAGCGAAAACCGTTCACGAAACTTGGGACACAAGTTACTTCTACGACGAACCACCCACCGCTACTACTAATAATACTACTGTTAATACCACCACAGCCACAGTAGTCGATCATCATCCCATTAATATTGATTACATCACAAGACATCATCATCATCAGCCGCCGCCGCCGCCACCAAACTTTCTTCATCAACGTCGTCAGAATACTAATTTCCTATGCAAACAAGAAATAGAATCATCAGAAAACATCATTAATAGCTTCATAAATTCCTCCTCCGATCCATTCGGACAACTTCCACAATTAGAAAGTCCGTCAATGCCCTTAGTAAAACGACAGAATTCAATATCTTTGATATCGGATTCGTCTCCGGAAGAAGAAGATGGACATCTTGGTGGTGGAAGTGGAAGTAGCAGTAGTAGTAGTAAAACGGCGGTGACGACGGATTGGAGAGCACTTGATAAGTTTGTTGCTTCTCAATTGAGCCATGGAGATGGAGTTGGAGTTGGAGGGGTGGATGATAGTACGGAAATGGGAATGCTATTAATGCAGAGTGGAAGAGAAGAACATGAAGGGAATAAGTTTATGAGTGAATTGTTTTTGAGTTCTAATTCGGATTGTGATATTGGCATTTGTGTATTTGATCATAAGTGA

>CrNAC-36

ATGAATGCGTTTTCACATGTTCCTCCTGGATTTCGTTTCCACCCCACTGATGAAGAATTGGTTGATTATTACTTAAGGAAGAAGGTTACTTCTAGAAGAATCGATCTTGATGTTATCAAAGATGTTGATCTTTACAAGATTGAACCATGGGATCTTCAAGGTAATTAATTAATTACGAGTAATCTTTAAAAAAATATATATTATAAAAAGAGATGTTATACAAAAATATATTTTCTTACGTCTTTTTTAAATAGAGCTATGCAGAATAGGCACAGAAGAGCAAAATGAGTGGTATTTTTTTAGCCACAAAGATAAAAAATACCCAACAGGAACAAGAACTAATAGAGCAACAGCAGCAGGATTCTGGAAAGCCACAGGAAGAGATAAGGCCATTTACTGTAAGCATGACCTAATTGGCATGAGAAAGACTCTTGTTTTCTATAAAGGAAGAGCTCCTAATGGTCAAAAATCAGATTGGATTATGCATGAATATCGACTCGAAACAGATGAAAATGGAACTCCTCAGGCAAGCTCAAACAATAACTTACCTAAGATTTTCATCCGTTAAAGTTAAAATGTCTAGTTCTGTCTATGCTGACTGACACACGCGATCGTTTTGGCAGGAAGAAGGTTGGGTGGTGTGTAGAGTGTTCAAGAAAAGGATAGCAACAATGAGGAAAATGAGTGAACATGAATCTCCAATATGGTATGAAGATCAGGTTTCATTTATGCCAGATATGGATTCACCAAAGCAGCAGCAGCAGCAGCAACAATATTCCCATTCCAATTTGAATAATACTTACCATTATCATCCTAATTATAACAATTGCAAGAAGGAGATGGATTTTCACTATCAAATTCCACAGGATCATCATTTTCTTCAACAACTACCTCTTCTTGAGAGTCCAAAATTGCTACAATCTGCACCATCATCAGTAGTCCCAAATTCAATGCCTGCTGCATTTGGTCTTAATATGAACCATGGAAGCTCTTTGCAACCTTCACTGTTACAGGTCCAGCAGGAACATATATTGCATCAGTCTCAAGATCATCATCATCCCCTGCATAATTCGGTTTATAGTAGCGATATGAGCAATGATCAAGGAGGAGATCAAGTAGCAACAGATTGGCGAGTTCTTGACAAATTTGTCGCGTCCCAACTCAGCCAAGAAGAACTATCAAAGGAAAATGACTACACCAATGCAAACAACAACTTTCGTGGTTCTGATGATTCGAATATGATGATCAAGGATTCGGAAAAACAGGAAATGGCAACAGAAAATGTCTCAACTTCATCCTCAAGTTGTCAAATTGATATGTGGAAGTGA

>CrNAC-15

ATGAGCATATCTGTAACCGATGAATCCGGCGGCGGGGTTCCACCTGGATTTAGATTTCATCCCACGGAGGAGGAACTTCTGCACTACTATCTGAAGAAGAAGATTGCACACGAAAAGATTGACTTGGATGTAATCCCAGATGTTGATCTCAACAAGCTAGAGCCATGGGATATTCAAGGTTTTGCATCCGCTGTTGCCATGTCTGCTTAACTAATTTGCTGTTTTCTTCCTCTCTTCCTGATATCAAATCAATGCGCCCATTTTTAATTTAATTAAAATAGGGTGTCAATTAGTTAATCTATTTGAATTAGAATAGATTTTAACAGGGAAAACCTTTTCACTTTTTTATATTTTGAGCCCATTAATCCTCTCACAAATAATTTATCTTTTCACATATTAAGAATAAAAAAATCTTCATTTTTTTTTCATTCTTATTTCCAATTCAAATAGACATCTGGTTAACTTTTGCAGAGAAATGTAAAATAGGATCCACACCGCAAAATGATTGGTATTTCTTTAGTCATAAAGACAAAAAATACCCAACTGGCACGCGTACCAACCGCGCAACTGCTGCAGGGTTTTGGAAGGCCACTGGCCGTGATAAGGTGATCTACTGTAATTCTCGATGCCGGATAGGCATGAGGAAGACGTTGGTTTTTTACAAAGGACGAGCTCCCCATGGCCAAAAGTCCGACTGGATCATGCATGAATATAGACTACTGGATGACAACCTCATGATCAGCACCACTAATAATAATGCTGTCGATGTGAATAATAATGTATAATTAAATACTATTACAATTCAATTGAAAAACCTTTTTTTTTTTATAATTAATTAATTAATAGCNNNNNNNNNNNNNNNNNNNNNNNNNNNNNNNNNNNNNNNNNNNNNNNNNNAATTCAATTGAAAAACCTTTTTTTTTTTATAATTAATTAATTAATAGCATTACTATCAATTTCTTGATTACAGGTCTGCATTAATGCCATGGAAGAATTACCACCAGTACTACAGGAAGAGGGTTGGGTG

>CrNAC-17

TCTCTCTCTCTCTCTATCTCTCTAGTTTCTTTGCAAAGCATAATCAATGGCACCAGAGCAGATGAACCTTTGTGTAAATGGTCAATCTCAAGTGCCTCCAGGATTCCGTTTCCATCCAACGGAGGAGGAGCTTCTGCATTATTATTTGAGGAAGAAAGTTGCCTCCAAGAAAATTGATCTTGATGTAATCCGCGATGTTGATCTCAACAAGCTCGAACCCTGGGATATTCAAGGTACCAAAAAACGGATCTATCGCGACTTATTGGTCGCAATTTGCTGTTTTTTTTTTTGTAGCGTATATCTTTTTTGGGGTCTCTTTTGAACTCATATTGTGACTTTTTTTTGGGGTCAGAAAAATGCAGAATTGGATCCACTCCGCAGAATGATTGGTACTTCTTTAGTCACAAAGATAAAAAGTATCCAACAGGAACTAGAACAAACCGTGCAACTGCAGCTGGATTTTGGAAGGCAACGGGTAGGGATAAAGTCATTTATAGCAACTCTAAAAGAATTGGAATGAGGAAAACTCTAGTCTTCTATAAAGGGCGTGCCCCTCATGGACAGAAGTCAGATTGGATCATGCATGAGTACAGACTCGAAGACACCACCCCGCCCCATGATGCATCATCTCTTGTGAGTCACCCCATCCATTTTCTTCCCTCATTTATTAATAATTTTGTCCTGAATCAATGTTTGTGGATAACAAATTATGAATTGTTGTATATTTTTAACATGTATGCTTTTCTAAAATGCATCATCTCCAATAAAAAAAATATAAGCCATTTAAGGAATTGAGGATAATAAAAAATATAAGAGTTTTACACGGTCATTTATTATATTTTTTTTCATTTATTATATTTTTTTTATATATATCTATCGCTTTATTTATTTATTACACTCTTATTAATTATCTATCATTCTCCTTATTTACTAAAGGTATAAATGTTATTTTGACTTAACAATTGTATTGTTTTCAATGTCCCTAATTCATGTGCACAACCCTATAACCCCTTATATTTATGACCAGAAGTAGTATATTATACTCTCCCTGTTTCAAAATAAGAGTCCTTTTAGCTCTTAGCACATATATCAAAGGTTTTAAAATCAAGAAAATATTTTACTAATATATCCTTAAAAAATATGAAGAGAGAAATATAAAAGAAGAGAGATGAGAGAAATCAATAAGGAGAGTGAGAGGGATAAAATTGAAAAAATATAATAAACGCTTGTTAAAAACTGTAAAAGGACTCTTATTGTGAAAAAAAAAATTTTTCTAGATAGTCACTTATTTTGAAATAGAGGGAGTAGCATTTTCAACTTTTAGTTTCAAAATATTAAAGACTTGATTAATAATAGAATCTCGAGAAATTAATTGTGAAAATATATACATGAGGTTTATACATACTCGTCCAACATTATATATACATATTTTATTTTTCTCTAGTTATATACATGGATAATTATTAGACATCTTAAAATATTTTGCCAATATATTACATTATAGACCAAGTGTTTCTATGGACATCTAATACACTTGTCAATAGAAAAAGCCTTTCATAATTAACTTACTTATGGCTAAACATGCATAGACACATAATTTTATTGTGAGATTTAGGGGTGAACAATACCTTTTCGATATTTGAAAATTATTTATGTATATAATTAAAAACATGATACATATATAACTAGTATATAGTGATGTATATTCATATAAATGAATATTCTTTGCTGATTTATTTAAGCAAAAGTAATTGGAGAAGAAAATTGTAATGAACTAGTTGTTGTGTGAATCTAGGCGTGCAGTATGGTAGGAGAGGGAAATTCTGAAGAAGGGTGGGTTGTGTGTCGAGTGTTCAAAAAGAAGAATTACCATAAAGCCCATGTGGATCAGAGTAACTCCCAAAACTCAATGATGGATCTTCCGGAGGGAAGTCTAATTGGAACGTCCAAAGATGGTATTCTTGATAGAATCCTTGTATACATGGGAAGATCCTCCTGCAAACAAGAAAACCCACCAATTAATAACATCCATGACGAAGACGACGTCGCCATGCAATTCATCAACAATAACCCAATTATGTCCGATGATAAATTCATGCATCTTCCTAGGCTGGAGAATCCATTATTCAACCAACATGATTGTAGTAGTTTCAGCGCCTCGCAGCCTTCAATGAATGATATAGCCGAAACTAATTCAGCTTCATGCACCACCGAAGCAGCCGCCGGGCTGAGTGACTGGGTGGCTCTTGATAGACTCGTGGCTTCTCAGCTAAATGGACAGGAAGAGGTTTTCTGCTTTCCGGCGGCGGCGGGATCCATCAGATCACACGGCTACCATCATCAACCACCTCAAACTCAAGCGTACGAGGGTGCAGGCAGCGAGTTGGACTTCTGGACCTTTGCCAGGTCATCATCCTCCGATCCGTTATGCCGCTTGTCAGTATGAAGGGAACAAGTTACTATCACGAAAAATAAATAGTATAATATATAATAGTA

>CrNAC-47

ATGGAGGATCTAAAAGCTCCCGTTGTTCCAACCTGGAATTCGCTTCCGCCTGGTGCCCGATTTTACCCTTCTGAAGAACAGCTCGTTTACTACTATCTCAGTTCAAAAAATGATGGCAGTAATTATTATGGAATTGATGTAATTAGGGAAATTGATCTTTACAGTTATGACCCATTCAATTTGCCCGAAATCTCTTGCTTTCGGTTTGGTAGGGGAGGGAGGAGGCGCCACTGGTACTGTTATGTGGGAAGGATCATCAGAGAAAGAGGGAGGAGAAGAGCCGGGTCTGGGTATTGGAAAAAGAGAGGGAAGGTTAAGGATATTGTGGGCGGCGGAGCTGCAGAGAAAATTGCAATGGGGACGCGCAAAAGCTTTGTATTTTATTTGGGAGATTCGCCAAAGAATGCAGTTAAGACTGACTGGGTCATGTATGAATATGCTCTTGGTGATCAAAACATGGTACCCATTTATTTGTTAAGAAATATTATTTTTTAAGAGATTGCTGCATAAATTAGCCGTCATTGCCTGTATGTTCAATTCTTGATAGCTGCATTAGTAGAATTGCTCTCATTCTTTTGCCAGATTAGTTCAAAGTTGAGTTCGGGATTGGCCTCCAGCGGTATGGTATTCTTTTGTTCTCGGCCATGGAACTTCCTGTAGTTTAATATGAATAATTAAATCACTTTTTCTCCTTTTTTCATTTAAAAAATTGTTAATTTGCTATTCTATTTGAATATCGCAGAAATCATGTTTAATTATCCATGTCATTTCATTTTTGCTTTTCTGGAGTAATAACTCTATTCATTACTATTATTAGATTTTGGGACTGGTTTTTGTTTGTTCTGTTTGTGTTATTGTTTTCTGCATACAAGGCAGGTTAATTGTTCCTTGCTTCAGTGACTAGAGTTTAGGTGGGTTTTTATTTTGTTAGGGGGGGAGGAGGATAAGCTTGAAATGAAAACTTGCTTGCTGTAAGGTTCTTAATTGTGCTTTTATCACCCAAAACATTATTCTGACTGGATATTGGTTTCATATCCCACTTATAAATAGTTTTATATCTTCATTTGTGTATTAATTGATAGACCTATGATTCTCGAATGTCACTTCTCAGTCCAGAAGATCTAGAAGATTTGAGTAGTGCACAGAGGGGAAGCGTGTATGCTACTACTTTTTCTTTGTTTTAAATAATTTTTTTGGAAGGGGGTGAGGTTGGGTGTAAATCTCATTCTTGTTTAGCAATGTTCTTTAATGAATGTTCTACTATTCCGACTAAAACTTCCATGCTTGGATATACATGCAGGCTTCTTTTGTCCTCTGCCGAGTGTTTTTGAGATCTTGTCCATTCAATAACTTAGAGAAGCACGTAGTTGTAAGTTTCTGTGGTGATGATAGTATTGCAACAGTGCATCGTTGTGTTGGTGTTCAGTGTGAGGGGACTACTGCATCTGTAATTGCAGAATCTAAAAGTCACAATAACAATTTTCATGATAATGACAATGAGGATTTGAAACTTTCATCTGGCCTGGATACAGTTAATCAAATTATTCATGAGCCAATGAATGAGAAGGTATCACTTTTATATCATAGTTGGCTTTACCCACTTATGTCATTTTTTTATTTTTTTTAA

>CrNAC-02

ATGCAAAGTCAAAAACAACCAATCCCCCATTTTTATCTTTAATTAATTTAAACTAATTTATTTTATTTATTTATGATGTAATCATACAAACCGACAAAAATCCCTCCTCCATAAAATCCCCATTCCTCTCCTTTACAACATTTCAAAACCTTTTCTTTTCTTCTTGTTCTTTTCTTGAAAATTCCTCCTCCCCTCCCAAACAAAGCCTAATTCCTCCTCTTCTTCTTCAACAAGAAAATCAAGAAAAGTTTATTTATTATTACCGGAAAAAAACCATGGTTGGAGGAAATTTACCACCTGGTTTTAGATTCCATCCTACTGATGAGGAGTTAATTATGTATTACCTCAAAAACCAGGCTACTTCTAGGCCGTGCCCGGTTTCTATCATCCCAGAAGTTGACATTTATAAATTTGATCCTTGGGAATTACCAGGTTAGTTAACTGCTAATCAACGCCACAATAAATTTTATGGTTTTCTTTTTCAGAAAAACGGCCCTTTTTGAAATAGTGTGTGATAAAAAGTGGGTTCTCCTTAATTGATTAATATATATTGTTTCTTGATTTTTTTTTTTTTTGTTGGTGTAGAGAAAACGGAATTTGGGGAAAATGAATGGTATTTTTTCACACCAAGAGATAGGAAGTATCCAAATGGGGTGCGTCCAAATAGAGCAGCAGTTTCTGGTTATTGGAAAGCCACGGGAACAGATAAATCAATATATAGTGGTTCTAAATATGTTGGTGTTAAAAAAGCCCTTGTTTTCTACCAAGGAAAACCTCCAAAAGGTATCAAGAGTGATTGGATTATGCACGAGTATCGTTTGATTGAATCAAGATCTCAAGTACCCACCAAACAAAATGGTTCCATGAGAGTAAGAACATTTTTATCATCATGTCTTTTTGATATGGAGTACCAAAATATCTACTGATTAATTAGTGATCATATTCATGCTTTCTGGGTATATTTTTTGAGTTAGTTTTAGGTCCAAATCTGTTTTTAAATAAAATAGTATTAGAGTCATCTAGTTCAATATTATTATGTCTAATCCTATGAATTTCATGCTCTAGATATTTAGTCCAAATTTGCACTATTTATTACTCCTAAGCTAGTGTTCTTGTTGCAAACTTCATAATCTAGATATTCAGTCTAGATTTTCAATACTTTTTACTACTAAGTTACTATGTTGTTGCAAACTTTACACTCCAGATATTCAGTCCAGTTTAACACTTTTTACAACTAAATTATTGTATTGTCGCATACTTCACACTCTAGACTAGTAGATTATTATCTTATCGAGCACACAGATGATCATATAATTAATTACTATGTTTCTTTGGTGAAATTTCAGTTGGATGATTGGGTTCTTTGTAGAATCTACAAGAAGAAAAATCTTGGAAAATTATCTATGGACATTAAAGTTGAAGATCAAAGTCAAGAAACATTAGTGGCAAATGAACTTGTTACTAGTCATGATGATGAACAACAACAACAAACATTCAAGTTTCCAAGGCCATGTTCATTATCTCATCTGTGGGAAATGGATTACATGGGCTCAATTCCTCAAATTTTTGGAGAAAATTCCATCTTTGATCAACAAAATATGTTCATGCTGAACAATAATAATAATAATAATGGAAATGTTAATACTCCTCGTCAATTAGGTGATCAAATGGGAAACCAATATTCAGAAGCCATGGTTAGATTCCAAGGAAATCAACCGGTTTATGTGAACCCGGTTTTTGAATTTCAGTGACACAATTATATATTTACATTATTTCTGTACTAGAAACTGATCAATTAGACGCGGACAAATCCGTGGCTAATTAGTCAGTACACAATAGGAGAAGACAAAAAAAAAATGAAAAAAAAAATGCCCCAAGTGGCCAATGGGATGTGGATTGATGGTTAGTTATAGAATGAAGAAAAATGATCAAACATTTGTAAAATTTTTATTATTTGTTTTCTAATTTGTGCAAAAAGTTAGAAATATATTGTTTTTTTTTAACTTAATAAATCATCATCGACTTATCTGAAAAAAATAAGAAAGTACAAGACTTATATGTAACATTAAATTATTTCTGAATGACTCGAATTTCAAGAACTT

>CrNAC-24

ATGGATCATAATAATAATAATAATAAACAGACCATTTTCCAATTCCCACCTGGCGTTAGATTTCATCCCTCTGATGAAGAACTCATCGTTTATTATTTGCTCAACAAACTTAATTCTCTTCCTTTGCCTGCTGCTGTTATTGCTGAAGTTGAGCTCTATAATTATAACCCTTGGGACTTACCAAGTAATTCTAATACCTTCGATATGGTTTAATTTCTTGGTTAATTATGATTTTCTAATAGTTATAATTAATGATATTGTGTTATTTATTACATATGATGATGCAGAGAAGGCTCTGTTTGGAGAAGATGAATGGTATTTCTTTAGTCCAAGGGACAGGAAGTATCCAAATGGAGCAAGGCCTAATAGAACAGCAGCTTCAGGTTATTGGAAGGCAACAGGAACTGATAAACCTATTCTAAATTCTTGTGGTGGAGAAAGAATAGGAGTCAAGAAAGCACTTGTTTTCTATATTGGAAAACCTCCAAATGGTAGCAAGACAGATTGGATTATGATTGAGTATCGAATTCCTGACACACTTATTATTCGACCTCAAAGATCTAAAGGTTCCATGAGGGTAAGTTATGTATCTTGAAAGTTAATTACTTCGCTTCTAGTTATAAACGAAACTAGACCTTTAACTTTGAGGGTGAGAGAGTTTATAACTTTCCAAGTCCTATCCTGTGCTTAGTTTAAATCCATAGAAATCTACAAGGGTATGTGGTTGCTAGATGTGCACTTTTTAGTCCCATGCCTATATATGTTTTAGATATGAGGCATCTATTTGAATTGGCAGGATGACAGAATCTAGAGTTATTATGATCATCTACTCATGTTTTGCATAAAGAAGACTTCATCTAGAAACATGCTAAGTATAAACCTTAATGAAGACTGATTCACTTCTGCTCTAGGAGCCCCACTCAATGCTTTATCACCCCTATGTTTACAGTAACTTCAACACATTACTCTATATAGTCCTGTTTTATATTATGTAATTTAGGCCTCTAATCAATAAAAGGAACCAGATGTTCAACTTTGAGGGCCCGAGATAGTATATAAAGAAAATTTTTAAGAGTTAGAGAGGGACGAACTTGAAGAAAATATATCTAAAAAAGAATATTTTGGGGAGACTGCAGGCATCAGTACCACCATGACCACCCCACCCCCCCAGTGGTTCTGTCCTTGCTATAACCTGAAGGTGGTCATGAGTCAAAATAAGAATAAAAACTTTCTGGGATTTGCTACAGATGATCAATTAGTCAGTACAAGATTTTATAAGGAATCTGATCATTTCAGTATATAATGCTAGCTACCTTTTATGCAGTTGGATGACTGGGTACTCTGTAGAATTAAGCAAAAAGGCAACATGTCAAAGAATGCATGGGATCATGTTCAACATAGTACCAGCAAACTGGTGGGAGACCTAACAAACATGAAAGAACAATTTCCATCAGTCAACACAAACAATGCTGCCTCAGACATCTGCTCAAACTATTTCCTATCCAAAGACTGCCATCTATTGGCTAAACTTCTTGCTACTCATCAATATTTTCCTAGTTCCATCTCCACTCTTTCTAGAACAACCTCTCAAAGCAGCAACAACAATGTCAAGAACTGGGATAGAGTCTATGAACACGGACAAGGTAAGGGTATTCCGGTGATTAACTCTTATAACTTCCATGGTTCTTTCAATTCACAAGAGAAGCCAAATGATGAGACTGAATATGGAAACTTTTCTCAACCAATACCACATTCAAACAAGCATGAGAATCTAGTAATCAGTAGTATGCTAGCTGCAAATGGTACGAGTTTCTGTAATCAACATGAATCTCAGGGAGCTGTATTCAAGAATAACTTGTCTAACGCCATCATGAACTTACAGGAGCTAGATGTAGCCGCATTTGCAGAAAGATTTCTACAGTGA

>CrNAC-01

AATTCGGTCGATTCTCTCTGTATTTTTTTCGTGTACCAATGCTCCCTATTCTCATCTCCTTCTCCTCCACCTCCTTTTCAACATCCAAAAATCACTCGGAAACTTCATTTCTAGTTTAAGCAATCTTCATCATATTAATTCGATCCTCCAATGGAAAGTACTGATTCGTCTACCGGAGGCGGCGGCGGTTGTTCGCAGCAGCCTCATCTTCCGCCGGGCTTTCGATTCCATCCGACCGATGAAGAATTGGTGGTCCACTATCTTAAGAAGAAAGCTGCCTCTGCTCCTCTTCCAGTTTCCATCATAGCCGAAGTTGATCTCTACAAGTTTGATCCGTGGGAACTTCCAGGTAAATACTAAAAAATATTGGGCGATATAAATTAAAATGAATTTAATTGGTTGTTTTCAGCGAGTAATTTTGATTATTTGGATTTTTAATTTTTTTTTTTAGCTAAGGCTACGTTTGGGGAACAAGAATGGTATTTCTTTAGTCCGAGGGATAGAAAATACCCTAACGGTGCCCGGCCGAACCGGGCGGCTACTTCTGGATACTGGAAAGCTACTGGAACTGATAAACCAGTTCTTACCTCTGGGGGAACTCAAAAGGTTGGTGTCAAGAAAGCGCTGGTTTTCTATGGTGGTAAACCTCCTAAAGGAATCAAAACCAATTGGATCATGCACGAGTATAGACTTGCCGATAATAAAACCAATTCAAAGCCTCCTGGATGCGATATTGCCAATAAGAAAGGCTCACTCAGGGTATGTGATTTCGAGATTAAACAAACCGTAGGTAATCGGAGAAAATGTATTATATATTGACTTTATTTTTTTTTTTTCAATGTAATTACAGCTTGATGATTGGGTATTATGTCGGATCTACAAGAAGAATAATACACCAAGGCCGATGGATCATGATAGGGATGATATGAATGATTTGATGGCATCAATACCACCTTCAATATCTCTAGGCCAGCCAAAATTGCCAGGACTAAAAACGACCAACTATGGAGCATTACTTGAGAATGAACACAACCTGTTTGATGGAATGCTGAGTGCCGATTTAAACAGTGGTGGATCTATGTCCCAATTAACTTCATCCGCTTCTAAGCAACAGCTTTCTCTACTCGCCGGAGCATCCAATGTTCTCCCGGCGAAACGGACGTTAAATTCTCTGTACTGGAACGATGACGTCGGTAACGGAAATTCGCCGCCTACTAAGAGATTTCTCGCGGATAGTAGCGACGGAAGTATGGCTACGAGAAATGATGAGAACGCATCAATCGCTAGTCTTCTCAGTCAACTTCCTCAAACACCATCGTTGCACCAACAATCAATGTTAGGTTCTCTGGGGGATGGCGTTTTCCGTCAACCTTATCAAGTTTCCGGTATGAATTGGTACTCTTAGATCATCAAGGATTCCGCCTCCGAAGCTGCTGCCGTGATGATTAATTGATAATATACAGAGATTATTTATGGTTATTTATTTTGGTTAGTTAATTAATCCCTATGGAGGATTAATTATATATGCTTTTAAGTGATGAACTTGAGGACGAAGGAAGAAGAGTTGAGAAATTGGATTTCCCCCAACGACGACGTAAGAGCGTTTTTCTGGCAAAGTCCATAGCCAATTTTAGGTGAGGCCATGTATATTATTACCTGCCCAGTTGAATATAGAAAAGCATTATCTGACTAATAGCTTTGGTTATTTTTAGCTAAGCAAAAAGGAATTTTCTACTTGATTTATAGAAATTATACACATAAATACAGATATATATATATATATATAGGGATATACATATAATTATATCATATGTTTT

>CrNAC-09

AAGGAAAAAAAAAAAAAAGATTTGGAGAATTCGACTTCTTCTTCCTTCTCCTCATCCTTCATATCGAAAAAAAAAAAAGAGTTGCAGAGAAGAATTATTTATTGAAATTATTAAAAATTCAACAAAAAAAAACGGAGAAGAAAATGGGTGTTCAAGAAACGGACCCTCTTGCTCAATTGAGTTTACCTCCTGGTTTTAGATTTTATCCAACGGATGAGGAGCTTCTTGTTCAGTATTTGTGCAGAAAAGTTGCTGGCCATCATTTTAATCTTCAAATTATTGGTGAAGTTGATCTTTACAAATTTGACCCATGGGTTCTCCCCAGTAAGTTTCAATTTTGTTTTCTCGAATTTTTGTGCATTTTTAATTTGGATTTCTAATGGCGAATTTGTTTAATTTTGTGAAGGTAAGGCAATTTTTGGGGAAAAAGAATGGTATTTTTTCAGTCCGAGAGACAGAAAGTACCCAAATGGATCGAGGCCGAACAGAGTAGCTGGCTCTGGTTATTGGAAAGCTACGGGAACGGACAAGATAATCACCACGGAAGGGCGAAAAGTTGGAATTAAAAAAGCTCTCGTGTTTTACGTTGGCAAAGCACCTAAAGGAACTAAAACCAATTGGATTATGCATGAGTACAGACTCTCTGAACCCCCTAGAAAAAATGGAAGCGCCAGGGTAAGTATTAATTTTTAAAATTAAATTAAAAAAAACGAAATTAATTACATTTCTATTGCAAATTCGATAGGAAATTGATTTTGAATTTTGGGAAATTTGCAGTTGGATGATTGGGTACTTTGTCGAATTTATAAGAAGAATTCAGGCGGTGCAGCAAAGCAACTTTCCGGTGTTAATAGTAAAGAATACAGCCATGGCTCGTCGTCGTCTTCTTCTTCTCAGTTCGACGATATGCTGGAATCCTTGCCGGAGATTGATGATCGGTTTTTCTCATTACCTAGAATGAATTCTCTGAAAACTCTTCATCAAGACCAGAAAATCAATCTTCAGACCTTAGGGTCCGGGAGTATTGATTGGGCCACTTTAGCCGGACTCAGTTCGGTGCCGGAACTCCTTCCCGTCGGTCAGAATCAAGCTCATCAGATTCATCAGGCAGGACAGGGGAATGTGAACCCGAACGACGTCGTCAGCGGTCACAGAGATATGTTCGTTCCTTCGTTTCAACAAATGTGCCACGTAGATGAGGAAGTACAAAGTGGGCTGAGAACTCATAACCGGGTTGATAATTCGGGTTTTTTACAGCCGCCGCCGCAGCAGCAGCAGCAGCAGCAGCACAGCTCGAACTTGATGAGTTTTCATCAGAATTCGATAGACCCGTATGGGATCCGGTACCCGACCCAAATGGGAAATGTGGGGTATAGGCAGTAAAAGTTGAAAAGTAGAGATGGGATTCAAATTGTGGTAGGAAAGATTATTTGGGGGGTGGTGTGTAAATATTTGAATTCTTTGGGCATACCTTTTGACTGTAATAATAGCAAAGGGGAAAGAATATTGGTTTCTTTTGGGGTAGGGGGTGAGGGGGGATTTGGTGGTGTTGGGGGCTGTTTTATTATGGACATCTGAACGTTGATAGAATATAGGGAAAAAGGAAAAACCATTTTAGATACCCCCATTTTGTTTTTGTATTATTAATTAGTGTACATATTCATTGTACACCAGAGAAATTCCAATTGAAAAACAAAAAAAAAATTACTTTCTGAGACA

>CrNAC-12

GATGAATTCAATGGAGAAAAATCCCAAATCAGAAATAATATTTCAACTTCCAGCTGGTTTTAGGTTTCATCCATCAGATGAAGAACTTATAGTTCATTACTTGGGAAAAAAAGCTAACAAATTCCCACTTCCTGCTTCTATTGTTGCTGAAGTTGAATTATATAAATTCAACCCTTGGGATCTACCAAGTATGTTTCTTTTTTTAATTAATTTCTTGCACTCTTTACTTCATTTTTTTTTTTTTTATAATTATTATCAAATACTAGTCTCATCAGATTTTACTACAAATTACTAACAGCATGCATGTGATTTTGAATACAAAATTAGCATATTTTTCTAATAATTTACGCGTTACTGCTTAATCAATTAGTTATAGAATACCTTACGTTTGATTCAATGAAATACAAAATTTTCATTTCTGGTTTTTACTAATGTTTATTTAAAAAAAGGGTTCGATCAATATTTTTTTTAGTTGATAAACTAAAGCAAATAGATATATGAGTGAAAGAAAACGGTTTATATATCTGCCATGCAAAGATTAAGTCTCATTTTCCTATGTGCAAAGCTGTTTACCTCAAGAAAGTTAGGTTATCATTACTTGAAGCAGGTTTAAACAGGAGGTTGATATATATATATATATATATTGATGATTTTCTTTTTGTTGTTTGGGGGTTTTTGATAGAGAAATGTTCATTTGGAGATGAAGAATGGTATTTTTTCACACCAAGGGATAGGAAGTATCCAAATGGAATGAGGCCTAATCGTATGGCAGGTTCAGGTTATTGGAAGGCTACTGGAATTGATAAGCCAATTGTTGCTTCTTGTGGATCAAAGGTTATAGGAGTTAAGAAAGCATTAGTATTCTACACTGGAAAACCCCCTAAAGGGAACAAGACTGATTGGACCATGCATGAGTATAGATTGCCTGAACCTGCTGCTCCTGCTGCTGATTCATCATGGACAACATCTACCACAAAACAAAACTCTATGAGAGTAAGTTTCTTCCTCTTCCTCATCAATCATCTATTATTACTTCCGGTCAGATATATAAGTTCTTTAAGGTGTATATATATATATATATATATAAATGATCAAGAAAAAAGTATGTATATAAGTCATTTTAATTCTAACGGCATTGATAAGAAGAATCAGTTTTGAAAAACACTTTCAAAATAGCTTTCTTTTAAAGTCTTTTTCAATGTTTTAAGCTAAACTTAATAAGCGGCAAAGAACCATTCAAAAAGAAGTTATAGTTTAACTTATTTTAAGTAATGAATAACCTCGTAAAAGTATTTATTATGTAACTTAAAGGTGTCTTACCTATATATTTATTTTACGTATCTATCTTTCCCTCTCTATCTCGCCAAGACTATAAAAATTATCTTACTTTTTACATTATATTCAAAATATATGTACATGATAAAAATGAACCCTACTTATTTTCTTGAGCTTTTCTCTTTGATTGATGTTTTTTTTTGCTTTGGTATTAATGAAGTTGGATGAATGGGTACTTTGCCGGGTGAGACAGAAAACAGGAATGAATTTTGGAAACTTTGGAGAAGAAAGAAATGGTCTTAATAACAACAACAACAACAACAAAACAGTCCAAAAACCTCAAAAAACAGCTAATTATTCCACCTTTGAAATTGTCAAAGATTATGATGATCTTTACTACAAAGAATATGGTCCAATGCTACCATTTCTCTTCAATGATAACTCTTCTTCTCAAGATTTAGCTTCCACAGATCAGACAACAATAAGTTTTGAAGGAAAAAATGTTAAGGATTCATCTTCATCATCAGTTTGTGAAGACAACTTCAACATTGGCATTGGCATTGTCAATGGCAAGAGAAAAATGTTTGAAGATCAAGACAACATTATTAGTCATAAGAAATTAGCATATCAAAAGGAGATGGAAAATGATGATGATGAGCTACTACCAATTACACCTTCTTCAAACAACAATAATATTGATCAACTTTGCTCAAGCATGATGTATCAAGAGCTCTACACTTTGGCATTCACATAATAGTTAAGATGGATCATTATTATTATTTTAGGTGTTTGGCAAAGCTTATTTTCATGGCTAGCCAAGAGCACTATTTCTTTTTATTTTAAGAGTGTGATTTTGAA

>CrNAC-46

ATGGCTTTCTTCCACCATGTCATTATCAACATTGCCAATATTGTCGTTACTCATTGTGAGAAATTTCAGACAATTGGCTCTGATACCATGAAAGTTTTCAGTGGGGAAAACAATCTATTGTTTTTTGTCTTATTAATATGAAAATAGTGTACAATATATAGATTGTCCATGTGACAAATCCTAAACAGATTATAAGTAGAAAACCTAACTAATTACAACTAGGAAACATAATTATACACAGTTTAGGATTAATAGTAATCCTATTGTAATTTAATTATATGTCAATCCTTAACAACCAAAGATAAAGAAGAAGAAATTGCCTCGTAATCTGATATTACATGAGAATCTTTACCATGATCCCCCTGATGAAATCACAGGTAATTAATTTGTGAATATGTTTTTTTTTGTGAAAATTTAATTTGAATAATATTTTGTAAAAAAATTTTAGGAAGATACAAGGAATTAACTACAGGGAATGAATGGTATTTCTTCACTCCAAGAGATGGAAAGTATCCTAATGGAGATCAGCCAAGTCGTGTTGCTGGGTCGGGTTATTGGAAGGCTATCAGAGCAGATAAACCTATTAAACATAATGGGAATGAGATTAGATTTAGAAAGGCATTAGTTTTTTACCAAGGAAAACCGCCTAAGGGTGAGAAAACTAGTTGGATTATGCATGAATCGTGTTAAGGAAGAAGGTACTAAGAAGAAAAGATGCGACAAAAACAAGAGGGTAAGATCTCTTTCACTTGATTTTATTGCTGCCACTTTTTGTATATTTGGAAATCTTTTCATATATTTCAAAATTTTTATTGCTGACCACTTTTTGTATATTTAGAAATCTTTTTATAAATTTCGAAATTTTTATTGTTGCCTCTTTTCATATATTTCAAAATCTTTTCATATATTTCGAAATGAAAAAAAGTATCCAAATTTTTTCTAAACATACAAATATATGCATATTCCCTTCAATATATATATATATATATATGCATGTTAAATATTTATTTGGCTCTCAAATTTTTATCTTTTTATTCTCTTTTTAGATGTCATGTAAAGGACGAAGGTACTAAGGAGAAAAGTAATGACACAGATATGAGGGTAAGATCTTTTTCTCTTCATAATATGAAACAGAGAGGTAGTCTTGATTTGTAGTATTTATTTCATGCACATGCGAGTTAAATACTTTGTGGCTTAAAAAACCTGCAATATAGTATATGAATTAGCTAATAATCTTTACGCTTATATTGATTTATTTTAGTTTGAATGTTTGTATTGATTTTTTTTGGTACTACATATTTTAATATTTTAATCATGTTATAGATTTTTAATATTTGTTTTGTTGGCATGGGCAGGTGGATGATTATATCTTATGCCGGATTTATAAGAAAAATGATAAGATGTTTAGGAGTCAACTATGTGGTGAGATTCAAACTCAAGATCTTCTTCTGGATGAAGAAAACAATATTAATATGACAAAAGAAGAAAATAAGAGTGAATTTGTGAATCCATATGGAGATTCCCAATTTTCTAGCTTTCAATCCGATTCTTAATCAGCCGGAGTGAATTTAAGGGTGTATCCTTCCGGAAACAACCCCTTTGGATATCTGACAGAAGAACATATGTGGAGTCTACCACCAGAAAATCAAGTGCTTCCAGAATTTAATTTTCCTGAAATTAAGTATTATGATTCTGGTTTATCAGAATTGGATAATATTCTTATATATCCAAATCCAACCATGGATGATCATCATTTATCAATGCAGCAAAGTCAGGTGGATAATCCTTCATTACCATTAACAAAGAGCTGA

>CrNAC-07

ATGGCTGCAGAGTTGCAATTACCGCCTGGATTCAGATTTCATCCGACCGATGAAGAACTCGTGATACATTATTTATGCCGGAAATGTGCGTCTCAACCGATTGCGGTCCCGATTATAGCTGAGATTGATCTCTATAAGTATAATCCTTGGGATCTTCCTGGTAATTTCCTAAGCTTTTACTATGAGATAATTTTTATCTAATTAAATGAGTGGCTGAAATTTGACTAGAAATGATGAACTGATGATTATATTAGGTATGGCGTTGTACGGGGAGAAGGAGTGGTATTTTTTTTCTCCGAGGGACAGAAAGTATCCGAACGGTTCGAGGCCGAATAGGGCGGCCGGAAGTGGTTATTGGAAGGCGACCGGTGCGGATAAACCGATTGGAAACCCGAAACCGATGGGGATTAAGAAAGCCTTGGTGTTTTACGCCGGCAAAGCCCCAAAAGGAGAGAAGACTAATTGGATTATGCATGAATACAGACTCGCCGATGTTGATCGATCCGCCCGTAAGAGGAACAACAGCTTAAGGGTGGGTACCTAACTTTTTCCTAATCTTACCCAAAATTACGAAAATACAAATAAAATTGGGAGAAACTGAAGAATTTTAATGAATTTAACGGAAAAAAAAAAAAGCAAAATAATTGAGTTGACTGATAAAAGGATTCTGATCGGTTGTTATTGTGCAGTTGGATGATTGGGTTCTGTGCCGCATATACAACAAGAAAGGTACAATCGACAAACAACAAATGATGGGCATTCGTAAAGCGGCAAGTCAGGTGATTGAAGAAGAGGATAAGAAGCCGGAAATTCTGAAGTCGGTGCCGGAAACAACACCGGTGGTGTACAGCGATTTCATGTACCTGGATCCATCAGATTCCGTTCCCAAGCTGCACACGAACTCCAGCTCATCTGAGCAAGTGGTGTCGCCGGAATTCTCTCAGAACACATGCGAAGTACAGAGCGAGCCGAAGCTGAATGACTGGGAGAAATCCACTTCTGCCCTTGATTTTCCATTCAGTTACGACGGCAATGTGCTTATGAACACTCAGTTTCCGAGCAATTATCAGATGTCGCCTCTCCAGGATATGTTCATGTTCTTGCAGAAGCCATTTTAAAAGACAAGAGGGAGGATGAAAATTCCTGGAAATTTTTACATTTTCTGCTCGCACGTGTAACATGAGACTTAGGTTTATGAGTGTACACGTGCGAACTCATTGGCATAGGCAGTAAAATATGGGTATTAGAATTGCTTGGGCAGGCAATTGTTAATTAATACATAGTATTATTACTTTTCATTAATTATTGTGATGTATATAATATATATATATATATTTTACTCAGTTGATGATACCATTTTGAGGGAAATGGTAAATGTTAGTGTAGAGAGAGGGTATATTAGTCAAATTATATGTTCCAATCTATGAACTTAGCCTCCATTAAAAGCATTAATAATTCAGTCAGTCAAGAGAGATCATCCAATTAAAAATGGTTTAATTATATTTTTTTATAAATTTTAAATACATTATTATTTTATTATATAATTATAACTAATTAATTTTTATTATTGATTGGAAAAAAGTCATTCTAGAAGAATGTCTATTTCAGATGAAGCAGGAAAAGCCTACCCTACAAAAGGCTCCGGAAAGCTTTTTCATGGGGTGATAATCATTTAAATAAATATATGGATTATTATATCTTCATATATTACATATATTAATCTTAAGTAATAAGAAATATATTAGCCTAAATTCTTCATTATTGATGATAATGAGAAAGTTGAATGGGTGAAGATTATATTCCGTAGCTGTTTATATATCATACTTCAATCTTATATATATATATATATATATAGATATTTTATATTTCATTAAAATGTTTGATTTTTTTTAAGCTTTCATGTATGTTTTTTGTCCACTAATTTCAATAAATAGTTATAAATTTCAATAAAATTAAGAATTTTGTAGGGAAAAATTTAAAATCGTTCTAAATATCATCTTGTTTGTGTAATTCTTATTTACTGAATAATTATTTGTGATTCTAATTTTTGTAGTGAAAGATATTGTTGTTTTCATATTAGTATTTAAGGTTATGTTTGTTTAAATTAAAACTGAAGTTTGAATTTTGAATTGTTTAAGTTATGAATTCATAGGCCTTATTTGTTAAATTAATTTACCACTTAAAATTAATTAGTTAAGAACTTATTGTAATAAATGTGTTTGATAATAAACATGATTAAGTGATTAATATTTAATTACATTTGATAACAATATTTACAAATTACATAATTATAGTTTTAAATTTAAGTGAAAGCTATTTTGCTAATCTCTGAAATCTATATATATATATTCTTTGTTAAATTATTACAATCTTTTGTATATAGTTATAACTTATTAATTAATTCATCATTCAAATTTCAGTTTTAAGATTCAAAAATCAGAACTTAAACGACTCAAAATTCAGATTTCAATTTCATTTTTATCAAATAAAGCCTTAATCTTAAGTTTGAACATTGAATCTGAATGCTAAATTAATTAATAAGTGGTAATTATATGAAAAAAGTATAATAATTTGACAAATAATATATATTTAGATTTTAGACTTTAGAATTAAGCAAAAGATTTTCACTTAAAATTTATAATTAACAATTAAGGACATCTTAAAAAATTAAGTGATTTGCAAAAATTGTTATAAAACACACTTAAACATTAACCATTTAATAATATCATTTATAAAACACATTTAATCCAATGAACTCTTAACTAATTAATTTTAAGCAGTAAGTTATTTTATCAAACAAAGGTCTAAGCAAGTTAGATTTACAAAATCATATTTAGGAAAAGCACTTAGATAGCACCATCGAAGTCATCCGATTCCATGGTATGACAGTTTACCATCGTCGTCCCAACCTCCTAAAGGCAACACTACTGGCCTACATATCCAATACCAAAATATCAGATCTGTAGATATCCGTCGTCAACACCGCCATCTTCTCCACCTTCATCAAAGAAACACAAAACTATGGAAAAGGAAAAGAAGAAGAAGAAGAAAAACCAGCACAACAAAAAAGGAGTAGACACAAAAAAAGGCATTAAAATGAAAAGATCAAGTGAAGCTCAAGCTTCGACTGAAGTTGGAGTCGGAAAGAATACATAATGAAACACGGTAAAAGTCGAGTTAGAAAGAAGGGTGAGAGATGTTGGGTAAAGAGAGGAAAGTTTATTGGAAGTCATTAATGCACATATGTGAATGAAAAAAGAGAAATTAGCTATAAATGATCGTGAGTTAATAACATACCACCCTGAAGTAAGGGGGTAAGATTACGTTACCTTCTAAACTAAAAAAATATGCAATTTACCTTCTATGACCAATAGTTAATGATTAATTTAAGTAAAATATCCAAAATACCCTTCATGTAAGTTATAATTTATTATAAAATAACCCTTAAAAATTTGTTGTAACAATCAGTTTAATCCATATTGTAGTTTATAGATATAGTTTGTTACAAAATATTTTTATGTTTATAACAATAAACTTTTATTTTATTTTTTTATAACATGAACTATTTTAAAAATATTAACCTAAAACTTCTAATCCTTTTAGACAGTGCCATCACCAATAAAATTATCCATATTTCCTGCAGCATTCTCGAAGAGATTAGCAGTATCCAAATGACTTATATCAATATAAACATCATCAATATTTGCATCATCATCATCATTATCATGAGTTGAATATTGAACAGTAAAATTAGCTTCAATTTATTTGTCATTCTTTTTAAGTGATGCCCGGTAAAGTTTAACCAGATATTTTGTTGTACAACAGGTACGGGACCAGTGAACTTTCATTCCACAACGGTAACATTTATTTCCATTATTTGCAAGATCTTCAACTTTCTCTTTTCCTTATTATTTTTTCCCACCTCTGGTGGTTATCATTATTTTCCGTGCTTGATAAGGTGTGAGACTAATATAGAAAATACTACCTGAAGACAAAACACCTATCGGAGTAGCACTAGAGAATTTCCAAGTTGTTGAACATCTTTTTCTTTTCTTACATCTTGTATATATTTTCTCTCTCGAAAAAAAGAAAAAAATACATCTTGTATATGTTTTTACAATCACATAATATTAATTCAATAACACCTATAAATTCTAACTCTTATTCAAAGGAAATAAATAATAATCAATAAATTAATTGAATTCTGATAAATATCATTCGTCATGTTTTCTATAGAGGAAATTAATGATTAGAATACTCTTCTAATTTTAGACGGAATGAACGTCCCAACAACAATATTAGTAGTAGTAGTAATGTTTAGGATTAATTTAATTTAAGAATAATTTTCTAATTATATTTAATTGTCCATTTTTGTCCTTGTTTTTTCCATTCTTCTTCTTTCTTTATCTAATGATGACTGTCGACGATTTTCAACGAGAAAATCCTCAGTGCCAATTAAACTCCGATGCATCTGAAAATTTTCCCTTTATTAATTCTTAGAGGCAAAACTTATCACATTCACCAGACTTTACAACAATGGCTTGACGTGGGGAAAAAGAAGATCGGAGAAATGACAAACCGCACCCCTTCACTGTTACAACCAATGCAAGATGACAACTCACACCCCTTCATTAAAAACCACCGATTCCAAGTGTACATCCAACTCAGCCTACGTGTCATTTTCATCACCGTTGGATCTCTTTTTATTTAGTACTACTATGATTGAAGCCTATAATCCTTCAGCAACTTCGAGGAAACTGTTAGCTTAAGTCCCTTCATCTTTACACGTACACAAAATTCTAAAACTATAGATGTAAGCAGTGACAAAATCAACTCTCTGTAAGCTTAATCGCCACGCACGCAACCTTACTTCATTTCTCGACACGTGTCCTTTCTCTCATACCCTTTCTAGCATCAATCCATTATAGATTAACGTTACCGCCCATATGTATTCTAGAAACCAGCTGCCTCTCCTTGCTACGTGTCCCTCCTTCTACCCTTATATATACCCCTCTTTCCCTTCTATCGGTGTTTCAACCTTCAAACCCCCAATTTTCCCCATAGCTCTCTCTTTCTCTCGCCCTCTCCTTCTCCATTTCTCTCTAGAGTAGACTTTTCTAGTGAGAAGAAAAAAAAGAAGAAATGGCCGCGGATTTGAAAATGCCCCCTGGATTCAGATTCCATCCGACTGATGAAGAACTCGTGATCCATTATTTATGCAGAAAATGTTCGTCTCAACCGATTGCGGTTCCGATTATAGCTGAACTTGATCTGTATAAGCATGATCCTTGGGATCTTCCTGGTAATTTTCTCATTTTTTACAAGATAATTTTATCAATGAAATAAATTTCCAGTTGAAATTTGACTAGAATTGATTAACTGATGATTATTAGGTATGGCGTTGTACGGGGAGAAGGAATGGTATTTTTTTTCTCCGAGGGAGCGTAAGTATCCAAACGGTTTGAGGCCAAACAGGGCAGCTGGGAGTGGTTATTGGAAGGCGACGGGGGCAGATAAACTGATTGGAAACCCTAAACCGATGGGGATTAAAAAGGCGTTGGTGTTTTATGCTGGCAAAGCACCTAAAGGAGAAAAGACTAATTGGATTATGCATGAATACAGACTAGCTGTTGTTGATCGTTCCGCCAGCAAAAAGAACAGCTCGAGGGTGGATATATATAATTTTTTCCCCATTTTCTACCAAAATTACGAAAAACCTTATAAAATTGGAATGAACATTAATGCGAAGAGCCGAAGAATTAATCAATTATAGATTTTATTTAATGAATTCAACAAAATAAAAAAACAAAATAAAATAATTGACTTGATTGATATAACGATTCTGATCGGACGGTTATTATTGTACAGTTGGATGATTGGGTTTTGTGCCGCATATACAACAAGAAGGGTACAATCGAGAAACAACAAATGATGGGCATCATGAAGGCAATAACTCAAGTGATTGAAGAAGAGGATAACAAGACGAAAATACTGAAGCCGGTGCCGGAAACAACACCGGTGGTCTACAATGATTTCATATACTTGGATTCGTCGGATTCTGTCCCGAAGCTACACACGACGAACTCCAGCTCATCGGAGAAAGTGGTAGCGCCAGAATTCTCTCAAAACACATGCGAAGTACAGAGCGAACCGAAGCTAAACGACTGGGAGAAGTCAGCTTCCTTCCTTGATTTTCCATTCAGTAGTTATGTGGATGTGCCGCCGCCGCCGCCGATGGACAACGGCGGCAATGTGCTTATGAATTCTCAGTTCCCGAGCAATTATCAGATGTCGCCGCTCCAGGATATGTTCATGTTCCAGCAGAAGCCATTTTAA

>CrNAC-06

GCATCCACGTAACTTCCGTAACTCAGTAGCAGCGGGGTTAAGCAGAAGGAAATCGATCACAGTACAACTGTTATTAATTAGAAGAGGAGAAAAAGGAAGCAATCGGAAGCTGTAATTACAGAAAGGAATGGATTATATAGGTTATAGGGAGTCCATAGACAGCGGCGATCGAAATCAGAAGCTGGATTTGCCGCCTGGATTTAGATTTCATCCGACGGACGATGAGCTCGTCACGCATTACTTGTGTCGGAAGTGCGCTGCTCAGCCGATTTCGGTACCAATTATAGCTGAAATTGATCTGTACAAGTTCAATCCCTGGGATCTTCCTGGTAATTAATTAAAATTTCTGGTTAAGATTTTATTCCGAGAGTGTTTGATTTTGATTTTCGTTCAATTTTAAGTTGAAAATTGTTTGATCCAAAATTTGAGTTTGAATTTATAAATGTTTTTTTTGTTTAAATAATTATTCTTGTGAATGATACAGGAATGGCTTTGTATGGTGAAAAGGAGTGGTATTTTTTCTCTCCAAGAGATAGGAAATATCCAAACGGTTCGAGGCCGAACAGGGCGGCAGGGACGGGGTATTGGAAAGCCACAGGAGCGGATAAGCCCATTGGGAAGCCTAAACCACTGGGAATAAAGAAGGCGCTTGTGTTTTATGCCGGAAAAGCCCCCAAAGGAATCAAAACCAATTGGATAATGCACGAGTACCGTCTCGCTAATGTCGACCGCTCCGCCGCCGGCAAAAGAACCAACTCCAGGGTATGTTATGTCCAATTTCGTTACTACATATTTCTGATATTGTAGACTTAGTAGCATTGGTAGTAACCTTACTAACTATGGACTTGGGCTGTGCCTGTGTATGTCAATGCAGCTTGACGATTGGGTATTGTGCCGAATATACAACAAAAAGGGTACTCTTGAAAAGTACAACAATGTGGATCAAATCCAAAATTCGATCGAATTCCAAGAAGACAAGCCAAGAATAATCGGATTTGGACAGTCAGAGACAGCCAGGAAATCTAATCCTCCGCCGATGCAACCAAATGTTCAACAAACTAGGAATGATTATCTGCATTTTGAAACTTCGGAGTCGGTGCCAAGGTTACACACAGACTCGAGCGGATCAGAGCAAGTGTTGTCTCCAGATTTCCAATGTGAAAAGGAAGTTCAAAGTGCGCCTAAATGGGATGAATTGGAGAGAACTCTTGATTATCAGATGAATTTCATGGACAGCTTCCAAAATGATGATGACCCTTTTGGTTCACAAATGCAGTACCATCAACACTTCTCTTCTCCTTTTCAAGATGTATTTATGTACATGCAGAAGCCCTTCTAAACTACCTTCCTATCTGATGAACAGAGAACTGATTCACCCATGGAAATATGGAAAAATTCTGTTATAAACTCGATCGATGTCCTGACCACGTGGCCCATATTCAACAATATCATTCTTCTGGTCTCATAATTGATCTTCATAATTGAAGACATAATTAATACATGAATGGGACGTAGGCTTGTTTACAGGTTCATAACAAAATTACAAAATTTTCCGGAGATAAAAGGCTTGGGTGGTTATTTAGACCAAGGAGTTGCACATAAACTTCCTTTCTTTTTCTTTACTCAGAAAAATGGCCATGGAAGTTGTGTGATAGTTTGATAAAAGATAAGACAATACAGAAGACGGAATAGAGTAGAAAAAATATCAACTCTTTTTAACTCTTCTTCTTCTAGCAGTAAGTAGGTAAGAAGAAGTGTAACTTGATGGGACTATGACAGGGTTAGCGGTAAAGTTATAGAATTAATACCCATCAAGAAAAAGGGGAAAACAAGGATGAAGCTGTAGAAGTGATGGCAGAGCCTCATTCATGTACCTGTGACATGTTATAATTTGTTTATTTGATTCTTTTTTTGCTTACTAAAAAGGTGATAAATGAAAGATTTTCT

>CrNAC-34

ATGTCCAATAATGCTGATGTTACAAATGACAATCATGGATCATCAGATCAGTGTAATAATTCTGATCGAAGGGATGAAATTCATGTAGTCAAGATTGATGAAGAAACAGAGGAATATTTTAAATCTTTTCCACCTGGTTATCGTTTTTGTCCAACTGATGCTGAACTTATTCTTCATTATTTGGAGAAGAAAATCAAGAATGAAAAATTGCCTCCTCATAGGATTCAAGAGGAAAATCTTTATAAATTTACTCCTGATGCTATTTCAGGTAATTATTTATTATTATATTTTTTTTTTATTTTAGTTTGCATTATTGTAATTCTTAATTTTGATCATATATGTTGTTACTATGTCATGATCATAAGCATTCATTCATGTATATTTGGAAAATTCTATTTTGAATCTATTTTGGTAGTATATTAAAATTTGATATTTTTTTATAACATTTTGGATTGTATATTAAAAAATAATAGTATTTTTTATATACAATTATTATCCATATATCTAATCTTATATAAGAGATTGTTTATAAAATTTTTCTTTTTATAATCTTTTACTTAATTGAACAAAGTCATTATTTTTTTAGTCACAACATGTTATTAGCATACATGAATGCTAAGTCTATCATATCAACTTTGTGGGAACTATTAATTATTATTATTATTATTATATTTTTTTATTTAAATTTTCATTATTGTATTCTTGATTTTGATGATTCATATGTTATTATTTACCTCGTGTTTATAATCACGAGCATGTGTAAGTTTCTTCAAGAATTAAGAATTTCTGGTTTAAATTCAGTAACGTTTTTTAGAAATTATTTTTTCTAGGAAGAAACAAATATTTATTAGCATGATAAGCCCCTTCTAATGGCTGAAGGAATAACTTCATGGCCATTTTTGAGTTCAGAACATAGGCTGGCCTGGGTTGAGCCACATTGTGGTCAAAGTGGCCATATTCTTTTAAACTTTTTCAAAAATCTATAATACTATATAAAATATACCTATTTTCTATTAAAAGTTAGTATTTTTATAATTTTAGCACCAAAATCAATTATGAGTAAATCATTAAATTACTGAAAATAATAATTCAATTTCAACAAACAATTACTGAGTAAGTATCATAACTGGTGTTTTTTAGGGTTTTGACTATTTTTCAACATATTTTTAGGCCTTTTTTATATTTTATTTATTTTGAAAAGTTCAATTATATAGTAAAAATTGTGTTTTTTAATGCCTAATTGTTTTTTTTAATAGAGATGTATCCAGTACTAGGTGAAAGGGAATGGTATTTTTTCACTCCGAGGGATAGAAAGTATTCGAACGGGACGCGTCCAAATAGAGCTGCTGGAACTGGTTATTGGAAGGCTACTGGGGCAGATAAGCCTATTAGAAACAGTGGTTCTACCATTGGATTTAGAAAAGCATTGGTTTTTTATGAAGGAAAACCACCTAAAGGTGACAAAACTAATTGGATCATGCATGAGTATAGAGTCAATGCTGAGCCAAGGAAGAAGAGTTGTGCCACTGATATGAGGGTTTGAATCCTATTTTCTCCCTTTTCACTTTGTTTTAGGTGTCGGAAATAAATTATTAGTATAGTGCAATTGATATGCGTTGTTATATAACCTGATACTTCTTTAATGTGTTTTATGTATATATATAGTTTTCCAATGCGCCATTAGATGATCATTAGTGAAATTCACTATTGTTAGTTAAATGCTCTTGTTTTATTATATTTGTATGATATTTCTTACTATTTTATGCTATGATATTATATATGTTTATTAGGGTGTTTATGTGTTTTTAAGTTTTTATTTTTAGATATATTTGTGGGTAAGTTTTTCTAAGTTTCTATTTTTAGATATATTTGTGCTTAAGTTCGTCCTCTAATATTTTGAATTTTTTTTCTTTGCTAATGTTCGTTTCTTTGCTTTTTTTAGTTGGATGATTATGTATTGTGCAGAATTTACAAGAAGACCGATAAGTCATTCAAGGGTCGTCAAAGGAATAACGGAGGAGGGGATGATTATGTCCCTAATCAAAATGAATATCTTTCTGCAAATTCTCAGGATCATAAGAACAATGCACAGCCAGGAACGGATAATGTTCATTATGGAAATCATGGTGATGGTTATAATCACATGGAAGATTATTCAAAACTGGACATTCCCACTCATTTACAACATCATGAAATCCCTGACATTTCTTCTGCAGAAGATCAAGAAATTCCTAATTATGATTCTGGTAATTTTTTTCAATCCTCCTCTGACTATGGATTTGGGCAAACTCCTCCAACTTGTTTCCTGCCAATGTCTTCTATAAATCCGATATCTTCGTGGGAATCAAAGCCAAGATTTATGCCTGTGCCTCCATTTTATTCTACCACAAACAATGTGTTTAGGACGGGCTATAATATGAGAGAAGCTGCCACTGCATCATGGGATTTCAAGCCGCCTCCAAGAGGATTTCAAGACTTTTATGATTTTGATATTCATAATAATTTAAATTTTGTTGATCCTGAAGTCAATTTAGATAATATTCTTAACAACCATCCTTCTTCATCTGATTGCAGTCCCTTAAACAGTCTTCAAAAAAGACAAAGAGATTGA

>CrNAC-39

ATGGAACACTACTTTTCTTGCCCGAATTTGCAAGAGTTCTCTTCAAACAATCCAATTCCTACTACTACTACGACTACTACTACTACTACTACTCATTCCGAAACCAATTACACCCATAATAATATTAATGATCATCAATACATATTAGGCCAACCTACATTAGCTTCTTCTGGTGGTGGTGGTGAGATCGGGGGTATCGCGGCACTTGATAATTACAGCTTATTCGGAGATCAACAAGGTCATGATCATGACATAATTAATAATATTAATATTGATCATTGCATGAGTGGCTTTTATGACCATGAAAATCTTTTTCTTGATCAATATTACGAACCAATACAAGCTGTACCTGTTTCTGATGTACCTGATGTTCATGTCCATGTCGTCAATAATAATAATATAAATAATGAACGGATGATGCTTCCGTCTGGTTTCCGATTTTGCCCTTACGATATTGAGCTCATCCGAGACTATTTGATGAAGAAGATTGCCCATCCCCAACTTAATTGGGATCATATTAAACAAGTCCAGCTTTATGACTGTGATCCGTCTCAACTTGCAGGTATTACTTTTTTTTTTTTTTTTCTTGATTTTTTTTTCCTTGGAGTGTTTCTTGATTTTCTTGTTCAGTAATTGAATATTTTTCATTTACCCTTGTTAAGGAATTACAAGAATGAATTATTTTTGAAAATTTTTTTTATGGAATTATTTTTGATATTTTATATAAATTCTTGCGATCATAATCAAAGCTGTAATTCTGTCCATCTATGTTAAATGTTACCCTTATTATGAAATTGGTCTACTTAGACTTGAATTTATTAGGGAGGAAGGGTTTGTCAAAAGCAAAACATTAATTTTCAATAATTTCCTGACAATCTTTATAATATTATTATTATTATTGTTATTATTGTTAAAATGGAATTATTATTAATTATTTCTTTTTGTGCAGCTTGTTATCCAAATGATGAAGGTGAATGGTACTTTTTCACTGAAAGGGACAAAAGATATCCAAACGGCGAGAGACCAAATAGATCAACAAGAAGTGGATATTGGAAAGCAACTGGAGCTAGAAGAAAAATTGTTGATAATGAAGGTATAGAAATTGGGAATAAGAGGCCATTAGTCTTTTATCAAGGTAAACATAAAGACAACCGAGAAAGAAGCAGCAAAGAGGAACCCAAAAAGACTGATTGGATTATGTATGAATATCAAGTTCATGATCCAACAACAACAACTTCTAATTCTAATCGCCGAAAAAGGGATGAATTTGAAAATACAACAATGAGGGTAAGAATCTCACTCTTTATTAATCAATGTTGTTATTTTAATCAGCTTTACACTATTCTTTCTTTAGAAATAATTTTTTTGGACTATTTTTATCTATTTATCGTCGACTACTTGCTATGTGTTAATACATAGCTTTAAAAAAATTATTTTTGAAAAGATGGTAACATGTGGTATTTTTTTAATTTTAATTTTATTTATTGATACTATTAAAAATGATAATTTTATAGGGAAAATATAGAAATAATCCCAAAAGTGAGGGCACTTACTTGCGTAATCCCTAA

>CrNAC-44

ATGGAACACTACTTTTCTTGCCCGAATTTGCAAGAGTTCTCTTCAAACAATCCAATTCCTACTACTACTACGACTACTACTACTACTACTACTCATTCCGAAACCAATTACACCCATAATAATATTAATGATCATCAATACATATTAGGCCAACCTACATTAGCTTCTTCTGGTGGTGGTGGTGAGATCGGGGGTATCGCGGCACTTGATAATTACAGCTTATTCGGAGATCAACAAGGTCATGATCATGACATAATTAATAATATTAATATTGATCATTGCATGAGTGGCTTTTATGACCATGAAAATCTTTTTCTTGATCAATATTACGAACCAATACAAGCTGTACCTGTTTCTGATGTACCTGATGTTCATGTCCATGTCGTCAATAATAATAATATAAATAATGAACGGATGATGCTTCCGTCTGGTTTCCGATTTTGCCCTTACGATATTGAGCTCATCCGAGACTATTTGATGAAGAAGATTGCCCATCCCCAACTTAATTGGGATCATATTAAACAAGTCCAGCTTTATGACTGTGATCCGTCTCAACTTGCAGGTATTACTTTTTTTTTTTTTTTTCTTGATTTTTTTTTCCTTGGAGTGTTTCTTGATTTTCTTGTTCAGTAATTGAATATTTTTCATTTACCCTTGTTAAGGAATTACAAGAATGAATTATTTTTGAAAATTTTTTTTATGGAATTATTTTTGATATTTTATATAAATTCTTGCGATCATAATCAAAGCTGTAATTCTGTCCATCTATGTTAAATGTTACCCTTATTATGAAATTGGTCTACTTAGACTTGAATTTATTAGGGAGGAAGGGTTTGTCAAAAGCAAAACATTAATTTTCAATAATTTCCTGACAATCTTTATAATATTATTATTATTATTGTTATTATTGTTAAAATGGAATTATTATTAATTATTTCTTTTTGTGCAGCTTGTTATCCAAATGATGAAGGTGAATGGTACTTTTTCACTGAAAGGGACAAAAGATATCCAAACGGCGAGAGACCAAATAGATCAACAAGAAGTGGATATTGGAAAGCAACTGGAGCTAGAAGAAAAATT

>CrNAC-29

AAGTCTTTCAACTTTTGGGTACTACTACTCACTTAAGTAACTAGTACCATTATCCCACTCCCTTTCTTCTTATCTTCTCCCACAATTTGCAGCCCTTTCTTCTCCCCTACTATAAAAACTTGTTCTAATTAGTTATTGATGACCACAAGAATAATTAGTTTATGGCAATTGCAGCAAATATGAGTACTAGCAGCAACCAACAAGATGAAAACAACAACAGCAACAACAAAGATAATGATGAACATGATCAAGATATGGTTATGCCTGGTTTTCGTTTCCACCCTACTGAAGAAGAACTTATTGAATTCTACCTTCGCCGTAAGGTTGAGGGCAAACGCTTCAATGTCGAACTCATTACTTTCCTTGATCTTTATCGCTACGACCCCTGGGAGTTGCCCGGTATTTATTCATTTCCCACTTTCCCCTTAACTTTTTCTAGGACTACTTTTTTTCTCATTTCCTTCCGTTTTTTAAAAACGCGATATGGTGACTTAGGGATTGATTCTTATAACTCGGAAGATGAGGGGTTCAGTGCTTGAACTTTTTTTTTTTTTTTTTATCTGCTCTCACTACTTTTAGATTTTTCTTTTTTTGTCAGCTTTGGCGGCGATTGGGGAGAAGGAATGGTATTTTTATGTGCCAAGAGATAGGAAGTATAGAAATGGAGATAGGCCGAATCGGGTAACAACTTCAGGGTATTGGAAAGCAACTGGAGCTGATAGAATGATTAGAACTGAGAACTTTAGATCAATTGGGTTGAAGAAAACACTTGTGTTTTACTCTGGGAAAGCCCCAAAAGGGATTAGAACTAGCTGGATTATGAATGAATATCGATTGCCTCATCATGAAACCGAGCGTCTACAAAAGGTAATAAAGTCTCAAGTCCAGAAAATGTTTTTCCCTAATTTTAGATAGTTAGTTCTAAATCATCAAATAAAACTTGAACCCATTTCAGATTTCATTGGTTTTCGATGTGGGTTGTCCTTAGATTCAAAGAATTTTAGACAATTTTACCCAAAGATCCAATGAAGATTTCTCAAATGAAAACCCATGAAAATGGATTCCTAGGACCAAACTCTTTTCTCAGTTAATTTTTCTTGCATTTTCTTTCAATGTAAGAGTCTTGAGATTCGGAAGGGATGACAGATCAAGAAAAATTCTATTATGAACTAAATCTTTGTAGACCAGAAGTTTTAAGTAGTTGTACGTGCCTGAAAGTTTAAATTTAGATGAGAAACAAAAGGAACTCAACCTTTTCTTCTTTCTAAAATGAAAACCCATGAAAATGAAATTCCATTGATATCAAGACTTTCTTCTCTGTTAAATTTTTCTGCATTTTCTTTCAACTTAATTAGGGTCTCCTGAGATTTAATCAGCAAAAAAATTCTGCTACGAGTGGCATCTTTGTGGACCAGAAGCTTTAACCACTTAGTACTGAATGTACTGAAAGTTTTTAGTTTTGGTCCATTGAATGAGCACTTTAATGTATGGAGAGAGAGAGAGAGAGAGAGAGAGAGAGAGAGAGAGACATGCAGAAAATTTCACAGAATCCTCGTTTTTTGCCTTAAATTCTCATAACTGCTATTCACGAAACCATCATCATCTTAAGTTCATATTCTAAAATTGGATCATGACACAAAATGACCCAAATTTATTATTTTTCAGGCAGAAATTTCACTTTGCAGAGTATACAAAAGAGCTGGAGTCGAAGACCATCCATCTCTCCCTCGTTTACTTCCAACAAGAACATCATCATCATCTTCAAAAGGAGGAACGCTACCCAATAATAAGAAAAACAACAACGAAACAACAACAACAACAACCACCTCATCATCATCTCCCAATCCCATTGAAAGTTTTCAAAATTTCGTAGGAAATCCAAATCCCCAGAAAATTATAGATGACAAAGTAAGCGAAACGAGTGGAAGCAGTACCACAGATGTCGGAACATCTCTAGGCCTCTCCCAACACATGAGCACCTATATTTCTTTAGCCCCGGCGGCCACCATTACGACCACCACCACATTAGGTCCAGATTGGGCCTGTGTTGGTGGTGGTGGTGGTCAGCCATCTTTCGTGGCGCCCAATTCAGTTGATGATCTTCATAGACTAGTTAATTATCAACAATATCATCATCATCATCATCCCTCACTGATGTCTAGTACTTTACAACCGCCCTTACCTCAGACGTCGTCGTTATCTAATACCTTAGCTCTTAACAATGTGCTTCCACCGGGTTCTCTTCAAGCAGCTTTTAATGATAGATTGTGGGATTGGAACGCGATGTCTGATCAAGCAAGTAAAGATTACAACAACGCGTTCAAATAATTAAAGTGTTCGACGAGATAGTTGTATGTATACATACATATATATAGGTACATTTATATATGTGTACTACTAATATGAATATTTTCTTCTTTTGAGGATATCCA

>CrNAC-23

ATGGAGGGAAATGGTTTTCCACCAGGGTTCAGATTCCATCCAACAGACAGTGAATTACTTGAATATTATCTCAAGAGAAAAATCATGGGACTAAATTTTGATTTTCAACTCATTTCAGAGCTAGACTTATACAAATTTTCCCCTTGGGATCTTCCAGGTAATAAAAACATTAATTTCCTTGAAGTTCAAACATGATAATTGACCTTAAAAATACTTATGTTTTTTTTTTTATTTAAAAAAAAAACCTTTTTTCTTGCAGAAAAAAGCCATTTCCAAGGAACAAACCAGGAATGGTACTTCTTTTGTCCAAGAAACAGAAAATTTGCATCAGGAGGGAGAACGAACCGGTCGAATGAAATCGGATACTGGAAAGTGAGTGGAAGAGATAGAGTTATTTATCATGGAAACAGAGTTTTAGGGATGAAAAAGATTTTAGTTTTCTACATTGGAAGAACACCAATAGGGGAAAGAACTGATTGGATTGTTCATGAATATAAACTTCCAGATAATCAGGAAATTTTGCAGGTAAATTAATCAAATTAGCCCTTAATTTATATGTTCTTGAAATTATTTAGTACTACTAATTTTTCTAACATCTTTTTTTTTTTTTTATGCAGGAAAATAATTCATTTGTACTTTGTAGATTTTTTCATAAGGGTGGTTTAGGTAGAAGAACAGGGGAACAAGTTGTGTCCCTTTCTGAGGAAAATCAGACTGAAAATTTGAAGAAGAATGATCAATGTGGTCCTTCTGATTCAACCCTTTTGTTTGATGAAGAAAAGGGTCTCATTGATTCATACTTTGATTCAGAAAATTGGCAGCTGCAGACACAGAATTCAACAATAGTTCATGCCTATAATCCTCAAGAACGCGATAAGGTTAGTATAAGTAAATAATTAAGAAGAATGATTAGTAGTTTTTGTTTGGATTAATTTCAGAATCAAAAGGGTTTTTTTAACAGATTTCTTCTTACTTTGTGGCAGATTATTGGCAAAAGTGTAGAAAATTCAGTTGGCCTTCAAATCCCAAGAACTGAAGAAATTGCCCTGTTTTCTAATCCTAATAATACTGAAAATGTCTCATTTGATCCAGAAAATTTTGACCCTTTTTTTGAAGAAGATGGCAGTTGGTTAAATGATCTCTTTGATGTAATCCCAGAGGTAAACAAAATCTTGAATTTTTCTTCATTTGACAACTAAAAAGCCTGTTTTAAAAAAAAATTTTTTTTCCCATTTTGATGAACTCAGGAATTTAATGGCCCTGGAGGAGTTTTTGATCTTGGAAATAATACAAATGCAGAAGCTACCCCTCCTTCCTCAGGTACTGCTAAAGAAAGCAGGATTTTAAAAAGCCTTAAATTTGTTCTTTATCAAAGTTGAGATTGATTTTATTTTTTTTCAATCTTGAAGCTCTTTCAGGTAACAAAAAAGAAGGAAAAGACAAAGAATCAGTCTGGGATTTTGTACCAGCACCTCCTGCATCAGCTGCTGAATTGCCGGCGATGAAAAATAGGGTAACAAATGAAGAAGAATCACTAAGAAACTCAAGCAAAATTCATAAGAAGATGAAAACTAAGAAGGCATCATCATCATTATCAGAGGCTAACAAGTTTGTATTTGTCTATGATCTTGCATTGTGGCTTGTTCTTCTTATTGTGGCTGCAAACATTGGCTTCTTTGCTTACAAGATTATATTGTCCTAG

>CrNAC-28

ATGGCAGCAGTTGAAGACATGAACGGTGGCGGCGTCATAGTTAGTGGTGGGAAAGATGAAGAAGATGATGTTCCACTGCCGGGGTTTAGGTTCCATCCAACTGATGAAGAACTTGTAGGGTTCTATTTAAGAAGGAAAGTGGAGAAAAGGCCCATCAGTATTGAGCTTATCAAGCAGATCGATATCTACAAACATGATCCTTGGAATCTACCAAGTAAGTCATATGCTCATCATGTACTAATTTCTTGGCTTGATCGGCCAAGACCTCTCTTATGATATCCTAAAAAGTACTTCTACTACTGAAAGATTTGCCATAACTAATCTTGTATATGTTTAGAAGGCTTTTAGCTAGGTTTCCATTAATTAATGATAAGTGATAATATGTAGGATTCGATTCAACCAGCATATAAGAAGGGTTTTTTCTTTTTTTTTTTTTTTAAGAAAATTAAATAGTGTATTAATATATGTTACTATGATCTAAAATTAGTTTAAGACAAATTTAATGGATTTATGTATTTTGTAAACCCTTTAATTAGCCAGTTTTGGTGCGAGAAAAGTCTCAAAAGTAACAAGTCTAAAATTTGGCCACAAAATGATACCTTTGTTCGAAGAAAATATATAAATCTCTACGAGAAAATCATTAAAGTACATCTCATGATTTTTAATTAATTAGGAAGAGATAATTAAAGGAACCCTTTTTTCTTAATCATATATGAGTATATATCTTTCTGCTAATAATTTGTAACCATCTATCCTTTTAATGAATTCGTCAGATTGTCTCTAATACACCTCTTAATACATTTTTCTTTGTGATACTTTTATATAGTAGTTTTGTTACAAGAAGAAAGTATAACTGCAAAGGGTGTAGGAGAAGATGTAAGAGGATTTTGAACCGTGTGAATTCATAGGGCTTAATTAACTTAATCATATGATCTTGATCAGATTAAGGATATTTATAATGTTTTGGTCATATCATATGTTGTTTTTCAGAAGCAAGTAATGTAGGAGACAAGGAATGGTACTTCTTTTGTAAAAGAGGAAGAAAATATAGGAATAGTATAAGACCAAATAGAGTAACAGGATCAGGATTTTGGAAAGCTACTGGCATTGATAGACCAATTTATTCCGCCGGTGGAGAAGGTCGAGATTGCATTGGCCTCAAGAAATCATTAGTTTACTATCGTGGAAGTGCTGGTAAAGGTACCAAAACTGATTGGATGATGCATGAATTTCGCCTTCCTGCTGATCATGACTTGAAAACCACCAAACATATTGATGCCAAAACCATTGCTCAAGAAGCTGTAAGTTATCCTCTTCTCCTCTTACCCTCAACCCTATTCTCCATGACTTAAAATAAGTTATAAACATATTTTTGAACTATTGTTCAAAGTATTCATAAATATGGAAAAAATATTCCGACGATTTCTTGTAACATTTTGTATTAGTGTTAAGAAAACAGTTGAAATAAAATTATACTCCAGAATATTTTTTAAAATTGTACACATTGTGATGTTAAAAATAAAAGTGTTATATCGAAAATAGTGGTAAGAATCTCATAGTATTAATTATATTTGCAGGAAGTTTGGACATTATGCAGGATATTCAAGAGGAATGTATCATACAGAAAGTGCATGCCGGAATGGAAGGATCATCAACCATCTTCTGCCAAGAAATTAAACCCCAATAATAATAATAATAATATAAATACTGATGCAAGCTCCAAAGCATGCAGTTTTGAGTCTAATAATGATCATGATATTAGACAAACTTACATCAGCTTTAGTACTTCTTCTCCAGTAATTACTACTAATAATAATCAATATGATGAGAGGAAACCCTTGTTATTTAGTGGCCATGCTGATCATATTACAAGGCAGATGTTTATGGGTCAATCTCATCATCATCATCATCATCAAGCTCCAATATCATCAAATGCAACCTTGATGAGTAGTGCATGTTCAGAGGTGACAGATCAATTCTTCAAACACGGAGATTGGGATGAGCTTAGATCTGTTGTGGAGTTCGGTGCTGCTGCTGCTGCTGCTTCTGCTGGTTCGCCCTCCTTCTTGTTGTAAACATAAATTAATTTATATACATATATATACACTACTAACTTTTACATATATCATTATGTGACAAGGAGATAACTCAGTTAGTTAGTAGCACTCCTGATCAGGGATAGATCTTAATTCGATTCACACCCCTATATATGTAATAGGAAAAAAAAAAACTTTAACCTATCATTTTGTTGTGACATGTATAATGAATCTATAATACATATGATATGGATGTTTGCTAAATTGGGATCTATGGGCCTTTTTGTTTAAACTAGGTTGTAATTAACTAGGTAAACGAGGGAGCAGGTTTTGATTAATCATCTGTTTTCTATATAGATTACTAATTTAGGAGTTCTTTTTCTTTGGTTTCTTTATTTTGGACTTGTTCAAATCTGGACTTATTATGGTAAGAATGTTCGGGGGCTTTTGTTATGTCACAGAAGACCTAG

>CrNAC-30

ATGGATGATTCTTGTGCTGCTGATCTTCAATTGCCCGGATTCAGATTCCATCCCACCGAAGAGGAGTTGCTTCAATTTTACCTCAAGAACATGTTACAAGGCAAGAAATTGCATTTTGATATTATTGGTTTTCTCAACATTTACCATCATGATCCTTGGGAATTGCCAGGTAAATTTTCAATCAATTCTAGGTATCTTGTTTTGTTTGTTATTTTGTGTATGCTTTCACCACCACCACCACCACCACAGTCCGTTTCTAATAATTATACTAATTGAATTTGATGAGAGAAGATAGAAGAATGGGAAAGTAACAGGAGTGGGTTTGTTTATGGTTGTGAGTGCAGGGCTGGCAAAGATTGGGGAGAGAGAATGGTATTTTTTTGTGCCAAGAGACAGAAAACAGGGAAGTGGAGGAAGACCAAATAGAACAACAAAAGCTGGATTTTGGAAGGCTACTGGTTCCGATCGTAGAATACTTTGTATATCTAATCCCAAGAACATGATTGGACTCAAGAAAACACTTGTTTTCTACAAGGGCAGAGCACCCCGTGGCTGTAAGACTGATTGGATCATGAATGAATATCGCCTACCTGATACATCCCCAGTCTCCCAGGTATCTTCAATTTTCTAACAATAACATTATTTTTTATATAAGAAGACAGATTTAAAACAAGATTTTTATGTTAAATGTAGGACCAAAACACAACTTAAATACACCTATAATAATAGTTAG

>CrNAC-16

GGCCTTCTTGAAAAAGGGAGGGATTTTACTAGAATCAATTTTTTAAGAACAGAAGAAAAATGGATGAGAAAAATGATGGAGATAAGATTGATGATGTTATGATGCCTGGGTTTCGATTTCATCCAACAGACGAGGAACTCGTGGGATTTTACCTAAAGAGAAAGATTCAGCAACGACCACTTCCAATTGAATTGATTAAGCAAGTCGATATTTACAAATATGATCCATGGGACCTTCCAAGTAAGCAAGCTTTCCTTTTATCTCCTAAACTCAATTCAGTTAAAAAGAATTATAATTTATATTGATTTACTTAAGAACTACTGTCAATGATTTGGATTCTTGGATTGAAACAGAGGTGGCATCGACAGGGGAGAAAGAGTGGTATTTCTACTGTCCAAGAGATCGTAAATACAGGAATAGTGCACGTCCGAATCGAGTTACAGGAGCTGGATTTTGGAAGGCTACTGGAACAGACAGGCCAATTTATTCATCAGATGGTACTAAATGCATAGGATTGAAGAAATCGCTGGTTTTTTACAGAGGCAGAGCTGCTAAAGGAATTAAAACTGATTGGATGATGCATGAGTTTCGTCTCCCTACTTCTGATTCTCCACCACCTAAGAAACTCTTGGACAAGGGCCTTCCTCCCCATGTAAGTTCTCTTCTCACTGAAAAAATTCCATCTTCTTTCAAGAAAAGCTTAATATAAGCTACTCATTTCTTTGAGGCCTTTCTCGTAATGAAGACTTTGTGTATACCAACAGGATGCATGGGCAATTTGCCGGATTTTCAAGAAAACAAATTCAATGGCACAGAGAGCACTTTCTCATGCCTGGATATCATCATCTCCCTTTTCCGAACCATCTTTACCCGAAATATTCCCACAATATTCTCAATTCAGTTCAGAAAACATATCATGTACTACAGAAACAGGATCTAACTTGCATCTCTGCAGCAACAAAAATGACTTAAATCCAACTTCTACCCCCACATTCTCACCTCTGAATTCCATGGTTTCAAAACCATCATTCTGCTGGGCAAATCCAAATGGGGATTTTCCTCCTAATTTCATGTTTTCCCCTCCTACATTCGATGTTTCTTCAATGATTTTCAATCCATCCCCTGTCTTAATCCCAGGAGGAGATGGAAATAATAATAATAATAATAAGGTCCCAGAAAACATTGAATTCGAAAGCTCAAATCAACCACAACATCATCATTTCAACAGTTTTTCATCGCCGGAAATTCAAGAAAGGACAATTGAATTTCCATTCAATTTGCCAACTACAAGTATAGGTGGCGGTGGTGATGATTGGAAGTCTCATTTATCGTGGGATTCTCCGGGAAATTGCAACAGTGAAATTTCTACTACTTATTCTTCCCCAAACAAGTGTTACACATGACATAATCCATTCCCGTCCTGTTCTGTTGATCACTAAAAGATGTTGTAGTTATATAT

>CrNAC-18

ATGGATCAAGAGAATAGAAATGAGAAAATGGAAGAAGTGATGTTGCCAGGGTTTAGGTTTCATCCAACAGATGAAGAATTGGTAGGGTTTTATTTGAGGAGAAAGATTCAGCATAGACCTCTTTCCATTGAACTTATTAAGCAACTTGATATCTACAAATATGATCCATGGGATCTTCCAAGTAAGAAAACAAACCCTTCATTTATGTTCATCTTAATATGGTGCTTGTTTTTTAGATCTTATGCTCAACAGTATAAAAAACATGTGTTAATGAAAAAAAAGTAAAGAAAAAATGTTTCTGAAAAATGAATCATTCTTCATGTTCTCTTATATTCTTTTTTACTGATTTTTAAGTTTTTCTTCGTTGTGTTTGGTTTTTCTTTAGTTGTCTTTTCCTTTGTCTTCAGTGCTTTCCTATATGGTTTTTTTTTATATGTTGGCTAGTTCGGACTCGGAATATTTGACCTAATAAAGAGTGAGTTATTACTTTATCATATTTTTTGAACAGTTTGATCATTTCAAAAGCATTTTTTTCCATCTTTTAACAGTTTCCACGAATTCATGCATGCATATTTAGTTTGCAGGCGATAATTTATATAAAAAAAATTTTAAAATATTGAAAGGGCGAACTTAAAGAAAATATCTACAAATCTAAAACATAAACATTTTCCGGAGATTTTTTAGGAATGCCTCTCCTTTAGTTCTATCCTTGTTTGTTATGAAAAGGAAAATGGAGTATTTCTTATGCTAATTATTATTGGTTTTTTCCTTTTGGAAATAAATATATTCAGAGTTGGCAACAACTGGGGAGAAAGAATGGTACTTTTACTGTCCAAGGGACAGAAAATATAGAAATAGTGCAAGGCCAAATAGAGTAACAGGAGCTGGATTTTGGAAGGCCACTGGCACAGATAGACCTATTTATTCATCTGAAAATTCCAAATGCATTGGCTTGAAGAAATCCCTTGTTTTCTACAAAGGTAGAGCTGCAAAAGGGATTAAAACTGACTGGATGATGCATGAGTTTCGGTTACCTTCAATCAATGATTCTGGACCTGCCAAACGATACTTGGACAAAGACATCCCTCCAAATGTATGTACAAACTTTAAATGTTTTTTATTCTTTTTTTTTTTTTAATGTTTAATCATTTATTCCTTTCGTTTCAATTTATTTGTTACACTTTAAGATAATACACCACTATTAAGGTATGTTTTCCATTTCTTTTGTACATCTTTTAAGCAATAAAAAAAAAAGACTTTTAAAAAAATAAATATATTGTTTTTATTTTCAACAGCATCAAATCAGTAGAAGTAATTCTGAAAAGTATTCTAATAGGAATATAACTTTTCAAAAATAATTTTTTTCTAGATTTTTTAAGCTAAAAGTAGATTAATAAGGTGTAGGAAAATGATTAGATTATTTTAATGGGATAACGAATAGTAGTCTTTTCTTGGAAATATATTTTTTTTGAATCATGTCTAAACACTTAGTTAGTAAATAAGGTAATATGCGAAAAAATTTTATTTAACAATCTCTTGATTTATTGAATCTGGTAGATATTTTGAAACGAAAAAAGTATGTTTAAGTGACAGATAAATTGAAATGTACAGAGTATTATGTATGTGTTAAAATAATTAAGATTAATTAATTATAATCCTTGAACTACATTATTTTTGCAGGATGGTTGGGCTATTTGTAGGATATTCAAGAAAGCTAATTCAACAACACAAAGAGCTCTTTCTCATTCTTGGGTATCTCCAACAGTACCTCATGAAAATGACCAAATATTATTAGGCCAACATATTATTATTGATGGCACTAATAATAATAATAATAATAACATTTTTAATTCAGACAACATTTCTCATGTGACAACCAAAAATAGTTCATCAGTAATCCAATTTGGAGATAGCAGCAGTAATAATGAAGTACAACAATCAATATCTAGTTTTTCTCCATTATTAGATCCTATAAATAATTTCCCTTATAAATCTATAATTACTCATGATCATCACCCAATATTATCATTTCCTAGTAGCATTGAAAATCATGATGAACCACCCTCATCAAAATGTACCACCAATAATATTATTAATGATGCTTCTTCCCTTCTCCTAAACATGTCATCCTCTTTCTTTGGTGATTTTGGAAGATCAAATTACAAAGATTTTGCAGAATTCGGCCCCGTTTCATCATCGATATCATCTCAAGATCAACAGTGTAACAATTTCTTGATGACATTACCTCAAGATATGATCATGAGAAGATCAAACTTTGATGATCAAGAAGATCAGACGGCTCTGATGAGTGCATCTCATCAAAAGGATGATCATCAATGGGGAGTTCCCATAAATTTGCCAATATTGAGTATGGGTGATCAAGCATGGAAATCAAATTTGATATGGGATTCTTCTTCACCTTGTCCTAGTAGTGATATTTCCACCACTTATTCTACTAACAAGTGCTATTCTTGA

>CrNAC-21

GTTCACGCCAAGTGTTCGTCCATATTATATTCCTTCTCTATTCTAAAGTCCCCCCCCTACCCTTCCTCCCATTGCCATTACTATAAAAAAGAAGCCTCACTTTCCTATCTTTCCCACGTACCCCCACTTCTCCTTCTTCTTCTTCTTTGCCTTTCATGAACTAATTAATTGCAAGACTGCTCCATTTTTTGAAATTGGAATCAATCGAGAGAAACAATTCTTGATAAATTTATGGAGAAGCAATTAAACTTTGTCAAGAATGGTGTCCTCAGATTACCACCTGGATTTCGATTTCATCCAACTGATGAAGAACTTGTTGTTCAATACTTAAGACGTAAGGTCTTTTCTTGTCCTCTGCCAGCTTCGATTATCCAAGAAGTTGATGTTTGTAAATCTGATCCTTGGGATTTGCCAGGTTGGTCCTTGATTAATTCTTCATTAATTCTACATTTCCCCAGTTAAAATTCTCAATATTTAGCATTCTCAAAACCAATAAAGTTGGAAGAAGAATTCTGCTTAGCTTAGAAAACCAATAAATCCTGGTTAATAGAATCTCAGGATATATGGAATATCGGAAAATTCTGTTATGGATAAGTTTGATGTCATGATAGAATTTTTCCATATCTCTAACAAGCATAATCAAATTCTTGGCTAATTAGTGGAAATGTGGCAAATTTTCTCTGTTTTTTTCCATTTGGGATTCCTTGGAAAATTTGTTCTGATTCTTGGTAATGGTGAAATTGATTAGGTGATTTGGAGCAAGAGAGGTACTTTTTCAGTACTAGGGAGGTAAAGTACCCAAATGGAAACAGATCAAACAGGGCAACTGGTTCTGGCTACTGGAAGGCAACTGGTGTAGACAAACAAATTGTAACATCTAAGAGCCACCAAGTAGTTGGCATGAAAAAAACCCTTGTTTTTTACAGAGGAAAGCCACCAAATGGGACCAGAACTGATTGGATTATGCACGAGTATCGCCTTGTTAATGTCCAAGAAACCGTTGCTGCTGCTGCCTGCCCCCATCAGCACTTAGCTCAGGTGTGTATATTCAAGAACAAGNNNNNNNNAAAAAAAAAAAAAAGAAAAAATCCGTTCTTTGTTATGTTTCGTGTTGTTGTTCTATGTCTTTTGTTCTGACACTGTTTTTTGTTTTTGTTGGTTGATTTTTTTGTTTAGGATAATTGGGTTCTGTGCCGCATATTTTTAAAGAGGAGAAGTGGGAAAAAGGATGAAGAAATGGTGAAGTCCCAAAACTGCAAAACAAAATCAGGGGAAATGCAGGGGAAAAAATCTGGGGTTGTTTTCTATGATTTCATGGCTAAGGAAAGAACTGATTTAAATCTAGCACCAGTTTCATCATCTTCGGGTTCCAGTGGAATTACAGAGGTTACTAGCAATAATGATTCAGATGATCATGAAGAAAGTAGCAGTTGCAGTAGTTTTAGTGCTTCAGTTAGAAGAAAAACTTGGCTTTAATTTTACAACTTTTTCATCAGAATAACTAATTTAGAGTTTTTTTTTTTCCCGTTTTAACTTCTCCAGTAAAAAAAAATGAACGC

>CrNAC-42

GAGGCAAATTTTACAATTCCAATTTTTCTCACTTACCCATTTCTCACTCTATATATCATCATTATGTATAAATAGGCCAATCTCTCCCTCCCTTTCTTCCCATACAGAGATTGGATTTGATTCGTCTTTACAGAGACAATATTAATAACTCTGTTCTTCTCTCTTCTGTTAAAGGGCTCTGTTTTTTTTCTGGTCTTAATTAGAGATATGGATAGTATAATAAGAGTTCCAATTGGGTTCAGATTTCGTCCTACAGATGAAGAGTTGATCATTCACTACTTGAAAAGAAAGGTTTTGTCTCTGCCTTTTCCTGCTTCCATTATACCTGAATTCCATGTTTTCCAAACCAATCCTCTTCACTTTCCAGGTAATAATCAATGATCCTTTCTTCCTGTTCTTGTTCTCAATTATTAATAAATTTCAAGCTTGATTTATGAATTTCAATCCACCTATTGCTTGTTAAAAAGGTGACCCGAGGGAGAATAGGTATTTCTTCTGCAACAGAAAGGTTTTGCCCATATCTACTATTGTCAGTGTCAGGGATGGTTCTGGGTACTGGAAACCCACCGGCCGCCAACGGAAAATTATATCCCCCGCCGCTAACAACCGAGTAGTGGGTACAAAAAGATCCCTGGCGTTCTACCATTATCAGGGGAAACAAAAACATGGCCATGGTTTAATGACTGATTGGGTTATGGATGAATACTGTCTATTGGCCCCAGATGAACAGCAGCAGGTATAGTATACTTCAATCAATCGATCAATCAAAAAATGGGTTGTAATTAAATTCTTGTTTTACAATTTGATTTTTTTTTTTCCCAGAATGTTCTGCAAATTGGAGACTGGTTTGTGTACTGCATACGCAGGAAGAAGAGGAAAACCAAGAATCAATATTCTTCAAGAAAGGATGAATCTTCTTCATTATGTTTAAGTGAAATAATTGATGTTTCTTGCAATAATGAATTAGATCAGGAAGCTAACAGTGAGTTTTAATTAGATCAGAAATTCTGTTGTTTGGAAACAAATTCTCCAAGTTGCTTAAAGAGTAAGCAGAAACTATGGCCAGTATAATAAAGGCAGAGTAGCAAATGCTTTTGGAACAAAAAGGCTAAATAATTTTCAGAATGTCATAAGATTTTATTATGCAAAGATGAAAGAAATTCAAGAATCCAATTTTTAT

>CrNAC-43

ATGGTTGCAGAGGAGAAGGGCAAAAAGAGAAATTATTGTGGAGGAGAATGGAAACAACATAGGATGAAAATACCAATGGGTTACAGGTTTATACCAACTGATACTGAATTAATATTGGATTATTTGCTTCAGAAAATTATTGGTAAACCACTTCCTGCTGACATAATTCCACAGATAGATGATCTCTATAGAATAGATCCACAACAACTCCCACTAGGTATGCTTATATATGATCAACAAAATAATGTTTCTTGCTATGGTTTTTGTTACTGTTTTTGTGCCCAAAATATGCCTCAATTGTTTTAGTAGCAAAATATTCAAGTAGTATTTTTTTTTTAATGTGTTTTAAATTAGCTATACAACGACTAAACTTGACATATAAAAGTATATGCAACTCGTGTGACACCAATTTCAAGAATAAAAATGTTTAGATATGTCATTCTCCCGTTGAGTATATAGGTTTGATCAAATATCTCATTAGGTGATTAATAGTTGGAATAATTGACGTGTTAGAACAATTTTATTTTTGAAATTGATGTCATACATAAGCTCTATTGATGTGGTTTTGATAGTTTTAGTCTTGTACCCTTAGATTAGATTTTAAACTCGAAAATTATTTACGTTTCTAAAATTTTTTATTCATAAATAATTTATCTTTTACATATTTCGAGTAAAAAATCTCCGCTTGTTACATTCTTATTTCTAATCTTTTAATATTTAATTATTACTTTTTGTGCTTGATAAGTTAATTTTTAGGAAATTCTGTTATAAATCATATGTTATGGATCTCTTAACTTGTATTAACTATCTCACCCTCTAATTATTGATTATGTCATCAATTAAGGGTGGGGATATAAAACTTTGTCAATAATATAATATTCTTAATTTTTATTGATTGGGAGCTAAATATATTATTTTACACAAAATTAACATAATTTTATACCGCATATAAAAATATAAAATAAAATTTAGGCTCTAATATAATTTTGCTCATACATACACACGACTTGAATTTGTTTGTATAAAAAAACTGTGCATGATGGTAGCAAACTATGTTACTTTAGTTCGTAGGTATAAAATTAATTAAAAAAAAAATCACTTTTTGAATATTTCGTGAGGTGAATAAATAGTTGACGGAAGAATTCCATGTGAAACTATCATTAGTATCTAATGCAGCCAACTTTTTTGTTTTTAGGCAAATATCAGTACTGGAAGGAGAATGAAGCATACTTTTTTACCCACCAACATCAAACCTATTTGCCAGGAGATAGAATGCCAAATGGTTTCTGGGAGGCTGATAAGAAAGATGAATACATTCTTGATGAACACAACCTGCAGATTGTTGGCTTTAAAAGTACGTTTACGTTTTATCGCGTAATAGAAAGAAAAGAAGAGGAGACCGATTGGGTAATGACTGAATTCAAAGTTCATTCAACCCTAATCCCTTATGATGCAGCAGATGAGAATCTTATGCTATGCAAAATTGAATATAACCCTCAAACAGAAGAATGA

>CrNAC-40

TCTGTCTGTGGGTTGTTTGATTGTGTTATTGCTACTGAGTGATCAAAACACACCTTCTTTTTCACGTCAGTACTCATCTCTTTCTTTCTTTCTCTCTCTCTCTCTCTCTCTATAAATATCAATCTCACTTCTCTTAGAAACTCAATAACACTAGTTCAAATTCTTCACCATTATATACATACATATATATACATATATCCAATTAGTTGTATACGTACTTGTCGATTTCATGGGAGACGACAATGTGAAGCTGCCACCCGGATTTCGATTCTATCCTACCGATGAAGAACTAGTCGTCCATTTCCTCCACCGTAAAGCGGCCCTCTTACCTTGCCATCCTGACGTCATCCCCGACCTTGATCTCTATCCTTATGATCCTTGGGACTTGGATGGTAATTATTTCATTTTTCCTCTTTCTTTTTTTTATTAGCTCCAAAAAGTGTTTCATATTCATAAATTATAATGCACAAAGCTTAGCCAATTAATTACTAAATACATGATCAATACTTGTTAGTTATATATATATATATGCATGATGAATATTTAATATTGATGAATTATAATATGTAATTTCAGGTAAAGCAATGGTGGAAGGGAATAAATGGTACTTTTATAGTAGGAGGACGCAAAGCAGAATCACGGGTAACGGATATTGGCAACCGTTGGGAGTGGAAGAACCAATATTCTCCATGACTTCCGGCCAAAAAGTTGGCATGAAGAAATTTTACGTGTTTTACATTGGTGAACCACAAGAAGGTGCCAAAACCAACTGGATAATGCAAGAATATAGACTTAGCTCCGACAGCGGTTCCACCAGTAGCAGTAGTAGTAGTAGTAGATCCTCCAAGAAAAGACATTCCAAATTGGTAAGTTATAAAAACAACCACCACAAAACACTCATCTCCTCTCCTCTCCTCTCCTCACACTATTTGGTTATCAAAATGTATCTATCCTCTCATAGTCAATTATTTTATATTTTCTATATATTTGTTTCCTCCTAACATTTTAAATATATATACTAAAATGTATTTTGGTATGGTGGCAGGATTATAGCAAATGGGTAGTATGTCGAGTGTACGAACGCAACAATGACGATGATGACGACGATGGCACAGAGCTTTCATGCTTGGATGAAGTTTTCTTATCTTTGGATGATCTGGAGGAGATTAGCCTGCCAAACTAGCTAATTACCAATATTAAAATTAGCTATACTATAAGTTAGATTGGGCCTAATTAACCCCCACCATATCCCAAATTATGTAGAAATTAGTTAATATTTTGGCATTGATCATGTTTTTTTTTTAATTTTCTTTTTCTTAA

>CrNAC-45

ATGTTGCCGGTGGGCTATAGATTTGCACCCACTGATGAAGAATTGATAAAGTATTATCTGGCCAATAAAGTCTTTTACAAACCTGTTCCAGTTAAAATCATCCGAGAAATTGATGCTACTTTTCTTTATAGTAAATCTCCAAACACTTTATGTACGTTTCATATTTTATATTATATTATATGATCATCATTAAATATTTTTTCATTTTGTTCGTTCACATTTAATTTCTTTTTTAGGAAATTGATGATATATATTTTTTAATTTTGGAGACAGTGGGAGATCCATATATTGAAAAAGAATGGTTTTTCTTTGTATATAAAGATGAATATTTTCGAGGGAAAATTATGAGGAATCGTCGGGTTGAAGATGGGGAAGGTTTCTGGCAATGCATTGGAGGGGAAGAGCCAATTTGCAACTCTAATGGACAAGTATTGGCTTACAAGATTCATTTGACCTATTTTTCAGGACCAATAACTAATGGCAAGAAAACAAATTGGAGAATGGAAGAATATAGGTTACTATTTGAATGTAATACAACAAATACACAAGAATCCTCAGAGGTATGTATTTTATTTTTCTTTTATTCAACATATTTATGTTTTTTGTTTTTTTTTTTTTTTTTTCGTACAAGTGATTTTTATTTTTATTTTTTCAGGCAGAATGGATACTGGGGAGAATTGTAA
