# Supplementary material for: Genome-wide identification of NAC transcription factors and regulation of monoterpenoid indole alkaloid biosynthesis in Catharanthus roseus
Source: Front Plant Sci. 2023 Dec 20;14:1286584. doi: 10.3389/fpls.2023.1286584 (PMC10785006; doi:10.3389/fpls.2023.1286584)
Supplement: Supplementary file 4 [file DataSheet_4.docx]

>CrNAC-01

PPGFRFHPTDEELVVHYLKKKAASAPLPVSIIAEVDLYKFDPWELPAKATFGEQEWYFFS

PRDRKYPNGARPNRAATSGYWKATGTDKPVLTSGGTQKVGVKKALVFYGGKPPKGIKTNW

IMHEYR

>CrNAC-02

PPGFRFHPTDEELIMYYLKNQATSRPCPVSIIPEVDIYKFDPWELPEKTEFGENEWYFFT

PRDRKYPNGVRPNRAAVSGYWKATGTDKSIYSGSKYVGVKKALVFYQGKPPKGIKSDWIM

HEYR

>CrNAC-03

PPGFRFHPTDEELVNYYLKRKIHGQEIELDIIPEVDLYKCEPWELAEKSFLPSRDPEWYF

FGPRDRKYPNGFRTNRATRAGYWKSTGKDRRVSSQNRPIGMKKTLVYYRGRAPQGIRTDW

VMHEYR

>CrNAC-04

PPGFRFHPTDEELVGYYLKRKTDGLEIELEVIPVIDLYKFDPWELPEKSFLPKRDKEWFF

FCPRDKKYPNGSRTNRATRSGYWKATGKDRKVVCQSAVIGFRKTLVFYRGRAPLGDRTDW

VMHEYR

>CrNAC-05

PPGFRFHPTDEELITHYLSPKVLDNSFSAIAIGEVDLNKVEPWDLPWKAKMGEKEWYFFC

VKDRKYPTGLRTNRATDAGYWKATGKDKEIFKVKSLVGMKKTLVFYRGRAPKGEKTNWVM

HEYR

>CrNAC-06

PPGFRFHPTDDELVTHYLCRKCAAQPISVPIIAEIDLYKFNPWDLPGMALYGEKEWYFFS

PRDRKYPNGSRPNRAAGTGYWKATGADKPIGKPKPLGIKKALVFYAGKAPKGIKTNWIM

HEYR

>CrNAC-07

PPGFRFHPTDEELVIHYLCRKCASQPIAVPIIAEIDLYKYNPWDLPGMALYGEKEWYFFS

PRDRKYPNGSRPNRAAGSGYWKATGADKPIGNPKPMGIKKALVFYAGKAPKGEKTNWIM

HEYR

>CrNAC-08

PPGFRFHPTDEELVDYYLRKKIAAKRIDLDVIKDVDLYKIEPWDLQELCKISNDEQNEWY

FFSHKDKKYPTGTRTNRATKAGFWKATGRDKAIYSKHSLIGMRKTLVFYKGRAPNGLKSD

WIMHEYR

>CrNAC-09

PPGFRFYPTDEELLVQYLCRKVAGHHFNLQIIGEVDLYKFDPWVLPSKAIFGEKEWYFFS

PRDRKYPNGSRPNRVAGSGYWKATGTDKIITTEGRKVGIKKALVFYVGKAPKGTKTNWIM

HEYR

>CrNAC-10

PPGFRFHPTDEELITHYLSKKVLETNFSAKAIGEVDMNKVEPWDLPCKFFNPNKKKGRAK

MGEKEWYFFCVRDKKYPTGLRTNRATAAGYWKATGKDKEIFRGKSLVGMKKTLVFYKGRA

PKGEKTNWVTHEYR

>CrNAC-11

PPGFRFHPTDEELITYYLINKISDSSFTGRAVADVDLNKCEPWDLPGKAKMGEKEWYFFS

LRDRKYPTGVRTNRATNTGYWKTTGKDKEIYNSVSSELVGMKKTLVFYRGRAPRGEKTNW

VMHEYR

>CrNAC-12

PAGFRFHPSDEELIVHYLGKKANKFPLPASIVAEVELYKFNPWDLPKKCSFGDEEWYFFT

PRDRKYPNGMRPNRMAGSGYWKATGIDKPIVASCGSKVIGVKKALVFYTGKPPKGNKTDW

TMHEYR

>CrNAC-13

PGFRFHPTDEELVRYYLRRKICSKPFRFDAISEIDIYKAEPWDLPGMSKLKTRDLEWYFF

SVLDKKYGNGSRTNRATDRGYWKTTGKDRPVYHKSQVVGMKKTLVYHSGRAPKGQRTNWV

MHEYR

>CrNAC-14

PPGFRFHPTDEELVGYYLRKKVASQKIDLDVIRDIDLYRIEPWDLQDKCRIGYEEQNEWY

FFSHKDKKYPTGTRTNRATIAGFWKATGRDKAVYDKSKLIGMRKTLVFYKGRAPNGQKTD

WIMHEYR

>CrNAC-15

PPGFRFHPTEEELLHYYLKKKIAHEKIDLDVIPDVDLNKLEPWDIQEKCKIGSTPQNDWY

FFSHKDKKYPTGTRTNRATAAGFWKATGRDKVIYCNSRCRIGMRKTLVFYKGRAPHGQKS

DWIMHEYR

>CrNAC-16

PGFRFHPTDEELVGFYLKRKIQQRPLPIELIKQVDIYKYDPWDLPKVASTGEKEWYFYCP

RDRKYRNSARPNRVTGAGFWKATGTDRPIYSSDGTKCIGLKKSLVFYRGRAAKGIKTDWM

MHEFR

>CrNAC-17

PPGFRFHPTEEELLHYYLRKKVASKKIDLDVIRDVDLNKLEPWDIQEKCRIGSTPQNDWY

FFSHKDKKYPTGTRTNRATAAGFWKATGRDKVIYSNSKRIGMRKTLVFYKGRAPHGQKSD

WIMHEYR

>CrNAC-18

PGFRFHPTDEELVGFYLRRKIQHRPLSIELIKQLDIYKYDPWDLPKLATTGEKEWYFYCP

RDRKYRNSARPNRVTGAGFWKATGTDRPIYSSENSKCIGLKKSLVFYKGRAAKGIKTDWM

MHEFR

>CrNAC-19

PGFRFSPTDEELICYYLKKKLEGSDKCVEVIPEIDICRHEPWDLPAKSIIQSDNEWFFFS

PRGRKYPNGSQSKRATACGYWKATGKERNVKSGSAVIGTKRTLVFHTGRAPKGQRTEWIM

HEY

>CrNAC-20

PGFRFHPTDEELVRYYLRRKACGKPFRFQAVSEIDVYKSEPWELACFSSLKTRDLEWYFF

SPVDRKYGNGSRLNRATGKGYWKATGKDRPVRHKNQTIGMKKTLVFHSGRAPDGKRTNWV

MHEYR

>CrNAC-21

PPGFRFHPTDEELVVQYLRRKVFSCPLPASIIQEVDVCKSDPWDLPGDLEQERYFFS

TREVKYPNGNRSNRATGSGYWKATGVDKQIVTSKSHQVVGMKKTLVFYRGKPPNGTRTDW

IMHEYR

>CrNAC-22

PPGFRFHPTDEELLHYYLKKKVSFQKFDMEVIREVDLNKIEPWELQERCKIGTTPQNEWY

FFSHKDRKYPTGSRTNRATNAGFWKATGRDKCIRNTFKKIGMRKTLVFYRGRAPHGQKTD

WIMHEYR

>CrNAC-23

PPGFRFHPTDSELLEYYLKRKIMGLNFDFQLISELDLYKFSPWDLPEKSHFQGTNQEWYF

FCPRNRKFASGGRTNRSNEIGYWKVSGRDRVIYHGNRVLGMKKILVFYIGRTPIGERTDW

IVHEYK

>CrNAC-24

PPGVRFHPSDEELIVYYLLNKLNSLPLPAAVIAEVELYNYNPWDLPKKALFGEDEWYFFS

PRDRKYPNGARPNRTAASGYWKATGTDKPILNSCGGERIGVKKALVFYIGKPPNGSKTDW

IMIEYR

>CrNAC-25

PPGFRFYPSDEELVCHYLYKKIANGGEVSKDTLVEIDLHTCEPWQLPEVAKLNSTEWYFF

SFRDRKYATGFRTNRATTTGYWKATGKDRTVIHPVTRSVVGMRKTLVFYKNRAPNGIKTG

WIMHEFR

>CrNAC-26

PVGYRFRPTDEELINHYLRLKITGFDKEVNIIREVDICKLEPWDLPDLSLVESYDDEWF

FCPKDRKYQNGQRLNRATLKGYWKATGKDRNIVSRKGVKIGMKKTLVFYTGRAPDGKRTN

WVIHEYR

>CrNAC-27

PPGFRFHPTDEELLYYYLRKKVSYEPIDLDVIRELDLNKLEPWDLKDKCRIGSGPQNEWY

FFSHKDKKYPTGTRTNRATMAGFWKATGRDKAIHLSTALASKRIGMRKTLVFYIGRAPHG

QKTDWIMHEYH

>CrNAC-28

PGFRFHPTDEELVGFYLRRKVEKRPISIELIKQIDIYKHDPWNLPKASNVGDKEWYFFCK

RGRKYRNSIRPNRVTGSGFWKATGIDRPIYSAGGEGRDCIGLKKSLVYYRGSAGKGTKTD

WMMHEFR

>CrNAC-29

PGFRFHPTEEELIEFYLRRKVEGKRFNVELITFLDLYRYDPWELPALAAIGEKEWYFYVP

RDRKYRNGDRPNRVTTSGYWKATGADRMIRTENFRSIGLKKTLVFYSGKAPKGIRTSWIM

NEYR

>CrNAC-30

PGFRFHPTEEELLQFYLKNMLQGKKLHFDIIGFLNIYHHDPWELPGLAKIGEREWYFFVP

RDRKQGSGGRPNRTTKAGFWKATGSDRRILCISNPKNMIGLKKTLVFYKGRAPRGCKTDW

IMNEY

>CrNAC-31

PLGFRFRPTDVELIDHYLRLKINGRHSEVQVIPEVDVCKWEPWDLPRTDTLLLLTGLSVI

KTDDPEWFFFCPRDRKYPNGHRSNRATDAGYWKATGKDRTIKSRKSSPSGQSNPQLIGMK

KTLVFYRGRAPKGERTNWIMHEYR

>CrNAC-32

PPGFRFHPTDEEIISYYLTEKVMNSGFSAKAIGEVDMNKCEPWDLPKRAKMGSEKEWFFF

CQRDRKYPTGMRTNRATESGYWKATGKDKEIYNSNNNKSGKGVGGGGNCVVVGMKKTLVF

YKGRAPKGEKSNWVMHEYR

>CrNAC-33

PPGFRFHPTDEELVLYYLKRKICRRRHRLDVIGETDVYKWDPEELPGTTIDTEFRFDLIF

DKISKLKTGDRQWFFFSPRDRKYPNGARSNRATRHGYWKATGKDRIITCNSRPVGVKKTL

VFYRGRAPTGERTDWVMHEY

>CrNAC-34

PPGYRFCPTDAELILHYLEKKIKNEKLPPHRIQEENLYKFTPDAISEMYPVLGEREWYFF

TPRDRKYSNGTRPNRAAGTGYWKATGADKPIRNSGSTIGFRKALVFYEGKPPKGDKTNWI

MHEYR

>CrNAC-35

PPGFRFHPRDEELICDYLMKKVVGGGGGCDEDQVQRYPGVRMVEVDLNKSEPWEIPESAC

VGGKEWYFYSQRDRKYSSGLRTNRATATGYWKATGKDRAVFRKAKLVGMRKTLVFYQGRA

PKGRKTDWVMHEFR

>CrNAC-36

PPGFRFHPTDEELVDYYLRKKVTSRRIDLDVIKDVDLYKIEPWDLQELCRIGTEEQNEWY

FFSHKDKKYPTGTRTNRATAAGFWKATGRDKAI

>CrNAC-37

PGFRFYPTEEELVSFYLRKKLEENIREPNLLDRVIPVIHIYEFEPWLLPKLSGELCSGDS

EQWFFFVPRQEREARGGRPNRTTASGYWKATGSPNYVYSSNNRVIGVKKSMVFYRGKAPT

GKKTTWKMNEYR

>CrNAC-38

LPSKDLEWYFFSPRDRKYPNGSRTNRATKAGYWKATGKDRKVNSQMRAVGMKKTLV

YYRGRAPHGARTDWVMHEYR

>CrNAC-39

PSGFRFCPYDIELIRDYLMKKIAHPQLNWDHIKQVQLYDCDPSQLAACYPNDEGEWYFF

ERDKRYPNGERPNRSTRSGYWKATGARRKIVDNEGIEIGNKRPLVFYQGKHKDNRERSSK

EEPKKTDWIMYEYQ

>CrNAC-40

PPGFRFYPTDEELVVHFLHRKAALLPCHPDVIPDLDLYPYDPWDLDGKAMVEGNKWYFYS

RRTQSRITGNGYWQPLGVEEPIFSMTSGQKVGMKKFYVFYIGEPQEGAKTNWI

MQEYR

>CrNAC-41

PGFRFYPTEEELVSFYLKNKLEGLRVEEINMVIPVLDIYHYNPWELPKYAGKYSRKDP

EEWFYFIPMQEKESRGGRPNRQTNEGYWKATGSPGYVYSLKNNKIIGGKRTMVFYNGRAP

YGKKTPWKMNEYK

>CrNAC-42

PIGFRFRPTDEELIIHYLKRKVLSLPFPASIIPEFHVFQTNPLHFPGDPRENRYFFC

NRKVLPISTIVSVRDGSGYWKPTGRQRKIISPAANNRVVGTKRSLAFYHYQGKQKHGHGL

MTDWVMDEY

>CrNAC-43

PMGYRFIPTDTELILDYLLQKIIGKPLPADIIPQIDDLYRIDPQQLPLGKYQYWKENEAY

FFTHQHQTYLPGDRMPNGFWEADKKDEYILDEHNLQIVGFKSTFTFYRVIERKEEET

DWVMTEFK

>CrNAC-44

PSGFRFCPYDIELIRDYLMKKIAHPQLNWDHIKQVQLYDCDPSQLAACYPNDEGEWYFFT

ERDKRYPNGERPNRSTRSGYWKATGARRKI

>CrNAC-45

PVGYRFAPTDEELIKYYLANKVFYKPVPVKIIREIDATFLYMGDPYIEKEWF

FFVYKDEYFRGKIMRNRRVEDGEGFWQCIGGEEPICNSNGQVLAYKIHLTYFSGPITNGK

KTNWRMEEYR

>CrNAC-46

KIKKKKLPRNLILHENLYHDPPDEITGRYKELTTGNEWYFFTPRDGKYPNGDQPSRVAGS

GYWKAIRADKPIKHNGNEIRFRKALVFYQGKPPKGEKTSWIMHE

>CrNAC-47

PPGARFYPSEEQLVYYYLSSKNDGSNYYGIDVIREIDLYSYDPFNLPEISCFRFGRGGRR

RHWYCYVGRIIRERGRRRAGSGYWKKRGKVKDIVGGGAAEKIAMGTRKSFVFYLGDS

KTWVMEY
