# Supplementary figures and images for: Genome-wide identification of NAC transcription factors and regulation of monoterpenoid indole alkaloid biosynthesis in Catharanthus roseus
Source: Front Plant Sci. 2023 Dec 20;14:1286584. doi: 10.3389/fpls.2023.1286584 (PMC10785006; doi:10.3389/fpls.2023.1286584)

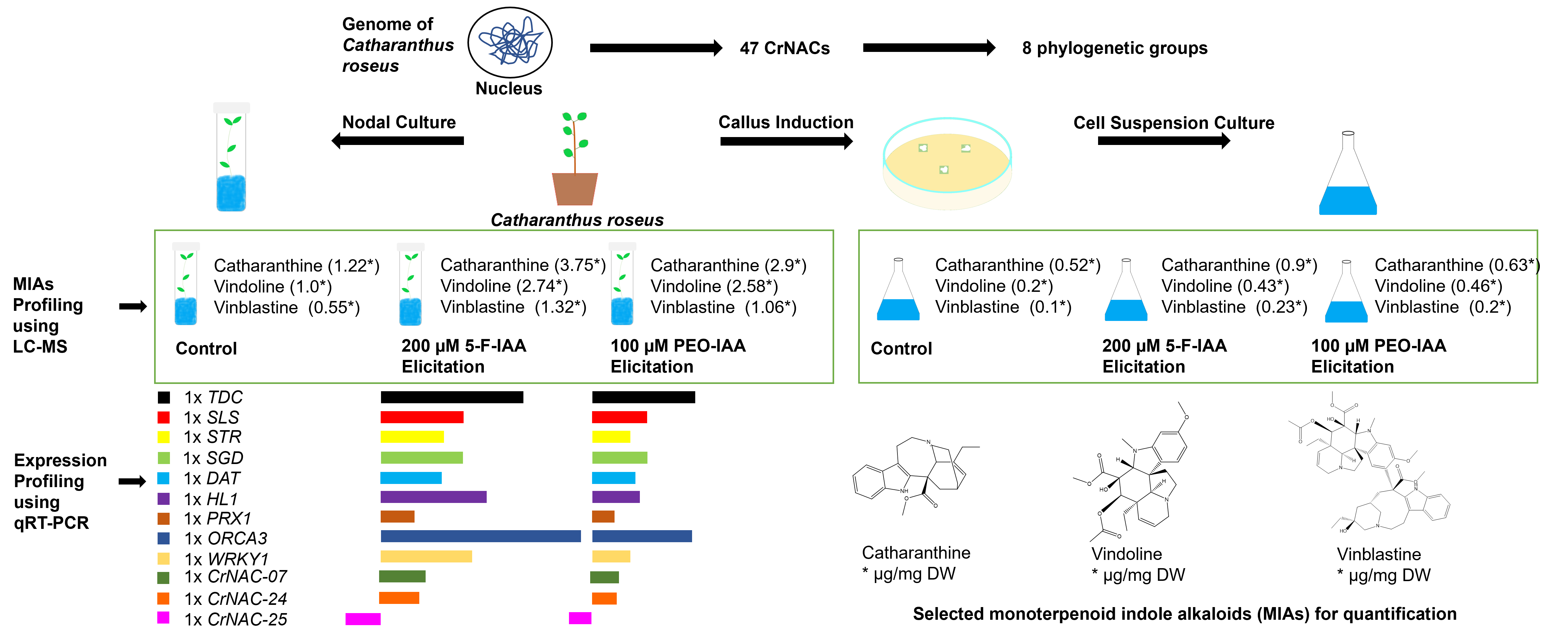

Supplement: Supplementary file 5 [file Image_1.tif]
